# Stereoselective Allylic Alkylations of Amino Ketones and Their Application in the Synthesis of Highly Functionalized Piperidines

**DOI:** 10.1002/chem.202000051

**Published:** 2020-02-21

**Authors:** Cynthia Prudel, Kai Huwig, Uli Kazmaier

**Affiliations:** ^1^ Organic Chemistry Saarland University, Campus C4.2 66123 Saarbrücken Germany

**Keywords:** allylic alkylation, chelates, enolates, ketones, palladium, piperidines

## Abstract

**Chelated ketone enolates** are excellent nucleophiles for allylic alkylations. Electron‐withdrawing groups on the allyl moiety allow subsequent intramolecular Michael additions giving rise to piperidines with up to five stereogenic centers.

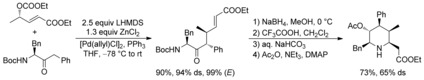

## Introduction

**Figure 1:**
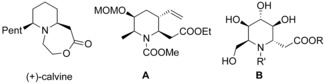
Natural occurring homopipecolic acid derivatives.

Piperidines are widespread found in nature, for example, as alkaloids, and are privileged structures in medicinal chemistry.^1^ Amino acids derived from piperidines, such as pipecolic acid and the elongated homopipecolic acid are also common, for example, in peptidic natural products and peptidomimetics.^2^ Calvine, a piperidinic lactone isolated from a ladybird beetle of genus Calvia^3^ can be seen as a bicyclic homopipecolic acid derivative (Figure 1). Highly substituted homopipecolic acid derivatives, for example, **A** have been used as synthetic intermediates in the synthesis of decahydroquinoline alkaloids such as the lepardins,^4^ while polyhydroxylated homopipecolic acids such as **B** act as glycosidase inhibitors.^5^

**Scheme 1:**
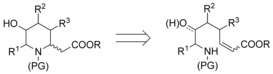
Homopipecolic acids via intramolecular aza‐Michael addition.

Therefore, it is not surprising that a wide range of protocols have been developed for the synthesis of piperidines in general, and also for homopipecolic acids in particular. Whereas these approaches are generally applicable for the synthesis of 2,6‐disubstituted^6^ or 3‐hydroxylated piperidines,^7^ methods for the synthesis of higher substituted piperidines are significantly less developed.^8^ Many procedures advanced for β‐amino acid syntheses^9^ can also be applied for homopipecolic acids. Besides homologation of the corresponding pipecolic acids using the Arndt–Eistert protocol,^10^ 1,4‐additions of amines are also very common. While an intermolecular amine addition requires a subsequent cyclization step,^11^ the intramolecular version (Scheme 1) is more straightforward, but the stereochemical outcome of the cyclization step is difficult to control.^12^

**Scheme 2:**
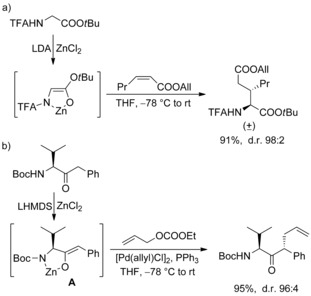
Michael addition and allylic alkylations of chelated enolates.

Our group is involved in the synthesis of amino acids using chelated enolates as thermally stable and highly selective nucleophiles.^13^ These enolates can be used in a wide range of reactions, such as transition‐metal‐catalyzed allylic alkylations^14^ or Michael additions, for example, towards nitroalkenes^15^ or α,β‐unsaturated esters (Scheme 2 a).^16^ Allylic alkylations cannot only be used for the synthesis of γ,δ‐unsaturated amino acids, but also for the modifications of peptides in a highly stereoselective fashion.^17^ Recently, we could show that Pd‐catalyzed allylic alkylations can also be performed with chiral chelated α‐aminoketone enolates with excellent diastereoselectivity (Scheme 2 b).^18^, ^19^ The stereochemical outcome of the reaction is mainly controlled by the enolate geometry and the sterical bulk of the side chain, which shields one face of the enolate in the allylation step. Especially good results are obtained with arylated enolates. Here the (*Z*)‐enolate **A** is formed almost exclusively, avoiding 1,3‐allyl strain^20^ between the aromatic ring and the side chain, which would be significant in the (*E*)‐enolate.

Our goal was now to use this approach for the synthesis of highly substituted homopipecolic acids and related structures via subsequent intramolecular aza‐Michael additions. Therefore, an electron‐withdrawing functionality at the double bond is required.

## Results and Discussion

**Scheme 3:**
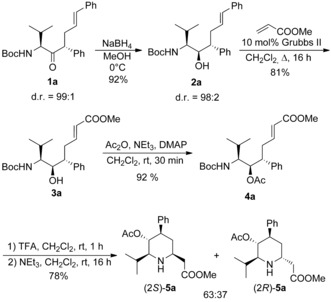
Synthesis of tetrasubstituted homopipecolic acids **5**.

We started our investigations with the previously synthesized unsaturated α‐amino ketone **1 a**,^18^ which could be reduced to the corresponding alcohol with excellent selectivity (Scheme 3). The configuration of the new formed stereogenic center was determined by NMR of the corresponding oxazolidinone, which was obtained by treatment of **2 a** with base.^18^ For the introduction of the required electron‐withdrawing group we decided to use a cross metathesis with Grubbs II catalyst.^21^ 2.5 Equivalents of methyl acrylate and 10 mol % of catalyst were necessary for complete conversion. The excess of acrylate was required, because the styrene formed during metathesis can also react with the acrylate in cross metathesis. Acetylation of the secondary alcohol **3 a** and subsequent deprotection of the nitrogen gave rise to the free amine salt, which was subjected to base treatment to undergo the desired aza‐Michael addition in good yield. The two diastereomeric homopipecolic acids **5 a** were formed as almost 2:1 mixture and their configuration was determined by NMR and X‐ray structure analysis. Obviously, this protocol is well suited for the synthesis of tetra‐substituted piperidine rings.

**Scheme 4:**
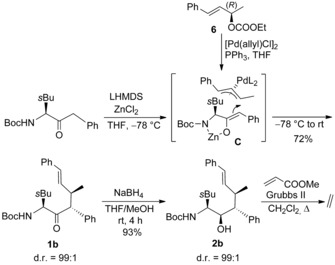
Attempt to synthesize pentasubstituted homopipecolic acids.

To get access to even higher substituted piperidines we decided to use chiral allylic substrates such as **6** to introduce an additional substituent at the 3‐position (Scheme 4). With (*R*)‐**6** a matched situation was observed and the allylation product **1 b** was obtained as a single diastereomer. Also the next step, the stereoselective reduction worked perfectly. Unfortunately, we were unable to convert **2 b** into the corresponding α,β‐unsaturated ester **3 b**, although a wide range of metathesis protocols has been investigated.

**Scheme 5:**
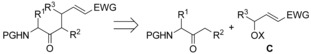
Allylic alkylation of chelated aminoketone with functionalized allyl substrates C.

Therefore, we decided to change our strategy and to introduce the electron‐withdrawing group (EWG) directly via allylic alkylation using functionalized allylic substrates **C** (Scheme 5). This is a more straightforward protocol, but it was unclear which substituents and EWG's are favoring the allylic alkylation and which the Michael addition, because in general, both reactions can proceed already at −78 °C (depending on the substitution pattern).

In addition, these allylic substrates might undergo deprotonation of the π‐allyl Pd intermediate under the basic reaction conditions, providing electron‐poor dienes, which might also cause side reactions. This might be the reason why these types of allylic substrates have been used only very sporadically in palladium‐catalyzed allylic alkylations.^22^

**Scheme 6:**
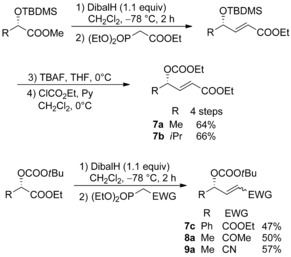
Synthesis of functionalized allylic substrates.

The corresponding methyl‐substituted allylic substrates can easily be obtained from *O*‐protected lactic acid ester via Dibal reduction/ Horner–Wadsworth–Emmons (HWE) olefination, which provides the corresponding α,β‐unsaturated ester **7 a** and ketone **8 a** as single (*E*)‐isomer, while in case of nitrile **9 a** a 3:7 (*E*/*Z*)‐mixture was obtained, which could easily be separated by flash chromatography. If a Boc‐protecting group was used on the lactate, the leaving group for the allylic alkylation could directly be introduced. In case of other carbamates it is recommended to introduce this leaving group after the HWE reaction, because Dibal reduction in this case resulted in the formation of side products. To investigate also the influence of the substitution pattern at the double bond we synthesized the isopropyl‐ and phenyl‐substituted esters **7 b** and **7 c** from the corresponding α‐hydroxy esters (Scheme 6).

With these allylic substrates in hand, we next investigated the allylic alkylation of several amino ketones with the lactic acid‐derived allylic substrate **7 a**. Under standard conditions we used 2.5 equiv LHMDS for the deprotonation (conditions **A**) and 1.3 equiv ZnCl_2_ for the formation of the chelated enolate complex. Alternatively, in some cases also 2.05 equiv LDA were used as base (conditions **B**). In this case, less base was used to avoid epimerization of the allylation product after the reaction with this stronger base. In general, better yields were obtained if the amino ketone was used in excess (1.5 equiv) compared to the allyl carbonate because of complete conversion of the allylic substrate (according to crude NMR and TLC). The results obtained are summarized in Table 1.

**Table 1:** Allylic alkylations using α,β‐unsaturated esters **7**.

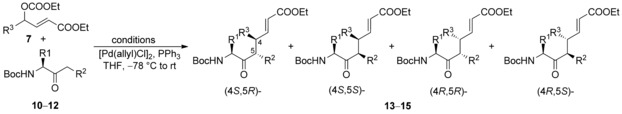													
								Ratio					
Entry	Ketone	R^1^	R^2^	**7**	R^3^	Cond.^[a]^	Prod.	(4*S*,5*R*)	(4*S*,5*S*)	(4*R*,5*R*)	(4*R*,5*S*)	Ratio (*E*/*Z*)	Yield [%]
													
1	**10 a**	Bn	Ph	(*S*)‐**7 a**	Me	A	**13 a**	94	6			>99:1	90
2^[b]^	**10 a**	Bn	Ph	(*S*)‐**7 a**	Me	A	**13 a**	97	3			>99:1	92
3	**10 a**	Bn	Ph	(*R*)‐**7 a**	Me	A	**13 a**	2^[c]^		71	27	98:2	96
4	**11 a**	*i*Pr	Ph	(*S*)‐**7 a**	Me	A	**14 a**	93	7			>99:1	91
5	**11 a**	*i*Pr	Ph	(*S*)‐**7 a**	Me	B	**14 a**	93	7			>99:1	99
6	**11 a**	*i*Pr	Ph	(*R*)‐**7 a**	Me	A	**14 a**	3^[c]^		95	2	80:20	91
7	**11 a**	*i*Pr	Ph	(*R*)‐**7 a**	Me	B	**14 a**	3^[c]^		93	4	87:13	99
8	**12 a**	*s*Bu	Ph	(*S*)‐**7 a**	Me	A	**15 a**	98	2				92
9	**12 a**	*s*Bu	Ph	(*S*)‐**7 a**	Me	B	**15 a**	96	4				88
10	**12 a**	*s*Bu	Ph	(*R*)‐**7 a**	Me	B	**15 a**	2		93	5^[d]^	88:12	99
11	**10 b**	Bn	Me	(*S*)‐**7 a**	Me	A	**13 b**	3^[e]^	97				59
12	**10 b**	Bn	Me	(*S*)‐**7 a**	Me	A^[f]^	**13 b**	5^[e]^	95				80
13	**10 b**	Bn	Me	(*S*)‐**7 a**	Me	B	**13 b**	3^[e]^	97				79
14	**10 b**	Bn	Me	(*R*)‐**7 a**	Me	B	**13 b**			20^[e]^	80	95:5	64
15	**11 b**	*i*Pr	*i*Pr	(*S*)‐**7 a**	Me	B	**14 b**	1^[e]^	99			>99:1	70
16	**10 b**	Bn	Me	(*S*)‐**7 b**	*i*Pr	B	**13 c**	<1^[e]^	>99			>99:1	48
17	**11 a**	*i*Pr	Ph	(*S*)‐**7 b**	*i*Pr	B	**14 c**	>99	<1			>99:1	95
18	**10 a**	Bn	Ph	(*R*)‐**7 c** ^[e,g]^	Ph	A	**13 d**	94^[e,h]^	6				89

[a] Reactions conditions: A: 2.5 equiv LHMDS, 1.3 equiv ZnCl_2_; B: 2.05 equiv LDA, 1.3 equiv ZnCl_2_. [b] Reaction quenched at −20 °C. [c] (*R*)‐**7 a**: 97 % *ee*. [d] The (5*S*/5*R*)‐isomers could not be separated in this case. [e] Change of configuration caused by change of CIP‐priorities of R^2^ or R^3^. [f] 4.0 equiv LHMDS were used. [g] 93 % purity. [h] Ratio determined by NMR.

To determine the diastereomeric ratio, the crude product was analyzed by NMR and also by HPLC, especially in cases where more than two diastereomers were formed (mismatched situations). In general, the diastereomers could easily be separated by flash chromatography, providing enantio‐ and diastereomerically pure products.

With the phenylalanine‐derived benzyl ketone **10 a** the allylation proceeded cleanly. With (*S*)‐**7 a** the (4*S*,5*R*)‐diastereomer of 1**3 a** was obtained with high diastereoselectivity and in high yield (entry 1). Only the formation of the (*E*)‐configured double bond was observed. The stereoselectivity could be improved by quenching the reaction mixture at −20 °C (entry 2). The reaction starts at around −65 °C and is finished at −20 °C. Interestingly, the opposite enantiomer of **7 a**, (*R*)‐**7 a** gave even a better yield, although this seemed to be the mismatched case (entry 3). Here, also traces of the (*Z*)‐isomer could be determined. This effect became more significant in reactions of the valine‐derived benzyl ketone **11 a**. The yields and selectivities were comparable to the results obtained with **10 a**, while no big difference was observed if the different bases are used (entries 4,5). In case of the (*R*)‐**7 a** a higher ratio for the (*Z*)‐isomer was formed. Here LDA not only gave a higher yield, but also a better (*E*/*Z*)‐ratio. A similar situation was found with the isoleucine‐derived benzyl ketone **12 a** (entries 8–10).

The high diastereoselectivity probably results from a clear preference of the conjugated (*Z*)‐enolate **C** over the (*E*)‐isomer. One might expect that the stereoselective outcome of alkyl‐substituted enolates should significantly depend on the steric size of the substituent. Therefore, we also subjected the ethyl and isobutyl ketones **10 b** and **11 b** to our reaction conditions. However, with the methyl‐substituted enolate resulting from **10 b**, no significant difference in diastereoselectivity (3:97) was observed, almost independent on the base used (entries 11–13). With these *n*‐alkyl substituted ketones the yields were significantly lower, compared to the aryl derivatives. Under our standard conditions using 2.5 equiv LHMDS a yield of only 59 % was obtained. It could be improved to 80 % by using 4 equiv of base or by switching to the stronger base LDA. With (*R*)‐**7 a** the yield dropped again, and in this case also the diastereoselectivity (entry 14). Here 5 % of the (*Z*)‐isomer were formed. Increasing the steric bulk of the enolate substituent also increased the diastereoselectivity of the reaction, although here with a small decrease in yield (entry 15).

With these results in hand, we next investigated the influence of the substitution pattern on the allyl substrate. Replacing the methyl substituent of (*S*)‐**7 a** by a sterically more demanding isopropyl group resulted in the exclusive formation of a single diastereomer, independent which ketone was used (entries 16 and 17). But also here, the alkyl ketone **10 b** gave significantly lower yield (entry 16).

Obviously, allylic carbonates bearing an ester functionality as an electron‐withdrawing group are excellent substrates for allylic alkylation. In none of the reactions 1,4‐addition of the enolate towards the double bond was observed. Therefore, we also investigated the lactic acid‐derived ketones **8 a** and nitriles **9 a**. The results observed with ketone **8 a** were comparable to those obtained with the corresponding ester (Table 2), the diastereoselectivities were only slightly worse. But in this case a stronger dependence on the workup temperature was observed. Here, quenching the reaction at −25 °C resulted in a significant increase in the diastereoselectivity (entries 2/3, 4/5). As expected, the yields and selectivities were lower with the alkyl ketone **10 b** (entry 6). Prolonging the reaction time had no significant influence on the yield of **16 b**, only more side products are formed. In this case, 20–25 % of the corresponding Michael adduct could be identified as one of the major side products. In all examples investigated, the (*E*)‐configured allylation product was formed exclusively.

**Table 2:** Allylic alkylations using α,β‐unsaturated ketone **8 a**.

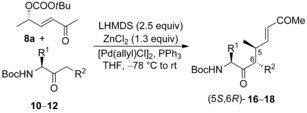							
					Ratio		
Entry	Ketone	R^1^	R^2^	Prod.	(5*S*,6*R*)	(5*S*,6*S*)	Yield [%]
1	**10 a**	Bn	Ph	**16 a**	95	5	96
2	**11 a**	*i*Pr	Ph	**17 a**	90	10	88
3^[a]^	**11 a**	*i*Pr	Ph	**17 a**	98	2	86
4	**12 a**	*s*Bu	Ph	**18 a**	94	6	94
5^[a]^	**12 a**	*s*Bu	Ph	**18 a**	99	1	85
6	**10 b**	Bn	Me	**16 b**	6^[b]^	94	43

[a] Reaction quenched at −25 °C. [b] Change of configuration caused by change of CIP priorities of R^2^.

As mentioned earlier, the HWE‐olefination provided a mixture of (*E*)‐ and (*Z*)‐α,β‐unsaturated nitrile **9 a**. In general, such unselective reactions are undesired, but in this case, it was an advantage, because it gave us the opportunity to investigate the two different allylic substrates separately (Table 3). One might expect the formation of four different products, depending on the allylic substrate used. The configuration at C‐4 results mainly from stereoretention in the allylic fragment, while the configuration at C‐5 is controlled by the adjacent side chain at C‐7. In all previous cases the (*E*)‐configured allylation product was formed almost exclusively.

**Table 3:** Allylic alkylations using α,β‐unsaturated nitriles **9 a**.

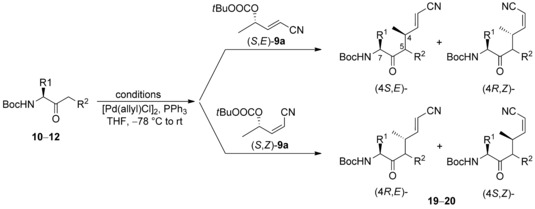												
								Ratio (5*R*/5*S*)				
Entry	Ketone	R^1^	R^2^	**9 a**	Cond.^[a]^	Product	Ratio (*E*/*Z*)	(4*S*,*E*)	(4*R*,*Z*)	(4*R*,*E*)	(4*S*,*Z*)	Yield [%]
1	**10 a**	Bn	Ph	(*S*,*E*)	A	**19 a**	36:64	85:15	95:5			82
2	**10 a**	Bn	Ph	(*S*,*E*)	B	**19 a**	60:40	91:9	96:4			89
3	**10 a**	Bn	Ph	(*R*,*E*)	A	**19 a**	87:13			98:2	86:14	93
4	**10 a**	Bn	Ph	(*S*,*Z*)	A	**19 a**	94:6			98:2	n.d.	95
5	**10 a**	Bn	Ph	(*S*,*Z*)	B	**19 a**	92:8			97:3	n.d.	95
6	**11 a**	*i*Pr	Ph	(*S*,*E*)	A	**20 a**	17:83	88:12	95:5			99
7	**11 a**	*i*Pr	Ph	(*S*,*E*)	B	**20 a**	42:58	91:9	>99:1			88
8	**11 a**	*i*Pr	Ph	(*S*,*Z*)	B	**20 a**	73:27			99:1	98:2	92
9	**10 b**	Bn	Me	(*S*,*E*)	B	**19 b**	73:27	5:95^[b]^	3:97^[b]^			88
10	**10 b**	Bn	Me	(*S*,*Z*)	B	**19 b**	91:9			8:92^[b]^	5:95^[b]^	80

[a] Reactions conditions: A: 2.5 equiv LHMDS, 1.3 equiv ZnCl_2_; B: 2.05 equiv LDA, 1.3 equiv ZnCl_2_. [b] Change of configuration caused by change of CIP priorities of R^2^.

The situation seemed quite different in case of the nitriles **9**. Here, the (*S*)‐configured compounds obviously represent the mis‐matched case (Table 3). Although the allylation products were formed with good diastereoselectivities, a significant isomerization of the double bond was observed. With LHMDS as a base, the (*Z*)‐isomer was formed preferentially from the (*E*)‐allylic substrate (ratio *E*/*Z* 1:2) (entry 1), while with LDA almost a 1:1 mixture was obtained (entry 2). If the (*S*,*E*)‐**9 a** is the mismatched case, the (*R*,*E*)‐isomer should represent the matched one. And indeed, with this (*E*)‐isomer, the (*E*)‐allylation product was formed with good *E*/*Z*‐selectivity and perfect diastereoselectivity (entry 3).

On the other hand, if the π‐allyl–Pd complex intermediate undergoes fast equilibration, the same product as from (*R*,*E*)‐**9 a** should also be obtained from the (*S*,*Z*)‐isomer. Very similar results were obtained using LHDMS as a base, although the diastereoselectivity for the minor (*Z*)‐isomer was lower in this case (entry 4). Also in this example with LDA a higher amount of the (*E*)‐isomer was obtained (entry 5). To prove the generality of this observation, we also subjected some of our other aminoketones to the same reaction conditions. Comparable results were obtained with the valine‐derived ketone **11 a** and ethyl ketone **10 b**, although the *E*/*Z*‐selectivity was worse (entries 6–10).

**Scheme 7:**
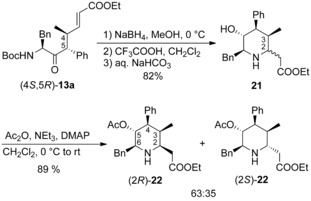
Synthesis of pentasubstituted homopipecolic acid esters **22**.

With these allylation products in hand, we came back to our original idea to synthesize highly substituted homopipecolic acid derivatives via aza‐Michael addition. In principle, all positions of the piperidine ring can be modified by using suitable aminoketone and allylic substrates. Also different functionalities (ester, ketone, nitrile) can be introduced at C‐1 allowing further synthetic transformations. Exemplarily, we subjected ester (4*S*,5*R*)‐**13 a** to the previously described (Scheme 1) reaction conditions. Reduction of the keto functionality delivered also here a single diastereomer (Scheme 7). Cleavage of the Boc‐group and cyclization under basic conditions provided the desired homopipecolic ester **21** in good overall yield as a ∼ 2:1 diastereomeric mixture, as determined by NMR, while the (2*R*)‐isomer was formed preferentially. Unfortunately, the two diastereomers could not be separated at this stage, that's why we tried to acylate the secondary piperidine‐*N* to get separable amides. Surprisingly, no *N*‐acylation was observed neither with Boc_2_O nor Cbz‐Cl, only uncharacterized by‐products were obtained. With Ac_2_O an acetylation was observed, but not as expected on the *N*‐ but on the OH‐functionality. But in this *O*‐acetylated form a separation of the two diastereomers (2*R*)‐ and (2*S*)‐**22** could be accomplished. To prove, if the acetate substituent has an influence on the stereoselectivity, we changed also the order of steps. But if the OH‐functionality was first acetylated and then the amine deprotected/cyclized, the diastereoselectivity was comparable (70:30).

**Scheme 8:**
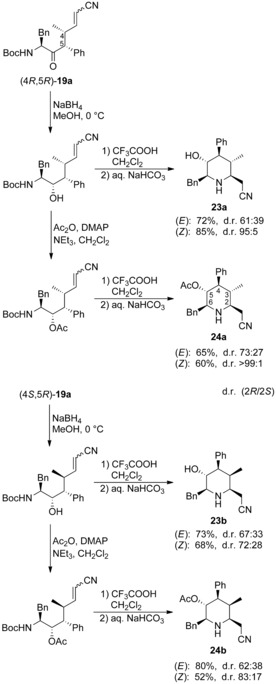
Synthesis of pentasubstituted homopipecolic acid nitriles **23** and **24**.

To investigate the influence of the 4‐methyl group and the double bond geometry on the stereochemical outcome of the reaction, we subjected all four nitrile allylation products **19 a** to deprotection/ cyclization. In case of the (4*R*) isomers, the (*Z*)‐isomer gave definitely a much higher selectivity in the cyclization step (Scheme 8). The acetate was also here found to be superior to the alcohols. In addition, we subjected the alcohols and acetates of the (4*S*)‐series to cyclization. In case of the (*E*)‐isomer, the configuration at C‐4 obviously has no tremendous influence on the selectivity, while the effect was significant in case of the (*Z*)‐isomer. Here, the reaction mixture had to be warmed to 30 °C for one hour for complete conversion.

## Conclusions

In conclusion we could show, that allylic alkylations of amino ketones are versatile tools in organic synthesis, not only for the synthesis of highly functionalized ketones, but also the generation of highly substituted piperidines and homopipecolic acid derivatives. Up to five stereogenic centers can be incorporated, while at least three of them are formed in a highly stereoselective fashion. The substituent at C‐6 originates from an α‐amino acid and controls most of the others. The configuration at C‐4 is the result of a highly stereoselective allylation of a chelated amino ketone enolate and the stereoselective reduction of the ketone functionality (C‐5) is directed by the two adjacent stereogenic centers. The configuration at C‐3 is transferred from the allylic substrate. Depending on the configuration of the allyl carbonate and its double bond geometry, both stereoisomers can be obtained in a highly stereoselective fashion. Several electron‐withdrawing groups can also be part of the allylic substrate, which allows the direct incorporation of esters, ketones or nitriles onto the piperidine ring. Obviously, the allylic alkylation is faster than a competitive Michael addition, because only with the highly reactive α,β‐unsaturated ketones a significant amount of Michael adduct was obtained as side product. α,β‐Unsaturated ketones and esters give (*E*)‐configured allylation products exclusively, while nitriles provide (*E*/*Z*)‐mixtures. Nevertheless, in the cyclization step all stereoisomers deliver the (2*R*)‐configured piperidines preferentially, although the selectivity depends on the substitution pattern and the olefin geometry.

## Experimental Section

**General remarks**: All air‐ or moisture‐sensitive reactions were carried out in dried glassware (>100 °C) under an atmosphere of nitrogen. Dried solvents were distilled before use. The products were purified by flash chromatography on silica gel (0.063–0.2 mm). Mixtures of EtOAc and petroleum ether were generally used as eluents. Analytical TLC was performed on pre‐coated silica gel plates (Macherey–Nagel, Polygram® SIL G/UV254). Visualization was accomplished with UV‐light and KMnO_4_ or Ninhydrin solution. Melting points were determined with a Laboratory Devices MEL‐TEMP II melting point apparatus and are uncorrected. ^1^H and ^13^C NMR spectra were recorded with Bruker AV II 400 [400 MHz (^1^H) and 100 MHz (^13^C)] spectrometer in CDCl_3_, unless otherwise specified. Chemical shifts are reported in ppm relative to TMS, and CHCl_3_ was used as the internal standard. Mass spectra were recorded with a Finnigan MAT 95 spectrometer (quadrupole) using the CI technique.

**Methyl (5*R*,6*R*,7*S***,***E*** **)‐7‐[(*tert*‐butoxycarbonyl)amino]‐6‐hydroxy‐8‐methyl‐5‐phenylnon‐2‐enoate (3 a)**: The solutions of amino alcohol **2 a** 18 (821 mg, 2.00 mmol) in CH_2_Cl_2_ (23 mL) and methyl acrylate (430 mg, 5.00 mmol) in CH_2_Cl_2_ (6 mL) were added simultaneously to a stirring solution of Grubbs II‐catalyst (180 mg, 212 μmol) dissolved in CH_2_Cl_2_ (6 mL). The reaction mixture was refluxed under nitrogen atmosphere for 16 h. The solvent was evaporated in vacuo and the crude product purified by column chromatography (silica, petroleum ether/Et_2_O 80:20, 60:40, 50:50) to yield **3 a** (629 mg, 1.61 mmol, 81 %) as a colorless viscous oil. [*α*]20D =−61.4 (*c*=1.00, CHCl_3_); ^1^H NMR (400 MHz, CDCl_3_): *δ*=7.23–7.36 (m, 5 H), 6.79 (m, 1 H), 5.82 (d, *J=*15.6 Hz, 1 H), 4.17 (d, *J=*10.3 Hz, 1 H), 3.74 (ddd, *J=*6.8, 6.8, 3.9 Hz, 1 H), 3.67 (s, 1 H), 3.45–3.50 (m, 1 H), 2.97 (td, *J=*7.6, 3.8 Hz, 1 H), 2.71 (dd, *J=*7.1, 7.1 Hz, 2 H), 1.89–1.97 (m, 1 H), 1.82 (d, *J=*7.0 Hz, 1 H), 1.42 (s, 9 H), 0.92 (d, *J=*6.8 Hz, 3 H), 0.87 (d, *J=*6.7 Hz, 3 H) ppm; ^13^C NMR (100 MHz, CDCl_3_): *δ*=166.8, 156.3, 147.0, 139.3, 129.3, 128.8, 127.3, 122.6, 79.3, 74.9, 57.5, 51.4, 46.5, 35.9, 28.4, 28.3, 20.4, 17.3 ppm; LC‐MS: Luna, 0.6 mL min^−1^, MeCN/H_2_O 50:50, *t*_R_ (**3 a**)=7.76 min; HPLC (Reprosil 100 Chiral‐NR 8 μm, *n*‐hexane/*i*PrOH 95:5, 1.5 mL min^−1^, 210 nm): *t*_R_ (**3 a**)=12.03 min; HRMS (CI) calcd for: C_22_H_33_NO_5_ [*M*+H]^+^: 392.2431, found: 392.2441.

**Methyl (5*R*,6*R*,7*S***,***E*** **)‐6‐acetoxy‐7‐[(*tert*‐butoxycarbonyl)amino]‐8‐methyl‐5‐phenylnon‐2‐enoate (4 a)**: To a solution of alcohol **3 a** (98.0 mg, 250 μmol) in CH_2_Cl_2_ (1.5 mL) NEt_3_ (43.0 μL, 306 μmol), Ac_2_O (29.0 μL, 301 μmol) and DMAP (3.0 mg, 24.6 μmol) were subsequently added at room temperature. The mixture was stirred for 0.5 h before it was washed with HCl (1 m). The aqueous layer was extracted three times with Et_2_O, the combined organic layers were dried (MgSO_4_), and the solvent was evaporated in vacuo. Flash chromatography (silica, petroleum ether/EtOAc 80:20) gave rise to **4 a** (99.7 mg, 230 μmol, 92 %) as a colorless resin. [*α*]20D =−82.2 (*c*=1.00, CHCl_3_); ^1^H NMR (400 MHz, CDCl_3_): *δ*=7.23–7.33 (m, 5 H), 6.76 (dt, *J=*15.6, 7.2 Hz, 1 H), 5.78 (d, *J=*15.6 Hz, 1 H), 5.13 (dd, *J=*7.8, 3.5 Hz, 1 H), 3.98 (d, *J=*10.6 Hz, 1 H), 3.67 (s, 3 H), 3.59–3.65 (m, 1 H), 3.60–3.11 (m, 1 H), 2.59–2.63 (m, 2 H), 2.05 (s, 3 H), 1.69–1.77 (m, 1 H), 1.41(s, 9 H), 0.89 (d, *J=*6.8 Hz, 3 H), 0.79 (d, *J=*6.7 Hz, 3 H) ppm; ^13^C NMR (100 MHz, CDCl_3_): *δ*=170.3, 166.7, 155.4, 146.3, 139.1, 129.2, 128.4, 127.2, 122.8, 79.1, 75.3, 55.6, 51.4, 45.7, 35.7, 28.5, 28.3, 21.0, 20.1, 16.7 ppm; LC‐MS (Luna, 0.6 mL min^−1^, MeCN/H_2_O 55:45): *t*_R_ (**4 a**)=11.81 min; HPLC (Reprosil 100 Chiral‐NR 8 μm, *n*‐hexane/*i*PrOH 95:5, 1.5 mL min^−1^, 210 nm): *t*_R_ (**4 a**)=25.75 min; HRMS (CI) calcd for: C_24_H_36_NO_6_ [*M*+H]^+^: 434.2537, found: 434.2537.

**Methyl 2‐[(2*S*,4*R*,5*R*,6*S*)‐5‐acetoxy‐6‐isopropyl‐4‐phenylpiperidin‐2‐yl]acetate [(2*S*)‐5 a] and methyl 2‐[(2*R*,4*R*,5*R*,6*S*)‐5‐acetoxy‐6‐isopropyl‐4‐phenylpiperidin‐2‐yl]acetate [(2*R*)‐5 a]**: Acetate **4 a** (94.0 mg, 220 μmol) was dissolved in CH_2_Cl_2_ (1.50 mL) and TFA (210 μL, 2.64 mmol) was added at 0 °C. After stirring for one hour at room temperature, the solvent was removed in vacuo. The residue was dissolved in CH_2_Cl_2_ (2.40 mL), NEt_3_ (180 μL, 1.10 mmol) was added and the reaction mixture was stirred overnight. The reaction mixture was diluted with CH_2_Cl_2_, the organic layer was washed three times with H_2_O and dried (Na_2_SO_4_). The solvent was removed in vacuo and column chromatography (silica, petroleum ether/EtOAc 80:20) provided a mixture of (2*S*)‐**5 a** and (2*R*)‐**5 a** (57.2 mg, 171 μmol, 78 %, d.r. 63:37) as colorless solid. The isomers were separated by further column chromatography (silica, petroleum ether/EtOAc 85:15) to obtain (2*S*)‐**5 a** (36.0 mg, 108 μmol) and (2*R*)‐**5 a** (21.2 mg 63.0 μmol) as colorless solids. (2*S*)‐**5 a**: M.p. 70–72 °C. [*α*]20D =+9.1 (*c*=1.00, CHCl_3_); ^1^H NMR (400 MHz, CDCl_3_): *δ*=7.17–7.28 (m, 5 H), 4.95 (dd, *J=*9.9, 9.9 Hz, 1 H), 3.69 (s, 3 H), 3.11 (m, 1 H), 2.76 (ddd, *J=*14.3, 10.4, 3.9 Hz, 1 H), 2.66 (dd, *J=*9.5, 2.3 Hz, 1 H), 2.46 (d, *J=*6.5 Hz, 2 H), 1.75–1.96 (m, 3 H), 1.73 (s, 3 H), 1.57 (ddd, *J=*12.9, 12.9, 11.1 Hz, 1 H), 1.01 (d, *J=*7.1 Hz, 3 H), 0.92 (d, *J=*6.9 Hz, 3 H) ppm; ^13^C NMR (100 MHz, CDCl_3_): *δ*=172.7, 169. 7, 141.6, 128.2, 127.9, 126.8, 74.4, 65.1, 52.9, 51.6, 49.4, 40.3, 39.5, 27.6, 20.6, 20.2, 15.5 ppm. (2*R*)‐**5 a**: M.p. 96–98 °C; [*α*]20D =−24.9 (*c*=1.00, CHCl_3_); ^1^H NMR (400 MHz, CDCl_3_): *δ*=7.16–7.29 (m, 5 H), 4.94 (dd, *J=*10.0, 10.0 Hz, 1 H), 3.70 (s, 3 H), 3.64–3.70 (m, 1 H), 2.99 (dd, *J=*15.0, 15.0, 10.0 Hz, 1 H), 2.89 (m, 1 H), 2.83 (dd, *J=*9.6, 1.9 Hz, 1 H), 2.47 (dd, *J=*15.0, 5.0 Hz, 1 H), 2.15 (ddd, *J=*13.5, 13.5, 5.4 Hz, 1 H), 1.75–1.82 (m, 2 H), 1.73 (s, 3 H), 0.94 (d, *J=*7.0 Hz, 3 H), 0.90 (d, *J=*6.9 Hz, 1 H) ppm; ^13^C NMR (100 MHz, CDCl_3_): *δ*=172.8, 169.6, 141.4, 128.3, 127.9, 126.9, 75.0, 57.8, 51.6, 48.8, 44.5, 37.0, 35.7, 27.1, 20.6, 20.2, 15.1 ppm; LC‐MS (Luna, 0.6 mL min^−1^, MeCN/H_2_O 50:50): *t*_R_ (2*R*)‐**5 a**=6.13 min, *t*_R_ (2*S*)‐**5 a**=7.97 min; HRMS (CI) calcd for: C_19_H_28_NO_4_ [*M*+H]^+^: 334.2013, found: 334.2026.

**Ethyl (4*S*,5*R*,7*S***,***E*** **)‐7‐[(*tert*‐butoxycarbonyl)amino]‐4‐methyl‐6‐oxo‐5,8‐diphenyloct‐2‐enoate [(4*S*,5*R*)‐13 a]** (Table 1, entry 1): In a Schlenk tube zinc chloride (44.3 mg, 325 μmol) was dried with a heat gun under high vacuum. After cooling to room temperature, ketone **10 a** (85.0 mg, 250 μmol) was added dissolved in THF (1.5 mL). At −78 °C LHMDS (1 m in THF, 630 μL, 630 μmol) was added and the solution was stirred for 0.5 h. In a second Schlenk tube [Pd(allyl)Cl]_2_ (1.2 mg, 334 μmol), PPh_3_ (3.50 mg, 13.3 μmol) and carbonate (*S*)‐**7 a** (36.1 mg, 167 μmol) were dissolved in THF (1.70 mL). The solution was stirred for 5 min before being added dropwise to the enolate solution. The reaction mixture was warmed to room temperature, before it was diluted with Et_2_O and hydrolyzed with aq. KHSO_4_ (1 m). The aqueous layer was extracted three times with Et_2_O, the combined organic layers were dried (MgSO_4_) and evaporated in vacuo. Flash chromatography (silica, petroleum ether/EtOAc 97:3, 95:5, 90:10) of the crude residue gave rise to (4*S*,5*R*)‐**13 a** (69.9 mg, 150 μmol, 90 %, d.r. 94:6) as a colorless solid. M.p. 105–107 °C; [*α*]20D =−122.4 (*c*=1.00, CHCl_3_); ^1^H NMR (400 MHz, CDCl_3_): *δ*=7.27–6.82 (m, 10 H), 6.61 (dd, *J=*15.7, 8.2 Hz, 1 H), 5.59 (dd, *J=*15.8, 0.8 Hz, 1 H), 4.80 (d, *J=*8.5 Hz, 1 H), 4.48 (ddd, *J=*8.0, 8.0, 6.3 Hz, 1 H), 4.08 (qd, *J=*7.1, 1.3 Hz, 2 H), 3.89 (d, *J=*10.3 Hz, 1 H), 3.22–3.12 (m, 1 H), 2.82 (dd, *J=*14.3, 6.0 Hz, 1 H), 2.67 (dd, *J=*14.1, 7.8 Hz, 1 H), 1.41 (s, 9 H), 1.21 (t, *J=*7.0 Hz, 3 H), 1.13 (d, *J=*6.5 Hz, 3 H) ppm; ^13^C NMR (100 MHz, CDCl_3_): *δ*=206.9, 166.3, 155.3, 150.6, 136.2, 135.4, 129.1, 129.0, 128.9, 128.3, 127.8, 126.6, 121.3, 80.1, 62.0, 60.1, 59.5, 39.1, 36.2, 28.2, 18.4, 14.1 ppm; (4*S*,5*S*)‐**13 a** (selected signals): ^1^H NMR (400 MHz, CDCl_3_): *δ*=6.80 (dd, *J=*15.7, 8.1 Hz, 1 H), 5.86 (d, *J=*15.7 Hz, 1 H), 4.48 (m, 1 H), 3.40 (d, *J=*10.0 Hz, 1 H), 0.71 (d, *J=*6.7 Hz, 1 H) ppm; HPLC (Reprosil 100 Chiral‐NR 8 μm, *n*‐hexane/*i*PrOH 90:10, 1.5 mL min^−1^, 210 nm): *t*_R_ (4*S*,5*S*)‐**13 a=**13.81 min, *t*_R_ (4*S*,5*R*)‐**13 a=**15.85 min; HRMS (CI) calcd for: C_28_H_35_NO_5_ [*M*+H]^+^: 466.2588, found: 466.2576.

**Ethyl 2‐[(2*R*/*S*,3*R*,4*R*,5*R*,6*S*)‐6‐benzyl‐5‐hydroxy‐3‐methyl‐4‐phenylpiperidin‐2‐yl]acetate (21)**: To a solution of (4*S*,5*R*)‐**13 a** (62.3 mg, 134 μmol) in THF/MeOH (9:1, 2.2 mL) NaBH_4_ (10.1 mg, 268 μmol) was added at 0 °C. After stirring at 0 °C until complete conversion was observed (TLC), the reaction mixture was diluted with Et_2_O and hydrolyzed by addition of citric acid (aq. 10 w%). The aqueous layer was extracted three times with Et_2_O and the combined organic layers were washed with satd. NaHCO_3_ and dried (MgSO_4_). The solvent was evaporated in vacuo and the crude product was reacted with TFA (895 μL, 11.6 mmol) in DCM (0.45 mL). After complete deprotection, the mixture was diluted with Et_2_O and hydrolyzed with satd. NaHCO_3_. The organic layer was extracted twice with Et_2_O, the combined organic layers were dried (MgSO_4_) and evaporated in vacuo. After column chromatography (silica, petroleum ether/EtOAc 60:40), **21** (39.4 mg, 107 μmol, 82 %) was obtained as a colorless solid. M.p. 95–97 °C. (2*R*)‐**21**: ^1^H NMR (400 MHz, CDCl_3_): *δ*=7.39–7.24 (m, 10 H), 4.01–3.83 (m, 3 H), 3.44 (dd, *J=*13.5, 2.7 Hz, 1 H), 3.29–3.24 (m, 1 H), 2.94 (dd, *J=*11.1, 4.0 Hz, 1 H), 2.87 (ddd, *J=*9.3, 9.3, 2.4 Hz, 1 H), 2.59 (dd, *J=*13.5,=9.8 Hz, 1 H), 2.36–2.25 (m, 2 H), 1.88–1.84 (m, 1 H), 1.69 (bs, 1 H), 1.08 (t, *J=*7.2 Hz, 3 H), 0.71 (d, *J=*7.1 Hz, 3 H) ppm; ^13^C NMR (100 MHz, CDCl_3_): *δ*=172.1, 140.2, 139.0, 129.3, 128.8, 128.7, 128.5, 126.7, 126., 69.0, 64.2, 60.4, 57.2, 55.7, 40.3, 39.0, 38.9, 14.0, 7.6 ppm. (2*S*)‐**21** (selected signals): ^1^H NMR (400 MHz, CDCl_3_): *δ*=3.07–3.01 (m, 1 H), 2.54–2.49 (m, 1 H), 1.82–1.77 (m, 1 H), 1.11 (t, *J=*7.2 Hz, 3 H), 0.93 (d, *J=*7.2 Hz, 3 H) ppm; ^13^C NMR (100 MHz, CDCl_3_) *δ*=172.0, 140.1, 138.8, 129.3, 128.7, 128.7, 128.4, 69.7, 60.3, 57.1, 55.3, 49.5, 40.0, 39.1, 37.0, 15.3, 14.1 ppm; HRMS (CI) calcd for: C_23_H_29_NO_3_ [*M*+H]^+^: 368.220, found: 368.2218.

**Ethyl 2‐[(2*R*,3*R*,4*R*,5*R*,6*S*)‐5‐acetoxy‐6‐benzyl‐3‐methyl‐4‐phenylpiperidin‐2‐yl]acetate [(2*R*)‐22] and ethyl 2‐[(2*S*,3*R*,4*R*,5*R*,6*S*)‐5‐acetoxy‐6‐benzyl‐3‐methyl‐4‐phenylpiperidin‐2‐yl]acetate [(2*S*)‐22]**: Alcohol **21** (37.3 mg, 101 μmol) was reacted with NEt_3_ (15.6 μL, 112 μmol), Ac_2_O (10.5 μL, 112 μmol) and DMAP (1.2 mg, 10.2 μmol) in CH_2_Cl_2_ (1.0 mL) at 0 °C. Due to incomplete conversion after stirring overnight further NEt_3_ (4.2 μL, 30.0 μmol) and Ac_2_O (2.9 μL, 30 μmol) were added to the reaction mixture. After column chromatography (silica, petroleum ether/Et_2_O 75:25, 70:30) **22** (36.8 mg, 90 μmol, 89 %, d.r. (2*R*/2*S*) 65:35) was obtained as a colorless solid. The isomers were separated by further column chromatography (silica, petroleum ether/EtOAc 80:20) and gave (2*R*)‐**22** (23.2 mg, 56.7 μmol) and (2*S*)‐**22** (12.5 mg, 30.6 μmol) as colorless solids. (2*R*)‐**22**: M.p. 86–88 °C. [*α*]20D =+24.1 (*c*=1.00, CHCl_3_); ^1^H NMR (400 MHz, CDCl_3_): *δ*=7.34–7.15 (m, 10 H), 5.42 (dd, *J=*11.4, 9.0 Hz, 1 H), 4.00–3.84 (m, 2 H), 3.27 (ddd, *J=*7.9, 5.6, 2.7 Hz, 1 H), 3.14 (dd, *J=*11.5, 4.3 Hz, 1 H), 3.00 (ddd, *J=*9.5, 9.5, 3.3 Hz, 1 H), 2.92 (dd, *J=*13.7, 3.1 Hz, 1 H), 2.60 (dd, *J=*13.7, 10.0 Hz, 1 H), 2.35–2.26 (m, 2 H), 1.94–1.86 (m, 1 H), 1.77 (s, 3 H), 1.07 (t, *J=*7.1 Hz, 3 H), 0.76 (d, *J=*7.1 Hz, 3 H) ppm; ^13^C NMR (100 MHz, CDCl_3_): *δ*=172.0, 170.9, 140.2, 138.3, 129.0, 128.5, 128.2, 128.1, 126.4, 126.3, 71.1, 62.6, 60.4, 57.0, 53.4, 40.6, 38.9, 38.7, 20.8, 14.0, 7.4 ppm. (2*S*)‐**22**: M.p. 78–80 °C. [*α*]20D =−1.1 (*c*=1.00, CHCl_3_); ^1^H NMR (400 MHz, CDCl_3_): *δ*=7.32–7.15 (m, 10 H), 5.44 (dd, *J=*11.4, 9.3 Hz, 1 H), 4.95–3.82 (m, 2 H), 3.29–3.25 (sh, 2 H), 3.17 (ddd, *J=*9.5, 9.5, 3.1 Hz, 1 H), 2.97–2.90 (m, 2 H), 2.57–2.50 (m, 2 H), 1.88–1.82 (m, 1 H), 1.78 (s, 3 H), 1.11 (t, *J=*7.2 Hz, 3 H), 0.98 (d, *J=*7.1 Hz, 3 H) ppm; ^13^C NMR (100 MHz, CDCl_3_): *δ*=171.7, 170.8, 140.1, 138.2, 129.0, 128.5, 128.3, 128.2, 126.4, 126.3, 71.8, 60.4, 55.6, 55.0, 47.2, 40.3, 39.1, 36.9, 20.8, 15.1, 14.1 ppm. HPLC (Reprosil 100 Chiral‐NR 8 μm, *n*‐hexane/*i*PrOH 95:5, 1.0 mL min^−1^, 210 nm): *t*_R_ (2*R*)‐**22**=18.16 min, *t*_R_ (2*S*)‐**22**=22.35 min; HRMS (CI) calcd for: C_25_H_31_NO_4_ [*M*+H]^+^: 410.2326 found: 410.2321.

The remaining experimental procedures, spectroscopic data, and copies of ^1^H and ^13^C NMR spectra are available in the Supporting Information.

## Conflict of interest

The authors declare no conflict of interest.

## Supporting information

As a service to our authors and readers, this journal provides supporting information supplied by the authors. Such materials are peer reviewed and may be re‐organized for online delivery, but are not copy‐edited or typeset. Technical support issues arising from supporting information (other than missing files) should be addressed to the authors.

Supplementary
