# Supplementary material for: Stereoselective Allylic Alkylations of Amino Ketones and Their Application in the Synthesis of Highly Functionalized Piperidines
Source: Chemistry. 2020 Feb 21;26(14):3181–8. doi: 10.1002/chem.202000051 (PMC7079034; doi:10.1002/chem.202000051)
Supplement: Supplementary file 1 — Supplementary [file CHEM-26-3181-s001.pdf]

CHEMISTRY
A European Journal

Supporting Information

Stereoselective Allylic Alkylations of Amino Ketones and Their Application in the Synthesis of Highly Functionalized Piperidines

Cynthia Prudel, Kai Huwig, and Uli Kazmaier*[a]

chem_202000051_sm_miscellaneous_information.pdf

Table of content

I. General Procedures 2

II. Synthesis of tetrasubstituted homopipecolic acid derivatives 3

III. Synthesis of allyl carbonates 4

IV. Allylic alkylation 6

IV.a Table 1 6

IV.b Table 2 11

IV.c Table 3 13

V. Synthesis of pentasubstituted homopipecolic acids 17

VI. NMR spectra, HPLC and GC chromatogramms 20

I. General Procedures

General procedure 1 (GP 1): Reduction of ketones: To a solution of the amino ketone (1.0 eq) in THF/MeOH (9:1, 10 mL/mmol) $NaBH_4$ (2.0 eq) was added at 0 °C. After stirring at 0 °C until complete conversion was observed (0.5 – 2 h, TLC), the reaction mixture was diluted with Et_2O and hydrolyzed by addition of citric acid (aq. 10 w%). The aqueous layer was extracted three times with Et_2O and the combined organic layers were washed with satd. $NaHCO_3$ and dried ($MgSO_4$). The solvent was evaporated *in vacuo* and the crude product was used without further purification unless specified otherwise.

General procedure 2 (GP 2): Acetylation of alcohols: To a solution of the corresponding alcohol (1.0 eq) in dichloromethane (5 mL/mmol) NEt_3 (1.2 eq), Ac_2O (1.2 eq) and DMAP (0.1 eq) were subsequently added at 0 °C. The solution was warmed to room temperature overnight and then washed with HCl (1 M). The aqueous layer was extracted three times with Et_2O, the combined organic layers were dried ($MgSO_4$), and the solvent was evaporated *in vacuo*. The crude product was used without further purification.

General procedure 3 (GP 3): Boc-deprotection and cyclization: The cyclization precursor (1.0 eq) was dissolved in dichloromethane (1.7 mL/mmol) and TFA (3.3 mL/mmol) was added at 0 °C. The reaction mixture was stirred at room temperature until complete conversion was observed (TLC), diluted with Et_2O and hydrolyzed with satd. $NaHCO_3$. The aqueous layer was extracted three times with Et_2O, the organic layers were dried ($MgSO_4$), the solvent was evaporated *in vacuo.* The crude product was purified by column chromatography.

General procedure 4 (GP 4): Dibal-HWE-one-pot reaction: To a solution of protected α-hydroxyester (1.0 eq) in dichloromethane (10 mL/mmol) Dibal (1.1 eq, 1.0 M in hexane) was dropwise added at −78 °C. After complete addition, the reaction mixture was stirred for two hours at this temperature. Then KO*t*Bu (1.1 eq) and the corresponding phosphonate (1.1 eq) were added at −78 °C. The mixture was warmed to room temperature overnight, citric acid (aq. 10 w%) was added at 0 °C and the mixture was stirred vigorously for 0.5 h. The aqueous layer was extracted three times with Et_2O, the combined organic layers were dried ($MgSO_4$), the solvent was evaporated *in vacuo* and the residue purified by column chromatography.

General procedure 5 (GP 5): Preparation of carbonates: The alcohol (1.0 eq) was dissolved in dichloromethane (0.7 mL/mmol) and cooled to 0 °C. At this temperature pyridine (2.5 eq) and ethyl chloroformate (1.2 eq) were added successively. The reaction was warmed to room temperature overnight. The reaction mixture was hydrolyzed with $KHSO_4$ (1 M) and the aqueous layer was extracted three times with dichloromethane. The combined organic layers were dried ($MgSO_4$), and the solvent was removed *in vacuo*. The residue was purified by column chromatography to obtain the carbonate.

General procedure 6 (GP 6): Pd-catalyzed allylic alkylation: In a Schlenk tube zinc chloride (1.95 eq) was dried with a heat gun under high vacuum. After cooling to room temperature, the aminoketone (1.5 eq) was added dissolved in THF (5 mL/mmol aminoketone). At −78 °C LHMDS (3.75 eq, 1 M in THF) or freshly prepared LDA (3.05 eq) was added and the solution was stirred for 0.5 h. In a second Schlenk tube $[Pd(allyl)Cl]_2$ (2 mol%), PPh_3 (8 mol%) and the

allylic substrate (1.0 eq) were dissolved in THF (5 mL/mmol allylic substrate). The solution was stirred for 5 min before being added dropwise to the enolate solution. The reaction mixture was either warmed to room temperature overnight or to −25 °C within 4 h. Then the solution was diluted with Et_2O and hydrolyzed with aq. $KHSO_4$ (1 M). The aqueous layer was extracted three times with Et_2O, the combined organic layers were dried ($MgSO_4$) and evaporated *in vacuo*. Column chromatography of the crude residue gave rise to the desired product.

II. Synthesis of tetrasubstituted homopipecolic acid derivatives

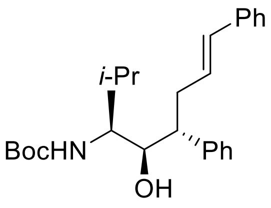

2a

***tert*-Butyl [(3*S*,4*R*,5*R*,*E*)-4-hydroxy-2-methyl-5,8-diphenyl-oct-7-en -3-yl]carbamate (2a)**: According to GP 1 the ketone **1a**[1] (896 mg, 2.20 mmol) was reduced with $NaBH_4$ (166 mg, 4.44 mmol) in MeOH (30 mL) in 60 min. The crude product was purified by column chromatography (silica, petroleum ether/diethyl ether 90:10) to yield **2a** (829 mg, 2.02 mmol, 92 %, *dr* 97:3) as a colorless resin. $[\alpha]_D^{20}$ = −65.8 (c = 1.00, CHCl3). ^{1}H-NMR (400 MHz, CDCl3): δ = 7.15 – 7.37 (m, 10 H), 6.43 (d, *J* = 15.8 Hz, 1 H), 6.06 (m, 1 H), 4.25 (d, *J* = 10.1 Hz, 1 H), 3.85 (m, 1 H), 3.55 (m, 1 H), 2.97 (m, 1 H), 2.64 – 2.77 (m, 2 H), 1.95 – 2.03 (m, 1 H), 1.81 (d, *J* = 6.7 Hz, 1 H), 1.44 (s, 9 H), 0.95 (d, *J* = 6.7 Hz, 3 H), 0.89 (d, *J* = 6.5 Hz, 3 H) ppm. ^{13}C-NMR (100 MHz, CDCl3): δ = 156.3, 140.3, 137.5,131.7, 129.4, 129.3, 128.7, 127.0, 126.0, 79.1, 74.7, 57.5, 47.6, 36.6, 28.3, 20.5, 17.4 ppm. Minor Diastereomer (*selected signals*): ^{1}H-NMR (400 MHz, CDCl3): δ = 6.36 (d, *J* = 15.8 Hz, 1 H), 5.96-6.03 (m, 1 H), 1.47 (s, 9 H) ppm. LC-MS (Luna, 0.6 mL/min, $MeCN/H_2O$ 80/20): t_R(3*S*,4*S*,5*R*,*E*)-**2a** = 12.71 min, t_R(3*S*,4*R*,5*R*,*E*)-**2a** = 13.58 min. HRMS (CI) calcd for: $C_{26}H_{36}NO_3$ [M+H]+: 410.2690, found: 410.2695.

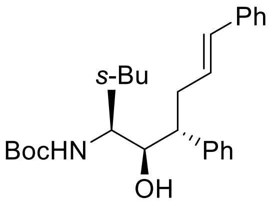

2b

***tert*-Butyl [(3*S*,4*S*,5*R*,6*R*,7*R*,*E*)-5-hydroxy-3,7-dimethyl-6,9-diphe-nylnon-8-en-4-yl]carbamate (2b):** According to GP 1 the ketone **1b**[1] (436 mg, 1.00 mmol) was reduced with $NaBH_4$ (76.0 mg, 2.00 mmol) in THF/MeOH (9:1, 16.5 mL) at room temperature in 4 h. The crude product was purified by column chromatography (silica gel, petroleum ether/diethyl ether 9:1) to yield **2b** (408 mg, 932 µmol, 93 %, *dr* 99:1) as a colorless solid, m.p. 47−49 °C $[\alpha]_D^{20}$ = +20.4 (c = 1.00, $CHCl_3$). ^{1}H-NMR (400 MHz, $CDCl_3$): δ = 7.20 – 7.42 (m, 10 H), 6.59 (d, *J* = 15.8 Hz, 1 H), 6.13 (dd, *J* = 15.8, 9.2 Hz, 1 H), 4.09 (m, 1 H), 3.70 (d, *J* = 10.4 Hz, 1 H), 3.40 (ddd, *J* = 10.4, 6.8, 6.8 Hz, 1 H), 2.91 – 3.02 (m, 1 H), 2.58 (dd, *J* = 10.6, 1.5 Hz, 1 H), 2.09 (d, *J* = 6.3 Hz, 1 H, O), 1.56 – 1.64 (m, 1 H), 1.40 – 1.48 (m, 1 H), 1.38 (s, 9 H), 0.90 – 1.00 (m, 1 H), 0.87 (d, *J* = 6.8 Hz, 3 H), 0.78-0.84 (m, 6 H) ppm. ^{13}C-NMR (100 MHz, $CDCl_3$): δ = 155.6, 139.9, 137.6, 135.1, 130.4, 130.1, 128.6, 127.1, 126.8, 126.1, 79.1, 72.1, 58.3, 52.5, 39.7, 35.6, 28.3, 24.3, 19.8, 16.1, 11.2 ppm. LC-MS (Luna, 0.6 mL/min, $MeCN/H_2O$ 70/30): t_R(**2b**) = 13.27 min. HRMS (CI) calcd for: $C_{28}H_{40}NO_3$ $[M+H]^+$: 438.3003, found: 438.3007.

[1] K. Huwig, K. Schultz, U. Kazmaier, *Angew.Chem.Int.Ed.,* **2015**,*54*,9120–9123; *Angew. Chem.*, **2015**, *127*, 9248 –9251

III. Synthesis of allyl carbonates

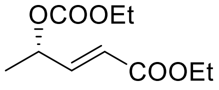

7a

Ethyl (*S*,*E*)-4-[(ethoxycarbonyl)oxy]pent-2-enoate (7a): According to GP 4 TBS-protected methyl L-(-)-lactate[2] (1.32 g, 6.03 mmol) in dichloromethane (60.5 mL) was reduced by Dibal (6.63 mL, 6.63 mmol) and reacted with KO*t*Bu (744 mg, 6.63 mmol) and ethyl 2-(diethoxy phosphoryl)acetate (1.62 g, 7.23 mmol). Column chromatography (silica, petroleum ether/EtOAc 90:10, 85:15, 80:20) provided the TBS-protected unsaturated ester (1.23 g, 4.76 mmol, 79 %) as a colorless oil. For deprotection, this ester (1.23 g, 4.76 mmol) was dissolved in THF (16 mL), and TBAF (1 M, 5.0 mL, 5.00 mmol) was added at 0 °C. After complete conversion (TLC), HCl (1 M) was added, and the aqueous layer was extracted three times with Et_2O. The combined organic layers were dried ($MgSO_4$) and the solvent was removed *in vacuo*. According to GP 5 the crude product was reacted with ethyl chloroformate (686 µL, 7.14 mmol) and pyridine (960 µL, 11.9 mmol) in dichloromethane (3.1 mL). After column chromatography (silica, petroleum ether/EtOAc 90:10) carbonate **7a** (83.0 mg, 3.86 mmol, 81 %) was obtained as a colorless oil. (*S*)-**7a**: $[\alpha]_D^{20}$ = −22.0 (c = 1.00, $CHCl_3$). The corresponding (*R*)-carbonate was prepared likewise. (*R*)-**7a**: $[\alpha]_D^{20}$ = +23.2 (c = 1.00, $CHCl_3$). ^{1}H-NMR (400 MHz, $CDCl_3$): δ = 6.88 (dd, *J* = 15.7, 5.1 Hz, 1 H), 6.01 (dd, *J* = 15.7, 1.5 H, 1 H), 5.34 (m, 2 H), 4.25 – 4.19 (m, 4 H), 1.42 (d, *J* = 6.8 Hz, 3 H), 1.34 – 1.28 (m, 6 H) ppm. ^{13}C-NMR (100 MHz, $CDCl_3$): δ = 165.9, 154.2, 145.5, 121.4, 72.6, 64.2, 60.6, 19.7, 14.2, 14.1 ppm. GC (CP-Chirasil-Dex *CB*, 60 °C (5 min) – 200°C (5 min), rate 4 °C/min, flow 1.2 mL/min): t_R(*S*)-**7a** = 27.43 min, 99%ee; t_R(*R*)-**7a** = 27.63 min, 97%ee). GC-MS (CI, 70eV) m/z [u] (%) = 99.1 (65, $[M{-}OCO_2Et{-}Et]^+$), 127.3 (100, $[M{-}OCO_2Et]^+$), 143.1 (10, $[M{-}CO_2Et]^+$).

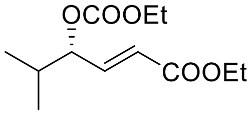

7b

Ethyl (*S*,*E*)-4-[(ethoxycarbonyl)oxy]-5-methylhex-2-enoate (7b): According to GP 4 methyl (*S*)-2-[(*tert*-butyldimethylsilyl)oxy]-3-methylbutanoate[3] (1.28 g, 5.19 mmol) in dichloromethane (52 mL) was reacted with Dibal (5.71 mL, 5.71 mmol), KO*t*Bu (641 mg, 5.71 mmol) and ethyl 2-(diethoxy phosphoryl)acetate (1.28 mg, 5.71 mmol). Column chromatography (silica, petroleum ether/EtOAc 90:10, 80:20) provided the unsaturated ester (1.11 g, 3.87 mmol, 75 %) as a colorless oil. For deprotection, the TBS-protected ester (1.09 g, 3.80 mmol) was dissolved in THF (13 mL) and TBAF (1 M, 4.19 mL, 4.19 mmol) was added at 0 °C. After complete conversion (TLC) HCl (1 M) was added and the aqueous layer was extracted three times with Et_2O. The combined organic layers were dried ($MgSO_4$) and the solvent was removed *in vacuo*. According to GP 5 the crude product was reacted with ethyl chloroformate (438 µL, 4.56 mmol) and pyridine (768 µL, 9.50 mmol) in dichloromethane (3.80 mL). After column chromatography (silica, petroleum ether/EtOAc 90:10) carbonate (*S*)-**7b** (816 mg, 3.34 mmol, 88 %) was obtained as a colorless oil. $[\alpha]_D^{20}$ = −8.9 (c = 1.00, $CHCl_3$). ^{1}H-NMR (400 MHz, $CDCl_3$): δ = 6.86 (dd, *J* = 15.8, 5.8 Hz, 1 H), 6.00 (dd, *J* = 15.8, 1.3 Hz, 1 H), 5.06 (ddd, *J* = 5.8, 5.8, 1.2 Hz, 1 H), 4.23 – 4.18 (m, 4 H), 2.04 – 1.96 (m, 1 H), 1.34 – 1.28 (m, 6 H), 0.98 (d, *J* = 6.7 Hz, 3 H),

---

[2] M. de Léséleuc, É. Godin, S. Parisien-Collette, A. Lévesque, S.K. Collins, *J. Org. Chem.*, **2016**, *81*, 6750–6756.
[3] R. J. Alfie, N. Truong, J. M. Yost, D. M. Coltarta, *Tetrahedron Lett.,* **2017**, *58*, 185–189.

0.96 (d, *J* = 6.7 Hz, 3 H) ppm. ^{13}C-NMR (100 MHz, $CDCl_3$): δ = 165.8, 154.6, 143.4, 122.9, 80.8, 64.2, 60.6, 32.0, 17.9, 17.7, 14.2, 14.2 ppm. GC (CP-Chirasil-Dex CB, 80 °C (2 min) – 200 °C (5 min), rate 10 °C/min, flow 1.2 mL/min): t_R(*S*)-**7b** = 12.33 min, 100 %ee. GC-MS (CI, 70eV) m/z [u] (%) = 127.2 (20, $[M{-}OCO_2Et{-}Et{+}H]^+$), 155.1 (100, $[M{-}OCO_2Et]^+$), 273.1 (8, $[M{+}H]^+$).

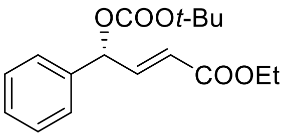

7c

Ethyl (*R*,*E*)-4-[(*tert*-butoxycarbonyl)oxy]-4-phenylbut-2-enoate (7c)[4]: According to GP 4 Boc-protected methyl (*S*)-(+)-mandelate (668 mg, 2.51 mmol) in dichloromethane (25 mL) was reduced by Dibal (2.76 mL, 2.76 mmol) and reacted with KO*t*Bu (338 mg, 3.01 mmol) and ethyl 2-(diethoxy phosphoryl)acetate (675 mg, 3.01 mmol). Column chromatography (silica, petroleum ether/ EtOAc 90:10, 80:20) provided unsaturated ester **7c** (360 mg, 1.17 mmol, 47 %) as a colorless oil (contaminated with 7 % starting material). $[\alpha]_D^{20}$ = +70.1 (c = 1.00, $CHCl_3$). ^{1}H-NMR (400 MHz, $CDCl_3$): δ = 7.41 – 7.32 (m, 5 H), 7.03 (dd, *J* = 15.7, 5.1 Hz, 1 H), 6.17 (dd, *J* = 5.1, 1.6 Hz, 1 H), 6.07 (dd, *J* = 15.7, 1.6 Hz, 1 H), 4.20 (q, *J* = 7.1 Hz, 2 H), 1.48 (s, 9 H), 1.29 (t, *J* = 7.1 Hz, 3 H) ppm. ^{13}C-NMR (100 MHz, $CDCl_3$): δ = 165.9, 152.4, 144.4, 137.1, 128.8, 128.8, 127.3, 121.8, 82.9, 76.5, 60.6, 27.7, 14.2 ppm. GC (CP-Chirasil-Dex CB, 60 °C (5 min) – 200°C (5 min), rate 4 °C/min, flow 1.2 mL/min): t_R(*S*)-**7c** = 42.39 min.

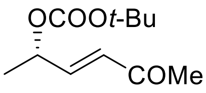

8a

(*S*,*E*)-*tert*-Butyl (5-oxohex-3-en-2-yl) carbonate (8a): According to GP 4 Boc-protected ethyl L-(−)-lactate (1.09 g, 4.99 mmol) in dichloromethane (50 mL) was reacted with Dibal (5.50 mL, 5.50 mmol), KO*t*Bu (673 mg, 6.00 mmol) and diethyl (2-oxopropyl)phosphonate (1.17 mg, 6.00 mmol). Column chromatography (silica, petroleum ether/EtOAc 90:10, 80:20) provided unsaturated carbonate **(*S*)-8a** (536 mg, 2.50 mmol, 50 %) as a colorless oil. $[\alpha]_D^{20}$ = −17.8 (c = 1.00, $CHCl_3$). ^{1}H-NMR (400 MHz, $CDCl_3$): δ = 6.72 (dd, *J* = 16.1, 5.1 Hz, 1 H), 6.23 (dd, *J* = 16.1, 1.5 Hz, 1 H), 5.31 (qdd, *J* = 6.7, 5.2, 1.5 Hz, 1 H), 2.28 (s, 3 H), 1.50 (s, 9 H), 1.42 (d, *J* = 6.7 Hz, 3 H) ppm. ^{13}C-NMR (100 MHz, $CDCl_3$): δ = 198.1, 152.5, 144.7, 129.7, 82.7, 71.8, 27.7, 27.4, 19.8 ppm. GC (CP-Chirasil-Dex CB, 80 °C (2 min) – 200 °C (5 min), rate 10 °C/min, flow 1.2 mL/min): t_R(*S*)-**8a** = 10.94 min, 99.7%ee). GC-MS (CI, 70eV) m/z [u] (%) = 97.1 (100, $[M{-}OCO_2tBu]^+$), 113.1 (41, $[M{-}CO_2tBu]^+$) , 141.1 (3, $[M{-}OtBu]^+$).

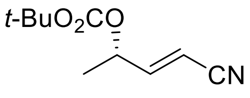

(*S*,*Z*)-9a

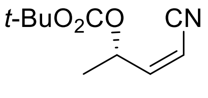

(*S*,*Z*)-9a

***tert*-Butyl (4-cyanobut-3-en-2-yl) carbonates** (*S*,*Z*)-**9a** and (*S*,*E*)-**9a:** According to GP 4 Boc-protected ethyl L-(−)-lactate (738 mg, 4.17 mmol) in dichloromethane (42 mL) was reduced by Dibal (4.58 mL, 4.58 mmol), reacted with KO*t*Bu (561 mg, 5.00 mmol) and diethyl (cyanomethyl)phosphonate (1.09 mg, 5.00 mmol). Column chromatography (silica, petroleum ether/Et_2O 80:20) provided

[4] **literature**: J. H. Lee, S. Lee, *Chem. Sci.*, 2013, **4**, 2922; $[\alpha]_D^{20}$ = +71.6 (c 1.00, $CHCl_3$).

a mixture of unsaturated nitriles **9a** (464 mg, 2.36 mmol, 57 %, *E*/*Z* 30:70) as a colorless oil. The isomers could be separated by further column chromatography (silica, petroleum ether/Et_2O 90:10, 85:15, 80:20) and gave (*S*,*E*)-**9a** (126 mg, 639 µmol, 15 %), (*S*,*Z*)-**9a** (315 mg, 1.60 µmol, 38 %) and a mixed fraction. (*S*,*E*)-**9a:** $[\alpha]_D^{20}$ = +17.6 (c = 1.00, $CHCl_3$). ^{1}H-NMR (400 MHz, $CDCl_3$): δ = 6.67 (dd, *J* = 16.4, 4.9 Hz, 1 H), 5.59 (dd, *J* = 16.4, 1.6 Hz, 1 H), 5.28 – 5.22 (m, 1 H), 1.50 (s, 9 H), 1.40 (d, *J* = 6.7 Hz, 3 H) ppm. ^{13}C-NMR (100 MHz, $CDCl_3$): δ = 152.4, 152.2, 116.6, 100.3, 83.1, 71.3, 27.7, 19.4 ppm. GC (*CP-Chirasil-Dex CB*, 80 °C (2 min) – 200 °C (5 min), rate 10 °C/min, flow 1.2 mL/min): t_R(*S*,*E*)-**9a** = 10.48 min, t_R(*R*,*E*)-**9a** = 10.90 min). GC-MS (CI, 70eV) m/z [u] (%) = 80.1 (50, $[M{-}OCO_2tBu]^+$), 96.1 (65, $[M{-}CO_2tBu]^+$), 198.1 (5, $[M+H]^+$). (*S*,*Z*)-**9a:** $[\alpha]_D^{20}$ = +8.8 (c = 1.00, $CHCl_3$). ^{1}H-NMR (400 MHz, $CDCl_3$): δ = 6.45 (dd, *J* = 11.4, 7.8 Hz, 1 H), 5.51 (dqd, *J* = 7.8, 6.7, 1.1 Hz, 1 H), 5.44 (dd, *J* = 11.4, 1.2 Hz, 1 H), 1.50 (s, 9 H), 1.46 (d, *J* = 6.7 Hz, 3 H) ppm. ^{13}C-NMR (100 MHz, $CDCl_3$): δ = 152.5, 152.2, 114.6, 100.1, 83.0, 71.6, 27.7, 19.7 ppm. GC (CP-Chirasil-Dex CB, 80 °C (2 min) – 200 °C (5 min), rate 10 °C/min, flow 1.2 mL/min): t_R(*R*,*Z*)-**9a** = 10.51 min, t_R(*S*,*Z*)-**9a** = 10.58 min, 99.9%ee. GC-MS (CI, 70eV) m/z [u] (%) = 80.1 (25, $[M{-}OCO_2tBu]^+$), 96.1 (28, $[M{-}CO_2tBu]^+$), 170.1 (4, $[M{-}HCN]^+$), 198.1 (1, $[M+H]^+$).

IV. Allylic alkylation

IV.a Table 1

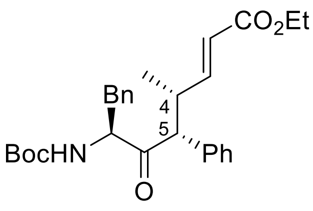

(4*R*,5*R*)-13a

(4*R*,5*R*)-13a, [*Tab.1*, *entry 3*]: According to GP 6 ketone **10a**[1] (102 mg, 300 µmol) was reacted with $ZnCl_2$ (53.2 mg, 391 µmol), LHMDS (1 M, 750 µL, 750 mmol), [Pd(allyl)Cl_2] (1.5 mg, 4.01 µmol), PPh_3 (4.20 mg, 16.0 µmol) and carbonate (*R*)-**7a** (43.3 mg, 200 µmol) in THF (2.00 mL). Aqueous work up and flash chromatography (silica, petroleum ether/EtOAc 97:3, 95:5, 90:10) gave rise to (4*R*,5*R*)-**13a** (89.4 mg, 192 µmol, 96 %, *E*/*Z* 98:2, *dr* 71:27:2) as a colorless solid, m.p. 81–83°C. ^{1}H-NMR (400 MHz, $CDCl_3$): δ = 7.28 – 7.10 (m, 9 H), 6.90 (dd, *J* = 15.7, 8.1 Hz, 1 H), 6.83 – 6.81 (m, 1 H), 5.87 (d, *J* = 15.8 Hz, 1 H), 4.79 (d, *J* = 8.8 Hz, 1 H), 4.54 – 4.44 (m, 1 H), 4.17 (q, *J* = 7.1 Hz, 2 H), 3.90 (d, *J* = 10.4 Hz, 1 H), 3.17 – 3.08 (m, 1 H), 2.79 (dd, *J* = 14.1, 6.4 Hz, 1 H), 2.64 (dd, *J* = 14.1, 7.7 Hz, 1 H), 1.40 (s, 9 H), 1.28(t, *J* = 7.1 Hz, 3 H), 0.81 (d, *J* = 6.8 Hz, 3 H) ppm. ^{13}C-NMR (100 MHz, $CDCl_3$): δ = 206.3, 166.5, 155.2, 151.2, 136.2, 135.0, 129.1, 129.0, 129.0, 128.2, 127.7, 126.4, 121.4, 80.0, 61.7, 60.1, 58.6, 38.3, 36.1, 28.1, 17.2, 14.2 ppm. (4*R*,5*S*)-**13a** (selected signals): ^{1}H-NMR (400 MHz, $CDCl_3$): δ = 6.54 (dd, *J* = 15.7, 8.3 Hz, 1 H), 5.55 (d, *J* = 15.7 Hz, 1 H), 5.00 (d, *J* = 7.9 Hz, 1 H), 3.53 (d, *J* = 9.5 Hz, 1 H), 1.38 (s, 9 H), 1.01 (d, *J* = 6.4 Hz, 3 H) ppm. (4*S*,5*R*,*Z*)-**13a** *(selected signals):* ^{1}H-NMR (400 MHz, $CDCl_3$): δ = 5.83 (dd, *J* = 10.6 Hz, 1 H), 5.51 (d, *J* = 10.8 Hz, 1 H) ppm. HPLC (Reprosil 100 Chiral-NR 8 µm, *n*-hexane/*i*PrOH 90:10, 1.5 mL/min, 210 nm): t_R(4*S*,5*R*,*Z*)-**13a** = 7.36 min, t_R**(4*R*,5*R*)-13a** = 9.68 min, t_R(4*S*,5*R*)-**13a** = 15.04 min, t_R(4*R*,5*S*)-**13a** = 20.25 min. HRMS (CI) calcd for: $C_{28}H_{35}NO_5$ $[M+H]^+$: 466.2588, found: 466.2592.

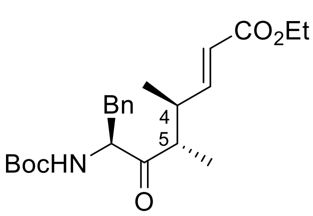

(4*S*,5*S*)-13b

(4*S*,5*S*)-13b, [*Tab.1*, *entry 12*]**:** According to GP 6 ketone **10b**[1] (83.0 mg, 300 µmol) was reacted with $ZnCl_2$ (53.3 mg, 391 µmol), LHMDS (1 M, 1.20 mL, 1.20 mmol), [Pd(allyl)Cl_2] (1.5 mg, 4.01 µmol), PPh_3 (4.20 mg, 16.0 µmol) and carbonate (*S*)-**7a** (43.2 mg, 200 µmol) in THF (2.00 mL). Aqueous work up and flash chromatography (silica, petroleum ether/EtOAc 97:3, 95:5, 90:10) gave rise to (4*S*,5*S*)-**13b** (64.2 mg, 159 µmol, 80 %, *dr* 5:95) as a colorless solid, m.p. 94–96 °C. ^{1}H-NMR (400 MHz, $CDCl_3$): δ = 7.32−7.14 (m, 5 H), 6.71 (dd, *J* = 15.6, 8.5 Hz, 1 H), 5.76 (d, *J* = 15.6, 1 H), 5.06 (d, *J* = 8.8 Hz, 1 H), 4.71 – 4.64 (m, 1 H), 4.17 (q, *J* = 7.0 Hz, 2 H), 3.04 (dd, *J* = 13.8, 7.3 Hz, 1 H), 2.90 (dd, *J* = 13.8, 8.5 Hz, 1 H), 2.72 – 2.63 (m, 1 H), 2.53 (dq, *J* = 6.9, 6.9 Hz, 1 H), 1.41 (s, 9 H), 1.27 (t, *J* = 7.2 Hz, 3 H), 1.02 (d, *J* = 6.5 Hz, 3 H), 0.85 (d, *J* = 7.0 Hz, 3 H) ppm. ^{13}C-NMR (100 MHz, $CDCl_3$): δ = 211.3, 166.3, 155.1, 149.8, 136.2, 129.2, 128.6, 126.9, 122.0, 79.9, 60.2, 59.6, 48.9, 38.6, 37.7, 28.2, 18.1, 14.2, 12.9 ppm. (4*S*,5*R*)-**13b** (*selected signals*): ^{1}H-NMR (400 MHz, $CDCl_3$): δ = 6.79 (dd, *J* = 15.8, 7.5 Hz, 1 H), 2.80 (dd, *J* = 13.8, 7.3 Hz, 1 H), 1.39 (s, 9 H) ppm. ^{13}C-NMR (100 MHz, $CDCl_3$): δ = 151.5, 121.3, 58.4, 48.1, 37.5, 13.1 ppm. HPLC (Reprosil 100 Chiral-NR 8 µm, *n*-hexane/*i*PrOH 95:5, 1.5 mL/min, 210 nm): t_R(4*S*,5*S*)-**13b** = 21.31 min, t_R**(**4*S*,5*R*)-**13b** =25.69 min. HPLC (OD-H Chiralcel RP-18 5 µm, *n*-hexane/*i*PrOH 95:5, 1.0 mL/min, 210 nm): t_R(4*S*,5*R*)-**13b** =12.51 min, t_R(4*S*,5*S*)-**13b** = 13.91 min. HRMS (CI) calcd for: $C_{23}H_{33}NO_5$ $[M+H]^+$: 404.2431, found: 404.2441.

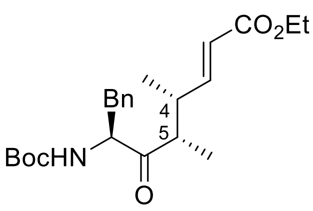

(4*R*,5*S*)-13b

(4*R*,5*S*)-13b, [*Tab.1*, *entry 14*]:**:** According to GP 6 ketone **10b**[1] (104 mg, 375 µmol) was reacted with $ZnCl_2$ (66.4 mg, 488 µmol), LDA (1.36 mL, 767 mmol), [Pd(allyl)Cl_2] (1.8 mg, 4.99 µmol), PPh_3 (5.20 mg, 19.8 µmol) and carbonate (*R*)-**7a** (54.1 mg, 250 µmol) in THF (2.40 mL). Aqueous work up and flash chromatography (silica, petroleum ether/EtOAc 97:3, 95:5, 90:10) gave rise to (4*R*,5*S*)-**13b** (64.0 mg, 159 µmol, 64 %, *E*/*Z* 95:5, *dr*(*E*) 20:80) as a colorless resin. ^{1}H-NMR (400 MHz, $CDCl_3$): δ = 7.33 – 7.22 (m, 3 H), 7.16 (m, 2 H), 6.81 (dd, *J* = 15.7, 7.0 Hz, 1 H), 5.75 (d, *J* = 15.7 Hz, 1 H), 5.06 (d, *J* = 8.7 Hz, 1 H), 4.70 (m, 1 H), 4.18 (q, *J* = 7.1 Hz, 2 H), 3.01 (dd, *J* = 13.9, 7.3 Hz, 1 H), 2.93 (dd, *J* = 13.9, 6.3 Hz, 1 H), 2.83 – 2.74 (m, 1 H), 2.65 – 2.57 (m, 1 H), 1.41 (s, 9 H), 1.29 (t, *J* = 7.1 Hz, 3 H), 0.93 (d, *J* = 6.6 Hz, 3 H), 0.86 (d, *J* = 6.9 Hz, 3 H) ppm. ^{13}C-NMR (100 MHz, $CDCl_3$): δ = 210.9, 166.4, 155.1, 151.0, 136.1, 129.2, 128.6, 127.0, 121.2, 79.9, 60.3, 58.8, 47.9, 38.1, 37.2, 28.2, 14.2, 14.0, 10.7 ppm. (4*R*,5*R*)-**13b** (*selected signals*): ^{1}H-NMR (400 MHz, $CDCl_3$): δ = 6.71 (dd, *J* = 15.7, 8.4 Hz, 1 H) ppm. ^{13}C-NMR (100 MHz, $CDCl_3$): δ = 150.1, 127.0, 122.0, 59.7, 38.6 ppm. (4*S*,5*S*,*Z*)-**13b** (*selected signals*): ^{1}H-NMR (400 MHz, $CDCl_3$): δ = 5.97 (dd, *J* = 11.9, 10.4 Hz, 1 H) ppm. HPLC (Reprosil 100 Chiral-NR 8 µm, *n*-hexane/*i*PrOH 95:5, 1.5 mL/min, 210 nm) t_R(4*R*,5*S*)-**13b** = 15.71 min, , t_R(4*R*,5*R*)-**13b** = 19.69 min, t_R(4*S*,5*S*,*Z*)-**13b** = 23.35 min. HRMS (CI) calcd for: $C_{23}H_{33}NO_5$ $[M+H]^+$: 404.2431, found: 404.2398.

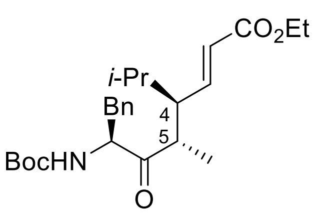

(4*S*,5*S*)-13c

(4*S*,5*S*)-13c, [*Tab.1*, *entry 16*]**:** According to GP 6 ketone **10b**[1] (83.3 mg, 300 µmol) was reacted with $ZnCl_2$ (53.2 mg, 391 µmol), LDA (1.1 mL, 615 µmol), [Pd(allyl)Cl_2] (1.5 mg, 4.01 µmol), PPh_3 (4.20 mg, 16.0 µmol) and carbonate (*S*)-**7b** (48.9 mg, 200 µmol) in THF (2.00 mL). Aqueous work up and flash chromatography (silica, petroleum ether/EtOAc 98:2, 96:4, 93:7) gave rise to (4*S*,5*S*)-**13c** (41.0 mg, 95.0 µmol, 48 %, *dr* >99:1) as a colorless solid, m.p. 77–79 °C. $[\alpha]_D^{20}$ = +12.9 (c = 1.00, $CHCl_3$). ^{1}H-NMR (400 MHz, $CDCl_3$): δ = 7.31 – 7.17 (m, 5 H), 6.59 (dd, *J* = 15.6, 10.8 Hz, 1 H), 5.69 (d, *J* =15.6 Hz, 1 H), 5.10 (d, *J* = 8.8 Hz, 1 H), 4.73 (ddd, *J* = 7.8, 7.8, 7.8 Hz, 1 H), 4.16 (q, *J* = 7.1 Hz, 2 H), 2.96 – 2.94 (m, 2 H), 2.64 (dq, *J* = 6.6, 6.6 Hz, 1 H), 2.30 (ddd, *J* = 10.6, 6.4, 6.4 Hz, 1 H), 1.63 – 1.58 (m, 1 H), 1.44 (s, 9 H), 1.27 (t, *J* = 7.1 Hz, 3 H), 0.83 (d, *J* = 6.7 Hz, 3 H), 0.83 – 0.78 (m, 6 H) ppm. ^{13}C-NMR (100 MHz, $CDCl_3$): δ = 211.2, 165.9, 155.0, 146.7, 136.4, 129.2, 128.6, 127.0, 124.2, 78.0, 60.2, 58.7, 50.3, 46.0, 38.8, 29.0, 28.3, 20.9, 19.5, 14.2, 11.4 ppm. LC-MS (Luna, 1.0 mL/min, 210 nm, MeCN/H_2O 55:45 – 65:35, 30 min): t_R(4*S*,5*S*)-**13c** = 14.76 min. HRMS (CI) calcd for: $C_{25}H_{37}NO_5$ $[M+H]^+$: 432.2755, found: 432.2744.

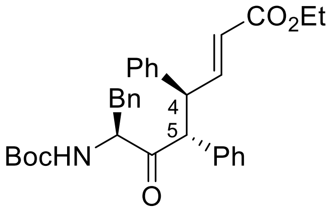

(4*S*,5*R*)-13d

(4*S*,5*R*)-13d, [*Tab.1*, *entry 18*]**:** According to GP 6 ketone **10a**[1] (76.0 mg, 225 µmol) was reacted with $ZnCl_2$ (39.9 mg, 293 µmol), LHMDS (0.56 mL, 560 µmol), [Pd(allyl)Cl_2] (1.1 mg, 3.00 µmol), PPh_3 (3.15 mg, 12.0 µmol) and carbonate (*R*)-**7c** (46.0 mg, 150 µmol) in THF (1.40 mL). Aqueous work up and flash chromatography (silica, petroleum ether/EtOAc 98:2, 96:4, 93:7,90:10) gave rise to (4*S*,5*R*)-**13d** (84.7 mg, 134 µmol, 89 %, *dr* 94:6) as a colorless solid, m.p. 144−146 °C. $[\alpha]_D^{20}$ = –166.5 (c = 1.00, $CHCl_3$). ^{1}H-NMR (400 MHz, $CDCl_3$): δ = 7.34 – 7.06 (m, 13 H), 6.83 – 6.75 (m, 3 H), 5.46 (d, *J* = 15.7, 1 H), 4.52 (d, *J* = 11.4 Hz, 1 H), 4.50 – 4.44 (m, 1 H), 4.26 – 4.21 (m, 2 H), 4.03 (m, 2 H), 2.72 (dd, *J* = 14.1, 6.9 Hz, 1 H), 2.52 (dd, *J* = 14.1, 7.2 Hz, 1 H), 1.42 (s, 9 H), 1.16 (t, *J* = 7.1 Hz, 3 H) ppm. ^{13}C-NMR (100 MHz, $CDCl_3$): δ = 204.6, 166.1, 155.0, 148.3, 140.4, 136.2, 134.3, 129.3, 129.2, 129.0, 128.8, 128.2, 128.1, 128.0, 127.2, 126.3, 122.7, 80.0, 60.8, 60.2, 57.8, 50.6, 35.9, 28.3, 14.1 ppm. (4*S*,5*R*)-**13d** (selected signals): ^{1}H-NMR (400 MHz, $CDCl_3$): δ = 5.85 (d, *J* = 15.6 Hz, 1 H), 4.16 (q, *J* = 7.1 Hz, 2 H), 2.33 – 2.26 (m, 2 H), 1.45 (s, 9 H) ppm. LC-MS (Luna, 1.0 mL/min, 210 nm, MeCN/H_2O 50:50 – 60:40, 35 min): t_R(4*S*,5*R*)-**13d** = 30.34 min. HRMS (CI) calcd for: $C_{33}H_{37}NO_5$ $[M]^+$ calcd.: 527.2666, found: 527.2680.

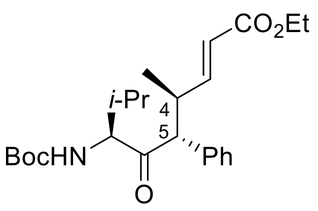

(4*S*,5*R*)-14a

(4*S*,5*R*)-14a, [*Tab.1*, *entry 5*]**:** According to GP 6 ketone **11a**[1] (87.0 mg, 300 µmol) was reacted with $ZnCl_2$ (57.2 mg, 420 µmol), LDA (1.10 mL, 615 µmol, [Pd(allyl)Cl_2] (1.5 mg, 4.10 µmol), PPh_3 (4.20 mg, 16.0 µmol) and carbonate (*S*)-**7a** (36.1 mg, 167 µmol) in THF (2.00 mL). Aqueous work up and flash chromatography (silica, petroleum ether/EtOAc 97:3, 95:5, 90:10) gave rise to (4*S*,5*R*)-**14a** (83.0 mg, 199 µmol, 99 %, *dr* 93:7) as a colorless solid, m.p.

114−117 °C. $[\alpha]_D^{20}$ = −111.5 (c = 1.00, $CHCl_3$). ^{1}H-NMR (400 MHz, $CDCl_3$): δ = 7.30−7.20 (m, 5 H), 6.60 (dd, *J* = 15.6, 8.0 Hz, 1 H), 5.59 (dd, *J* = 15.6, 1.0 Hz, 1 H), 4.87 (d, *J* = 9.5 Hz, 1 H), 4.25 (dd, *J* = 9.5, 5.4 Hz, 1 H), 4.07 (qd, *J* = 7.1, 1.5 Hz, 2 H), 3.77 (d, *J* = 10.4 Hz, 1 H), 3.20 – 3.11 (m, 1 H), 2.02 (m, 1 H), 1.47 (s, 9 H), 1.20 (t, *J* = 7.1 Hz, 3 H), 1.12 (d, *J* = 6.5 Hz, 3 H), 0.72 (d, *J* = 6.8 Hz, 3 H), 0.47 (d, *J* = 6.8 Hz, 3 H) ppm. ^{13}C-NMR (100 MHz, $CDCl_3$): δ = 207.6, 166.3, 155.9, 150.5, 135.4, 128.9, 128.8, 127.7, 121.4, 79.9, 65.0, 62.0, 60.1, 40.1, 28.3, 28.3, 19.8, 18.3, 16.4, 14.1 ppm. (4*S*,5*S*)-**14a** (*selected signals*): ^{1}H-NMR (400 MHz, $CDCl_3$): δ = 6.86 (dd, *J* = 15.6, 8.3 Hz, 1 H), 5.89 (d, *J* = 15.6 Hz, 1 H), 3.71 (d, *J* = 10.5 Hz, 1 H), 1.36 (s, 9 H), 0.94 (d, *J* = 6.7 Hz, 3 H), 0.81 (d, *J* = 6.8 Hz, 3 H), 0.68 (d, *J* = 6.8 Hz, 3 H) ppm. HPLC (Reprosil 100 Chiral-NR 8 µm, *n*-hexane/*i*PrOH 90:10, 1.5 mL/min, 210 nm): t_R(4*S*,5*S*)-**14a** = 8.61 min, t_R(4*S*,5*R*)-**14a** = 9.68 min. HRMS (CI) calcd for: $C_{24}H_{35}NO_5$ [M+H]$^+$: 418.2588, found: 418.2592.

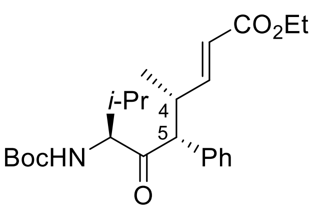

(4*R*,5*R*)-14a

(4*R*,5*R*)-14a, [*Tab.1*, *entry 7*]**:** According to GP 6 ketone **11a**[1] (109 mg, 374 µmol) was reacted with $ZnCl_2$ (66.3 mg, 486 µmol) LDA (1.36 mL, 767 µmol), [Pd(allyl)Cl_2] (1.8 mg, 4.99 µmol), PPh_3 (5.23 mg, 19.9 µmol) and carbonate (*R*)-**7a** (54.1 mg, 250 µmol) in THF (2.40 mL). Aqueous work up and flash chromatography (silica, petroleum ether/EtOAc 96:4, 93:7, 90:10) gave rise to (4*R*,5*R*)-**14a** (103 mg, 248 µmol, 99 %, *E*/*Z* 87:13, *dr*(*E*) 93:4:3) as a colorless resin. ^{1}H-NMR (400 MHz, $CDCl_3$): δ = 7.36 – 7.19 (m, 5 H), 6.86 (dd, *J* = 15.8, 8.2 Hz), 5.84 (d, *J* = 15.8, 1 H), 4.78 (d, *J* = 9.7 Hz, 1 H), 4.20 – 4.11 (m, 3 H), 3.78 (d, *J* = 10.4 Hz, 1 H), 3.17 – 3.08 (m, 1 H), 2.00 – 1.92 (m, 1 H), 1.45 (s, 9 H), 1.29 (t, *J* = 10.4 Hz, 3 H), 0.83 (d, *J* = 6.7 Hz, 3 H), 0.66 (d, *J* = 6.7 Hz, 3 H), 0.53 (d, *J* = 6.8 Hz, 3 H) ppm. ^{13}C-NMR (100 MHz, $CDCl_3$): δ = 206.9, 166.3, 155.8, 150.7, 135.2, 129.1, 128.9, 127.7, 121.7, 79.8, 64.0, 61.9, 60.2, 39.2, 28.2, 28.2, 19.6, 17.2, 16.6, 14.2 ppm. (4*R*,5*S*)-**14a** (*selected signals*): ^{1}H-NMR (400 MHz, $CDCl_3$): δ = 6.61 (dd, *J* = 15.7, 8.0 Hz, 1 H), 5.59 (d, *J* = 15.6 Hz, 1 H) ppm. (4*S*,5*R*,Z)-**14a** (*selected signals*): ^{1}H-NMR (400 MHz, $CDCl_3$): δ = 5.81 (dd, *J* = 11.5, 11.5 Hz, 1 H), 5.48 (d, *J* = 11.5 Hz, 1 H), 4.91 (d, *J* = 9.2 Hz, 1 H), 1.46 (s, 9 H), 1.08 (d, *J* = 6.5 Hz, 3 H), 0.71 (d, *J* = 6.6 Hz, 3 H), 0.48 (d, *J* = 6.8 Hz, 3 H) ppm. ^{13}C-NMR (100 MHz, $CDCl_3$): δ = 151.2, 129.0, 128.5, 127.4, 19.0, 16.5 ppm. HPLC (Reprosil 100 Chiral-NR 8 µm, *n*-hexane/*i*PrOH 99.5:0.5 - 95:5, 40 min, 1.0 mL/min, 210 nm): t_R(4*S*,5*R*,*Z*)-**14a** = 15.37 min, t_R(4*R*,5*R*)-**14a** = 16.68 min, t_R(4*S*,5*R*)-**14a** = 30.49 min, t_R(4*R*,5*S*)-**14a** = 38.68 min. HRMS (CI) calcd for: $C_{24}H_{36}NO_5^+$ [M+H]$^+$: 418.2588, found: 418.2598.

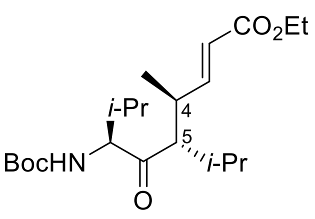

(4*S*,5*S*)-14b

(4*S*,5*S*)-14b, [*Tab.1*, *entry 15*]**:** According to GP 6 ketone **11b**[1] (58.0mg, 225 µmol) was reacted with $ZnCl_2$ (40.0 mg, 293 µmol), LDA (820 µL, 461 mmol), [Pd(allyl)Cl_2] (1.1 mg, 3.01 µmol), PPh_3 (3.15 mg, 12.0 µmol) and carbonate (*S*)-**7a** (32.5 mg, 150 µmol) in THF (1.60 mL). Aqueous work up and flash chromatography (silica, petroleum ether/EtOAc 97:3, 95:5, 90:10) gave rise to (4*S*,5*S*)-**14b** (22.5 mg, 58.7 µmol, 70 %, *dr* 99:1) as a colorless solid, m.p.

101−103 °C. $[\alpha]_D^{20}$ = –48.1 (c = 1.00, $CHCl_3$). ^{1}H-NMR (400 MHz, $CDCl_3$): δ = 6.87 (dd, *J* = 15.6, 1 H), 5.81 (d, *J* = 15.6 Hz, 1 H), 4.86 (d, *J* = 9.8 Hz, 1 H), 4.22 – 4.13 (m, 3 H), 2.77 (m, 1 H), 2.69 (dd, *J* = 7.3, 6.2 Hz, 1 H), 2.26 – 2.16 (m, 1 H), 2.01 – 1.92 (m, 1 H), 1.44 (s, 9 H), 1.29 (t, *J* = 7.1 Hz, 3 H), 0.99 – 0.98 (m, 9 H), 0.88 (d, *J* = 6.7 Hz, 3 H), 0.79 (d, *J* = 6.7 Hz, 3 H) ppm. ^{13}C-NMR (100 MHz, $CDCl_3$): δ = 210.7, 166.4, 155.8, 151.5, 121.0, 79.7, 65.1, 60.3, 58.9, 36.8, 28.6, 28.3, 27.8, 20.7, 20.6, 17.0, 19.7, 16.5, 14.2 ppm. LC-MS (Luna, 1.0 mL/min, 210 nm, MeCN/H_2O 50:50 – 65:35, 30 min): t_R(4*S*,5*S*)-**14b** = 18.02 min, t_R(4*S*,5*R*)-**14b** = 23.03 min. HRMS (CI) calcd for: $C_{21}H_{37}NO_5$ $[M+H]^+$: 384.2744, found: 384.2752.

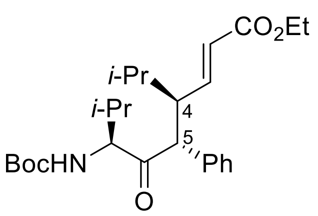

(4*S*,5*R*)-14c

(4*S*,5*R*)-14c, [*Tab.1*, *entry 17*]**:** According to GP 6 ketone **11a**[1] (87.0 mg, 300 µmol) was reacted with $ZnCl_2$ (53.1 mg, 390 µmol), LDA (1.1 mL, 615 µmol), [Pd(allyl)Cl_2] (1.5 mg, 4.01 µmol), PPh_3 (4.20 mg, 16.0 µmol) and carbonate (*S*)-**7b** (48.9 mg, 200 µmol) in THF (2.00 mL). Aqueous work up and flash chromatography (silica, petroleum ether/EtOAc 98:2, 96:4, 93:7, 90:10) gave rise to (4*S*,5*R*)-**14c** (84.7 mg, 190 µmol, 95 %, *dr* >99:1) as a colorless solid, m.p. 87–80 °C. $[\alpha]_D^{20}$ = –144.9 (c = 1.00, $CHCl_3$). ^{1}H-NMR (400 MHz, $CDCl_3$): δ = 7.15 – 7.26 (m, 5 H), 6.51 (dd, *J* = 15.5, 10.9 Hz, 1 H), 5.48 (d, *J* = 15.5 Hz, 1 H), 4.82 (d, *J* = 9.5 Hz, 1 H), 4.25 (dd, *J* = 9.5, 6.4 Hz, 1 H), 4.14-4.01 (m, 3 H), 3.00 (ddd, *J* = 10.8, 10.8, 2.9 Hz, 1 H), 2.00 – 1.92 (m, 1 H), 1.85 – 1.77 (m, 1 H), 1.47 (s, 9 H), 1.20 (t, *J* = 7.2 Hz, 3 H), 0.92 (dd, *J* = 6.2 Hz, 6 H), 0.64 (d, *J* = 6.6 Hz, 3 H), 0.52 (d, *J* = 6.8 Hz, 3 H) ppm. ^{13}C-NMR (100 MHz, $CDCl_3$): δ = 207.0, 165.8, 155.8, 146.0, 135.3, 129.3, 128.7, 127.5, 124.2, 79.9, 64.1, 60.0, 58.5, 51.1, 29.6, 28.4, 28.3, 22.0, 19.6, 16.7, 16.6, 14.1 ppm. LC-MS (Luna, 1.0 mL/min, 210 nm, MeCN/H_2O 50:50 – 65:35, 30 min): t_R(4*S*,5*R*)-**14c** = 23.47 min. HRMS (CI) calcd for: $C_{26}H_{39}NO_5$ $[M+H_2]^+$: 447.2979, found: 447.2977.

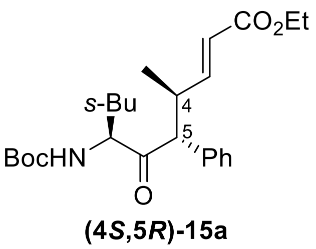

(4*S*,5*R*)-15a

(4*S*,5*R*)-15a, [*Tab.1*, *entry 8*]**:** According to GP 6 ketone **12a**[1] (68.8 mg, 225 µmol) was reacted with $ZnCl_2$ (39.9 mg, 293 µmol), LHMDS (1 M, 560 µL, 560 mmol), [Pd(allyl)Cl_2] (1.1 mg, 3.00 µmol), PPh_3 (3.15 mg, 12.0 µmol) and carbonate (*S*)-**7a** (32.5 mg, 150 µmol) in THF (1.60 mL). The reaction was quenched at −25 °C. Flash chromatography (silica, petroleum ether/ EtOAc 95:5, 90:10, 80:20) gave rise to (4*S*,5*R*)-**15a** (59.6 mg, 138 µmol, 92 %, *dr* 98:2) as a colorless solid, m.p. 100−102 °C. $[\alpha]_D^{20}$ = −118.7 (c = 1.00, $CHCl_3$). ^{1}H-NMR (400 MHz, $CDCl_3$): δ = 7.31 – 7.21 (m, 5 H), 6.61 (dd, *J* = 15.7, 8.2 Hz, 1 H), 5.59 (dd, *J* = 15.7 0.7 Hz, 1 H), 4.85 (d, *J* = 9.7 Hz, 1 H), 4.30 (dd, *J* = 9.7, 5.4 Hz, 1 H), 4.08 (qd, *J* = 7.1, 1.6 Hz, 2 H), 3.78 (d, *J* = 10.4 Hz, 1 H), 3.20 – 3.11 (m, 1 H), 1.76 (m, 1 H), 1.47 (s, 9 H), 1.21 (t, *J* = 7.2 Hz, 3 H), 1.13 (d, *J* = 6.4 Hz, 3 H), 0.92 – 0.76 (m, 2 H), 0.73 (d, *J* = 6.7 Hz, 3 H), 0.47 (t, *J* = 7.3 Hz, 3 H) ppm. ^{13}C-NMR (100 MHz, $CDCl_3$): δ = 207.6, 166.3, 155.9, 150.5, 135.5, 128.9, 128.8, 127.7, 121.3, 79.8, 65.1, 62.0, 60.1, 40.1, 34.6, 28.3, 23.5, 18.3, 16.1, 14.1, 10.9 ppm. (4*S*,5*S*)-**15a** (*selected signals*): ^{1}H-NMR (400 MHz, $CDCl_3$): δ = 6.86 (dd, *J* = 15.7, 8.3 Hz, 1 H), 5.89 (d, *J* = 15.7 Hz, 1 H) ppm. HPLC (Reprosil 100 Chiral-NR 8 µm, *n*-hexane/*i*PrOH 99.5:0.5 - 95:5,

40 min, 1.0 mL/min, 220 nm): t_R(4*S*,5*S*)-**15a** = 25.20 min, t_R(4*S*,5*R*)-**15a** = 30.24 min. HRMS (CI) calcd for: $C_{25}H_{37}NO_5$ $[M+H_3]^+$: 434.2901, found: 434.2905.

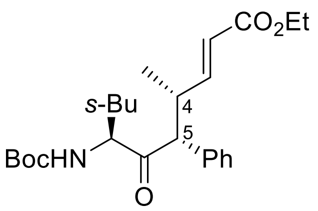

(4*R*,5*R*)-15a

(4*R*,5*R*)-15a, [*Tab.1*, *entry 10*]**:** According to GP 6 ketone **12a**[1] (115 mg, 375 µmol) was reacted with $ZnCl_2$ (66.4.2 mg, 488 µmol), LDA (767 µL, 750 mmol) in THF (1.20 mL), [Pd(allyl)Cl_2] (1.8 mg, 4.99 µmol), PPh_3 (5.20 mg, 19.8 µmol) and carbonate (*R*)-**7a** (54.1 mg, 250 µmol) in THF (2.40 mL). Aqueous work up and flash chromatography (silica, petroleum ether/EtOAc 97:3, 95:5, 90:10) gave rise to (4*R*,5*R*)-**15a** (108 mg, 247 µmol, 99 %, *E*/*Z* 88:12, *dr* 93:5:2) as a colorless resin. ^{1}H-NMR (400 MHz, $CDCl_3$): δ = 7.36 – 7.10 (m, 5 H), 6.85 (dd, *J* = 15.7, 8.1 Hz, 1 H), 5.84 (d, *J* = 15.7 Hz, 1 H), 4.20 – 4.09 (m, 3 H), 3.76 (d, *J* = 10.5 Hz, 1 H), 3.13 (m, 1 H), 1.78 – 1.67 (m, 1 H), 1.45 (s, 9 H), 1.29 (t, *J* = 9.3 Hz, 2 H), 1.02 – 0.75 (m, 5 H), 0.65 (d, *J* = 6.7 Hz, 3 H), 0.52 (t, *J* = 7.5 Hz, 3 H) ppm. ^{13}C-NMR (100 MHz, $CDCl_3$): δ = 206.8, 166.4, 155.7, 150.7, 135.3, 129.1, 128.8, 127.7, 121.7, 79.8, 63.8, 61.9, 60.2, 39.2, 34.5, 28.2, 23.6, 17.2, 15.9, 14.2, 10.9 ppm. (4*R*,5*S*)-**15a** (*selected signals*): ^{1}H-NMR (400 MHz, $CDCl_3$): δ = 6.60 (dd, *J* = 15.6, 8.1 Hz, 1 H), 5.58 (d, *J* = 15.6 Hz, 1 H) ppm. (4*S*,5*R*,*Z*)-**15a** (*selected signals*): ^{1}H-NMR (400 MHz, $CDCl_3$): δ = 5.47 (d, *J* = 11.6 Hz, 1 H), 4.87 (d, *J* = 8.9 Hz, 1 H), 1.46 (s, 9 H), 0.69 (d, *J* = 6.7 Hz, 3 H), 0.48 (t, *J* = 7.6 Hz, 3 H) ppm. ^{13}C-NMR (100 MHz, $CDCl_3$): δ = 151.2, 129.0, 128.5, 127.4, 119.6, 28.3, 19.0, 14.2 ppm. HPLC (Reprosil 100 Chiral-NR 8 µm, *n*-hexane/*i*PrOH 99.5:0.5 - 95:5, 40 min, 1.0 mL/min, 220 nm): t_R(4*S*,5*R*,*Z*)-**15a** = 15.43 min, t_R(4*R*,5*R*)-**15a** = 16.64 min, t_R(4*S*,5*R*)-**15a** = 30.52 min, t_R(4*R*,5*S*)-**15a** = 37.81 min. HRMS (CI) calcd for: $C_{25}H_{37}NO_5$ $[M+H]^+$: 432.2744, found: 432.2755.

IV.b Table 2

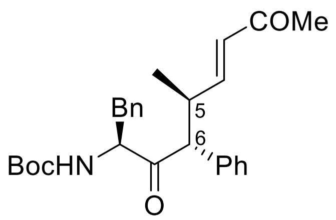

(5*S*,6*R*)-16a

(5*S*,6*R*)-16a [*Tab.2*, *entry 1*]**:** According to GP 6 ketone **10a**[1] (49.9 mg, 0.147 µmol) was reacted with $ZnCl_2$ (30.1 mg, 221 µmol), LDA (0.53 mL, 301 µmol), (0.7 mg, 1.96 µmol), PPh_3 (2.06 mg, 7.84 µmol) and carbonate (*S*)-**8a** (21.0 mg, 98.0 µmol) in THF (1.00 mL). Aqueous work up and flash chromatography (silica, petroleum ether/EtOAc 95:5, 9:1, 85:15, 8:2) gave rise to (5*S*,6*R*)-**16a** (44.2 mg, 93.9 µmol, 96 %, *dr* 95:5) as a colorless solid, m.p. 78−80 °C. $[\alpha]_D^{20}$ = −107.4 (c = 1.00, $CHCl_3$). ^{1}H-NMR (400 MHz, $CDCl_3$): δ = 7.32−7.10, 6.85 − 6.80 (m, 10 H), 6.41 (dd, *J* = 16.1, 8.0 Hz, 1 H), 5.83 (d, *J* = 16.1 Hz, 1 H), 4.80 (d, *J* = 8.4 Hz, 1 H), 4.48 (ddd, *J* = 8.0, 8.0, 6.1 Hz, 1 H), 3.91 (d, *J* = 10.0 Hz, 1 H), 3.23 – 3.13 (m, 1 H), 2.83 (dd, *J* = 14.2, 6.1 Hz, 1 H), 2.68 (dd, *J* = 14.2, 7.9 Hz, 1 H), 2.02 (s, 3 H), 1.42 (s, 9 H), 1.14 (d, *J* = 6.6 Hz, 3 H) ppm. ^{13}C-NMR (100 MHz, $CDCl_3$): δ = 206.8, 198.5, 155.3, 149.9, 136.2, 135.3, 130.9, 129.2, 129.0, 128.5, 128.3, 127.9, 126.6, 80.2, 62.1, 59.5, 39.2, 36.2, 28.2, 26.6, 18.2 ppm. HPLC (Reprosil 100 Chiral-NR 8 µm, *n*-hexane/iPrOH 90:10, 30 min, 1.5 mL/min, 220 nm): t_R(5*S*,6*S*)-**16a** = 17.44 min, t_R(5*S*,6*R*)-**16a** = 22.21 min. HRMS (CI) calcd for: $C_{27}H_{33}NO_4$ $[M+H]^+$: 436.2482, found: 436.2488.

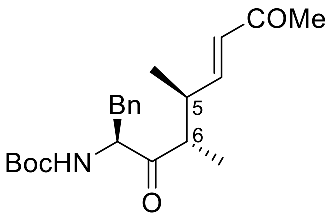

(5*S*,6*S*)-16b

(5*S*,6*S*)-16b, [*Tab.2*, *entry 6*]**:** According to GP 6 ketone **10b**[1] (83.0 mg, 299 µmol) was reacted with $ZnCl_2$ (53.1 mg, 390 µmol), LDA (1.1 mL, 615 µmol), [Pd(allyl)Cl_2] (1.5 mg, 4.01 µmol), PPh_3 (4.20 mg, 16.0 µmol) and carbonate (*S*)-**8a** (37.1 mg, 199 µmol) in THF (2.00 mL). Aqueous work up and flash chromatography (silica, petroleum ether/EtOAc 95:5, 92:8, 90:10) gave rise to (5*S*,6*S*)-**16b** (32.1 mg, 86 µmol, 43 %, *dr* 6:94) as a pale yellow resin.^{1}H-NMR (400 MHz, $CDCl_3$): δ = 7.32 – 7.16 (m, 5 H), 6.57 (dd, *J* = 16.0, 8.1 Hz, 1 H), 6.01 (d, *J* = 16.0 Hz, 1 H), 5.04 (d, *J* = 8.5 Hz, 1 H), 4.71 – 4.64 (m, 1 H), 3.04 (dd, *J* = 13.8, 7.2 Hz, 1 H), 2.91 (dd, *J* = 13.8, 6.7 Hz, 1 H), 2.74 – 2.66 (m, 1 H), 2.58 (dq, *J* = 6.7, 6.7 Hz, 1 H), 2.23 (s, 3 H), 1.42 (s, 9 H), 1.04 (d, *J* = 6.6 Hz, 3 H), 0.86 (d, *J* = 6.8 Hz, 3 H) ppm. ^{13}C-NMR (100 MHz, $CDCl_3$): δ = 211.3, 198.4, 155.1, 148.8, 136.1, 131.4, 129.2, 128.6, 127.0, 80.0, 59.6, 48.9, 38.5, 37.7, 28.2, 27.0, 17.7, 12.7 ppm HPLC (Reprosil 100 Chiral-NR 8 µm, *n*-hexane/iPrOH 90:10, 30 min, 1.0 mL/min, 220 nm): t_R(5*S*,6*R*)-**16b** = 26.76 min, t_R(5*S*,6*S*)-**16b** = 29.31 min. HRMS (CI) calcd for: $C_{22}H_{31}NO_4$ $[M+H]^+$: 374.2326, found: 374.2338.

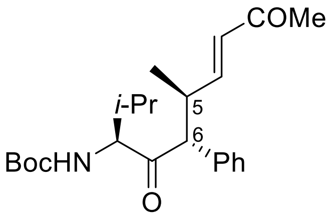

(5*S*,6*R*)-17a

(5*S*,6*R*)-17a, [*Tab.2*, *entry 3*]**:** According to GP 6 ketone **11a**[1] (65.7 mg, 225 µmol) was reacted with $ZnCl_2$ (46.1 mg, 228 µmol), LDA (0.82 mL, 461 µmol), [Pd(allyl)Cl_2] (1.1 mg, 3.01 µmol), PPh_3 (3.15 mg, 12.0 µmol) and carbonate (*S*)-**8a** (32.2 mg, 150 µmol) in THF (1.40 mL). The reaction was quenched at −25 °C, work up and flash chromatography (silica, petroleum ether/EtOAc 90:10, 85:15, 80:20) gave rise to (5S,6*R*)-**17a** (50.2 mg, 130 µmol, 86 %, *dr* 98:2) as a colorless solid, m.p. 116−118 °C. $[\alpha]_D^{20}$ = −146.9 (c = 1.00, $CHCl_3$). ^{1}H-NMR (400 MHz, $CDCl_3$): δ = 7.31 – 7.21 (m, 5 H), 6.41 (dd, *J* = 16.0, 8.0 Hz, 1 H), 5.83 (d, *J* = 16.0 Hz, 1 H), 4.89 (d, *J* = 9.3 Hz, 1 H), 4.25 (dd, *J* = 9.3, 5.4 Hz, 1 H), 3.80 (d, *J* =10.3 Hz, 1 H), 3.20 – 3.11 (m, 1 H), 2.06 – 1.98 (m, 4 H), 1.47 (m, 10 H), 1.13 (d, *J* = 6.5 Hz, 3 H), 0.73 (d, *J* = 6.6 Hz, 3 H), 0.49 (d, *J* = 6.7 Hz, 3 H) ppm. ^{13}C-NMR (100 MHz, $CDCl_3$): δ = 207.4, 198.4, 155.9, 149.7, 135.3, 130.9, 128.8, 128.8, 127.7, 79.9, 65.0, 62.1, 40.1, 28.3, 28.3, 26.6, 19.7, 18.1, 16.4 ppm. (5*S*,6*S*)-**17a** (selected signals): ^{1}H-NMR (400 MHz, $CDCl_3$): δ = 6.74 (dd, *J* = 16.1, 8.0 Hz, 1 H), 6.13 (d, *J* = 16.1 Hz, 1 H), 4.56 (d, *J* = 8.7 Hz, 1 H) ppm. LC-MS (Luna, 1.0 mL/min, 254 nm, MeCN/H_2O 40:60 – 50:50, 40 min): t_R(5*S*,6*R*)-**17a** = 24.94 min, t_R(5*S*,6*S*)-**17a** = 30.36 min. HRMS (CI) calcd for: $C_{23}H_{33}NO_4^+$ $[M]^+$: 387.2404, found: 387.2410.

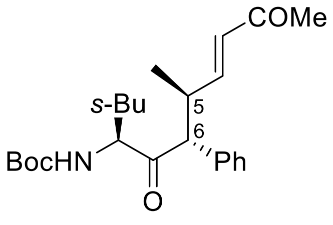

(5*S*,6*R*)-18a

(5*S*,6*R*)-18a [*Tab.2*, *entry 5*]**:** According to GP 6 ketone **12a**[1] (44.9 mg, 147 µmol) was reacted with $ZnCl_2$ (30.1 mg, 221 µmol), LHMDS (0.37 mL, 370 µmol), [Pd(allyl)Cl_2] (0.72 mg, 1.96 µmol), PPh_3 (2.1 mg, 7.84 µmol) and carbonate (*S*)-**8a** (21.0 mg, 98.0 µmol) in THF (1.00 mL). Aqueous work up and flash chromatography (silica, petroleum ether/EtOAc 85:15, 80:20) gave

rise to (5*S*,6*R*)-**18a** (35.6 mg, 88.7 µmol, 85 %, *dr* 99:1) as a colorless solid, m.p.: 86−88 °C. $[\alpha]_D^{20}$ = −123.6 (c = 1.00, $CHCl_3$). ^{1}H-NMR (400 MHz, $CDCl_3$): δ = 7.31 − 7.21 (m, 5 H), 6.40 (dd, *J* = 16.0, 8.0 Hz, 1 H), 5.82 (dd, *J* = 16.0, 0.7 Hz, 1 H), 4.83 (d, *J* = 9.7 Hz, 1 H), 4.29 (dd, *J* = 9.7, 5.4 Hz, 1 H), 3.78 (d, *J* = 10.4 Hz, 1 H), 3.16 (m, 1 H), 2.02 (s, 3 H), 1.81 – 1. 70 (m, 1 H), 1.47 (s, 9 H), 1.13 (d, *J*= 6.5 Hz, 3 H), 0.92 – 0.77 (m, 2 H), 0.72 (d, *J* = 6.7 Hz, 3 H), 0.46 (t, *J* = 7.3 Hz, 3 H) ppm. ^{13}C-NMR (100 MHz, $CDCl_3$): δ = 207.4, 198.4, 155.9, 149.7, 135.4, 130.9, 128.8, 127.7, 79.8, 65.0, 62.1, 40.2, 34.5, 28.2, 26.6, 23.5, 18.1, 16.0, 10.9. (5*S*,6*R*)-**18a** (selected signals): ^{1}H-NMR (400 MHz, $CDCl_3$): δ = 6.73 (dd, *J* = 16.0, 8.0 Hz, 1 H), 6.06 (d, *J* = 16.0 Hz, 1 H) ppm. LC-MS (Luna, 1.0 mL/min, 190 nm, MeCN/H_2O 40:60 – 50:50, 40 min): t_R(5*S*,6*R*)-**18a** = 31.64 min, t_R(5*S*,6*S*)-**18a** = 36.54 min. HRMS (CI) calcd for: $C_{24}H_{35}NO_4$ $[M+H]^+$: 402.2639, found: 402.2566.

IV.c Table 3

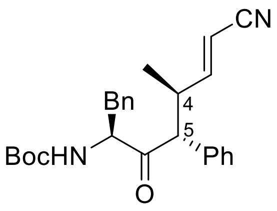

(4*S*,*E*)-19a

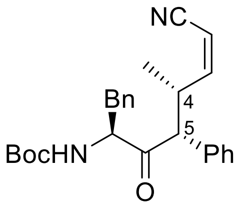

(4*R*,*Z*)-19a

(4*R*,*Z*)-19a and **(4*S*,*E*)-19a** [*Tab. 3, entry 2*]**:** According to GP 6 ketone **10a**[1] (227 mg, 669 µmol) was reacted with $ZnCl_2$ (119 mg, 870 µmol), LDA (2.43 mL, 1.37 mmol), [Pd(allyl)Cl_2] (3.3 mg, 8.92 µmol), PPh_3 (9.36 mg, 35.7 µmol) and carbonate (*S,E*)-**9a** (88.0 mg, 446 µmol) in THF (5.00 mL). Aqueous work up and flash chromatography (silica, petroleum ether/EtOAc 90:10, 85:15) gave rise to a mixture of (4*R*,*Z*)-**19a** and (4*S*,*E*)-**19a** (166 mg, 397 µmol, 89 %, *E*/*Z* 60:40, *dr*(*E*) 91:9, *dr*(*Z*) 96:4) as a colorless solid. The isomers were separated by further flash chromatography (silica, petroleum ether/EtOAc 85:15) to obtain (4*R*,*Z*)-**19a** (70.0 mg, 167 µmol) and (4*S*,*E*)-**19a** (92.1 mg, 220 µmol) as colorless solids. (4*R*,*Z*)-**19a:** $[\alpha]_D^{20}$ = −182.5 (c = 1.00, $CHCl_3$). M.p.: 94−96 °C. ^{1}H-NMR (400 MHz, $CDCl_3$): δ = 7.33 − 7.12, 6.85 − 6.81 (m, 10 H), 6.40 (dd, *J* = 10.4, 10.4 Hz, 1 H), 5.29 (d, *J* = 10.9 Hz, 1 H), 4.77 (d, *J* = 7.8 Hz, 1 H), 4.31 (ddd, *J* = 7.8, 7.8, 6.2 Hz, 1 H), 3.97 (d, *J* = 10.0 Hz, 1 H), 3.54 – 3.44 (m, 1 H), 2.67 (dd, *J* = 14.2, 5.4 Hz, 1 H), 2.59 (dd, *J* = 14.2, 8.6 Hz, 1 H), 1.40 (s, 9 H), 0.89 (d, *J* = 6.9 Hz, 3 H) ppm. ^{13}C-NMR (100 MHz, $CDCl_3$): δ = 206.7, 157.4, 155.5, 136.3, 134.6, 129.2, 129.2, 129.0, 128.4, 128.1, 126.6, 126.6, 115.9, 99.3, 80.2, 62.1, 59.2, 39.3, 36.4, 28.2, 17.6 ppm. (4*S*,*E*)-**19a**: $[\alpha]_D^{20}$ = −176.5 (c = 1.00, $CHCl_3$). M.p.: 138−140 °C. ^{1}H-NMR (400 MHz, $CDCl_3$): δ = 7.29 – 7.27, 7.15–7.12, 6.84 – 6.82 (m, 10 H), 6.37 (dd, *J* = 16.4, = 8.1 Hz, 1 H), 5.11 (dd, *J* = 16.4, 1.1 Hz, 1 H), 4.77 (d, *J* = 8.1 Hz, 1 H), 4.40 (ddd, *J* = 8.1, 8.1, 6.1 Hz, 1 H), 3.89 (d, *J* = 9.9 Hz, 1 H), 3.22 – 3.16 (m, 1 H), 2.77 (dd, *J* = 14.2, 5.9 Hz, 1 H), 2.65 (dd, *J* = 14.2, 8.3 Hz, 1 H), 1.41 (s, 9 H), 1.12 (d, *J* = 6.5 Hz, 3 H) ppm. ^{13}C-NMR (100 MHz, $CDCl_3$): δ = 206.4, 157.6, 155.3, 136.1, 134.6, 129.3, 129.0, 129.0, 128.4, 128.2, 126.7, 117.2, 99.8, 80.3, 61.6, 59.3, 39.9, 36.2, 28.2, 17.7 ppm. HPLC (Reprosil 100 Chiral-NR 8 µm, *n*-hexane/iPrOH 97:3 – 93:7, 47 min, 1.0 mL/min, 210 nm) t_R(4*R*,5*S*,*E*)-**19a** = 25.05 min, t_R(4*R*,5*R*,*Z*)-**19a** = 31.60 min, t_R(4*S*,5*R*,*E*)-**19a** = 39.75 min, t_R(4*S*,5*S*,*Z*)-**19a** = 42.43 min. HRMS (CI) calcd for: $C_{26}H_{30}N_2O_3$ $[M+H]^+$: 419.2329, found: 419.2335.

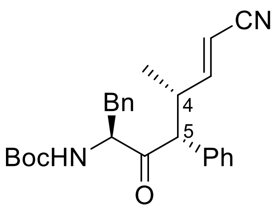

(4*R*,*E*)-19a

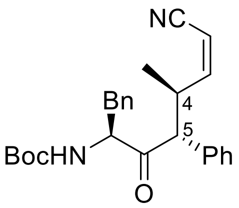

(4*S*,*Z*)-19a

(4*R*,*E*)-19a and **(4*S*,*Z*)-19a**, [*Tab. 3, entry 4*]**:** and According to GP 6 ketone **10a**[1] (102 mg, 300 µmol) was reacted with $ZnCl_2$ (53.2 mg, 391 µmol), LHMDS (0.75 mL, 750 µmol), [Pd(allyl)Cl_2] (1.5 mg, 4.01 µmol), PPh_3 (4.20 mg, 16.0 µmol) and carbonate (*S*,*Z*)-**9a** (39.5 mg, 200 µmol) in THF (2.0 mL). Aqueous work up and flash chromatography (silica, petroleum ether/EtOAc 95:5, 90:10, 80:20) gave rise to (4*R*,*E*)-**19a** and (4*S*,*Z*)-**19a** (79.6 mg, 190 µmol, 95 %, *E*/*Z* 94:6, *dr*(*E*) 98:2) as a colorless solid. The isomers were separated by further flash chromatography (silica, petroleum ether/EtOAc 85:15) to obtain (4*R*,*E*)-**19a** (74.8 mg, 178 µmol) and (4*S*,*Z*)-**19a** (4.8 mg, 11.5 µmol) as colorless solids. (4*R*,*E*)-**19a:** $[\alpha]_D^{20}$ = −173.8 (c = 1.00, $CHCl_3$). M.p.: 115−117 °C. ^{1}H-NMR (400 MHz, $CDCl_3$): δ = 7.31 – 7.28 (m, 3 H), 7.18 – 7.13 (m, 5 H), 6.85 – 6.83 (m, 2 H), 6.70 (dd, *J* = 16.4, 7.9 Hz, 1 H), 5.41 (dd, *J* = 16.4, 1.0 Hz, 1 H), 4.72 (d, *J* = 7.7 Hz, 1 H), 4.40 – 4.35 (m, 1 H), 3.93 (d, *J* = 10.3 Hz, 1 H), 3.18 – 3.08 (m, 1 H), 2.66 – 2.56 (m, 2 H), 1.43 (s, 9 H), 0.82 (d, *J* = 6.8 Hz, 3 H) ppm. ^{13}C-NMR (100 MHz, $CDCl_3$): δ = 206.5, 158.2, 155.5, 136.2, 134.4, 129.3, 129.1, 128.9, 128.4, 128.1, 126.3, 117.5, 100.0, 80.6, 62.1, 58.6, 39.1, 36.4, 28.2, 16.7 ppm. (4*S*,*Z*)-**19a:** $[\alpha]_D^{20}$ = +66.2 (c = 1.00, $CHCl_3$). M.p.: 132−134 °C. ^{1}H-NMR (400 MHz, $CDCl_3$): δ = 7.32 − 7.10 (m, 8 H), 6.85 − 6.80 (m, 2 H), 6.08 (dd, *J* = 10.9, 10.9, 8.0 Hz, 1 H), 5.02 (d, *J* = 10.9 Hz, 1 H), 4.79 (d, *J* = 8.3 Hz, 1 H), 4.46 (ddd, *J* = 8.1, 8.1, 6.1 Hz, 1 H), 3.96 (d, *J* = 10.3 Hz, 1 H), 3.73 – 3.63 (m, 1 H), 2.74 (dd, *J* = 14.2, 6.0 Hz, 1 H), 2.64 (dd, *J* = 14.2, 8.1 Hz, 1 H), 1.42 (s, 9 H), 1.16 (d, *J* = 6.5 Hz, 3 H) ppm. ^{13}C-NMR (100 MHz, $CDCl_3$): δ = 205.9, 156.6, 155.4, 136.2, 135.0, 129.2, 129.0, 129.0, 128.3, 128.0, 126.6, 115.6, 99.3, 80.2, 61.8, 59.1, 39.1, 36.2, 28.2, 18.8 ppm. HPLC (Reprosil 100 Chiral-NR 8 µm, *n*-hexane/iPrOH 90:10, 1.5 mL/min, 220 nm): t_R(4*R*,5*R*,*E*)-**19a** = 13.49 min, t_R(4*S*,5*R*,*Z*)-**19a** = 15.97 min, t_R(4*R*,5*S*,*E*)-**19a** = 26.00 min. HRMS (CI) calcd for: $C_{26}H_{30}N_2O_3$ $[M+H]^+$: 419.2329, found: 419.2335.

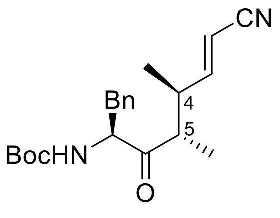

(4*S*,*E*)-19b

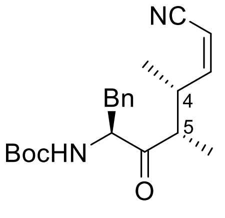

(4*R*,*Z*)-19b

(4*S*,*E*)-19b and **(4*R*,*Z*)-19b**, [*Tab. 3, entry 9*]**:** According to GP 6 ketone **10b**[1] (83.0 mg, 299 µmol) was reacted with $ZnCl_2$ (53.0 mg, 389 µmol), LDA (1.09 mL, 615 mmol) in THF (1.00 mL) the solution of [Pd(allyl)Cl_2] (1.4 mg, 7.65 µmol), PPh_3 (4.20 mg, 16.0 µmol) and carbonate (*S*,*E*)-**9a** (39.3 mg, 199 µmol) in THF (1.00 mL). Aqueous work up and flash chromatography (silica, petroleum ether/EtOAc 95:5, 92:8, 90:10, 85:15) gave rise to (4*R*,*Z*)-**19b** and (4*S*,*E*)-**19b** (62.4 mg, 175 µmol, 88 %, *E*/*Z* 73:27, *dr*(*E*) 5:95, *dr*(*Z*) 3:97) as a colorless resin. The isomers could not be separated by flash chromatography. (4*S*,*E*)-**19b:** ^{1}H-NMR (400 MHz, $CDCl_3$): δ = 7.33 – 7.15 (m, 5 H), 6.49 (dd, *J* = 16.4, 8.3 Hz, 1 H), 5.31 (d, *J* = 16.4 Hz, 1 H), 5.00 (d, *J* = 8.4 Hz, 1 H), 4.67 – 4.61 (m, 1 H), 3.01 (dd, *J* = 13.8, 7.4 Hz, 1 H), 2.90 (d, *J* = 13.8, 6.9 Hz, 1 H), 2.81 – 2.73 (m, 1 H), 2.55 – 2.49 (m, 1 H), 1.41 (s, 9 H), 1.03 (d, *J* = 6.8 Hz, 3 H), 0.83 (d, *J* = 7.0 Hz, 3 H) ppm. ^{13}C-NMR (100 MHz, $CDCl_3$): δ = 210.9, 156.8, 155.2, 136.0, 129.2, 128.7, 127.1, 117.2, 100.6, 80.2, 59.3, 48.8, 38.9, 37.6, 28.2, 17.6,

12.0 ppm. (4*R*,*Z*)-**19b** (*selected signals*): 6.42 (dd, *J* = 10.6, 10.6 Hz, 1 H), 5.31 (d, *J* = 10.6 Hz, 1 H), 1.43 (s, 9 H), 1.08 (d, *J* = 6.8 Hz, 3 H), 0.96 (d, *J* = 6.8 Hz, 3 H) ppm. ^{13}C-NMR (100 MHz, $CDCl_3$): δ = 211.0, 156.6, 155.1, 136.1, 129.3, 126.6, 127.0, 115.7, 99.6, 80.0, 59.9, 48.4, 39.2, 37.5, 28.2, 16.8, 13.3. HPLC (Reprosil 100 Chiral-NR 8 µm, *n*-hexane/iPrOH 97:3, 1.5 mL/min): t_R(4*R*,5*S*,*Z*)-**19b** and t_R(4*R*,5*R*,*Z*)-**19b** = 20.19 min, t_R(4*S*,5*S*,*E*)-**19b** = 26.03 min, t_R(4*S*,5*R*,*E*)-**19b** = 29.57 min. HRMS (CI) calcd for: $C_{21}H_{28}N_2O_3$ [M+H]$^+$: 357.2173, found: 357.2188.

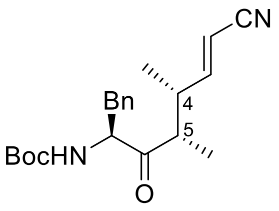

(4*R*,*E*)-19b

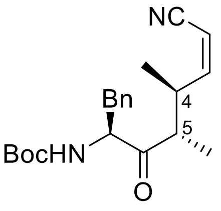

(4*S*,*Z*)-19b

(4*R*,*E*)-19b and **(4*S*,*Z*)-19b** [*Tab. 3, entry 10*]**:** According to GP 6 ketone **10b**[1] (63.3 mg, 228 µmol) was reacted with $ZnCl_2$ (40.4 mg, 297 µmol), LDA (0.83 mL, 615 mmol), [Pd(allyl)Cl_2] (1.1 mg, 3.04 µmol), PPh_3 (3.19 mg, 12.0 µmol) and carbonate (*S*,*Z*)-**9a** (30.0 mg, 152 µmol) in THF (1.00 mL). Aqueous work up and flash chromatography (silica, petroleum ether/EtOAc 95:5, 92:8, 90:10, 85:15) gave rise to (4*S*,*Z*)-**19b** and (4*R*,*E*)-**19b** (43.5 mg, 122 µmol, 80 %, *E*/*Z* 91:9, *dr*(*E*) 3:97, *dr*(*Z*) 5:95) as a colorless resin. The isomers were separated by further flash chromatography (silica, petroleum ether/EtOAc 90:10, 85:15) to obtain (4*R*,*E*)-**19b** (39.6 mg, 111 µmol) as a colorless resin and (4*S*,*Z*)-**19b** (3.9 mg, 10.9 µmol) as a colorless solid. (4*R*,*E*)-**19b:** $[\alpha]_D^{20}$ = −121.4 (c = 1.00, $CHCl_3$). ^{1}H-NMR (400 MHz, $CDCl_3$): δ = 7.33 – 7.16 (m, 5 H), 6.58 (dd, *J* = 16.4, 7.5 Hz, 1 H), 5.26 (dd, *J* = 16.4, 1.2 Hz, 1 H), 4.99 (d, *J* = 8.6 Hz, 1 H), 4.67 – 4.61 (m, 1 H), 2.99 (dd, *J* = 13.7, 7.5 Hz, 1 H), 2.92 (dd, *J* = 13.7, 6.5 Hz, 1 H), 2.78 – 2.70 (m, 1 H), 2.57 (qd, *J* = 6.9, 6.4 Hz, 1 H), 1.42 (s, 9 H), 0.95 (d, *J* = 6.9 Hz, 3 H), 0.83 (d, *J* = 7.0 Hz, 3 H) ppm. ^{13}C-NMR (100 MHz, $CDCl_3$): δ = 210.6, 157.8, 155.2, 136.1, 129.2, 128.7, 127.1, 117.2, 99.9, 80.3, 58.9, 48.1, 38.6, 37.8, 28.2, 14.3, 11.4 ppm. (4*S*,*Z*)-**19b**: M.p.: 120–124 °C. $[\alpha]_D^{20}$ = +12.6 (c = 1.00, $CHCl_3$). ^{1}H-NMR (400 MHz, $CDCl_3$): δ = 7.32 – 7.17 (m, 5 H), 6.27 (dd, *J* = 10.7, 10.7 Hz, 1 H), 5.34 (d, *J* = 11.0 Hz, 1 H), 4.99 (d, *J* = 8.6 Hz, 1 H), 4.65 – 4.59 (m, 1 H), 3.12 – 2.98 (m, 2 H), 2.89 (dd, *J* = 13.8, 7.0 Hz, 1 H), 2.67 (dq, *J* = 7.1, 7.1 Hz, 1 H), 1.41 (s, 9 H), 1.04 (d, *J* = 6.7 Hz, 3 H), 0.89 (d, *J* = 7.0 Hz, 3 H) ppm. ^{13}C-NMR (100 MHz, $CDCl_3$): δ = 210.5, 156.5, 155.2, 136.2, 129.3, 128.6, 127.0, 115.6, 99.9, 80.1, 60.1, 48.4, 39.4, 37.3, 28.2, 17.8, 13.9 ppm. HRMS (CI) calcd for: $C_{21}H_{28}N_2O_3$ [M+H]$^+$: 357.2173, found: 357.2178. HPLC (Chiracel OD-H 5 µm, *n*-hexane/*i*PrOH 95:5, 1.0 mL/min): t_R**(**4*S*,5*S*,*Z*)-**19b** = 17.11 min, t_R(4*R*,5*S*,*E*)-**19b** = 19.04 min, t_R(4*R*,5*R*,*E*)-**19b** = 24.68 min, t_R(4*S*,5*R*,*Z*)-**19b** = 28.00 min. HRMS (CI) calcd for: $C_{21}H_{28}N_2O_3$ [M+H]$^+$: 357.2173, found: 357.2175.

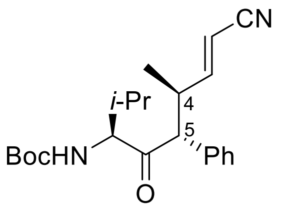

(4*S*,*E*)-20a

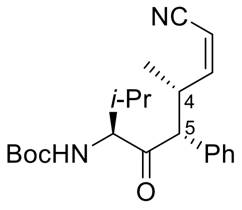

(4*R*,*Z*)-20a

(4*S*,*E*)-20a and **(4*R*,*Z*)-20a** [*Tab. 3, entry 7*]**:** According to GP 6 ketone **11a**[1] (96.0 mg, 329 µmol) was reacted with $ZnCl_2$ (102 mg, 749 µmol), LDA (1.20 mL, 1.62 mmol), [Pd(allyl)Cl_2] (2.2 mg, 5.99 µmol), PPh_3 (6.28 mg, 24.0 µmol) and carbonate (*S*,*E*)-**9a** (50.7 mg, 300 µmol) in THF (2.0 mL). Aqueous work up and flash chromatography (silica, petroleum ether/EtOAc 9:1, 8:2) gave rise to (4*R*,*Z*)-**20a** and (4*S*,*E*)-**20a** (98.1 mg, 265 µmol, 88 %, *E*/*Z* 42:58, *dr*(*E*) 91:9, *dr*(*Z*) >99:1) as a colorless solid. The isomers were separated by further column chromatography (silica, petroleum ether/EtOAc 90:10) to obtain (4*R*,*Z*)-**20a** (55.6 mg, 148 µmol) and (6*S*,*E*)-**20a** (40.5 mg, 109 µmol). (4*R*,*Z*)-**20a:** M.p.: 97–99 °C. $[\alpha]_D^{20}$ = −155.4 (c = 1.00, $CHCl_3$). ^{1}H-NMR (400 MHz, $CDCl_3$): δ = 7.37 – 7.15 (m, 5 H), 6.34 (dd, *J* = 10.6, 10.6 Hz, 1 H), 5.29 (d, *J* = 10.9 Hz, 1 H), 4.85 (d, *J* = 9.5 Hz, 1 H), 4.15 (dd, *J* = 9.5, 5.4 Hz, 1 H), 3.82 (d, *J* = 10.0 Hz, 1 H), 3.52 – 3.42 (m, 1 H), 1.93 – 1.85 (m, 1 H), 1.45 (s, 9 H), 0.89 (d, *J* = 6.8 Hz, 3 H), 0.48 (d, *J* = 6.7 Hz, 3 H) ppm. ^{13}C-NMR (100 MHz, $CDCl_3$): δ = 207.0, 156.5, 156.0, 134.5, 129.0, 129.0, 127.9, 115.6, 99.9, 79.9, 64.6, 62.0, 40.5, 28.3, 28.2, 19.8, 17.6, 16.4 ppm. (4*S*,*E*)-**20a:** M.p.: 87–89 °C. $[\alpha]_D^{20}$ = −153.6 (c = 1.00, $CHCl_3$). ^{1}H-NMR (400 MHz, $CDCl_3$): δ = 7.34 – 7.13 (m, 5 H), 6.36 (dd, *J* = 16.5, 8.0 Hz, 1 H), 5.10 (dd, *J* = 16.4, 1.1 Hz, 1 H), 4.83 (d, *J* = 9.4 Hz, 1 H), 4.20 (dd, *J* = 9.5, 5.7 Hz, 1 H), 3.77 (d, *J* = 10.4 Hz, 1 H), 3.22 – 3.12 (m, 1 H), 2.02 – 1.95 (m, 1 H), 1.46 (s, 9 H), 1.11 (d, *J* = 6.5 Hz, 3 H), 0.70 (d, *J* = 6.6 Hz, 3 H) 0.50 (d, *J* = 6.8 Hz, 3 H) ppm. ^{13}C-NMR (100 MHz, $CDCl_3$): δ = 206.9, 157.4, 155.9, 134.6, 129.1, 128.8, 128.1, 117.2, 99.9, 80.4, 64.8, 61.7, 40.8, 28.3, 28.2, 19.7, 17.7, 16.5 ppm. (5*S*,6*S*,*E*)-**20a** (selected signals). ^{1}H-NMR (400 MHz, $CDCl_3$): δ = 6.64 (dd, *J* = 16.4, 8.4 Hz, 1 H), 5.42 (d, *J* = 16.5 Hz, 1 H), 3.71 (d, *J* = 10.0 Hz, 1 H), 1.38 (s, 9 H), 0.83 (d, *J* = 6.8 Hz, 3 H) ppm. HPLC (Reprosil 100 Chiral-NR 8 µm, *n*-hexane/iPrOH 95:5, 1.5 mL/min) t_R(4*R*,5*R*,*Z*)-**20a** = 14.24 min, t_R(4*S*,5*R*,*E*)-**20a** = 19.04 min, t_R(4*S*,5*S*,*E*)-**20a** = 23.12 min. HRMS (CI) calcd for: $C_{22}H_{30}N_2O_3$ $[M]^+$: 370.2251 found: 370.2254.

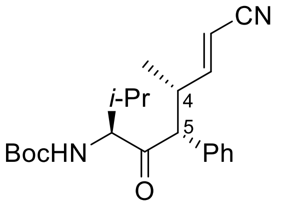

(4*R*,*E*)-20a

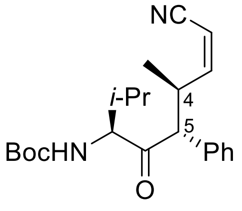

(4*S*,*Z*)-20a

(4*S*,*Z*)-20a and **(4*R*,*E*)-20a** [*Tab. 3, entry 8*]: According to GP 6 ketone **11a**[1] (87.0 mg, 300 µmol) was reacted with $ZnCl_2$ (53.1 mg, 390 µmol), LDA (1.09 mL, 615 mmol), [Pd(allyl)Cl_2] (1.5 mg, 4.01 µmol), PPh_3 (4.20 mg, 16.0 µmol) and carbonate (*S*,*Z*)-**9a** (39.3 mg, 199 µmol) in THF (2.0 mL). Aqueous work up and flash chromatography (silica, petroleum ether/EtOAc 95:5, 93:7, 9:1, 8:2) gave rise to (4*S*,*Z*)-**20a** and (4*R*,*E*)-**20a** (67.8 mg, 183 µmol, 92 %, *E*/*Z* 73:27, *dr*(*E*) 99:1, *dr*(*Z*) 98:2) as a colorless solid. The isomers were separated by further flash chromatography (silica, petroleum ether/EtOAc 85:15) to obtain (4*S*,*Z*)-**20a** (16.8 mg, 47.3 µmol) and (4*R*,*E*)-**20a** (45.5 mg, 127 µmol) as colorless solids . (4*S*,*Z*)-**20a:** $[\alpha]_D^{20}$ = −153.7 (c = 1.00, $CHCl_3$). M.p.: 115−117 °C. ^{1}H-NMR (400 MHz, $CDCl_3$): δ = 7.36 – 7.08 (m, 5 H), 6.07 (dd, *J* = 10.9, 10.9 Hz, 1 H), 5.01 (d, *J* = 10.9 Hz, 1 H), 4.86 (d, *J* = 9.5 Hz, 1 H), 4.20 (dd, *J* = 9.5, 6.1 Hz, 1 H), 3.83 (d, *J* = 10.5 Hz, 1 H, 3-H), 3.71 – 3.60 (m, 1 H), 2.00 – 1.92 (m, 1 H), 1.46 (s, 9 H), 1.13 (d, *J* = 6.5 Hz,

3 H), 0.65 (d, *J* = 6.7 Hz, 3 H), 0.51 (d, *J* = 6.7 Hz, 3 H) ppm. ^{13}C-NMR (100 MHz, $CDCl_3$): δ = 206.3, 156.4, 156.0, 135.0, 128.9, 128.9, 127.9, 115.6, 99.4, 80.0, 64.6, 61.9, 39.9, 28.3, 28.2, 19.6, 18.8, 16.6. (4*S*,5*S*,*Z*)-**20a** (selected signals)*:* ^{1}H-NMR (400 MHz, $CDCl_3$): δ = 5.31 (d, *J* = 11.0 Hz, 1 H), 3.51 – 3.43 (m, 1 H), 1.39 (s, 9 H) ppm. ^{13}C-NMR (100 MHz, $CDCl_3$): δ = 156.0, 99.8 ppm. (4*R*,*E*)-**20a:** $[\alpha]_D^{20}$ = −181.6 (c = 1.00, $CHCl_3$). M.p.: 103 – 105 °C. ^{1}H-NMR (400 MHz, $CDCl_3$): δ = 7.36 – 7.25 (m, 5 H), 6.63 (dd, *J* = 16.4, 8.2 Hz, 1 H), 5.38 (dd, *J* = 16.4, 0.9 Hz, 1 H), 4.77 (d, *J* = 9.5 Hz, 1 H), 4.09 (dd, *J* = 9.5, 6.7 Hz, 1 H), 3.80 (d, *J* = 10.4 Hz, 1 H), 3.17 – 3.08 (m, 1 H), 1.89 – 1.80 (m, 1 H), 1.48 (s, 9 H), 0.83 (d, *J* = 6.9 Hz, 3 H), 0.59 (m, 6 H) ppm. ^{13}C-NMR (100 MHz, $CDCl_3$): δ = 206.5, 157.6, 155.9, 134.3, 129.0, 129.0, 128.0, 117.2, 100.4, 80.2, 63.7, 62.1, 40.0, 28.2, 28.0, 19.5, 16.8, 16.7 ppm. HPLC (Reprosil 100 Chiral-NR 8 µm, *n*-hexane/*i*PrOH 95:5, 1.5 mL/min): t_R(4*R*,5*R*,*E*)-**20a** = 14.05 min, t_R(4*S*,5*R*,*Z*)-**20a** = 17.08 min, t_R(4*S*,5*S*,*Z*)-**20a** = 26.36 min, t_R(4*R*,5*S*,*E*)-**20a** = 29.08 min. HRMS (CI) calcd for: $C_{22}H_{30}N_2O_3$ $[M+H_3]^+$: 373.2486, found: 373.2494.

V. Synthesis of pentasubstituted homopipecolic acids

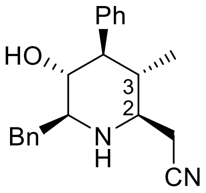

(2*R*)-23a

2-[(2*R*,3*S*,4*R*,5*R*,6*S*)-6-Benzyl-5-hydroxy-3-methyl-4-phenylpiperidin-2-yl]acetonitrile (**23a):** According to GP 1 the cyclization precursor (4*R*,5*R*,*Z*)-**19a** (33.4 mg, 80.0 µmol) was reduced with $NaBH_4$ (6.04 mg, 160 µmol) in THF/MeOH (9:1, 0.80 mL). According to GP 3 the crude alcohol in dichloromethane (0.30 mL) was reacted with TFA (533 µL, 6.92 mmol), diluted with Et_2O and hydrolyzed with satd. $NaHCO_3$. After column chromatography (silica, petroleum ether/EtOAc 60:40) **23a** (21.8 mg, 68.0 µmol, 85 %, *dr*(2*R*/2*S*) 95:5) was obtained as a colorless solid, $[\alpha]_D^{20}$ = –14.3 (c = 1.00, $CHCl_3$), m.p.: 126–128 °C. (The corresponding (*E*)-isomer gave 72%, *dr*(2*R*/2*S*) 61:39, m.p.: 83–89 °C). (2*R*)-**23a**: ^{1}H-NMR (400 MHz, $CDCl_3$): δ = 7.38 – 7.23 (m, 10 H), 3.47 (dd, *J* = 9.4, 9.4 Hz, 1 H), 3.39 (dd, *J* = 13.4, 2.7 Hz, 1 H), 2.85 (ddd, *J* = 9.2, 9.2, 2.8 Hz, 1 H), 2.62 – 2.42 (m, 4 H), 2.25 (dd, *J* = 10.6, 10.6 Hz, 1 H), 1.81 – 1.70 (m, 1 H), 1.59 (bs, 1 H), 0.66 (d, *J* = 6.60, 3 H) ppm. ^{13}C-NMR (100 MHz, $CDCl_3$): δ = 140.2, 138.3, 129.4, 129.0, 128.7, 127.4, 126.6, 117.4, 75.8, 62.8, 58.4, 58.0, 40.4, 38.8, 22.9, 15.3. (2*S*)-**23a**: *(selected signals):* ^{1}H-NMR (400 MHz, $CDCl_3$): δ = 3.48 (dd, *J* = 9.4, 9.4 Hz, 1 H), 3.41 (dd, *J* = 13.6, 3.8 Hz, 1 H), 3.30 (ddd, *J* = 10.2, 4.4, 4.4 Hz, 1 H), 2.97 (ddd, *J* = 9.2, 9.2, 3.2 Hz, 1 H), 2.73 (dd, *J* = 16.8, 10.5 Hz, 1 H), 0.61 (d, *J* = 6.5 Hz, 3 H) ppm. ^{13}C-NMR (100 MHz, $CDCl_3$): δ =139.7, 137.7, 129.2, 129.0, 128.8, 127.5, 126.7, 117.9, 76.5, 55.4, 54.2, 53.3, 39.2, 37.9, 16.5, 16.3. HPLC (Reprosil 100 Chiral-NR 8 µm, *n*-hexane/iPrOH 85:15, 1.5 mL/min, 210 nm): t_R(2*S*)-**23a** = 11.96 min, t_R(2*R*)-**23a** = 13.45 min. HRMS (CI) calcd for: $C_{21}H_{24}N_2O$ $[M+H]^+$: 321.1961 found: 321.1975.

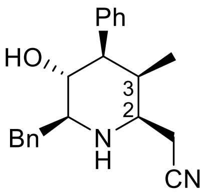

(2*R*)-23b

2-[(2*R*,3*R*,4*R*,5*R*,6*S*)-6-Benzyl-5-hydroxy-3-methyl-4-phenylpiperidin-2-yl]acetonitrile (23b): According to GP 1 the alcohol (4*S*,5*R*,*Z*)-**19a** (19.0 mg, 45.4 µmol) was reduced with $NaBH_4$ (3.4 mg, 91.0 µmol) in THF/MeOH (9:1, 0.45 mL). According to GP 3 the crude product in dichloromethane (0.15 mL) was reacted with TFA (301 µL, 3.91 mmol). After complete conversion, the reaction mixture was diluted with Et_2O, hydrolyzed with satd. $NaHCO_3$ and heated to 30 °C for 1 h. After work up and column chromatography (silica, petroleum ether/EtOAc 60:40) **23b** (10.9 mg, 31.0 µmol, 68 %, *dr*(2*R*/2*S*) 72:28) was obtained as a colorless solid, m.p.: 94–98 °C. (the corresponding (*E*)-isomer gave 73%, *dr*(2*R*/2*S*) 65:35, m.p.: 84–88 °C). (2*R*)-**23b**: ^{1}H-NMR (400 MHz, $CDCl_3$): δ = 7.41 – 7.23 (m, 10 H), 3.92 (dd, *J* = 10.8, 8.8 Hz, 1 H), 3.42 – 3.40 (m, 1 H), 3.25 (ddd, *J* = 7.6, 7.6, 2.8 Hz, 1 H), 2.93 (dd, *J* = 10.9, 4.2 Hz, 1 H), 2.86 (ddd, *J* = 9.0, 9.0, 2.8 Hz, 1 H), 2.64 – 2.58 (m, 1 H), 2.34 (d, *J* = 8.8 Hz, 2 H), 2.09 – 2.02 (m, 1 H), 0.73 (d, *J* = 7.1 Hz, 3 H) ppm. ^{13}C-NMR (100 MHz, $CDCl_3$): δ = 139.2, 138.1, 129.4, 128.8, 128.8, 128.7, 127.1, 126.6, 117.5, 68.5, 63.9, 56.5, 55.1, 39.1, 38.9, 22.0, 6.9. (2*S*)-**23b**: *(selected signals):* ^{1}H-NMR (400 MHz, $CDCl_3$): δ = 3.99 (dd, *J* = 10.8, 9.0 Hz, 1 H), 3.23 – 3.19 (m, 1 H), 3.00 – 2.96 (m, 2 H), 2.81 – 2.68 (m, 2 H), 2.01 – 1.95 (m, 1 H), 0.93 (d, *J* = 7.2 Hz, 3 H) ppm. ^{13}C-NMR (100 MHz, $CDCl_3$): δ = 139.2, 138.1, 129.2, 128.9, 128.7, 126., 69.1, 56.8, 55.6, 49.0, 38.9, 20.9, 15.2. HPLC (Reprosil 100 Chiral-NR 8 µm, *n*-hexane/*i*PrOH 85:15, 20 min, 1.5 mL/min, 220 nm): t_R(2*S*)-**23b** = 14.23 min, t_R(2*R*)-**23b** = 17.12 min. HRMS (CI) calcd for: $C_{21}H_{24}N_2O$ $[M+H]^+$: 321.1961 found: 321.1963.

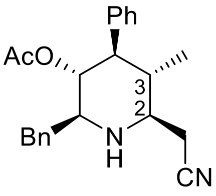

(2*R*)-24a

2-[(2*R*,3*S*,4*R*,5*R*,6*S*)-5-Acetoxy-6-benzyl-3-methyl-4-phenylpiperidin-2-yl]acetonitrile (24a): According to GP 1 the cyclization precursor (4*R*,5*R*,*Z*)-**19a** (54.0 mg, 129 µmol) was reduced with $NaBH_4$ (9.8 mg, 258 µmol) in THF/MeOH (9:1, 2.16 mL). The crude alcohol was reacted with NEt_3 (22.0 µL, 158 µmol), Ac_2O (14.9 µL, 158 µmol) and DMAP (1.6 mg, 13.1 µmol) in dichloromethane (1.0 mL) at 0 °C according to GP 2. In accordance with GP 3 the crude product in dichloromethane (0.37 mL) was reacted with TFA (860 µL, 11.2 mmol). After column chromatography (silica, petroleum ether/EtOAc 80:20) **24a** (28.1 mg, 77.4 µmol, 60 %, *dr*(2*R*/2*S*) 99:1) was obtained as a colorless solid (the corresponding (*E*)-isomer gave 65 %, *dr*(2*R*/2*S*) 71:29). (2*R*)-**24a**: M.p.: 121–124 °C. $[\alpha]_D^{20}$ = +19.2 (c = 1.00, $CHCl_3$). ^{1}H-NMR (400 MHz, $CDCl_3$): δ = 7.36 – 7.18 (m, 10 H), 5.02 (dd, *J* = 10.4, 9.3 H, 1 H), 3.00 (ddd, *J* = 9.5, 9.5, 3.3 Hz, 1 H), 2.93 (dd, *J* = 13.6, 3.3 Hz, 1 H), 2.64 – 2.59 (m, 1 H), 2.59 – 2.45 (m, 3 H), 2.41 (dd, *J* = 11.1, 11.1 Hz, 1 H), 1.84 (m, 1 H), 1.68 (s, 3 H), 0.70 (d, *J* = 6.6 Hz, 3 H) ppm. ^{13}C-NMR (100 MHz, $CDCl_3$): δ = 169.7, 139.6, 137.8, 129.1,129.1 128.7, 128.7, 127.1, 126.7, 117.3, 76.8, 61.2, 58.0, 55.8, 40.7, 38.8, 22.9, 20.4, 15.3 ppm. (2*S*)-**24a**: M.p.: 88–90 °C. $[\alpha]_D^{20}$ = -84.6 (c = 1.00, $CHCl_3$). ^{1}H-NMR (400 MHz, $CDCl_3$): δ = 7.37 – 7.17 (m, 10 H), 5.00 (dd, *J* = 9.6, 9.6 Hz , 1 H), 3.35 (ddd, *J* =10.1, 4.1, 4.1 Hz, 1 H), 3.14 (ddd, *J* = 9.4, 9.4, 3.5 Hz, 1 H), 2.97 (dd, *J* = 13.7, 3.4 Hz, 1 H), 2.76 (dd, *J* = 16.8, 10.4 Hz, 1 H), 2.52 (dd, *J* = 13.8, 9.5 Hz, 1 H) 2.49 – 2.43 (m, 3 H), 1.69 (s, 3 H), 0.64 (d, *J* = 6.1 Hz, 3 H). ^{13}C-NMR (100 MHz, $CDCl_3$): δ = 169.6, 139.0, 137.1, 129.0, 129.0, 128.8, 128.8, 127.2, 126.9, 117.8, 77.2, 54.0, 53.9, 50.6, 39.0, 38.2, 20.4, 16.4, 16.2 ppm. HPLC (Reprosil 100 Chiral-NR 8 µm, *n*-hexane/*i*PrOH 70:30, 1.5 mL/min, 210 nm): t_R(2*S*)-**24a**

= 11.25 min, t_R(2*R*)-**24a** = 13.51 min. HRMS (CI) calcd for: $C_{23}H_{26}N_2O_2$ $[M+H]^+$: 363.2067 found: 363.2063.

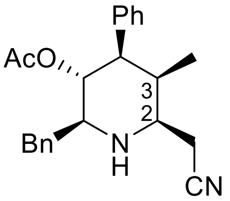

(2*R*)-24b

2-[(2*R*,3*R*,4*R*,5*R*,6*S*)-5-Acetoxy-6-benzyl-3-methyl-4-phenylpiperidin-2-yl]acetonitrile (24b): According to GP 1 the cyclization precursor (4*S*,5*R*,*Z*)-**19a** (11.6 mg, 27.7 µmol) was reduced with $NaBH_4$ (2.1 mg, 55.5 µmol) in THF/MeOH (9:1, 0.48 mL). The crude alcohol was reacted with NEt_3 (4.10 µL, 29.0 µmol), Ac_2O (2.77 µL, 29.0 µmol) and DMAP (0.3 mg, 2.80 µmol) in dichloromethane (0.5 mL) at 0 °C according to GP 2. In accordance with GP 3 the crude product in dichloromethane (0.1 mL) was reacted with TFA (94 µL, 1.22 mmol), diluted with Et_2O and hydrolyzed with satd. $NaHCO_3$. After column chromatography (silica, petroleum ether/EtOAc 80:20), **24b** (5.2 mg, 14.4 µmol, 52 %, *dr*(2*R*/2*S*) 83:17) was obtained as a colorless solid (the corresponding (*E*)-isomer gave 80 %, *dr*(2*R*/2*S*) 62:38). The diastereomers were separated by reversed phase preparative chromatography (*Luna*, 5 µm, C18(2), 100 Å, 250 x 21.2mm, 23 mL/min, ACN/H_2O 10/90 (8 min) –(1 min)→ 40/60 –(20 min)→ 75/25 (2 min) –(1 min)→ 90/10) (2*R*)-**24b**: M.p.: 55–57 °C. $[\alpha]_D^{20}$ = +22.2 (c = 1.00, $CHCl_3$). ^1H-NMR (400 MHz, $CDCl_3$): δ = 7.36 – 7.16 (m, 10 H), 5.42 (dd, *J* = 11.3, 9.2 Hz, 1 H), 3.25 (ddd, *J* = 7.3, 7.3, 2.1 Hz, 1 H), 3.14 (dd, *J* = 11.5, 4.2 Hz, 1 H), 3.02 – 2.96 (m, 1 H), 2.93 (dd, *J* = 13.6, 3.2 Hz, 1 H), 2.60 (dd, *J* = 13.6, 9.8 Hz, 1 H), 2.36 (d, *J* = 7.0 Hz, 2 H), 2.14 – 2.07 (m, 1 H), 1.78 (s, 3 H), 0.79 (d, *J* = 7.1 Hz, 3 H) ppm. ^{13}C-NMR (100 MHz, $CDCl_3$): δ = 170.9, 139.5, 137.7, 129.1, 128.8, 128.3, 128.3, 126.8, 126.6, 117.3, 70.5, 62.4, 56.5, 52.9, 39.4, 38.8, 21.8, 20.8, 6.7 ppm. (2*S*)-**24b**: M.p.: 48–50 °C. $[\alpha]_D^{20}$ = +6.3 (c = 1.00, $CHCl_3$). ^1H-NMR (400 MHz, $CDCl_3$): δ = 7.36 – 7.15 (m, 10 H), 5.45 (dd, *J* = 11.3, 9.2 Hz, 1 H), 3.25 – 3.18 (m, 2 H), 3.13 (ddd, *J* = 9.1, 9.1, 3.5, 1 H), 2.97 (dd, *J* = 13.8, 3.5 Hz, 1 H), 2.78 (d, *J* = 7.5 Hz, 2 H), 2.62 (d, *J* = 13.4, 9.8 Hz, 1 H), 2.08 – 2.01 (m, 1 H), 1.77 (s, 9 H), 0.98 (d, *J* = 7.1 Hz, 3 H) ppm. ^{13}C-NMR (100 MHz, $CDCl_3$): δ = 170.7, 139.1, 137.4, 128.9, 128.8, 128.4, 128.2, 126.8, 126.7, 117.7, 71.1, 55.5, 55.2, 46.8, 39.2, 38.8, 20.8, 20.6, 15.0 ppm. LC-MS: *Luna*, 1.0 mL/min, 190 nm, MeCN/H_2O 30:70 – 50:50, 35 min, t_R(2*S*)-**24b** = 24.49 min, t_R(2*R*)-**24b** = 25.46 min. HPLC (Reprosil 100 Chiral-NR 8 µm, *n*-hexane/*i*PrOH 85:15, 1.0 mL/min, 210 nm): t_R(2*S*)-**24a** = 26.48 min, t_R(2*R*)-**24a** = 28.25 min. HRMS (CI) calcd for: $C_{23}H_{26}N_2O_2$ $[M+H]^+$: 363.2067 found: 363.2069.

VI. NMR spectra, HPLC and GC chromatogramms

(3*S*,4*R*,5*R*,*E*)-3-(*tert*-Butoxycarbonylamino)-4-hydroxy-2-methyl-5,8-diphenyl-oct-7-en (2a)

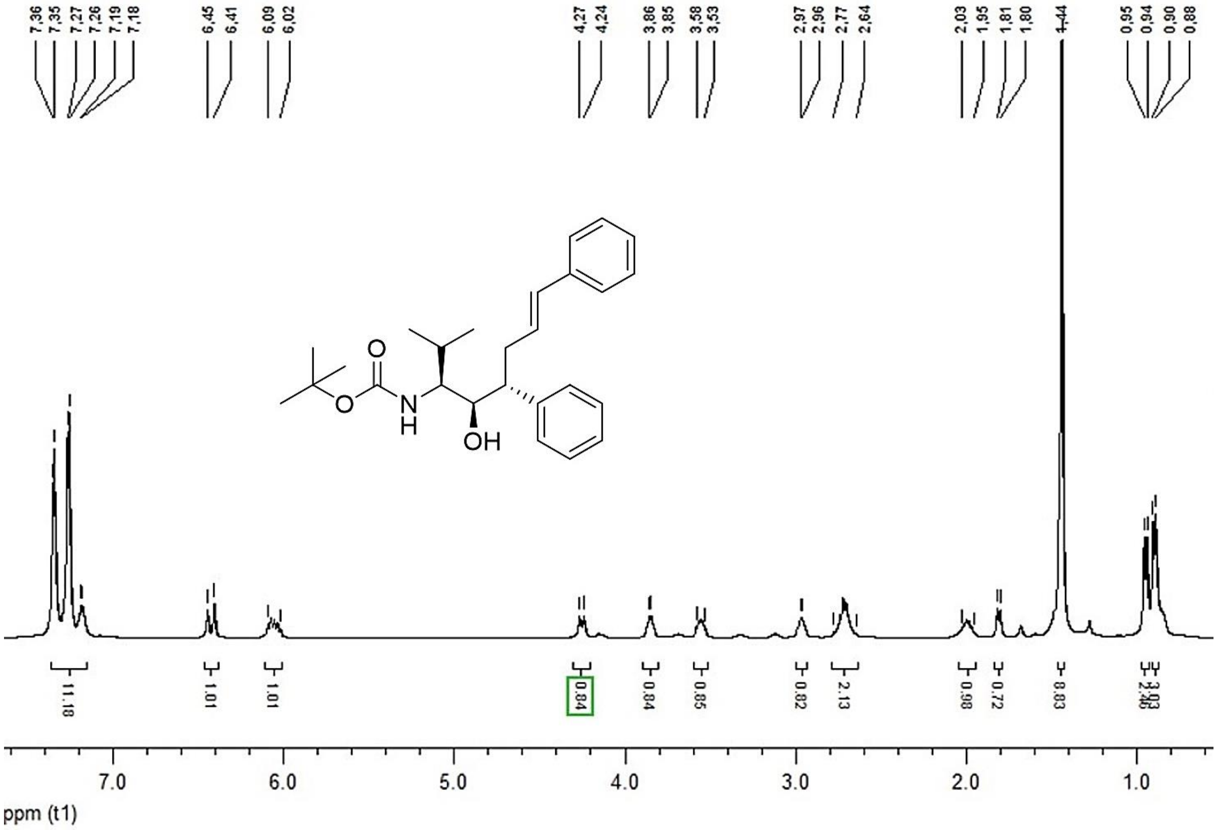

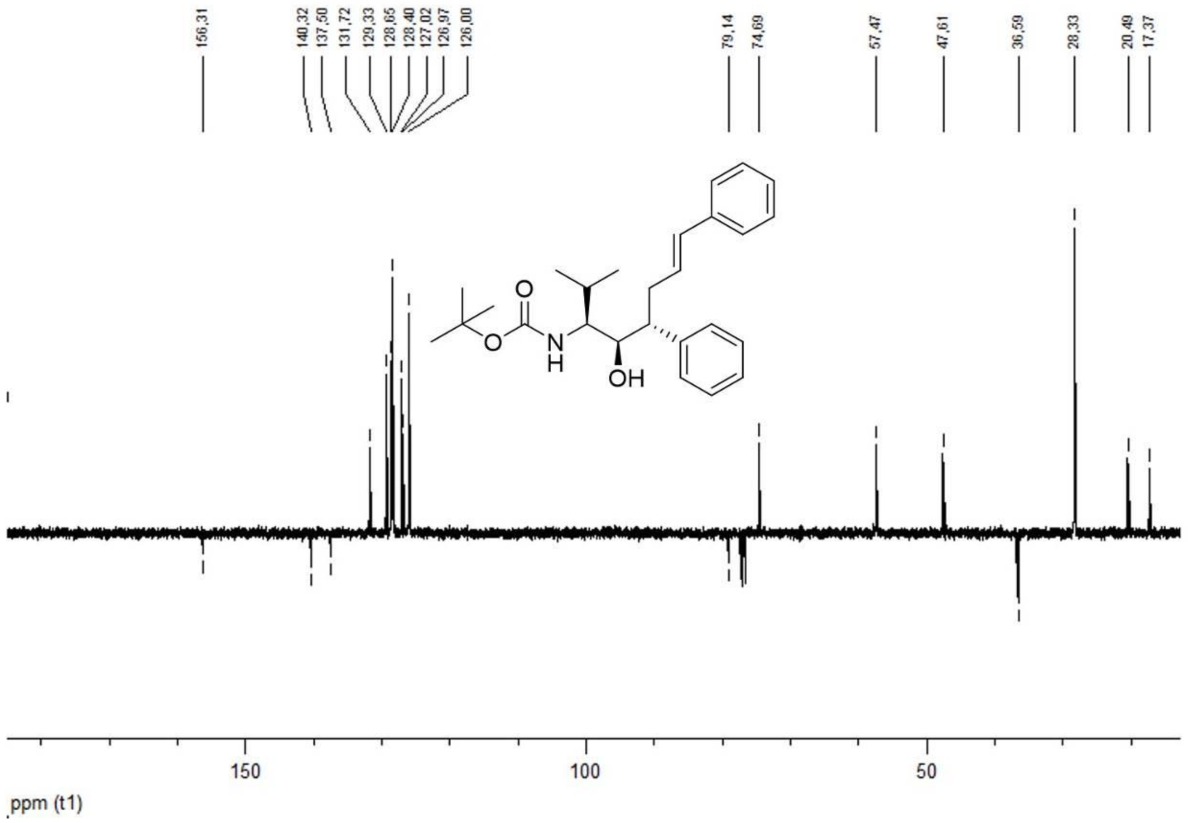

HPLC (2a)

Column: Phenomenex Luna 3u C18(2) RP (50 x 4.6 mm, 100 Å, 5 μm)

Eluent: MeCN/H_2O 80/20, 0.6 ml/min

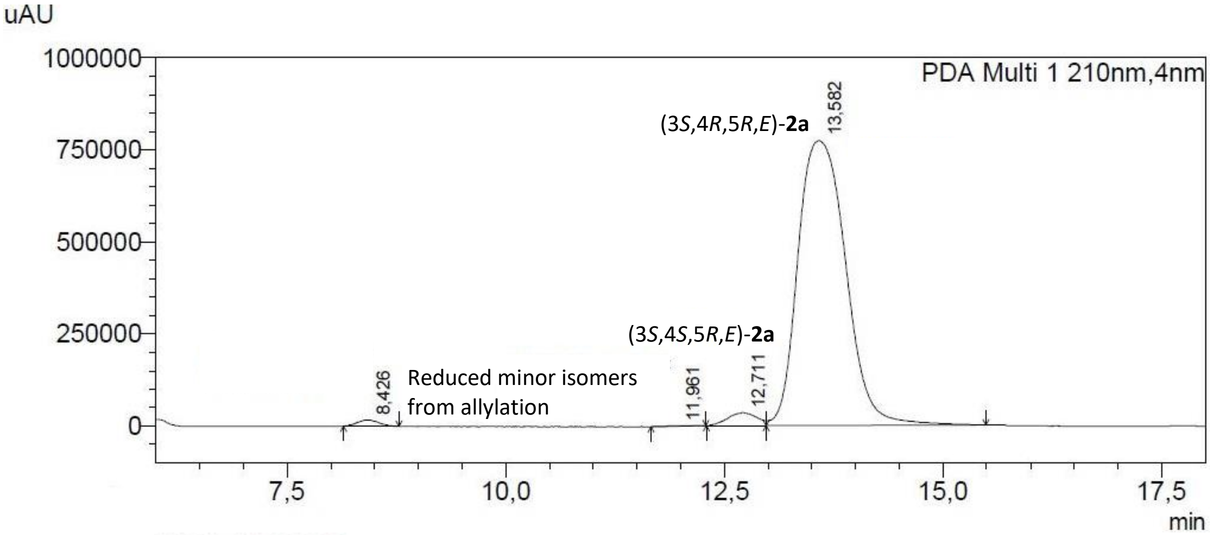

PDA Ch1 210nm

Peak#	Ret. Time	Area	Area%	
1	8,426	281006	0,920	Reduced minor isomers from allylation
2	11,961	34659	0,113	
3	12,711	813680	2,663	(3*S*,4*S*,5*R*,*E*)-**2a**
4	13,582	29422252	96,303	(3*S*,4*R*,5*R*,*E*)-**2a**
Total		30551596	100,000	

Determination of configuration:

(4*S*,5*R*)-5-((*R*,*E*)-1,4-diphenylbut-3-en-1-yl)-4-isopropyloxazolidin-2-on)

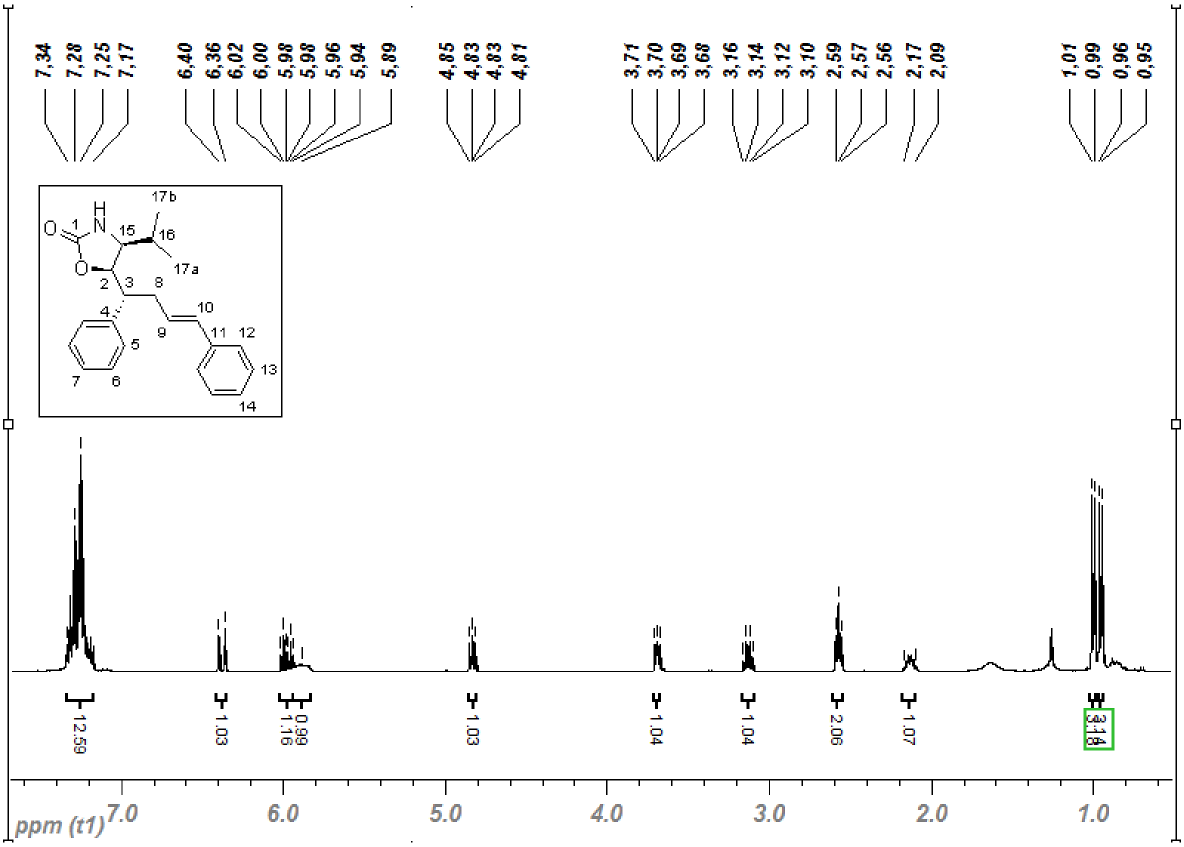

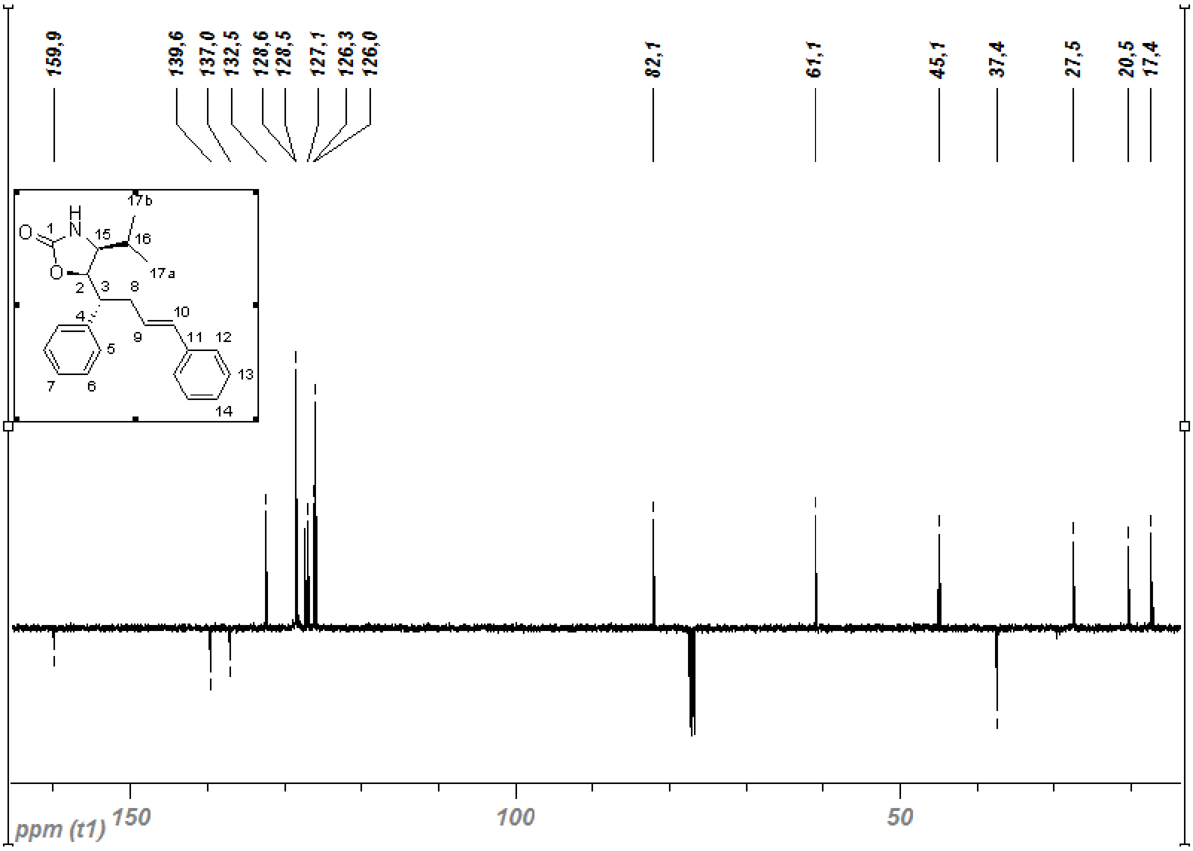

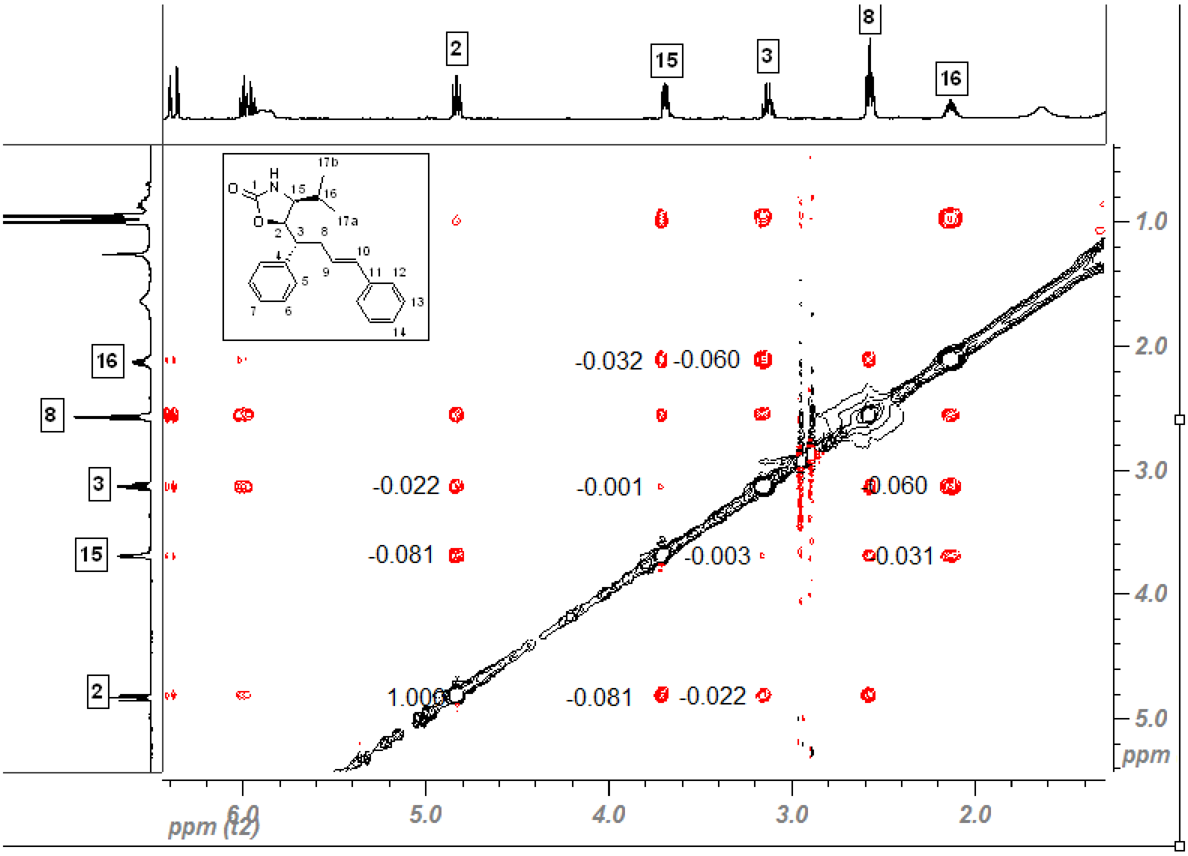
2
15
3
8
16
-0.032
-0.060
-0.022
-0.001
0.060
-0.081
-0.003
0.031
1.000
-0.081
-0.022
ppm (t2)
ppm

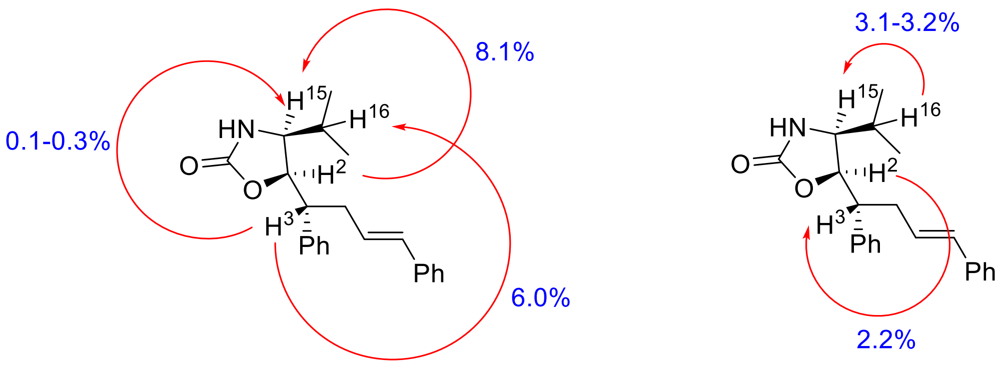
8.1%
0.1-0.3%
6.0%
3.1-3.2%
2.2%

***tert*-Butyl ((3*S*,4*S*,5*R*,6*R*,7*R*,*E*)-5-hydroxy-3,7-dimethyl-6,9-diphenylnon-8-en-4-yl)carbamate (2b)**

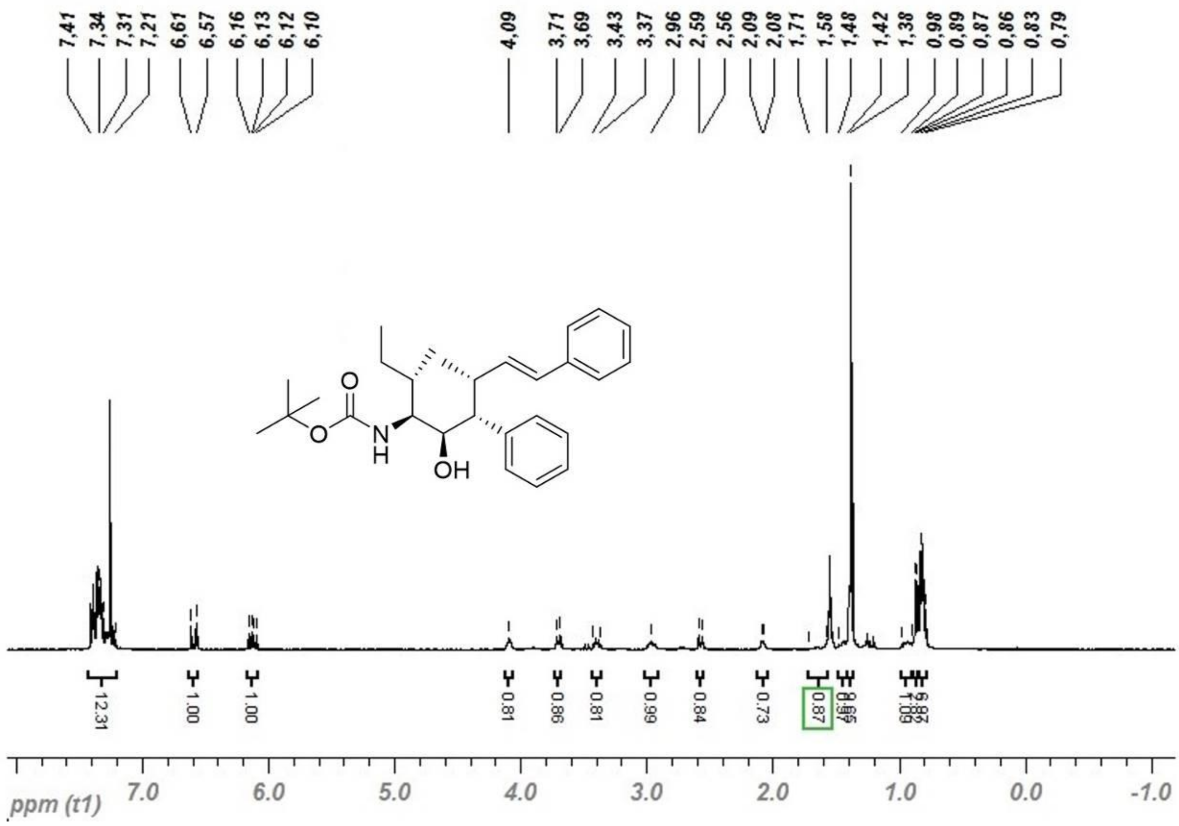

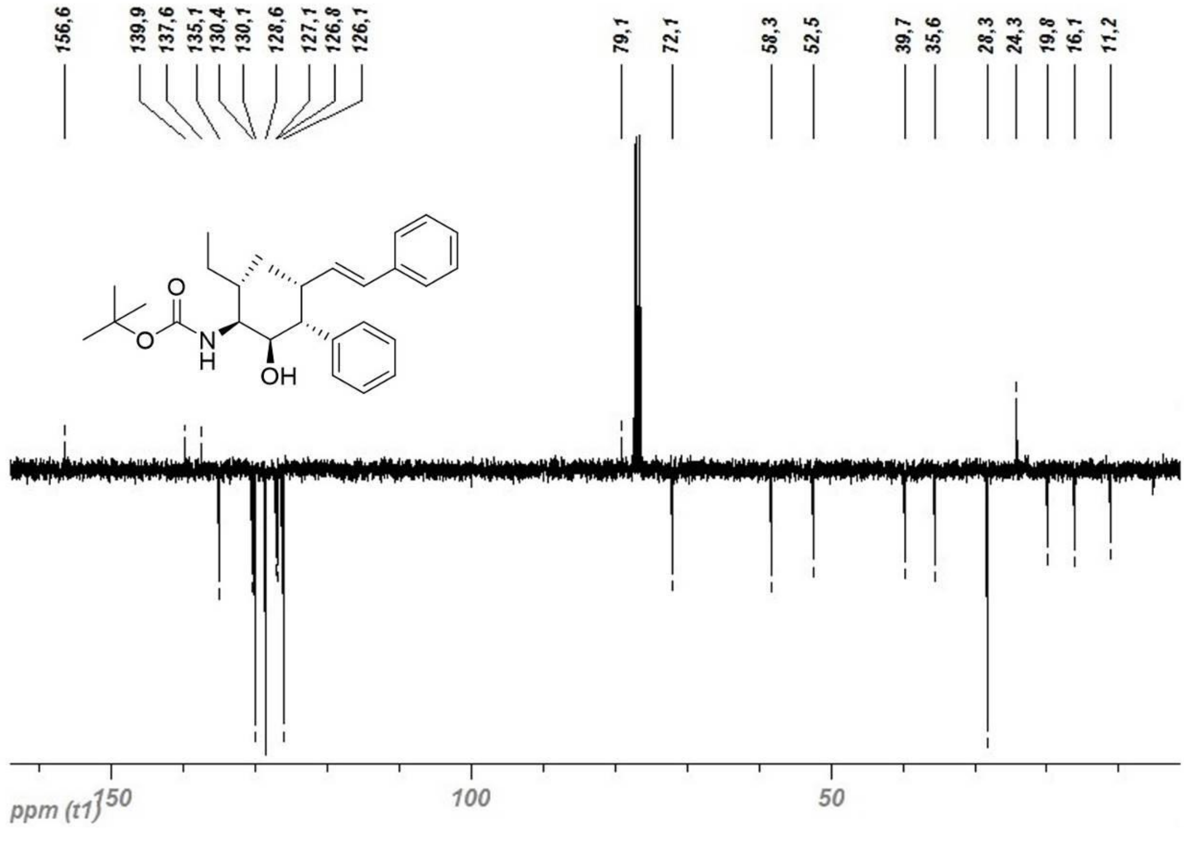

HPLC (2b)
Column: Phenomenex Luna 3u C18(2) RP (50 x 4.6 mm, 100 Å, 5 μm)
Eluent: MeCN/H_2O 65/35, 0.6 ml/min

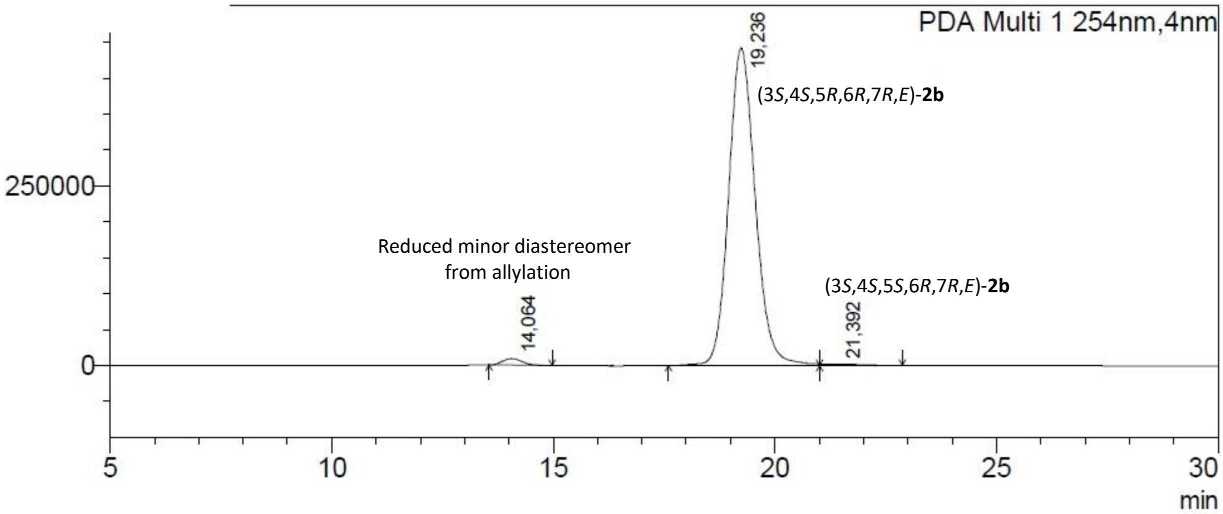

1	14,064	288225	1,523	Reduced minor diastereomer from allylation
2	19,236	18511702	97,837	(3*S*,4*S*,5*R*,6*R*,7*R*,*E*)-**2b**
3	21,392	120989	0,639	(3*S*,4*S*,5*S*,6*R*,7*R*,*E*)-**2b**
Total		18920916	100,000	

Methyl (5*R*,6*R*,7*S*,*E*)-7-((*tert*-butoxycarbonyl)amino)-6-hydroxy-8-methyl-5-phenylnon-2-enoate (3a)

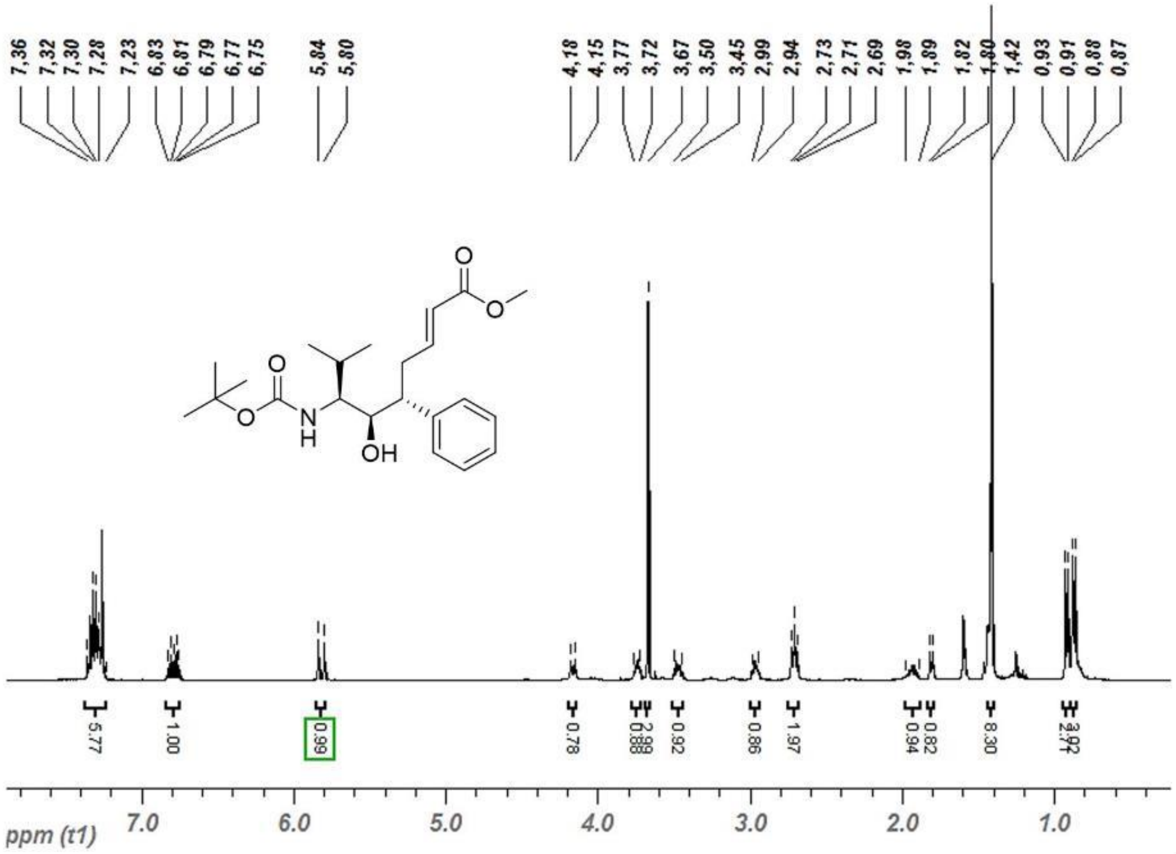

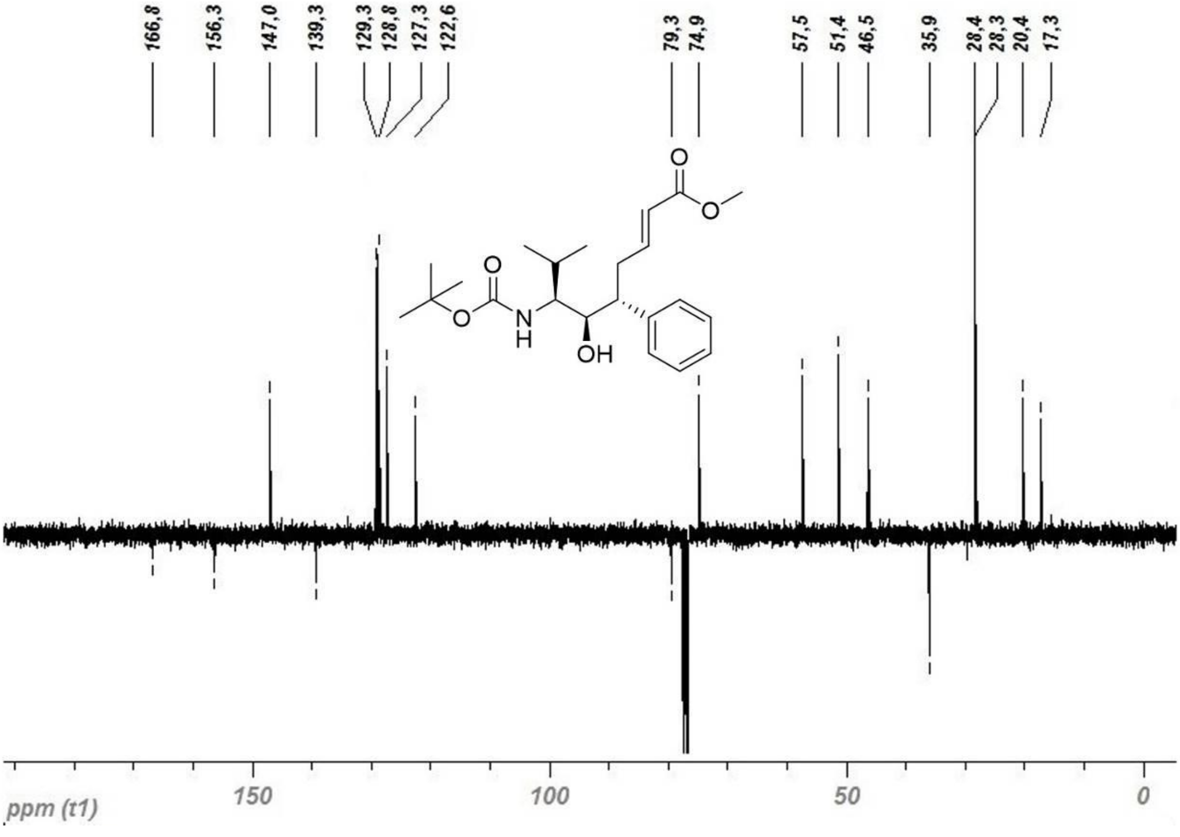

HPLC (3a)
Column: Reprosil 100 Chiral-NR 8 µm
Eluent: Hexane/iPrOH 90:10, 1.5 ml/min, 20°C, 210 nm

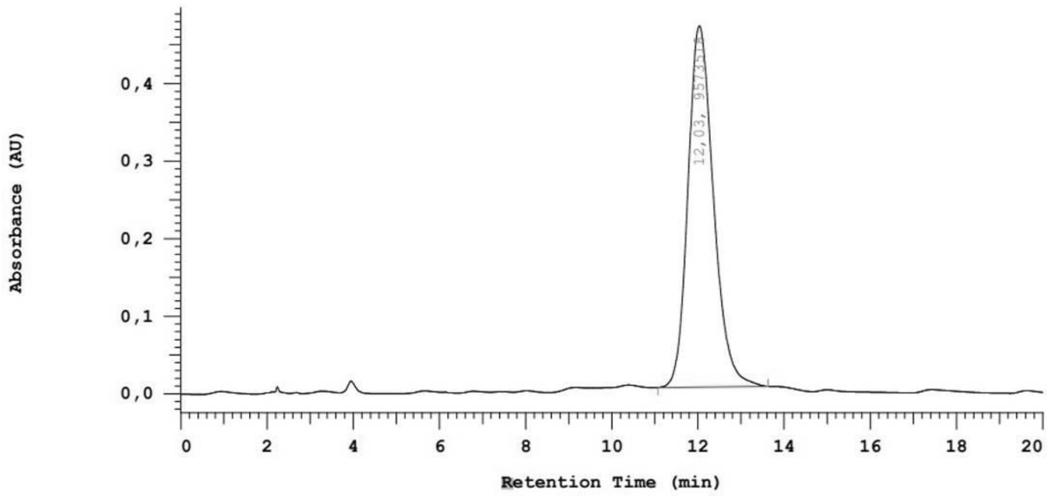

No.	RT	Area	Area %
1	12,03	9573518	100,000
		9573518	100,000

Methyl (5*R*,6*R*,7*S*,*E*)-6-acetoxy-7-[(tert-butoxycarbonyl)amino]-8-methyl-5-phenylnon-2-enoate (4a)

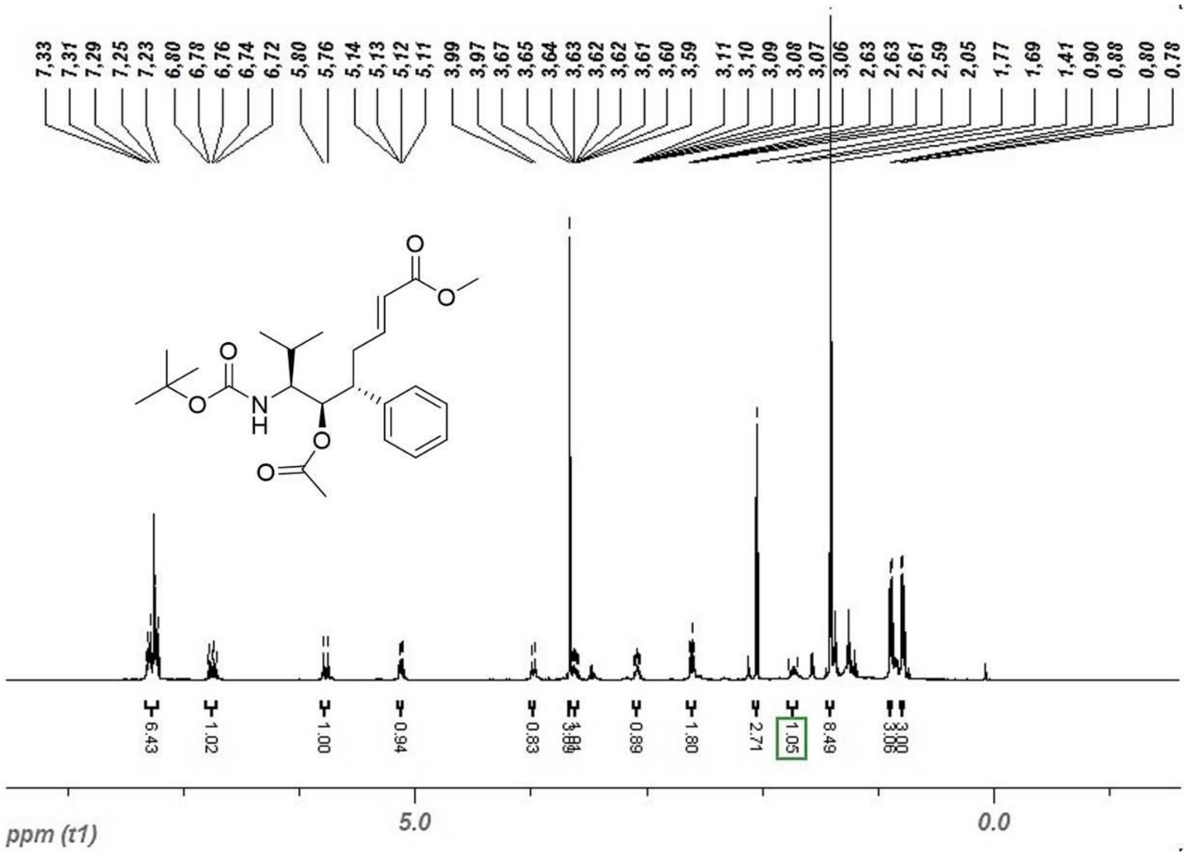

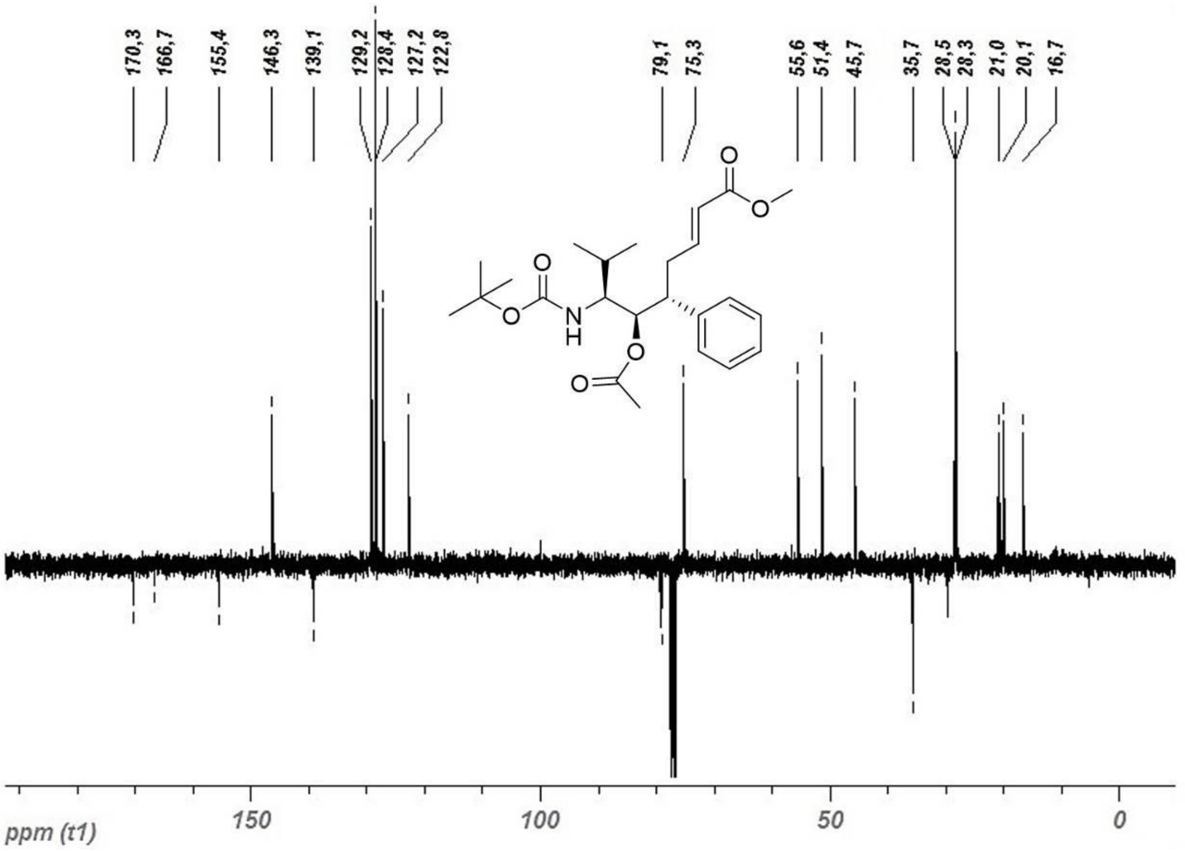

HPLC (4a)
Column: Reprosil 100 Chiral-NR 8 µm
Eluent: Hexane/iPrOH 90:10, 1.0 ml/min, 20°C, 210 nm

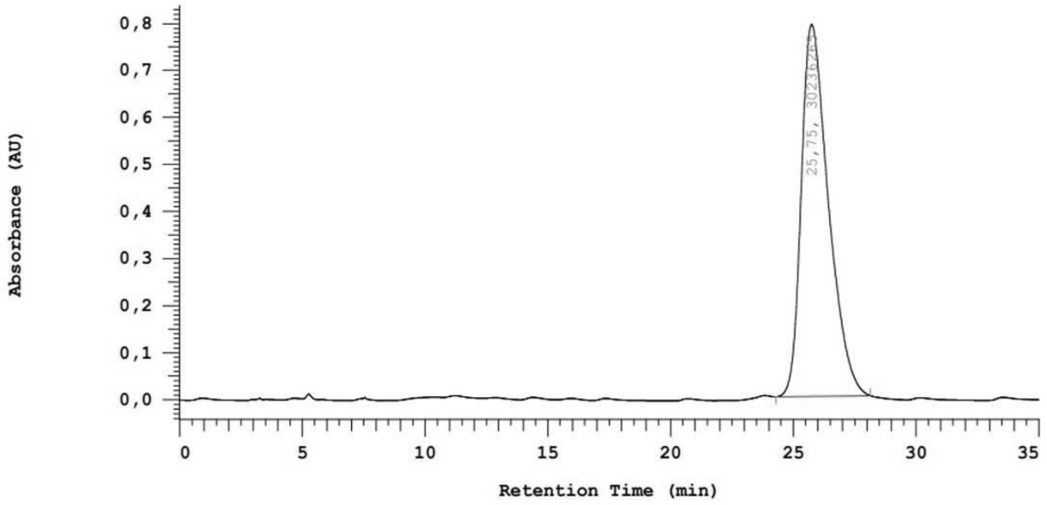

No.	RT	Area	Area %
1	25,75	30236265	100,000
		30236265	100,000

Methyl 2-[(2*S*,4*R*,5*R*,6*S*)-5-acetoxy-6-isopropyl-4-phenylpiperidin-2-yl]acetate [(2*S*)-5a]

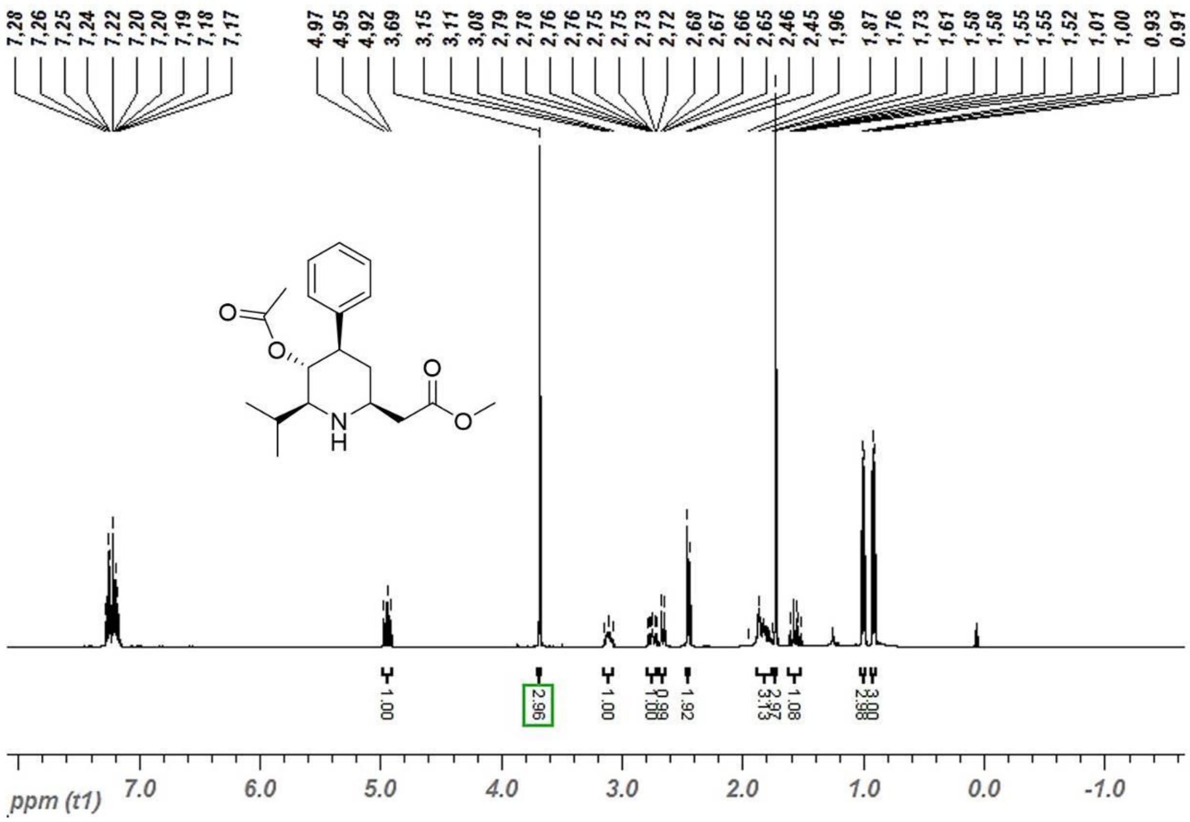

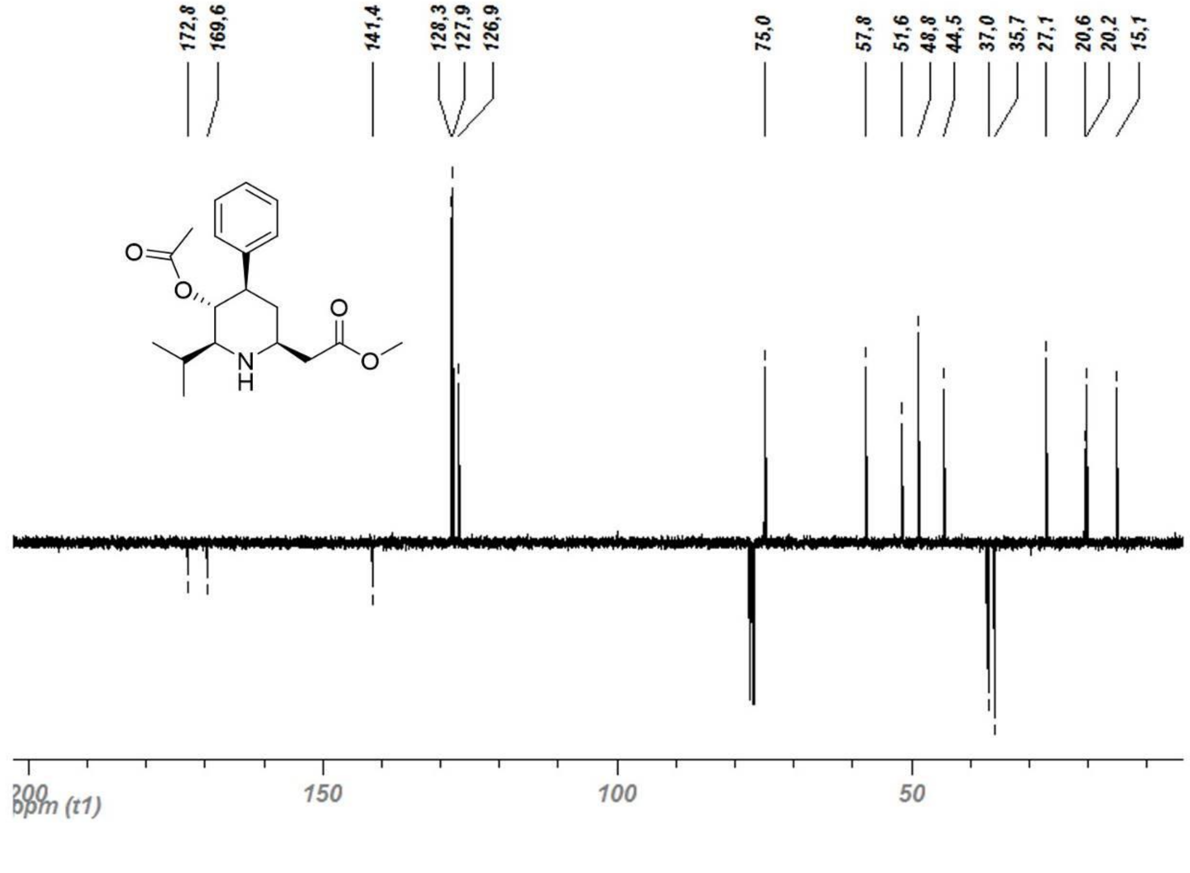

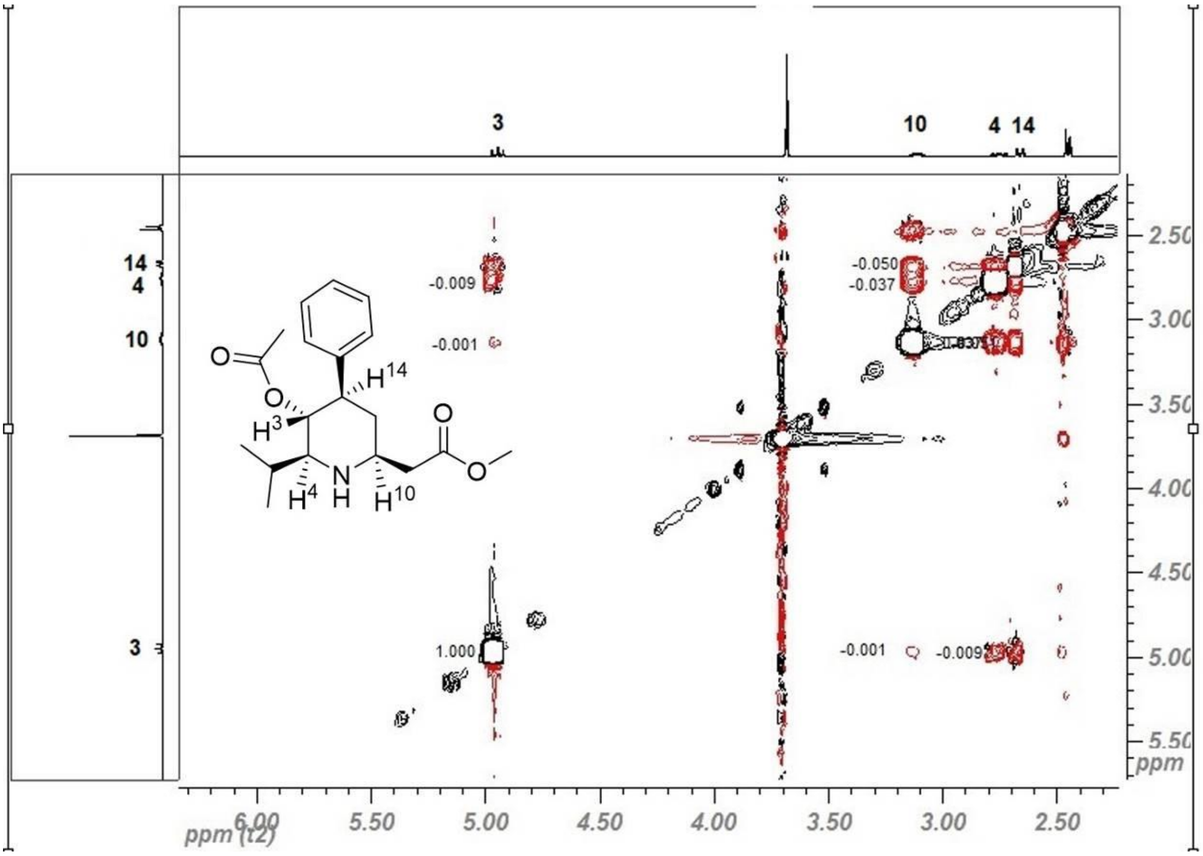

3
10
4 14
-0.009
-0.001
-0.050
-0.037
1.000
-0.001
-0.009
H3
H14
H4
H10
N
H
O
ppm (t2)
6.00
5.50
5.00
4.50
4.00
3.50
3.00
2.50
ppm

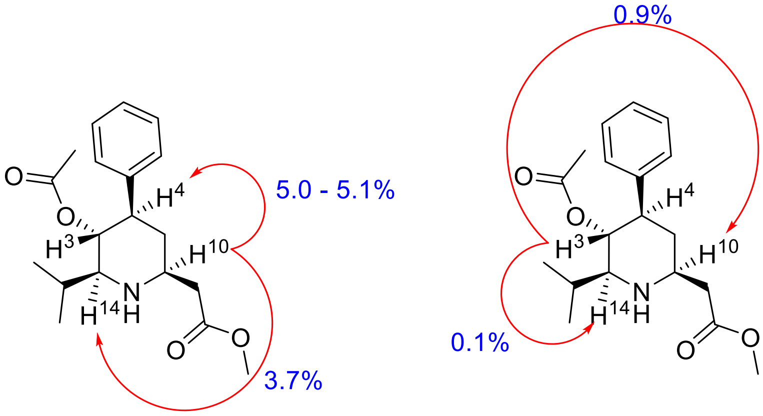

5.0 - 5.1%
3.7%
0.9%
0.1%
H4
H3
H10
H14
N
H
O

Methyl 2-[(2*R*,4*R*,5*R*,6*S*)-5-acetoxy-6-isopropyl-4-phenylpiperidin-2-yl]acetate [(2*R*)-5a]

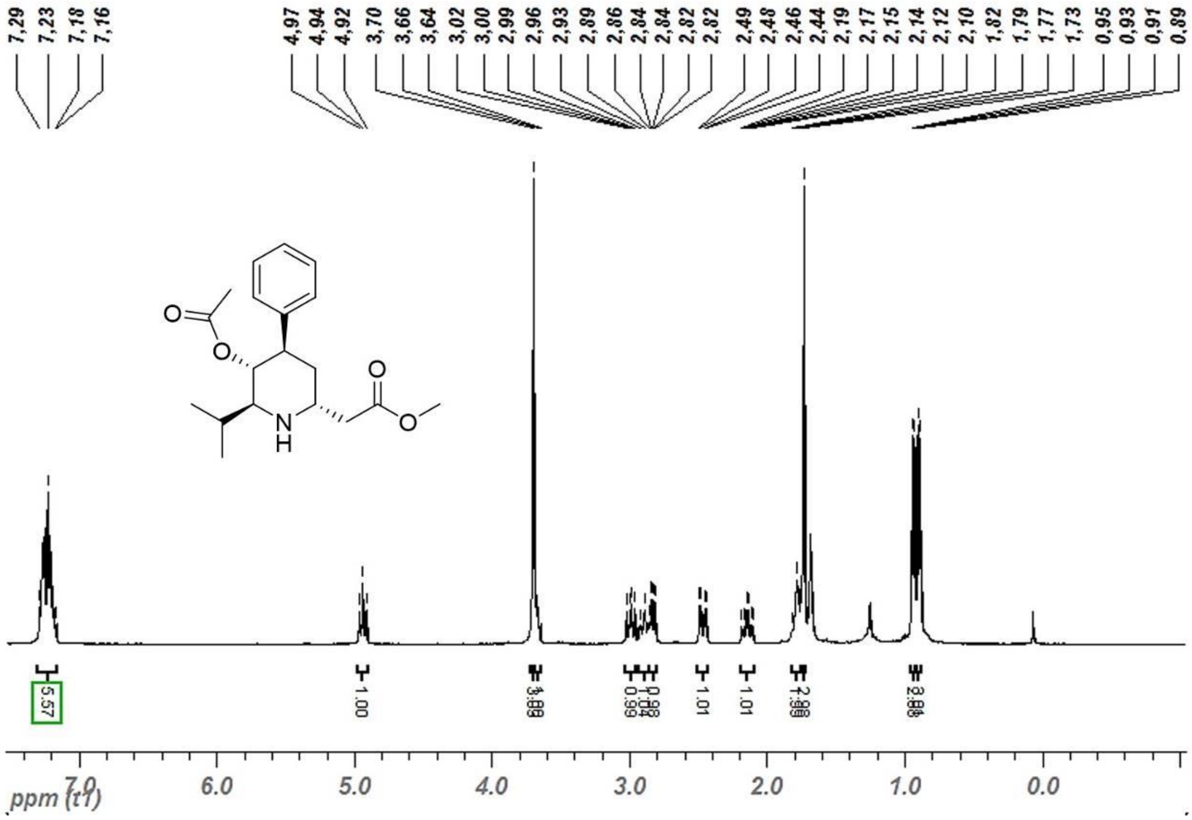

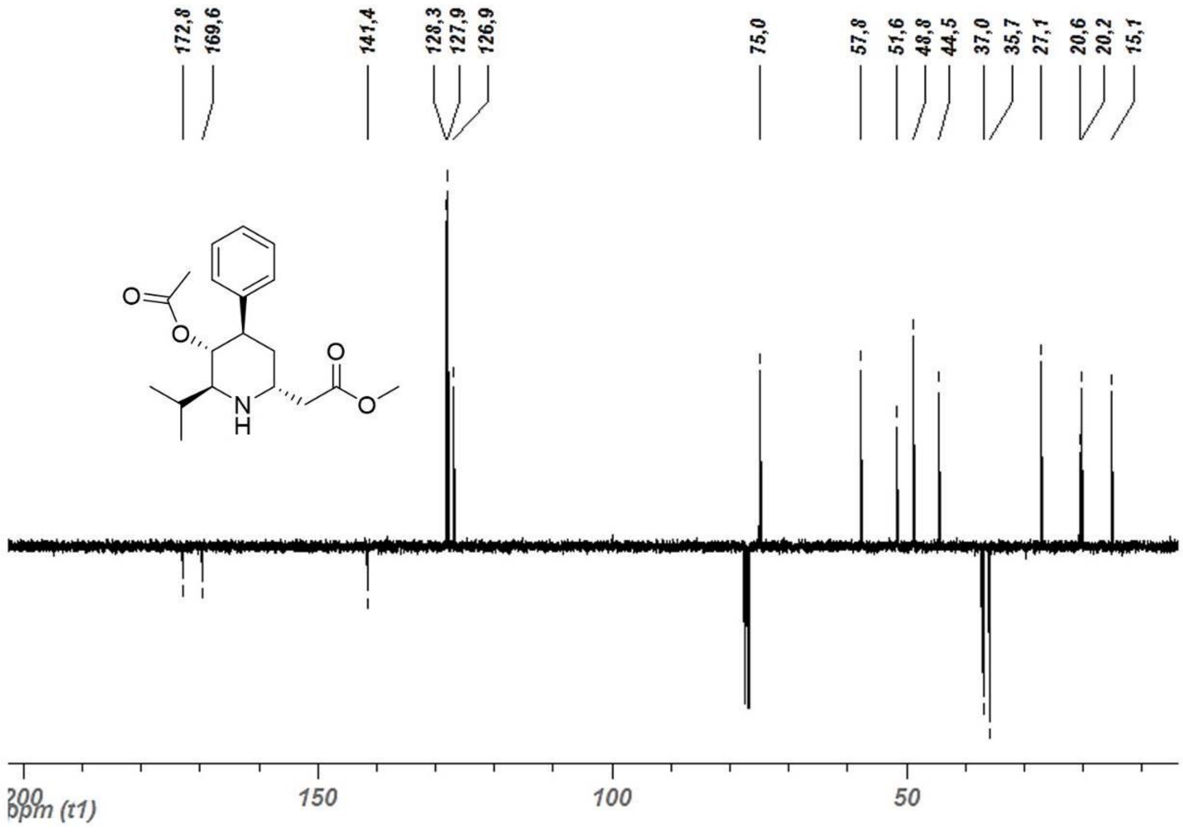

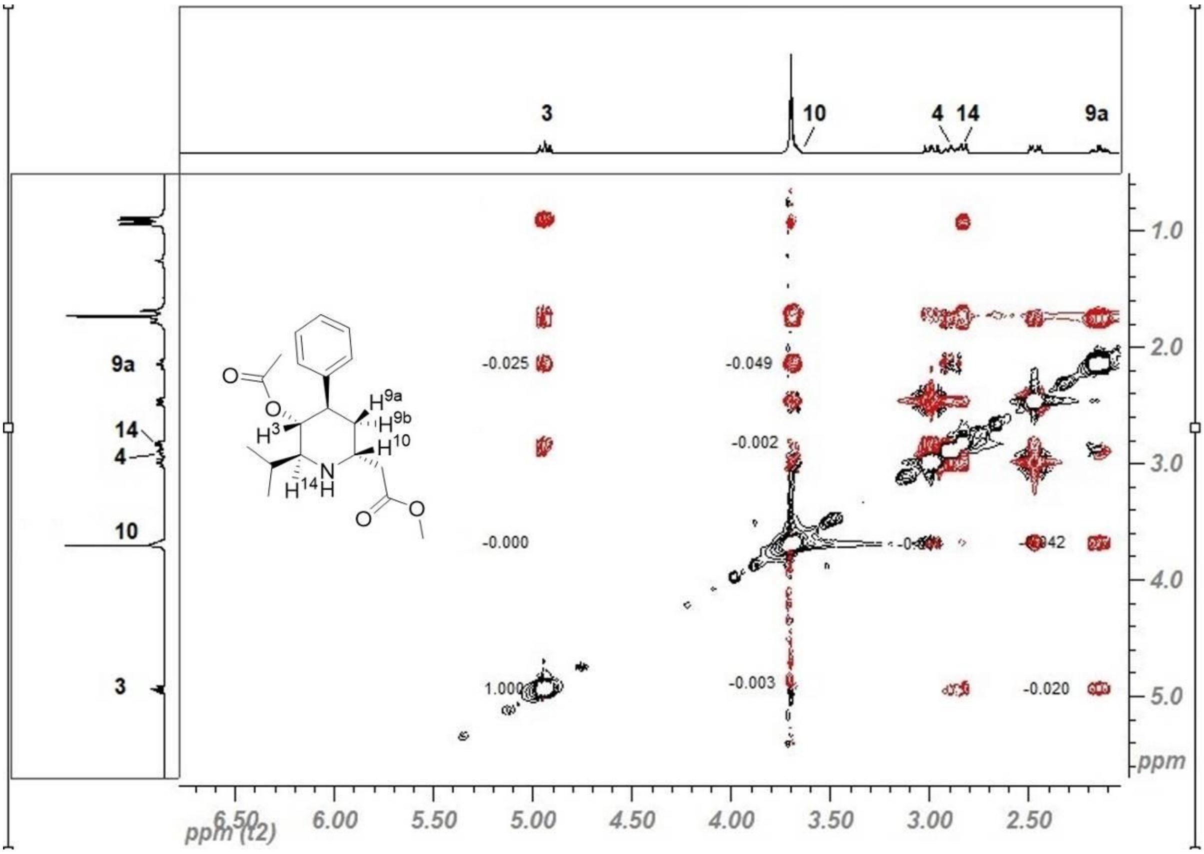
3
10
4
14
9a
-0.025
-0.049
-0.002
-0.000
1.000
-0.003
-0.020
1.0
2.0
3.0
4.0
5.0
ppm
6.50
6.00
5.50
5.00
4.50
4.00
3.50
3.00
2.50
ppm (t2)

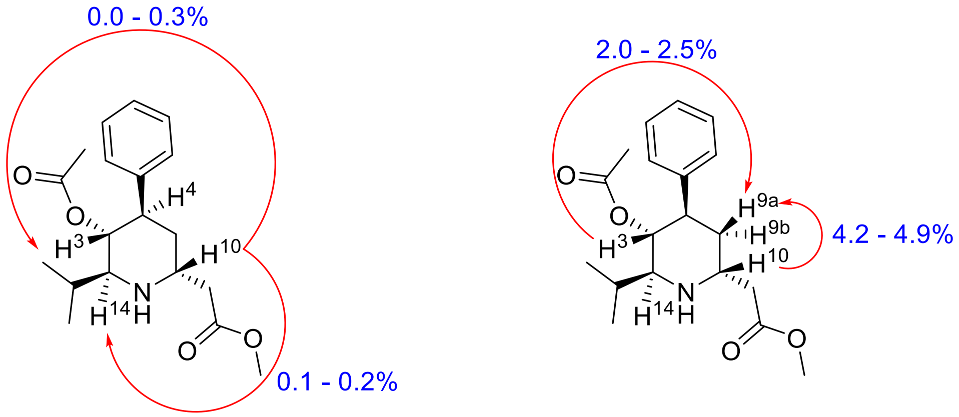
0.0 - 0.3%
0.1 - 0.2%
2.0 - 2.5%
4.2 - 4.9%

LCMS [(2*S*)-5a and **(2*R*)-5a]**

Column: Phenomenex Luna 3u C18(2) RP (50 x 4.6 mm, 100 Å, 5 μm)

Eluent: MeCN/H_2O 50:50, 0.6 mL/min

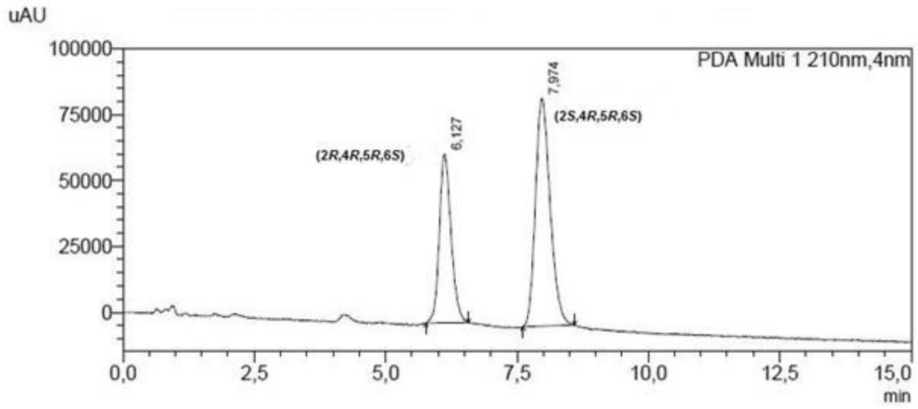

PDA Ch1 210nm

Peak#	Ret. Time	Area	Area%
1	6,127	994034	36,985
2	7,974	1693636	63,015
Total		2687670	100,000

Ethyl (*S*,*E*)-4-[(ethoxycarbonyl)oxy]pent-2-enoate (7a)

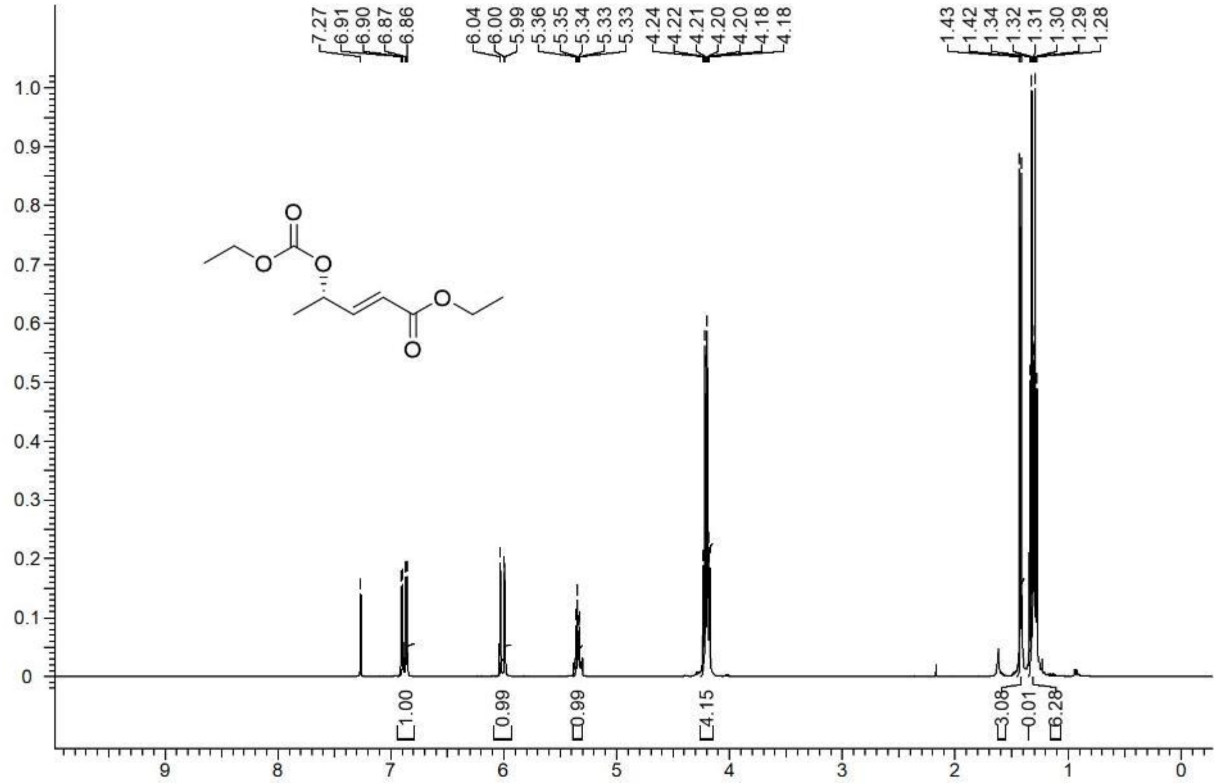

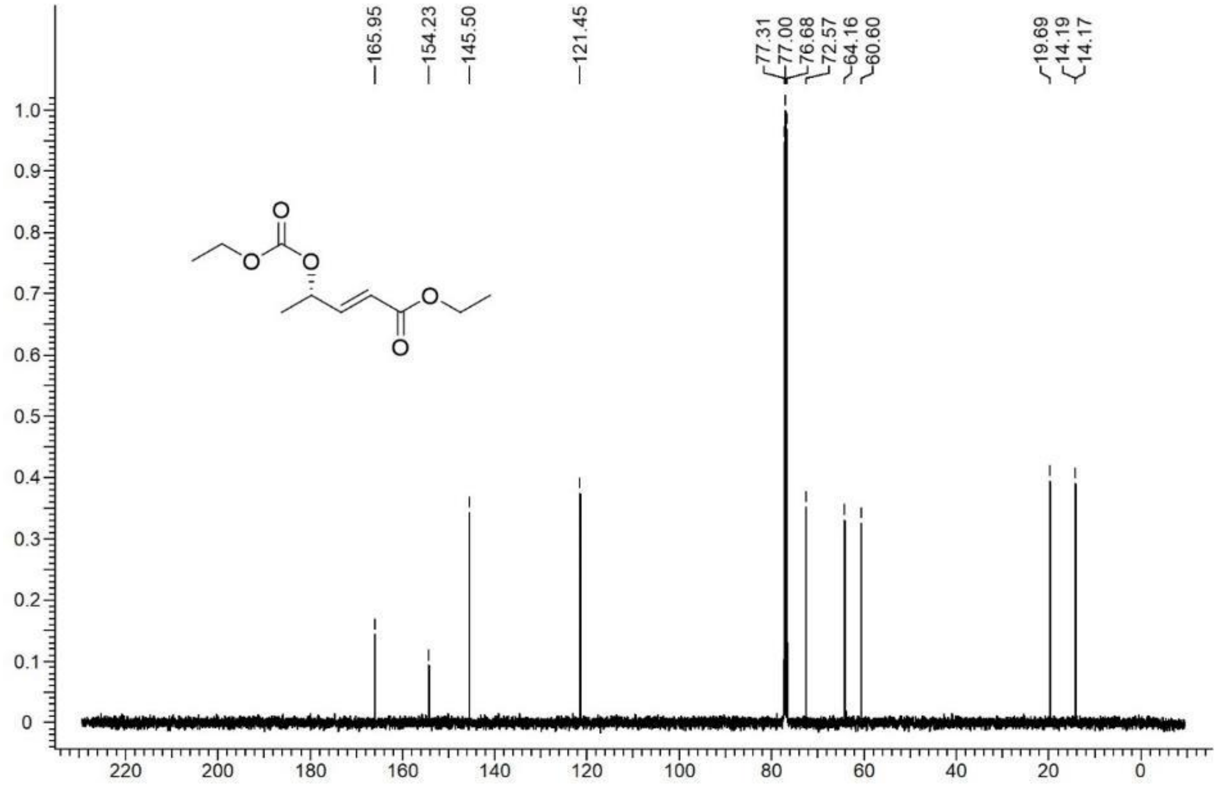

GC FID (7a)
Column: Varian CP-Chirasil-Dex CB (25 m x 0.25 mm, 0.25 µm ID)
Parameters: 60 °C (5 min) – 200°C (5 min), rate 4 °C/min, flow 1.2 mL/min)

(*S*)-**7a** (99% ee)

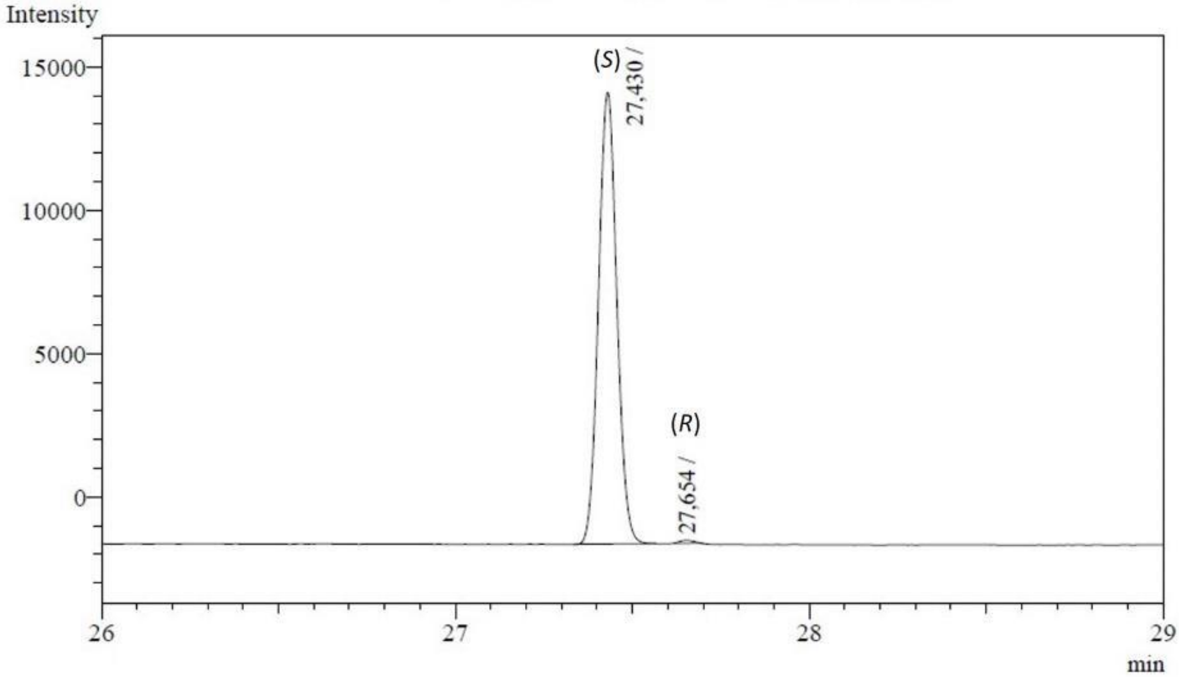

Peak#	Ret.Time	Area	Height	Conc.	Unit	Mark	ID#	Cmpd Name
1	27.430	56851	13526	99.497				
2	27.654	288	100	0.503				
Total		57139	13626					

(*R*)-**7a** (97%ee)

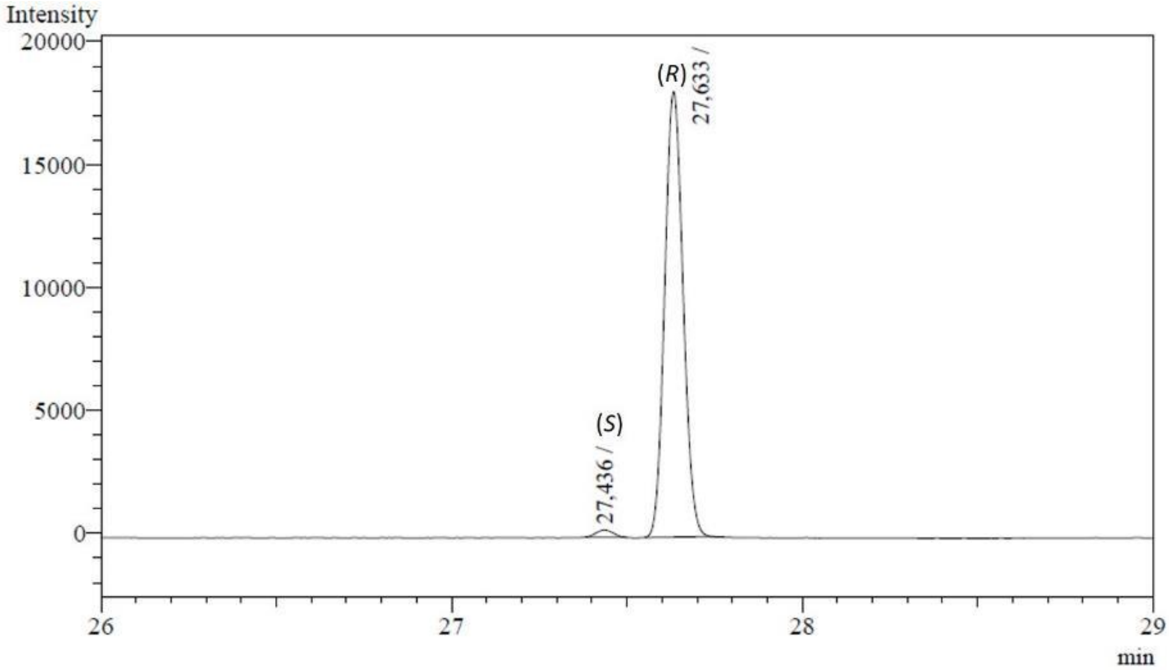

Peak#	Ret.Time	Area	Height	Conc.	Unit	Mark	ID#	Cmpd Name
1	27.436	1000	280	1.494				
2	27.633	65920	16554	98.506				
Total		66920	16834					

Ethyl (*S*,*E*)-4-[(ethoxycarbonyl)oxy]-5-methylhex-2-enoate (7b)

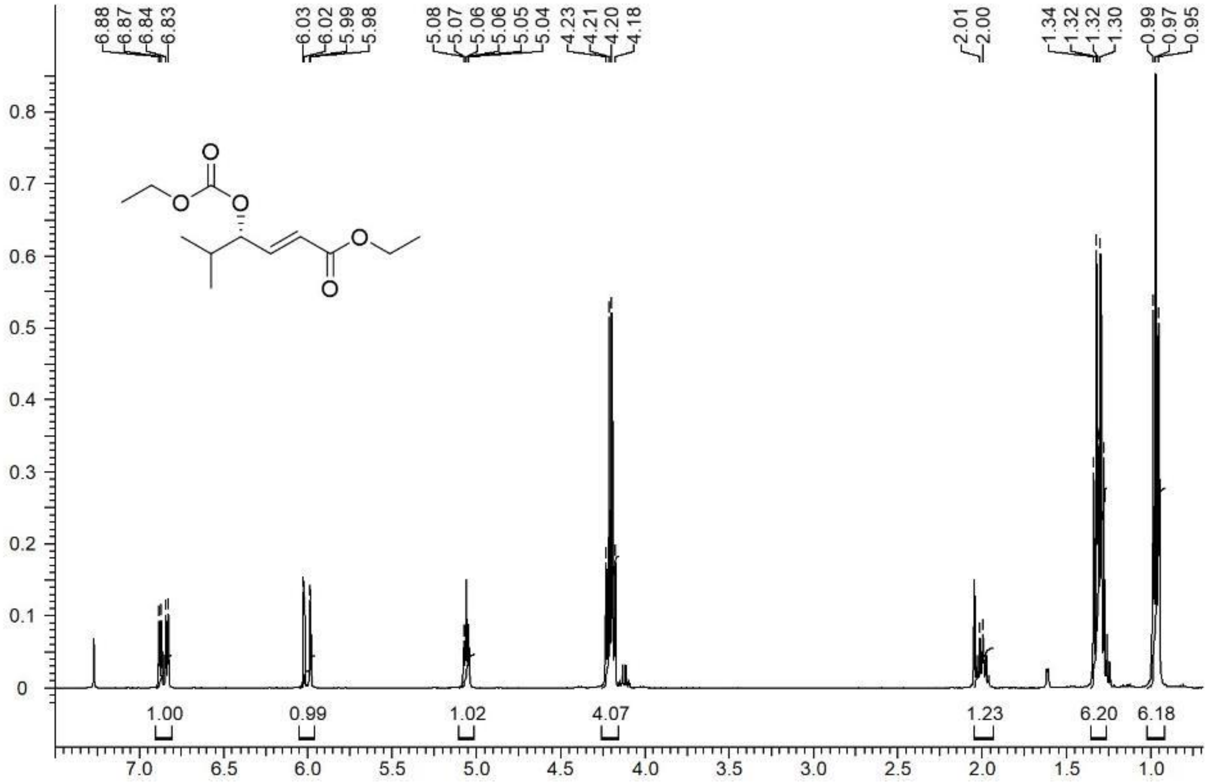

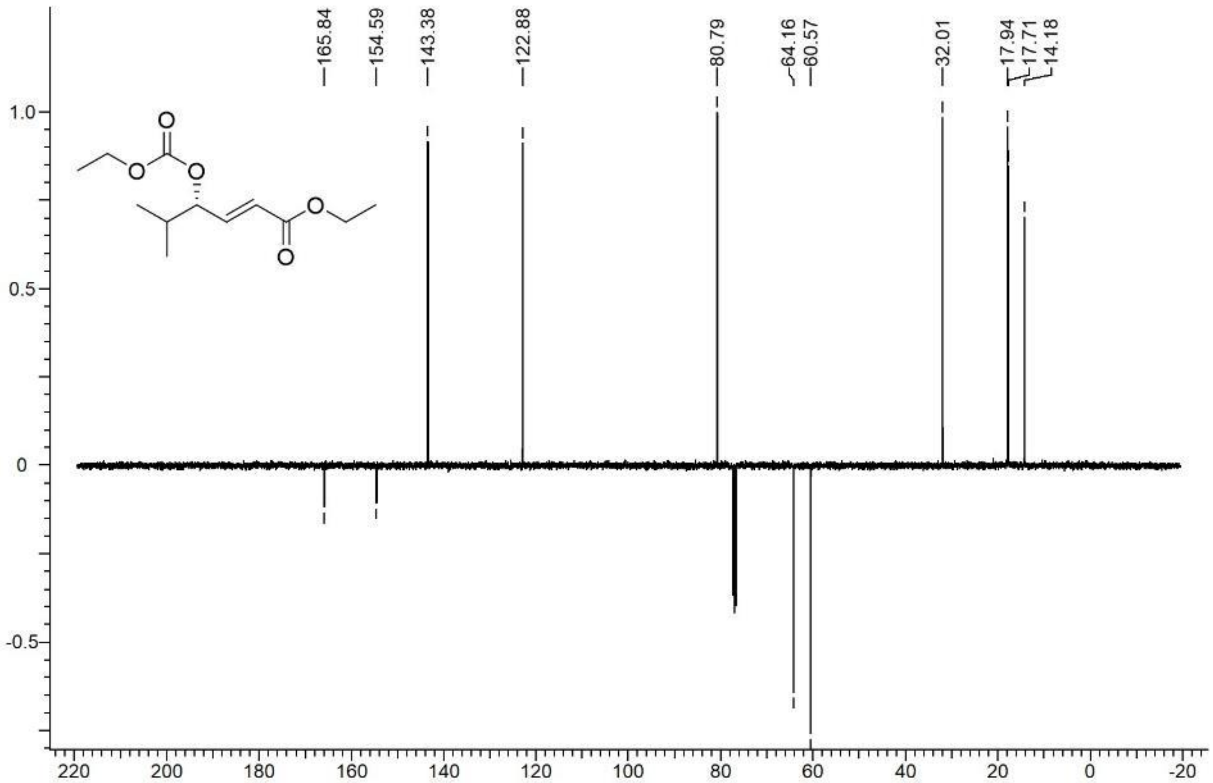

GC FID (7b) (100% ee)
Column: Varian CP-Chirasil-Dex CB (25 m x 0.25 mm, 0.25 µm ID)
Parameters: 80 °C (2 min) – 200 °C (5 min), rate 10 °C/min, flow 1.2 mL/min)

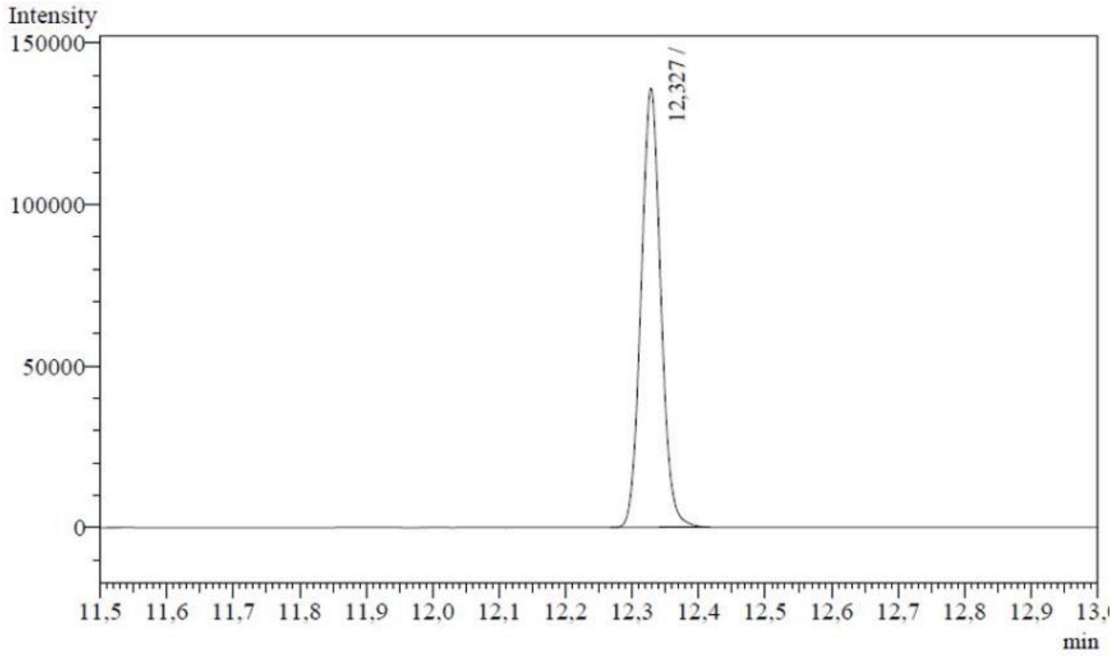

Peak#	Ret.Time	Area	Height	Conc.	Unit	Mark	ID#	Cmpd Name
1	12.327	279547	78984	100.000				
Total		279547	78984					

Ethyl (*R*,*E*)-4-[(*tert*-butoxycarbonyl)oxy]-4-phenylbut-2-enoate (7c)

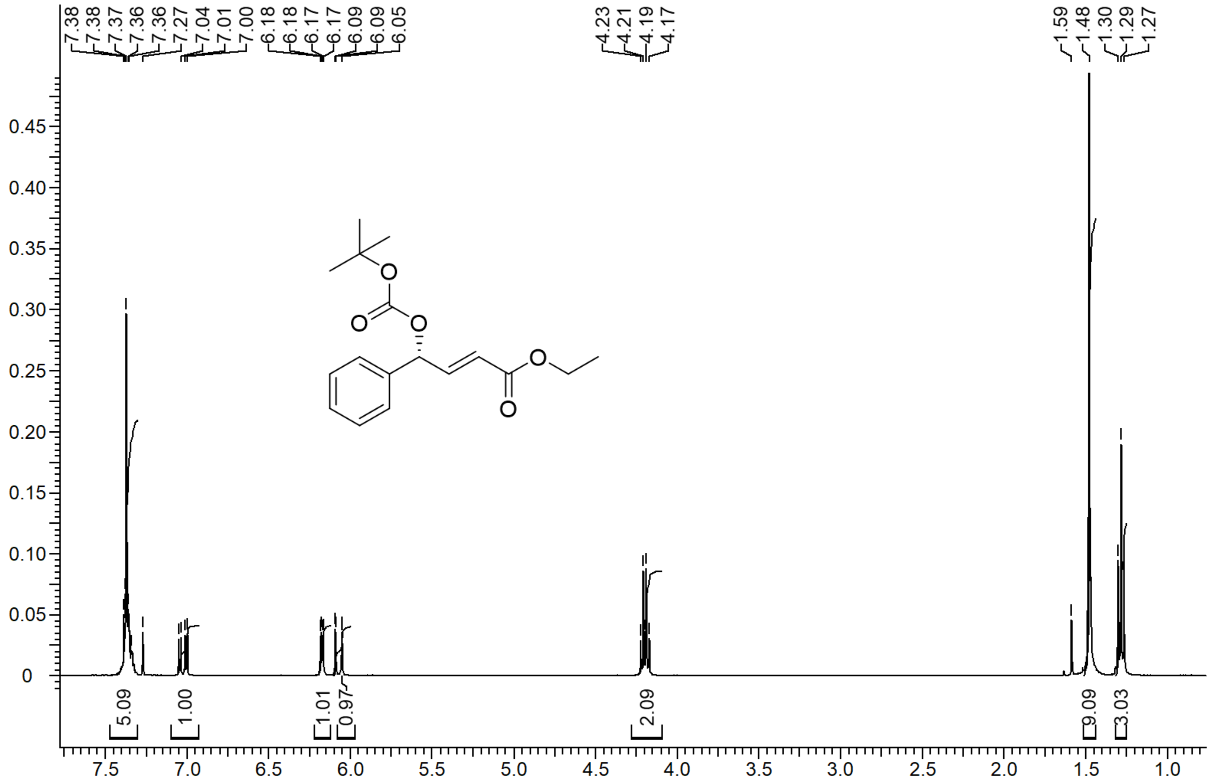

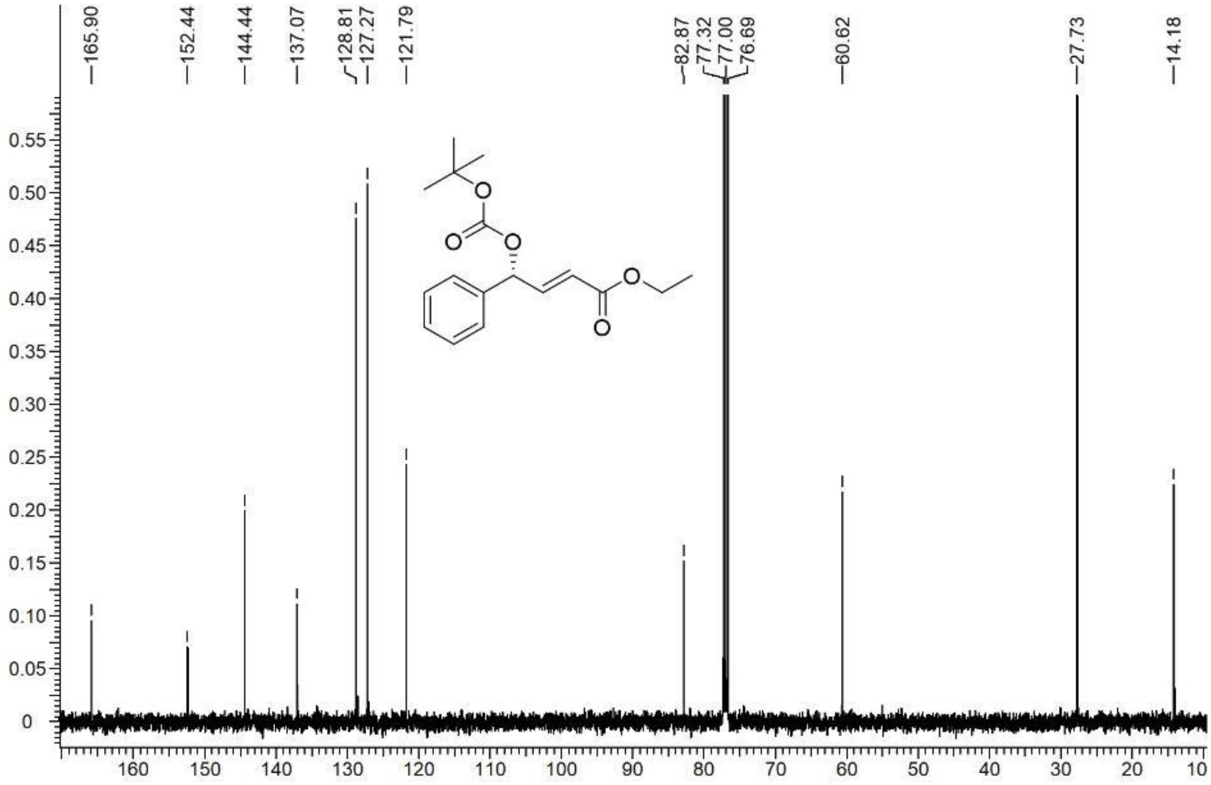

GC FID (7c) (100% ee)
Column: Varian CP-Chirasil-Dex CB (25 m x 0.25 mm, 0.25 µm ID)
Parameters: 60 °C (5 min) – 200°C (5 min), rate 4 °C/min, flow 1.2 mL/min)

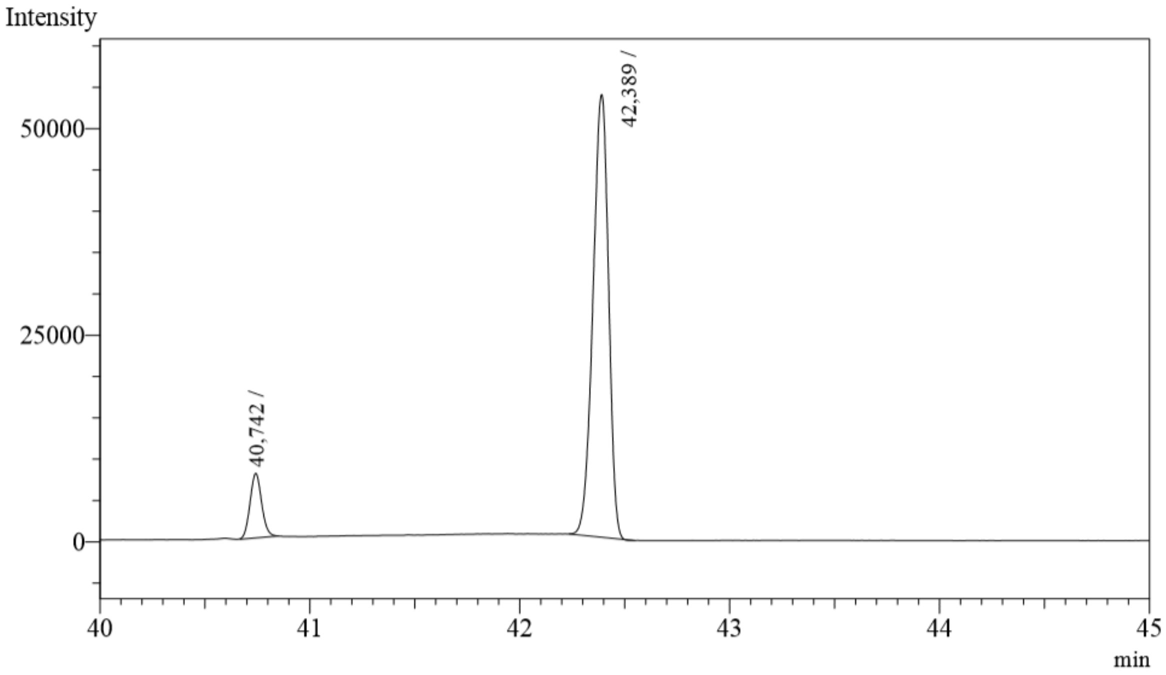

Peak#	Ret.Time	Area	Height	Conc.	Unit	Mark	ID#	Cmpd Name
1	40,742	30042	7667	9,806				
2	42,389	276321	52970	90,194				
Total		306363	60637					

* Peak at 40.74 min is the inseperable starting material

(*S*,*E*)-*tert*-Butyl (5-oxohex-3-en-2-yl) carbonate (8a)

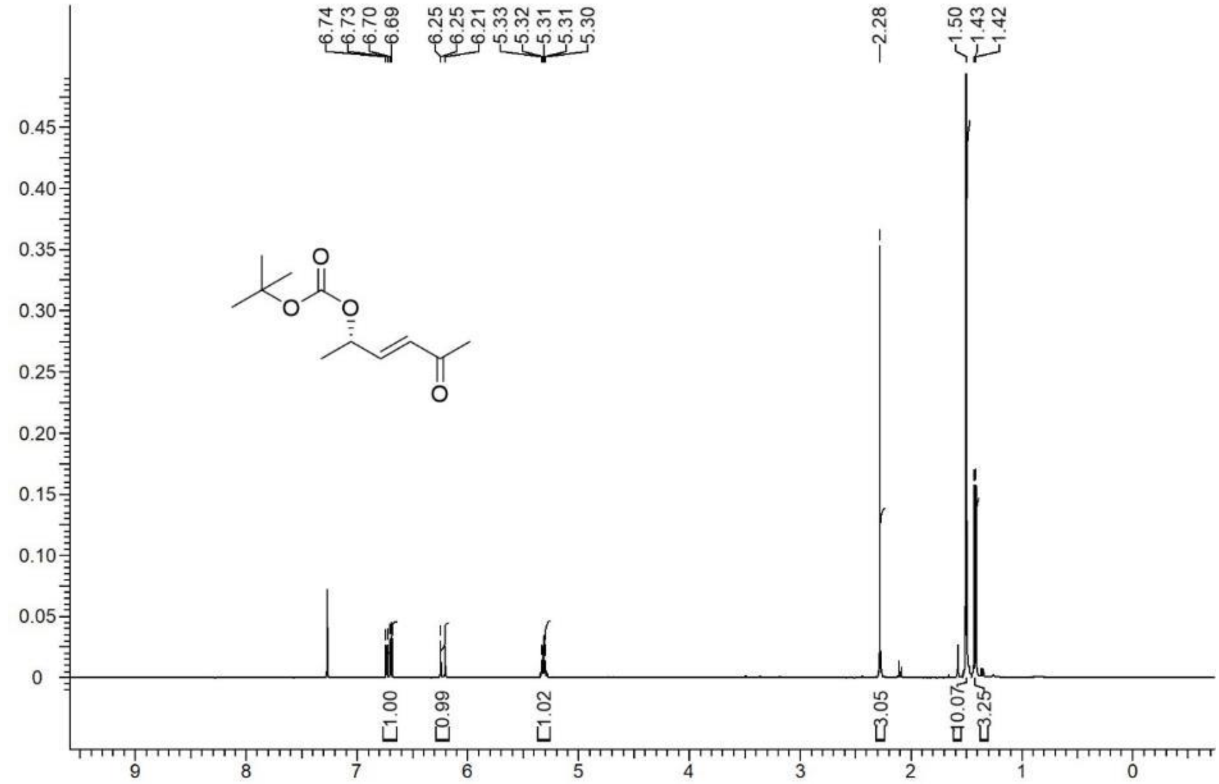

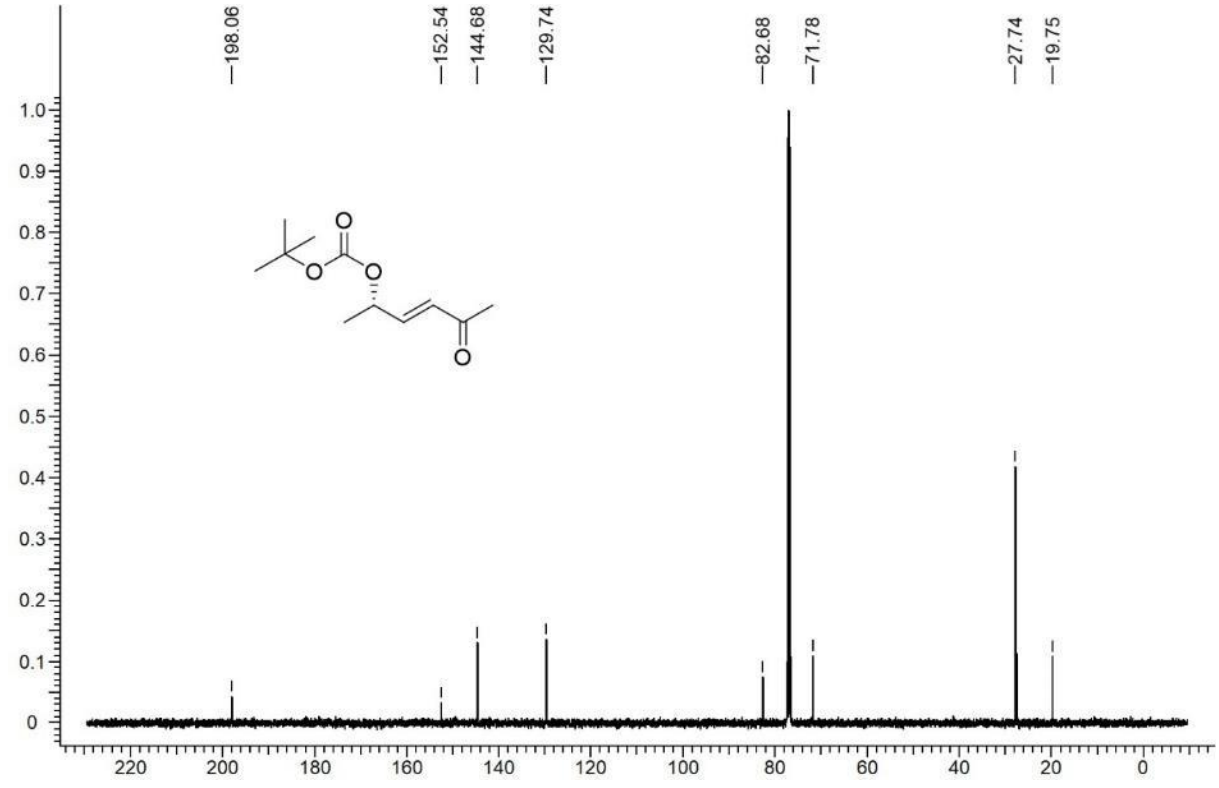

GC FID (8a) (99.7% ee)
Column: Varian CP-Chirasil-Dex CB (25 m x 0.25 mm, 0.25 µm ID)
Parameters: 80 °C (2 min) – 200 °C (5 min), rate 10 °C/min, flow 1.2 mL/min)

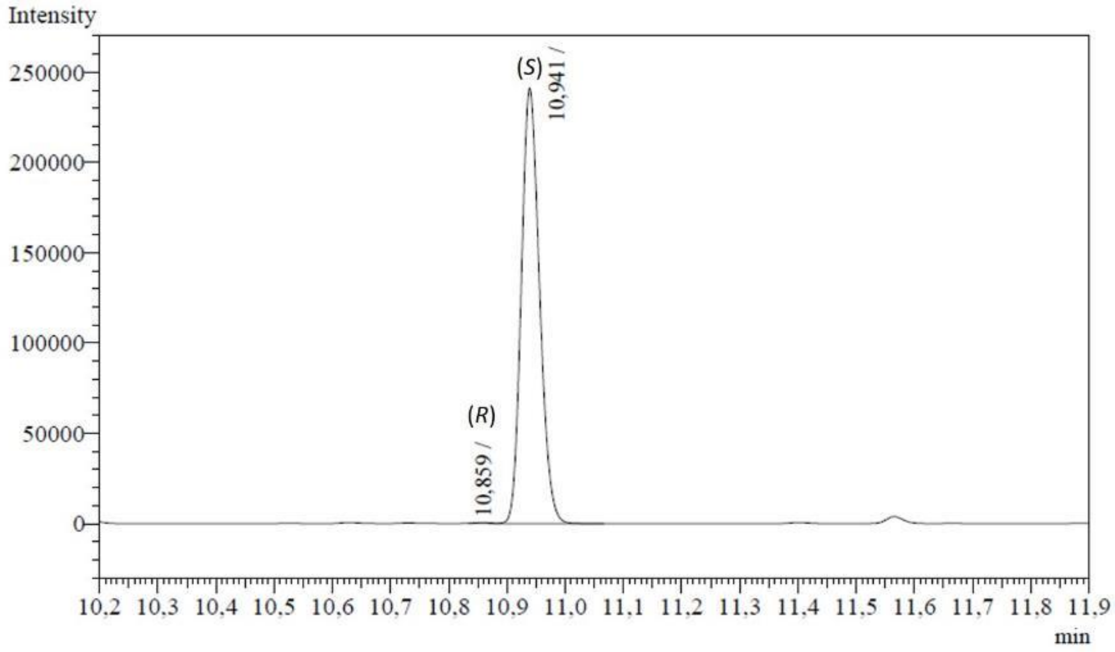

Peak#	Ret.Time	Area	Height	Conc.	Unit	Mark	ID#	Cmpd Name
1	10.859	741	375	0.141				
2	10.941	523765	223392	99.859		V		
Total		524506	223767					

(*S*,*E*)-*tert*-Butyl (4-cyanobut-3-en-2-yl) carbonate (9a)

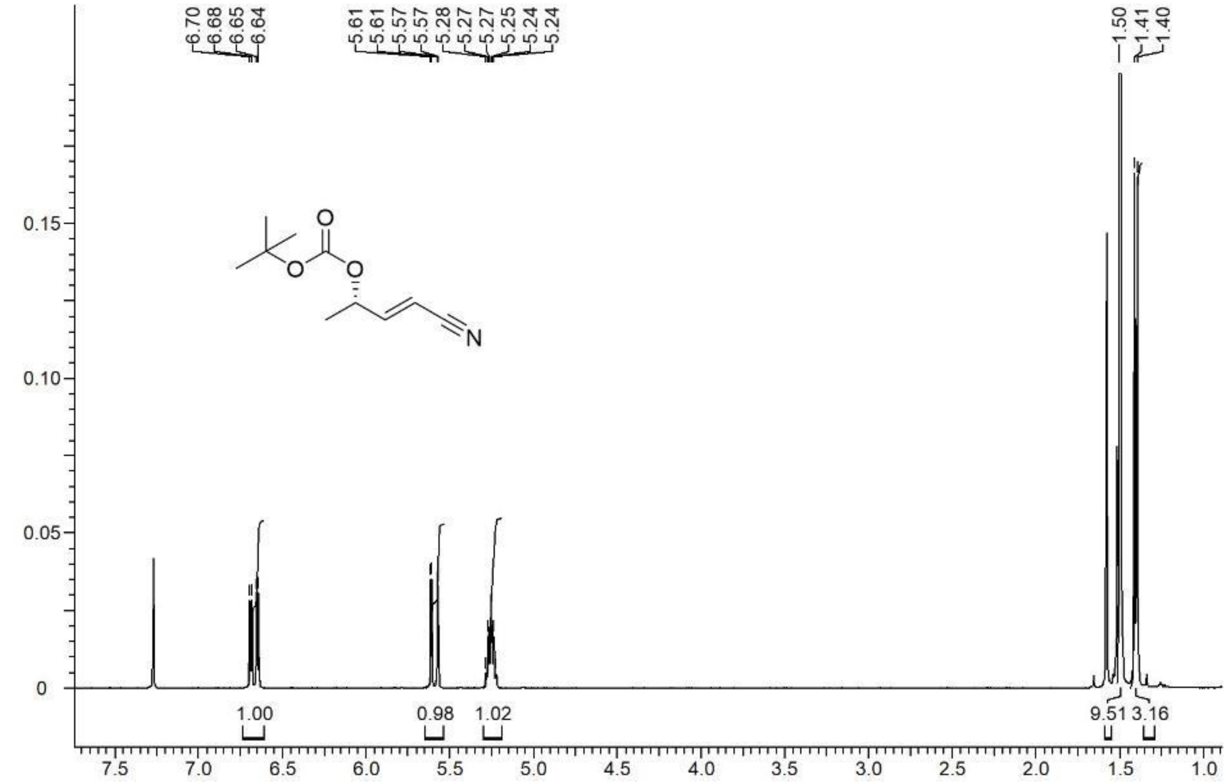

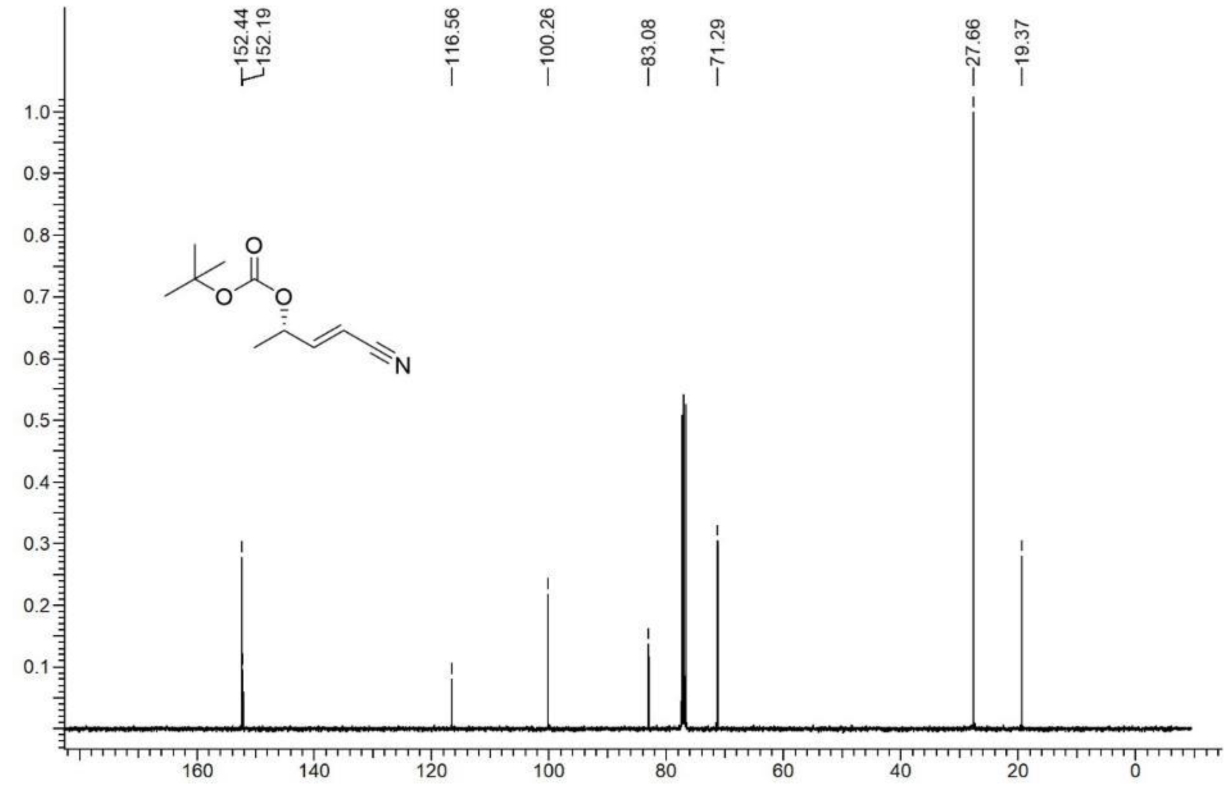

(*S*,*Z*)-*tert*-Butyl (4-cyanobut-3-en-2-yl) carbonate (9a)

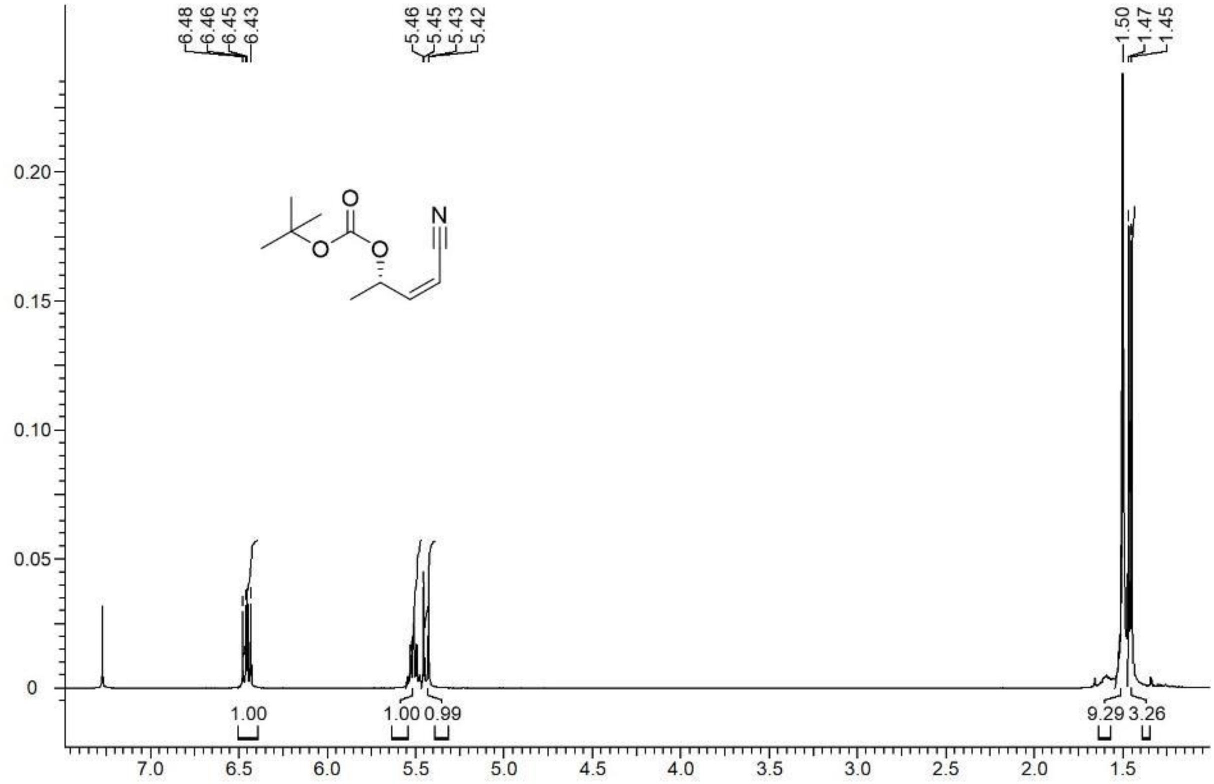

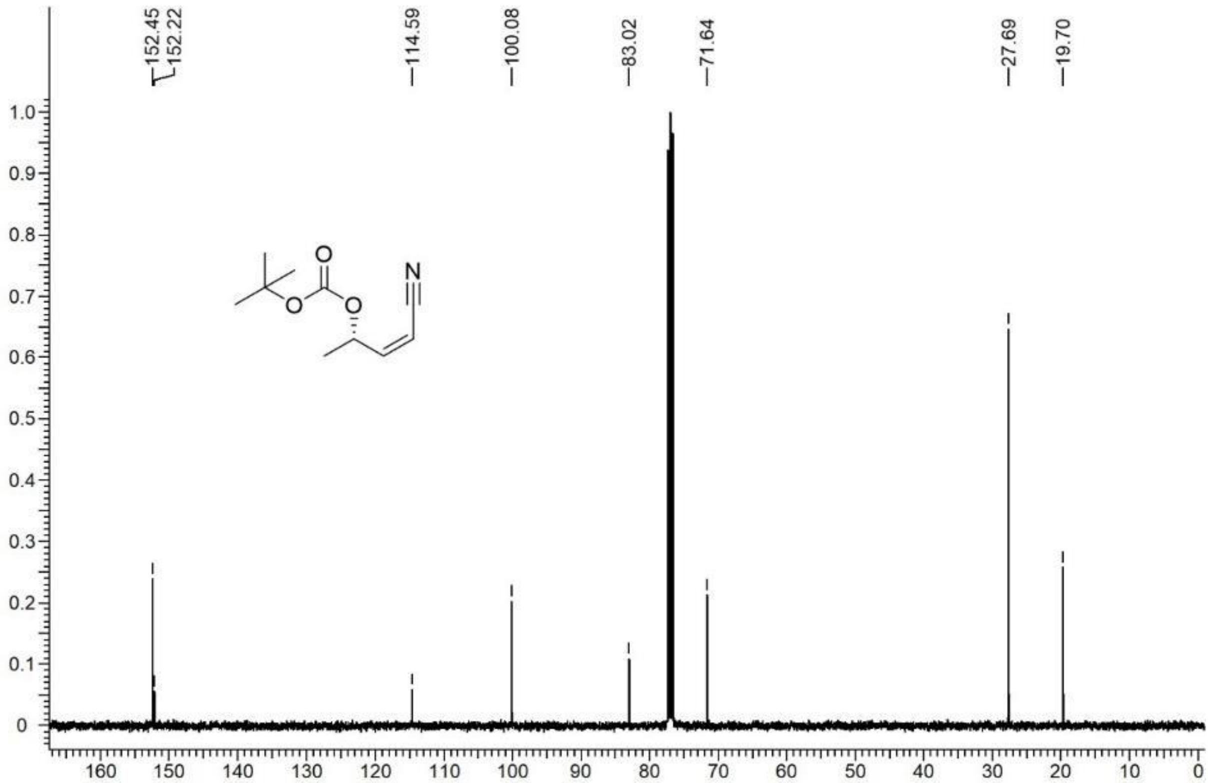

GC FID (9a)
Column: Varian CP-Chirasil-Dex CB (25 m x 0.25 mm, 0.25 µm ID)
Parameters: 80 °C (2 min) – 200 °C (5 min), rate 10 °C/min, flow 1.2 mL/min)

(*S*,*E*)-(**9a**) (100% ee)

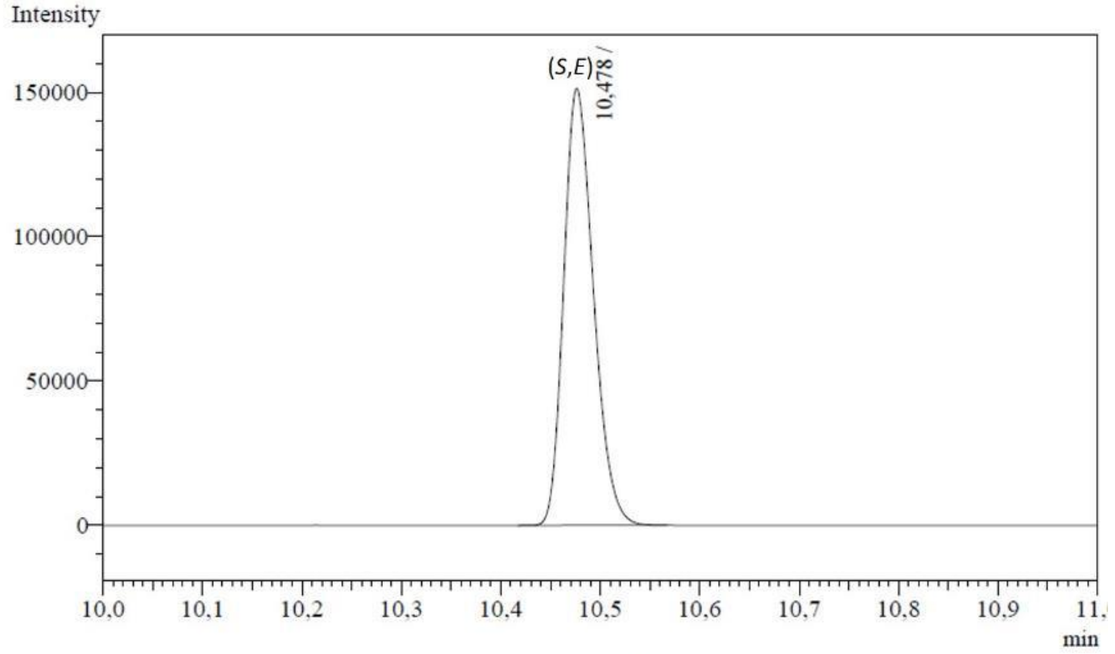

Peak#	Ret.Time	Area	Height	Conc.	Unit	Mark	ID#	Cmpd Name
1	10,478	314139	85247	100,000				
Total		314139	85247					

(*S*,*Z*)-(**9a**) (99.9% ee)

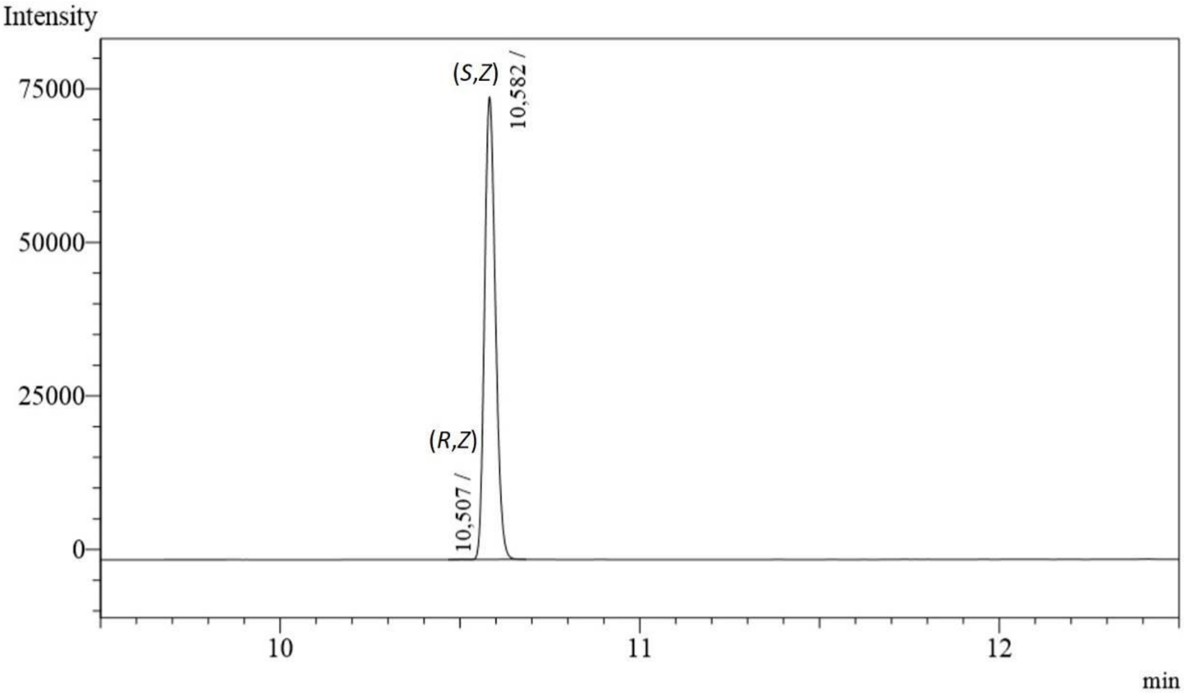

Peak#	Ret.Time	Area	Height	Conc.	Unit	Mark	ID#	Cmpd Name
1	10,507	45	23	0,028				
2	10,582	158480	56746	99,972				
Total		158525	56769					

Ethyl (4*S*,5*R*,7*S*,*E*)-7-[(*tert*-butoxycarbonyl)amino]-4-methyl-6-oxo-5,8-diphenyloct-2-enoate (13a)

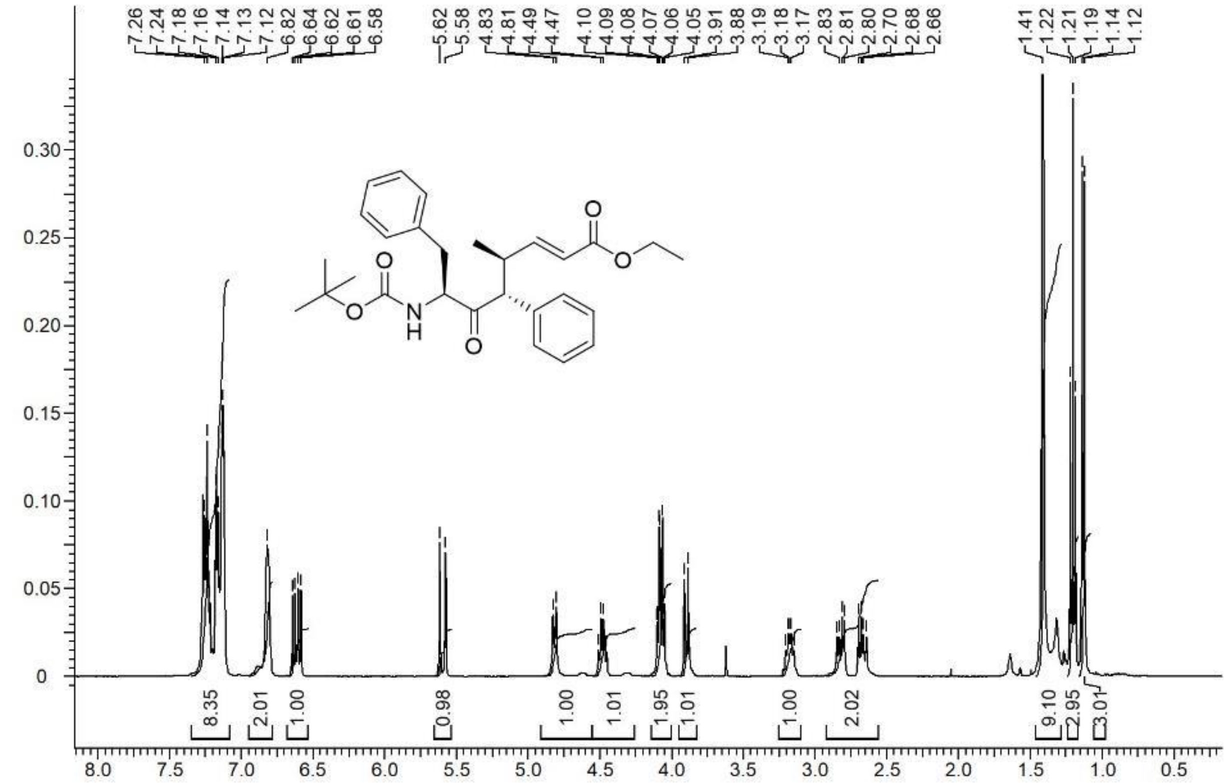

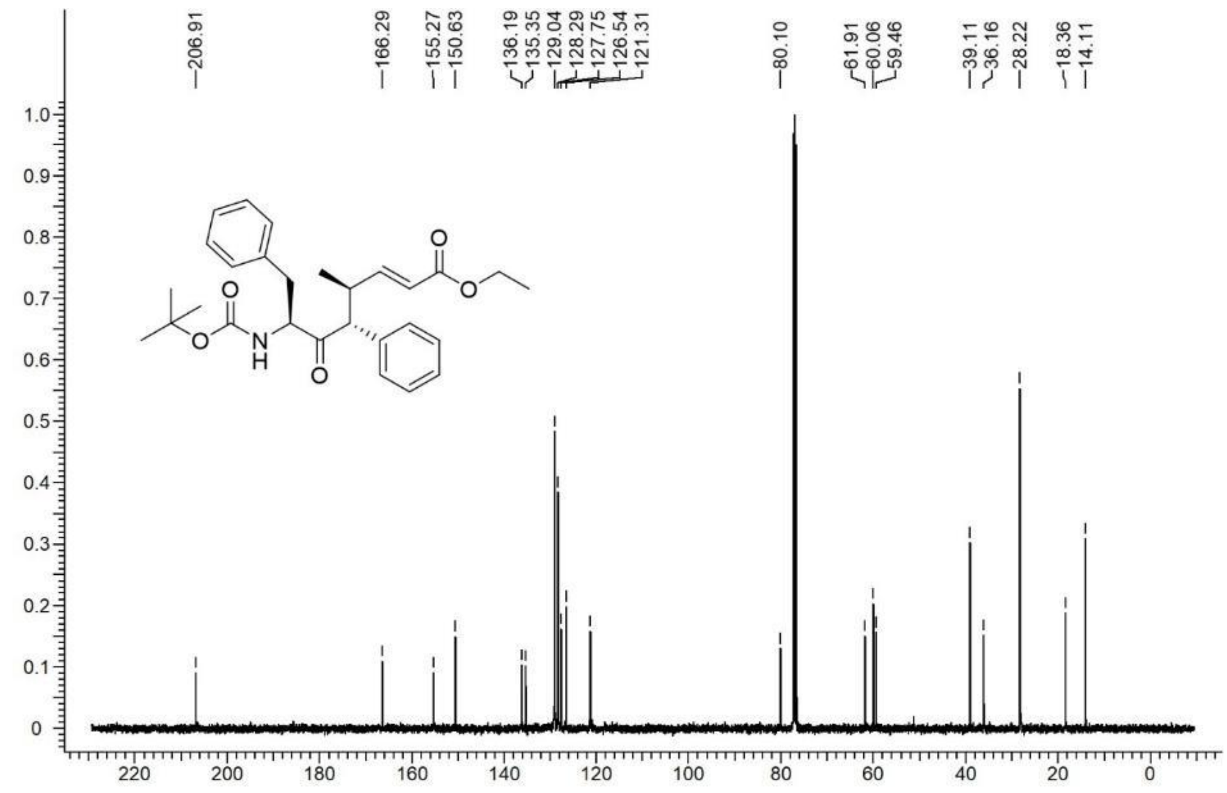

HPLC [crude (4*S*,5*R*)-13a]
Table 1, entry 1
Column: Reprosil 100 Chiral-NR 8 µm
Eluent: Hexane/iPrOH 90:10, 1.5 ml/min, 20°C, 210 nm

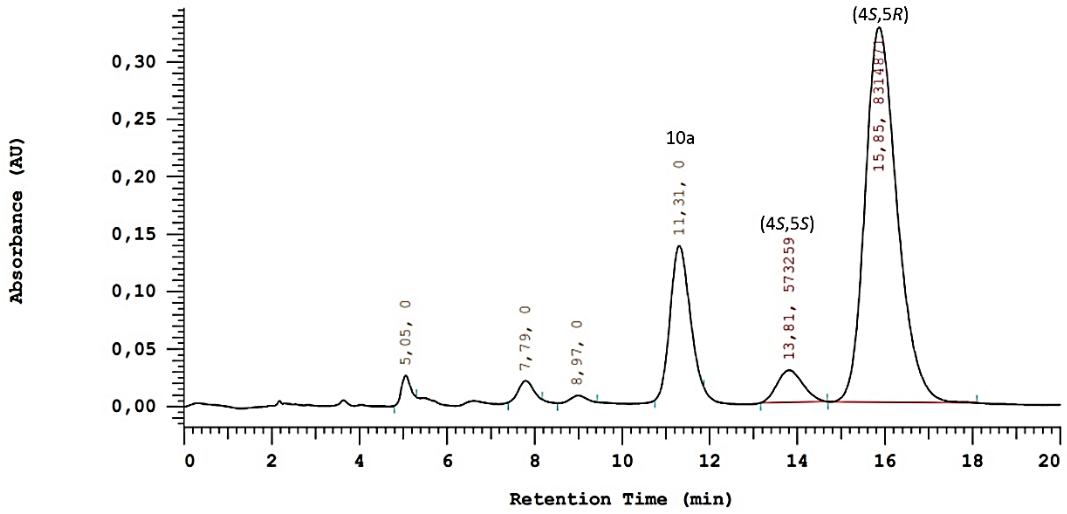

No.	RT	Area	Area %
1	5,05	0	0,000
2	7,79	0	0,000
3	8,97	0	0,000
4	11,31	0	0,000
5	13,81	573259	6,450
6	15,85	8314871	93,550
		8888130	100,000

Ethyl (4*R*,5*R*,7*S*,*E*)-7-[(*tert*-butoxycarbonyl)amino]-4-methyl-6-oxo-5,8-diphenyloct-2-enoate (13a)

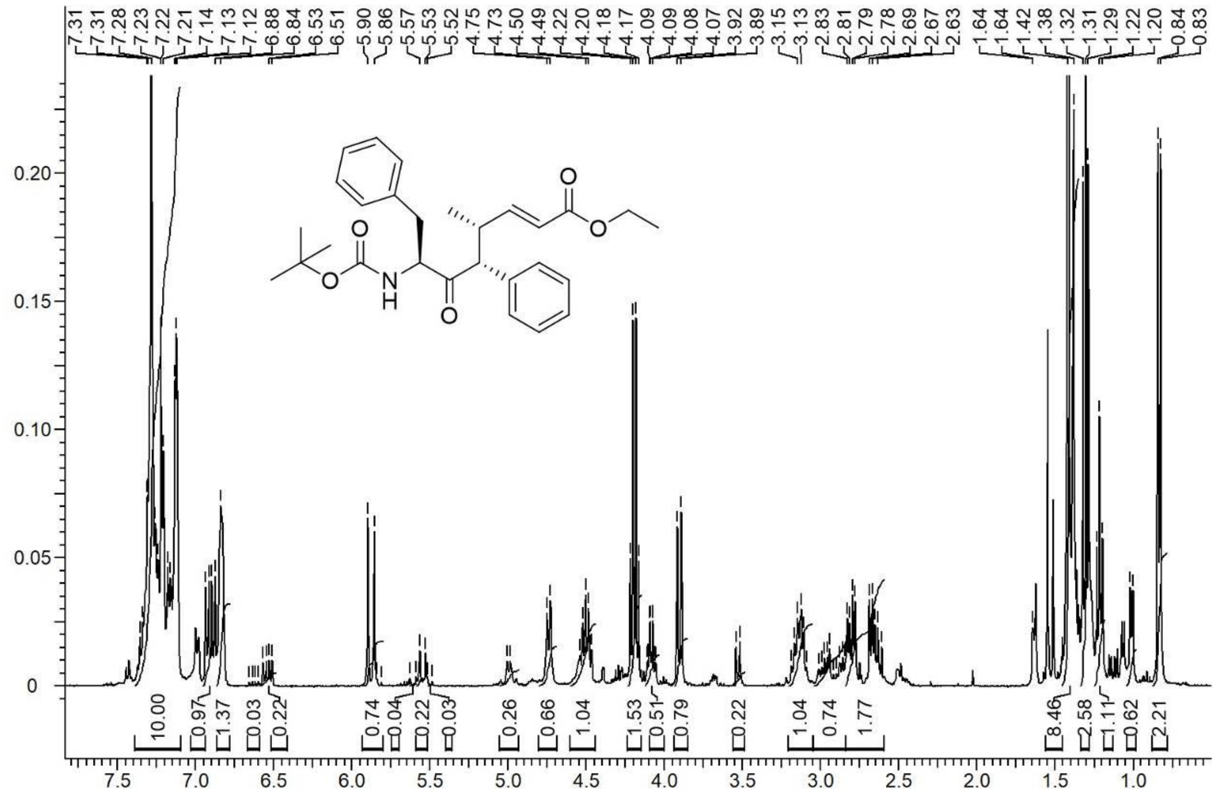

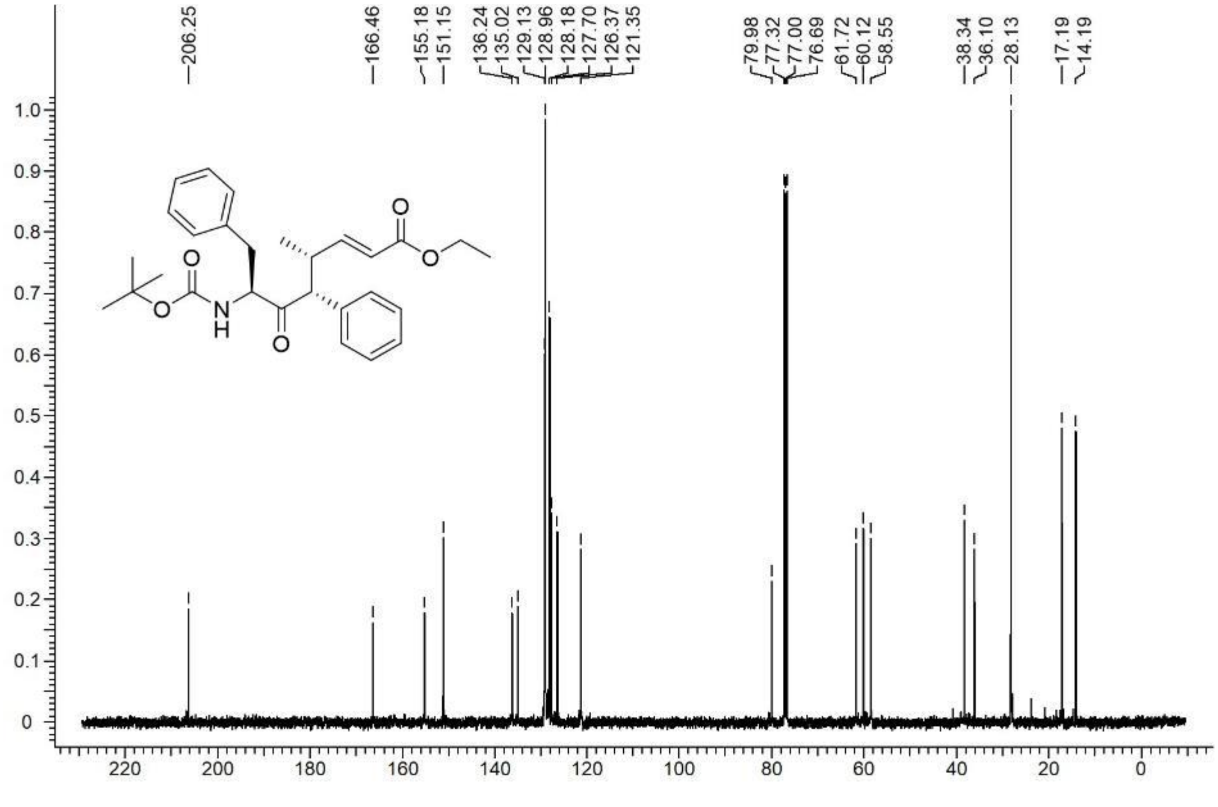

HPLC [(4*R*,5*R*)-13a] – Table 1, entry 3
Column: Reprosil 100 Chiral-NR 8 µm
Eluent: Hexane/iPrOH 90:10, 1.5 ml/min, 20°C, 210 nm

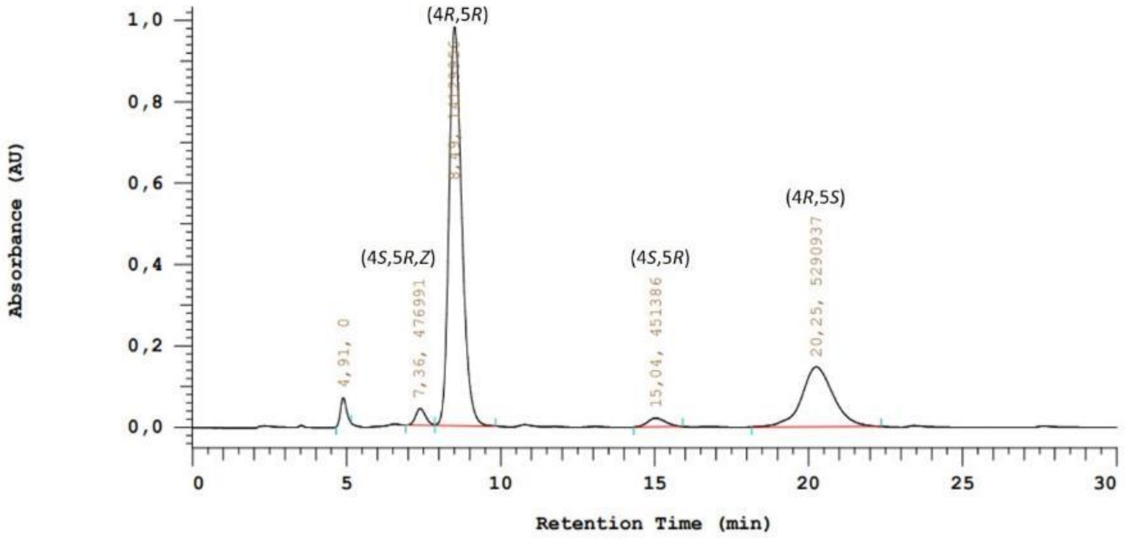

No.	RT	Area	Area %
1	4,91	0	0,000
2	7,36	476991	2,344
3	8,49	14129956	69,437
4	15,04	451386	2,218
5	20,25	5290937	26,001
		20349270	100,000

Ethyl (4*S*,5*S*,7*S*,*E*)-7-[(*tert*-butoxycarbonyl)amino]-4,5-dimethyl-6-oxo-8-phenyloct-2-enoate (13b)

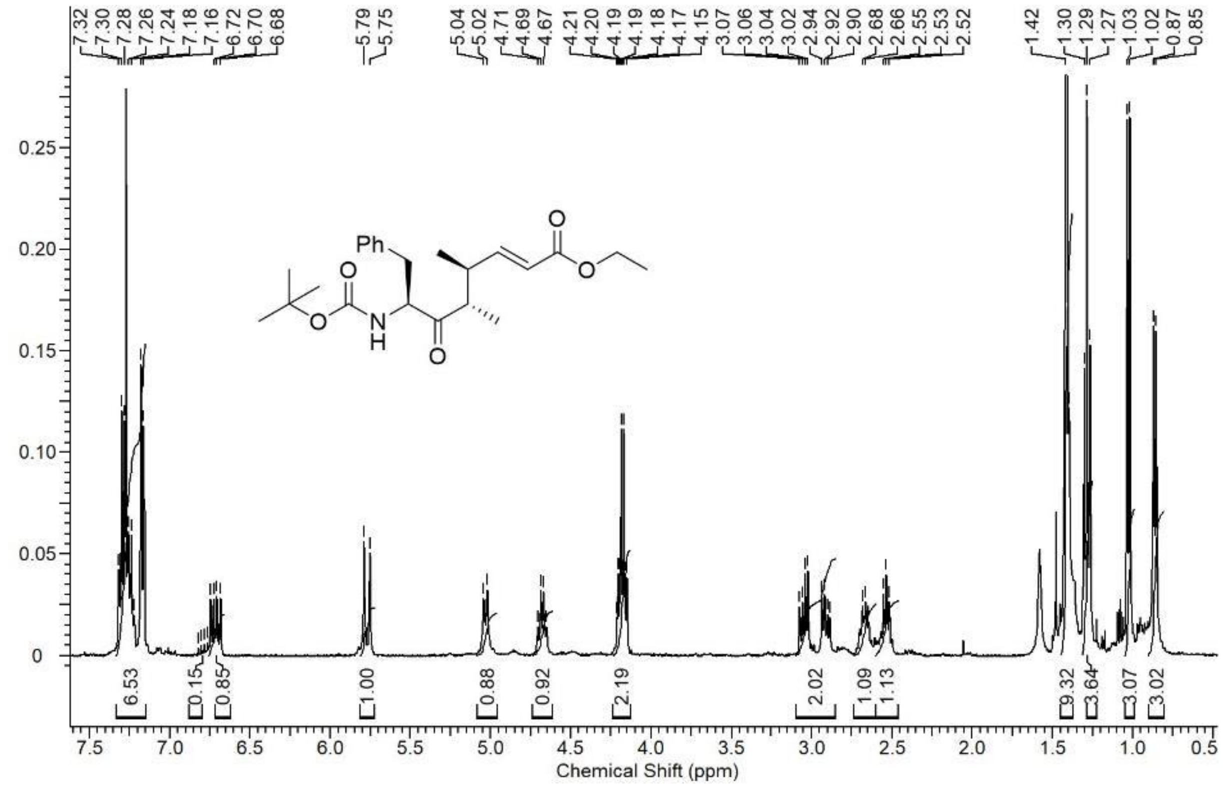

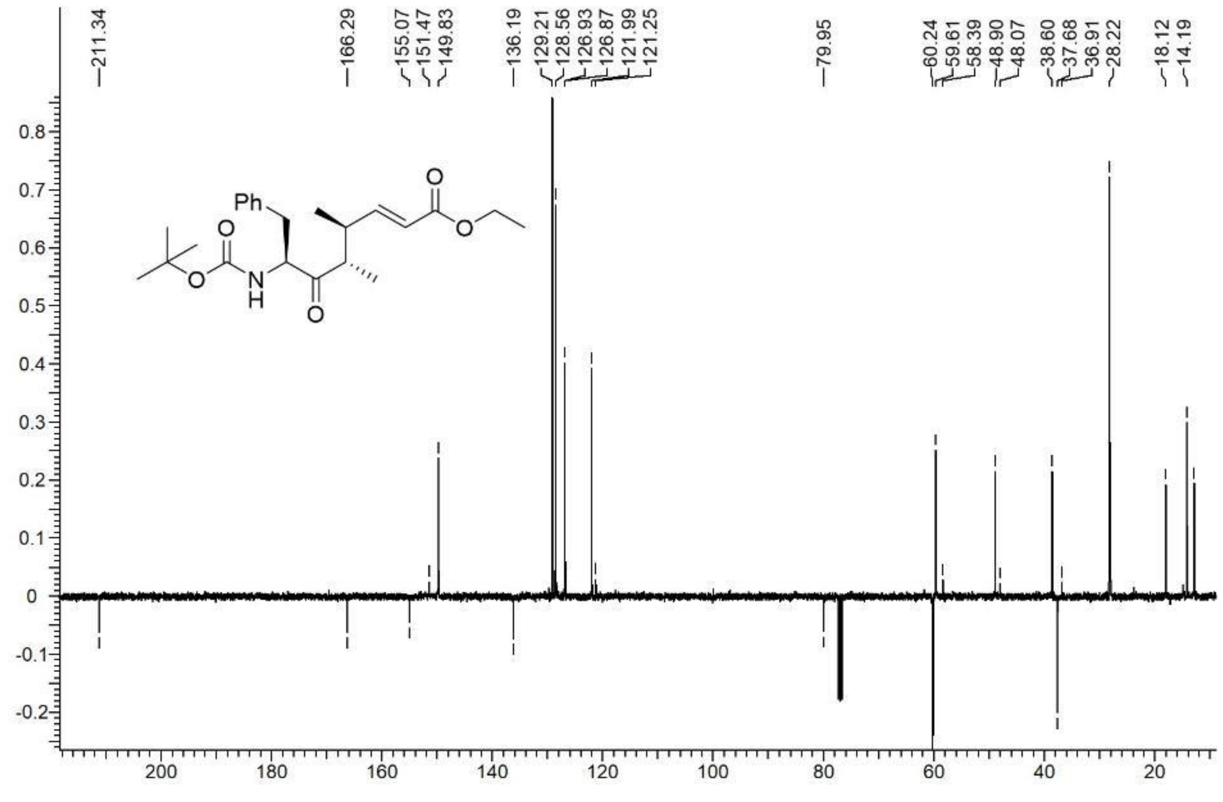

HPLC [(4*S*,5*S*,7*S*,*E*)-13b] – Table 1, entry 13
Column: Reprosil 100 Chiral-NR 8 µm
Eluent: Hexane/iPrOH 95:5, 1.5 ml/min, 20 °C, 210 nm

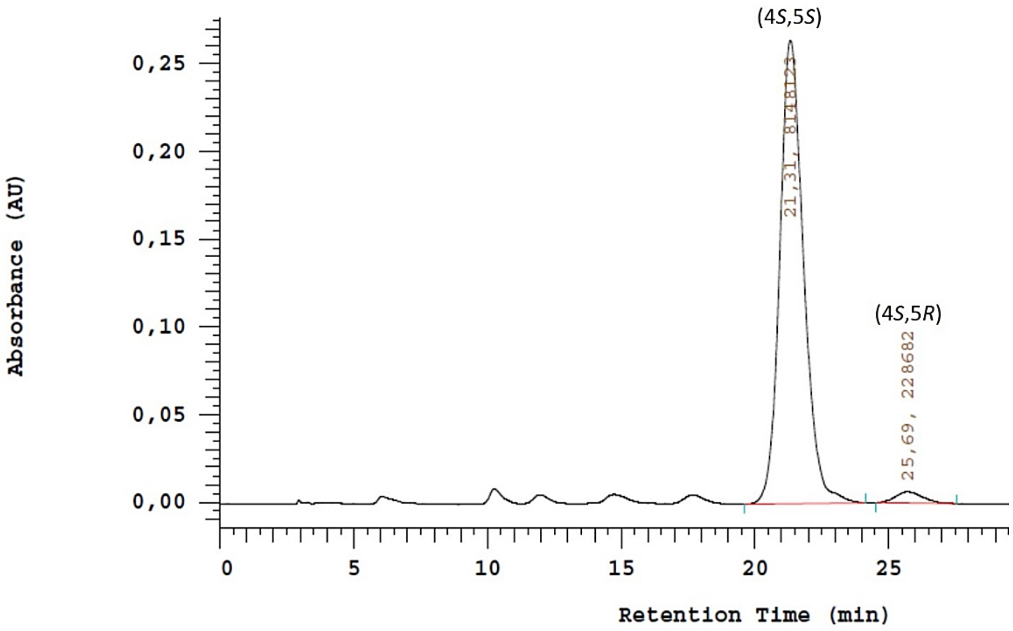

No.	RT	Area	Area %
1	21,31	8148123	97,270
2	25,69	228682	2,730
		8376805	100,000

Ethyl (4*R*,5*S*,7*S*,*E*)-7-[(*tert*-butoxycarbonyl)amino]-4,5-dimethyl-6-oxo-8-phenyloct-2-enoate (13b)

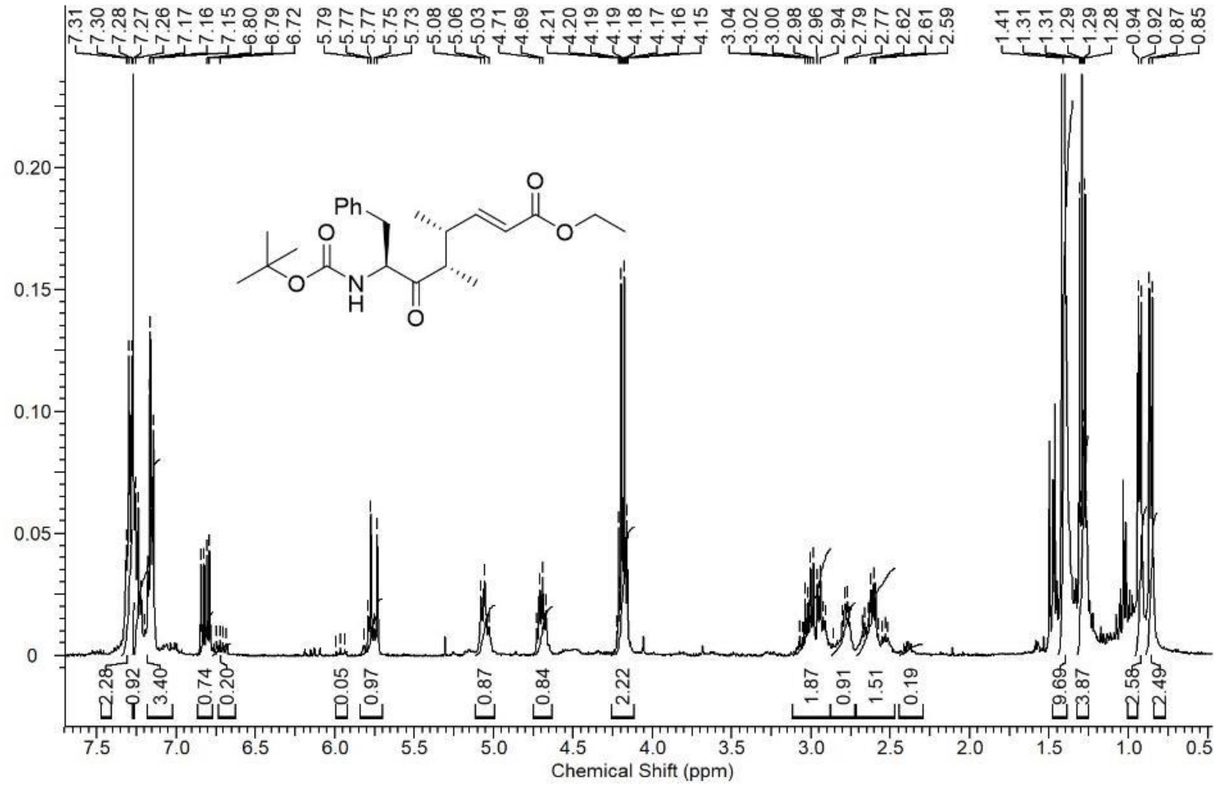

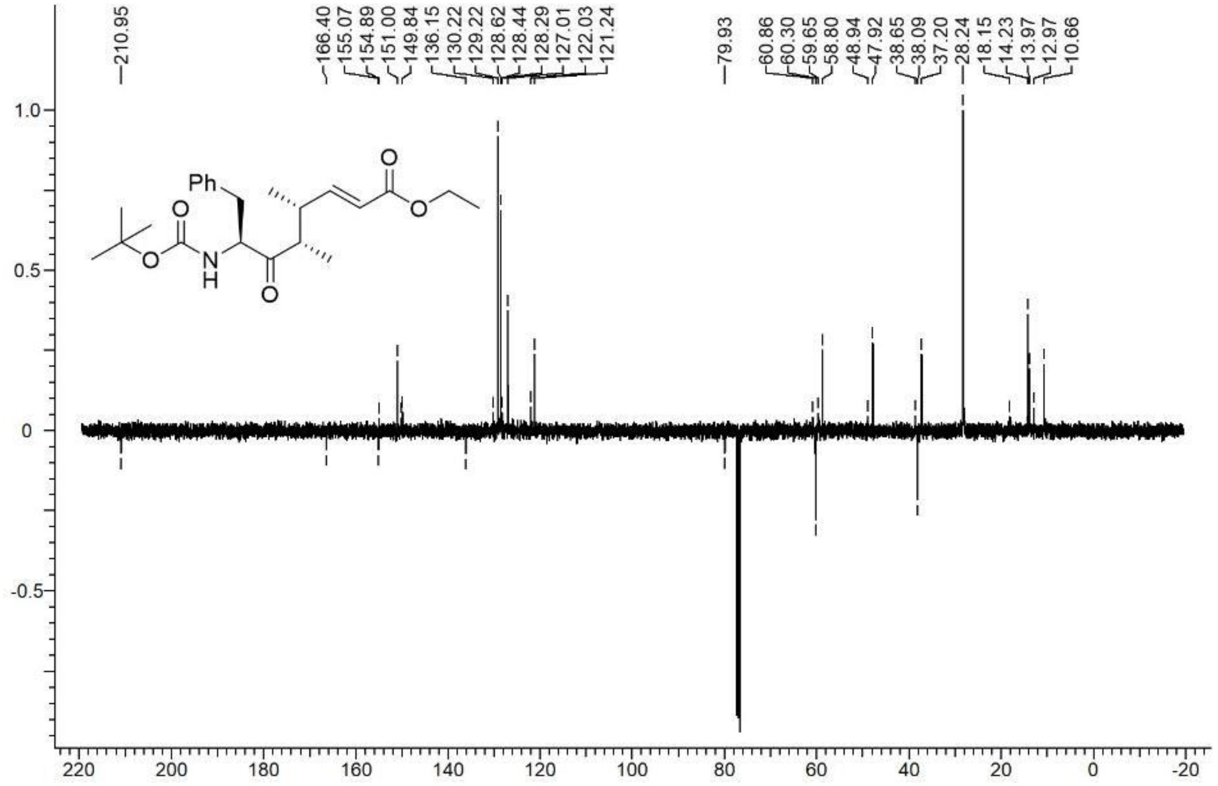

HPLC [(4*R*,5*S*)-13b] – Table 1, entry 14
Column: Reprosil 100 Chiral-NR 8 µm
Eluent: Hexane/iPrOH 95:5, 1.0 ml/min, 20 °C, 210 nm

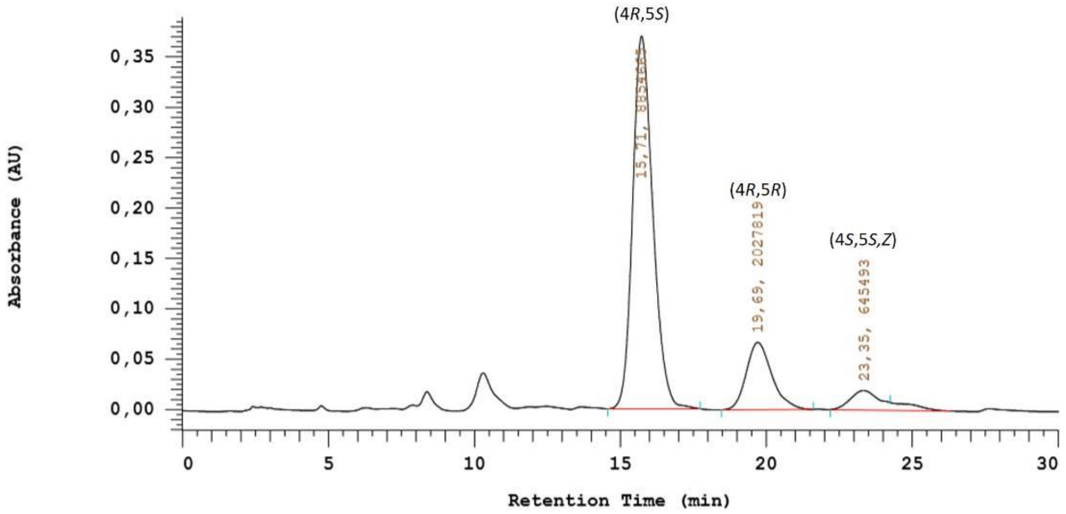

No.	RT	Area	Area %
1	15,71	8854665	76,810
2	19,69	2027819	17,590
3	23,35	645493	5,599
		11527977	100,000

Ethyl (4*S*,5*S*,7*S*,*E*)-7-[(*tert*-Butoxycarbonyl)amino]-4-isopropyl-5-methyl-6-oxo-8-phenyloct-2-enoate (13c)

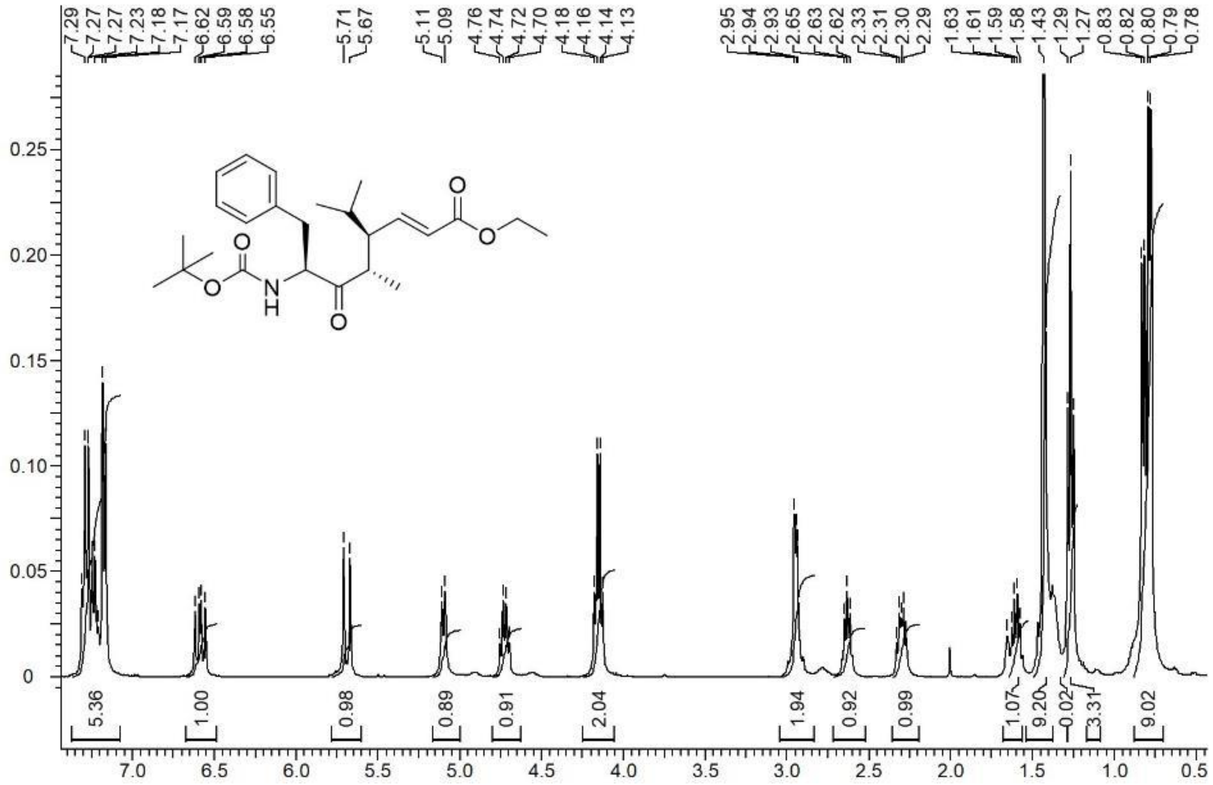

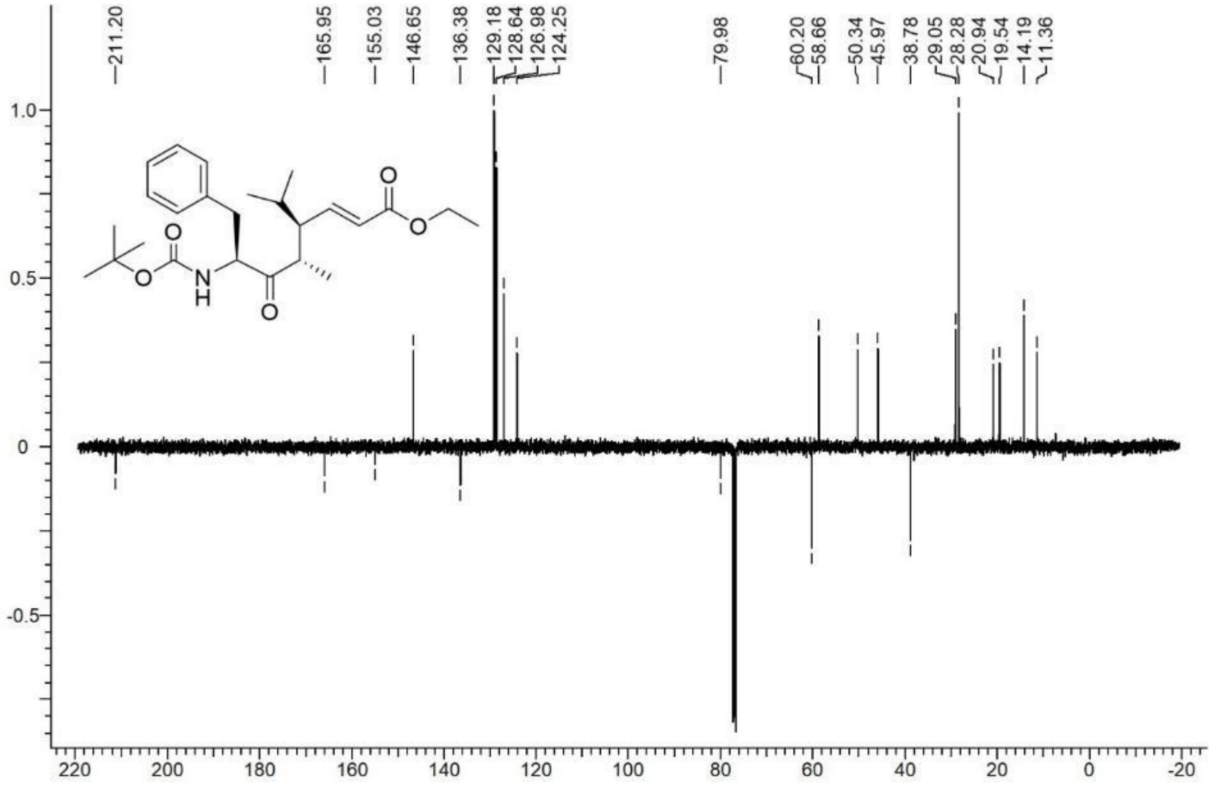

LCMS [(4*S*,5*S*,7*S*,*E*)-13c] – Table 1, entry 16
Column: Phenomenex Luna 3u C18(2) RP (50 x 4.6 mm, 100 Å, 5 μm)
Eluent: MeCN/H_2O 55:45 – 65:35, 30min, 1.0 ml/min

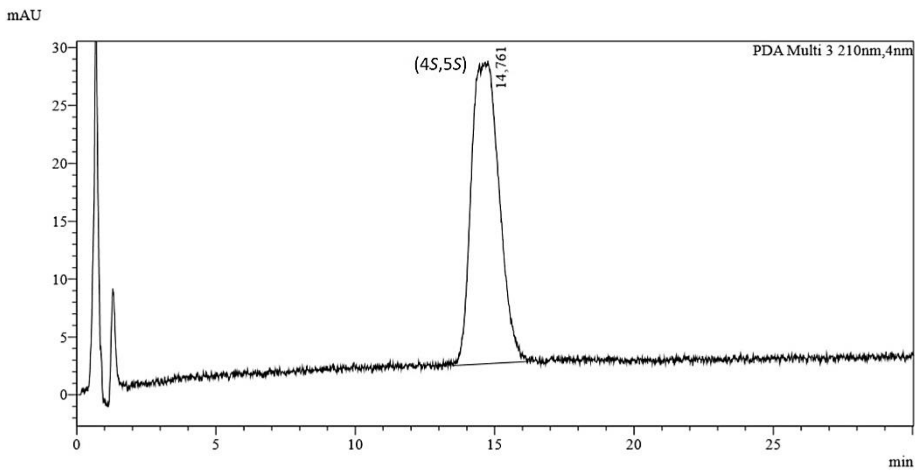

PDA Ch3 210nm

Peak#	Ret. Time	Area	Area%
1	14,761	1817882	100,000
Total		1817882	100,000

Ethyl (4*S*,5*R*,7*S*,*E*)-7-[(*tert*-butoxycarbonyl)amino]-6-oxo-4,5,8-triphenyloct-2-enoate (13d)

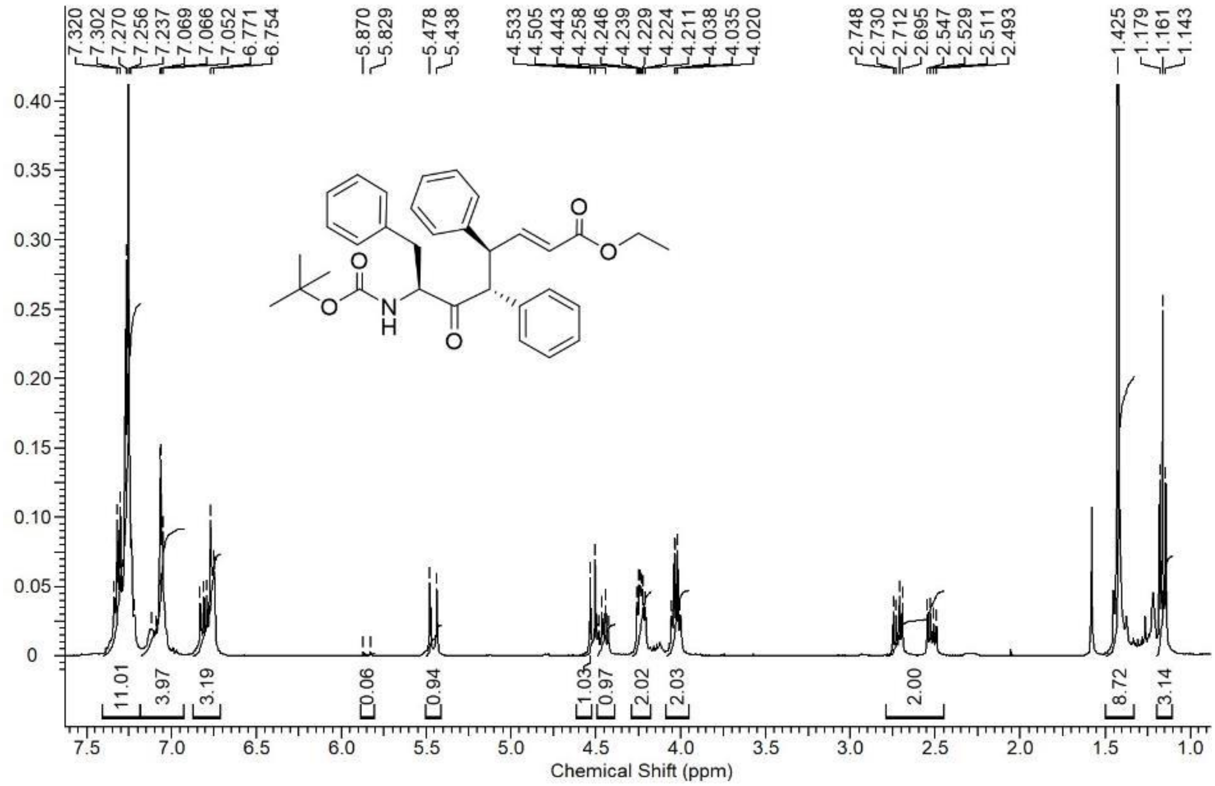

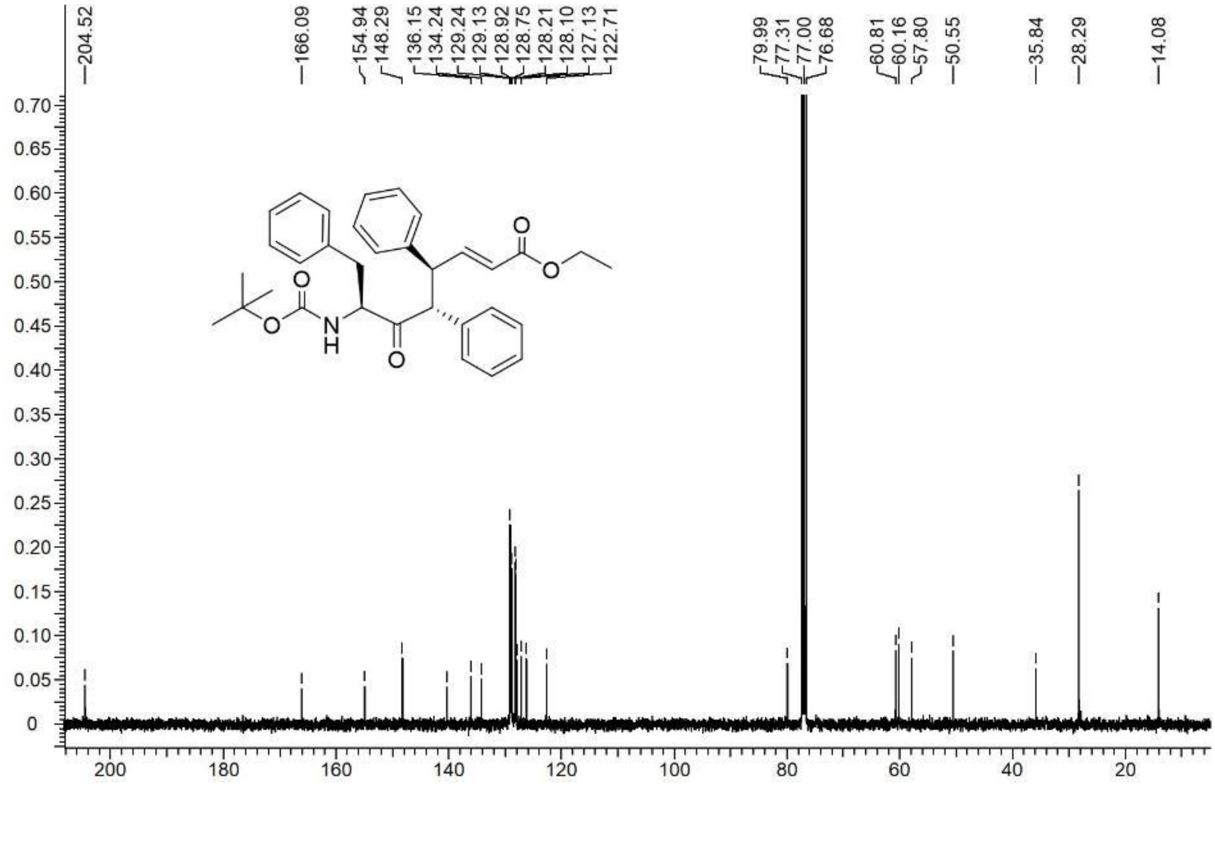

LCMS [(4*S*,5*R*,7*S*,*E*)-13d] – Table 1, entry 18
Column: Phenomenex Luna 3u C18(2) RP (50 x 4.6 mm, 100 Å, 5 μm)
Eluent: MeCN/H_2O 50:50 – 60:40, 35min, 1.0 ml/min

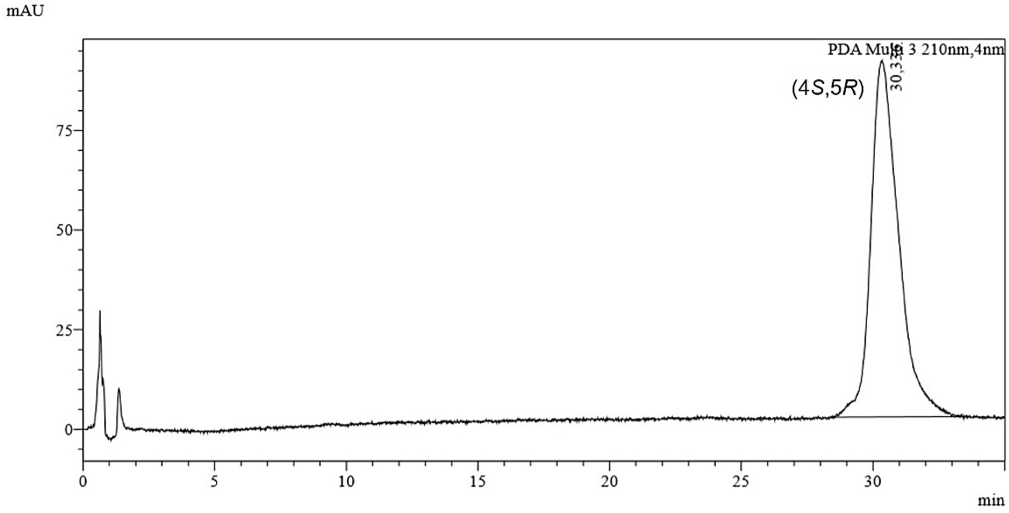

PDA Ch3 210nm

Peak#	Ret. Time	Area	Area%
1	30,336	6572350	100,000
Total		6572350	100,000

Ethyl (4*S*,5*R*,7*S*,*E*)-7-[(*tert*-butoxycarbonyl)amino]-4,8-dimethyl-6-oxo-5-phenylnon-2-enoate (14a)

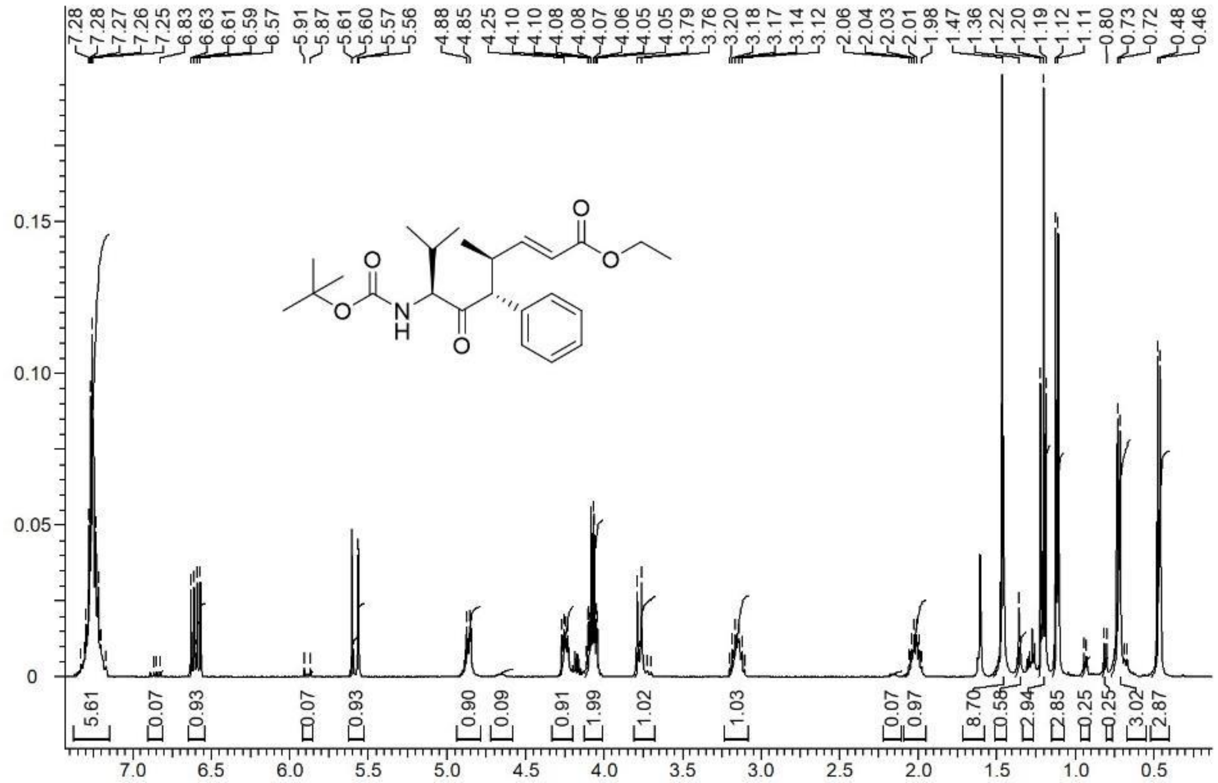

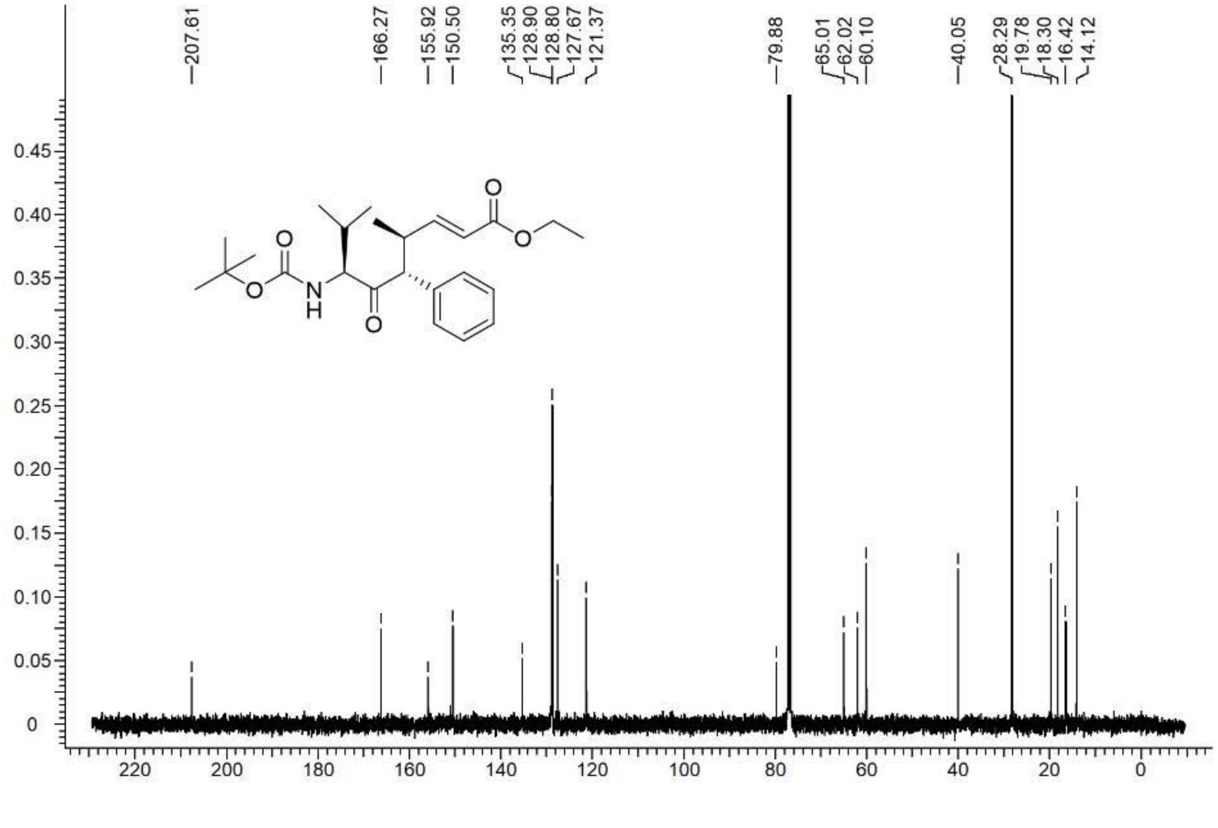

HPLC [(4*S*,5*R*,7*S*,*E*)-14a] – Table 1, entry 4
Column: Reprosil 100 Chiral-NR 8 µm
Eluent: Hexane/iPrOH 90:10, 1.5 ml/min, 20°C, 210 nm

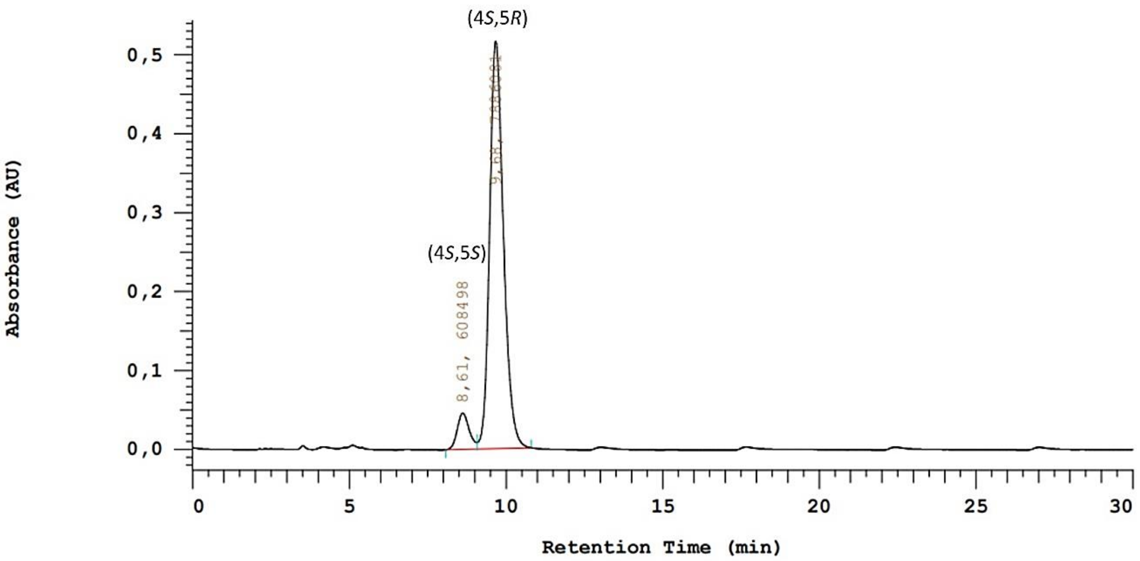

No.	RT	Area	Area %
1	8,61	608498	7,163
2	9,68	7886081	92,837
		8494579	100,000

Ethyl (4*R*,5*R*,7*S*,*E*)-7-[(*tert*-butoxycarbonyl)amino]-4,8-dimethyl-6-oxo-5-phenylnon-2-enoate (14a)

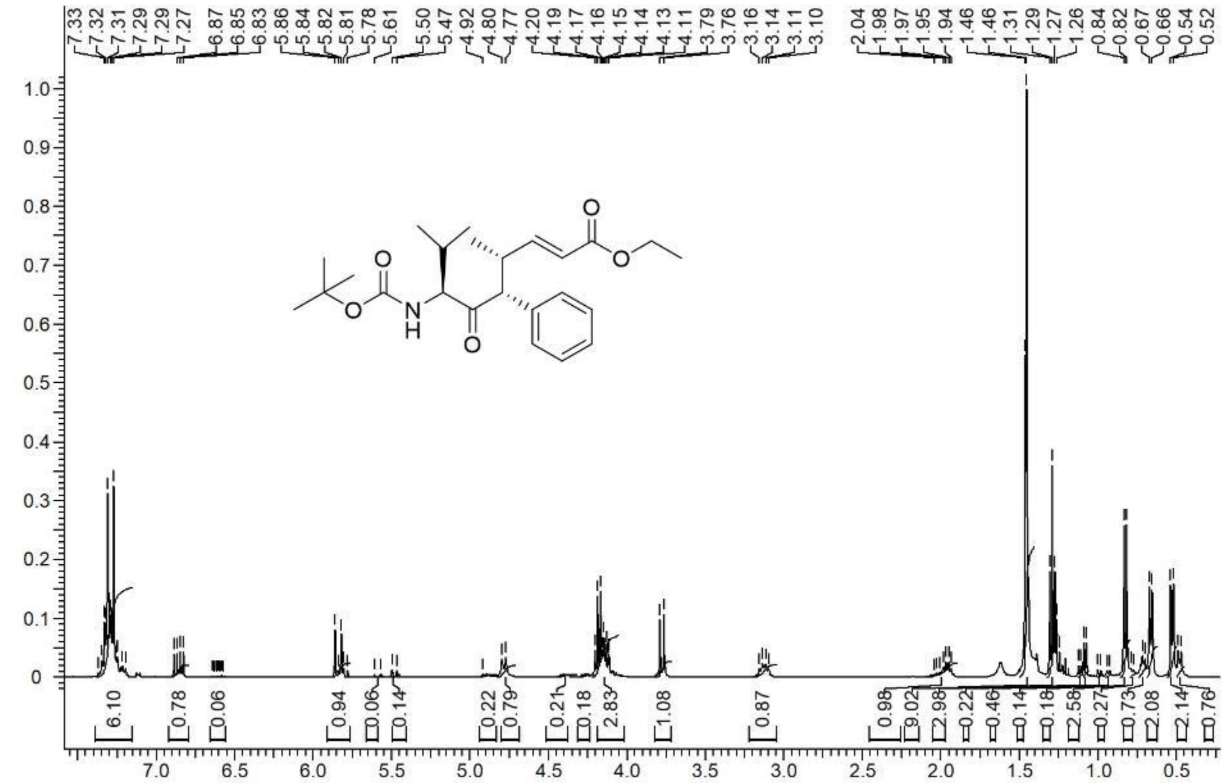

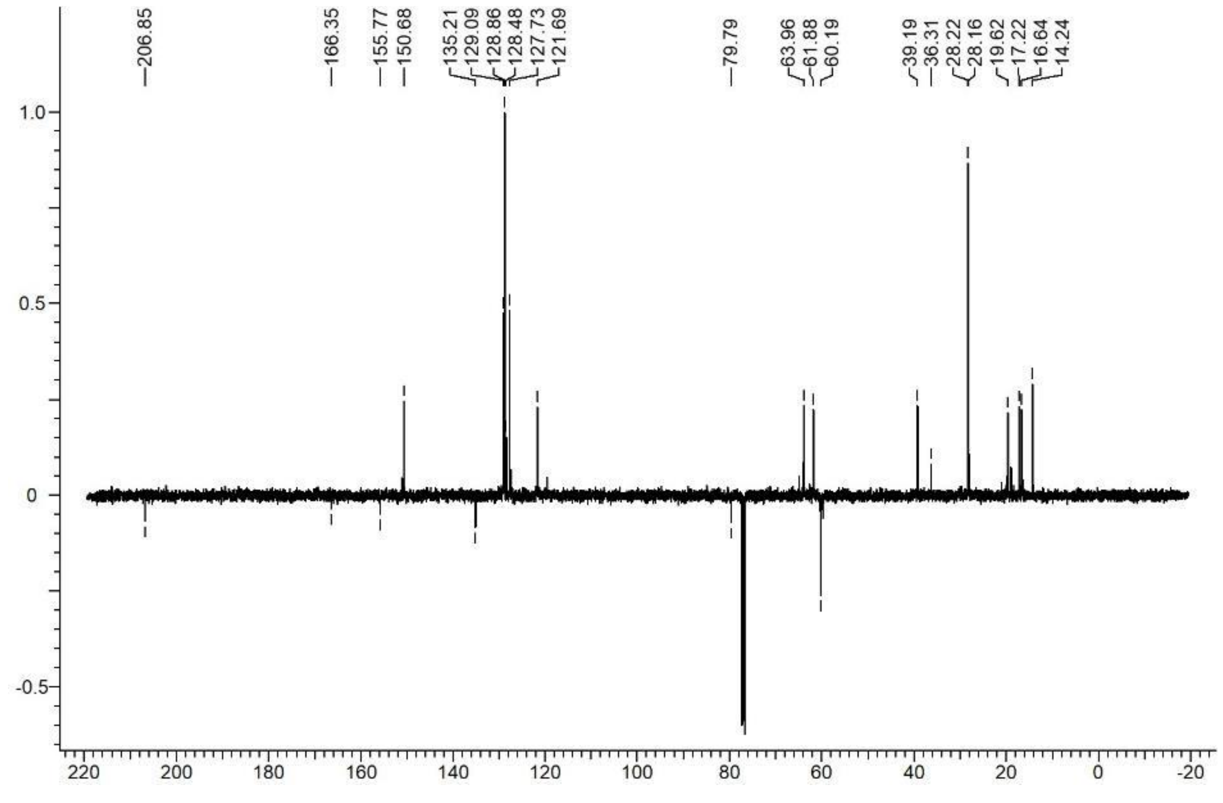

HPLC [(4*R*,5*R*,7*S*,*E*)-14a] – Table 1, entry 7
Column: Reprosil 100 Chiral-NR 8 µm
Eluent: Hexane/iPrOH 99.5:0.5-95:5, 40min, 1.0mL/min, 210nm

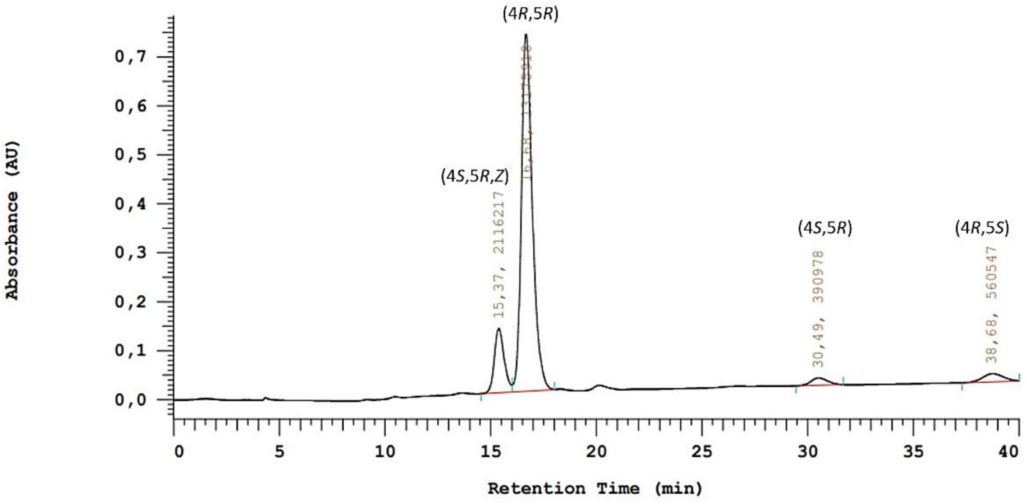

No.	RT	Area	Area %
1	15,37	2116217	13,028
2	16,68	13175918	81,114
3	30,49	390978	2,407
4	38,68	560547	3,451
		16243660	100,000

Ethyl (4*S*,5*S*,7*S*,*E*)-7-[(*tert*-butoxycarbonyl)amino]-5-isopropyl-4,8-dimethyl-6-oxonon-2-enoate (14b)

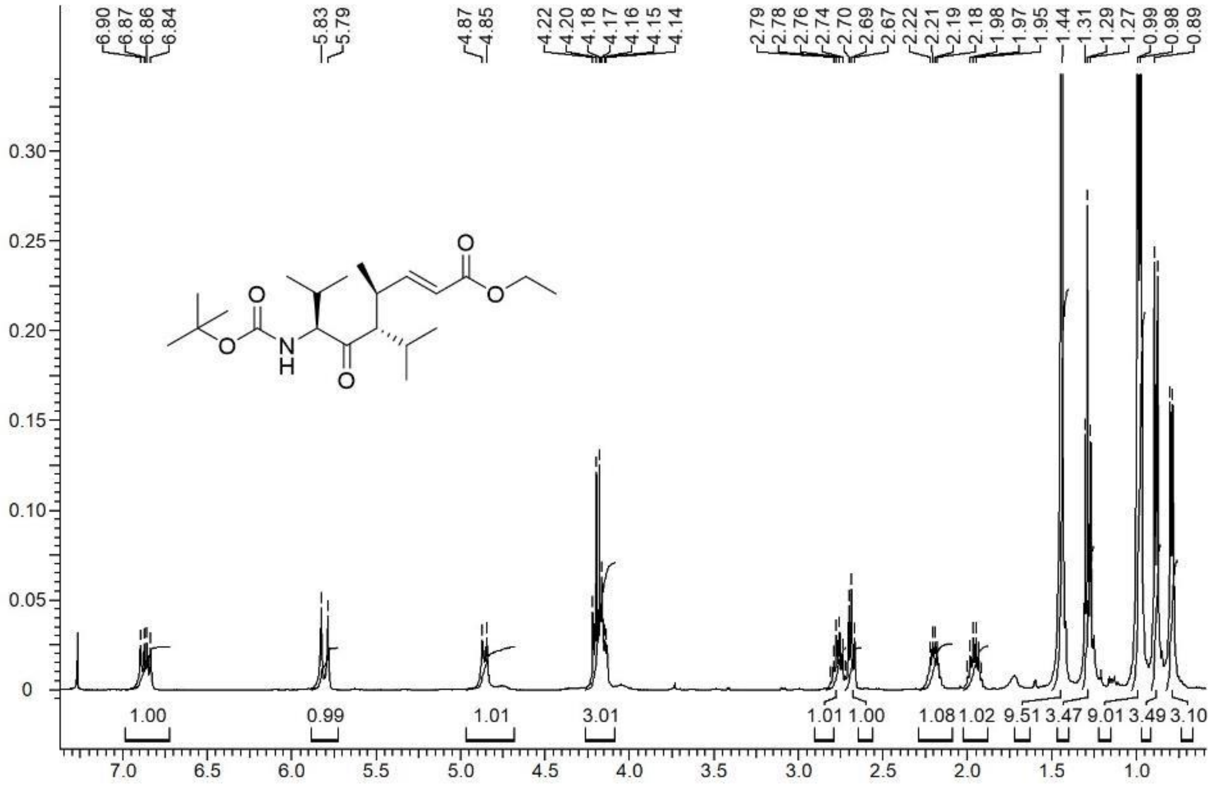

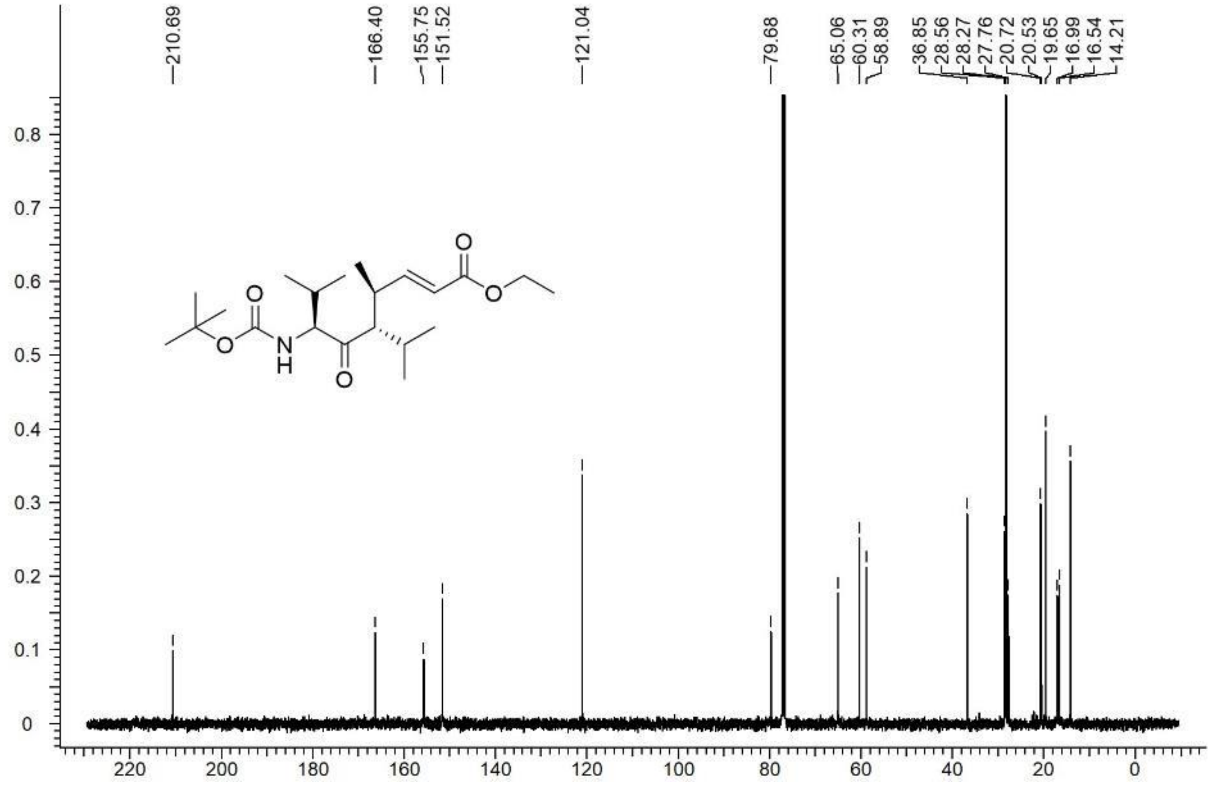

LCMS [crude (4*S*,5*S*,7*S*,*E*)-14b] – Table 1, entry 15
Column: Phenomenex Luna 3u C18(2) RP (50 x 4.6 mm, 100 Å, 5 μm)
Eluent: MeCN/H_2O 50:50 – 65:35, 30min, 1.0 ml/min

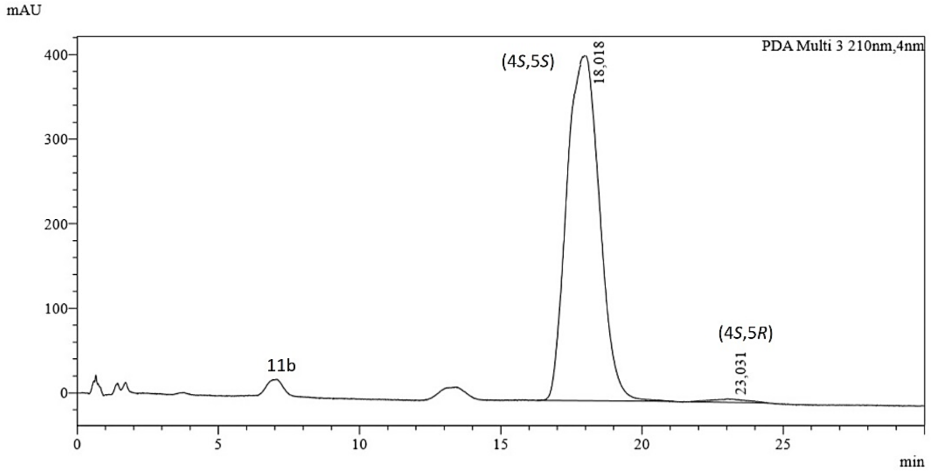

PDA Ch3 210nm

Peak#	Ret. Time	Area	Area%
1	18,018	32591078	98,969
2	23,031	339648	1,031
Total		32930727	100,000

Ethyl (4*S*,5*R*,7*S*,*E*)-7-[(*tert*-butoxycarbonyl)amino]-4-isopropyl-8-methyl-6-oxo-5-phenylnon-2-enoate (14c)

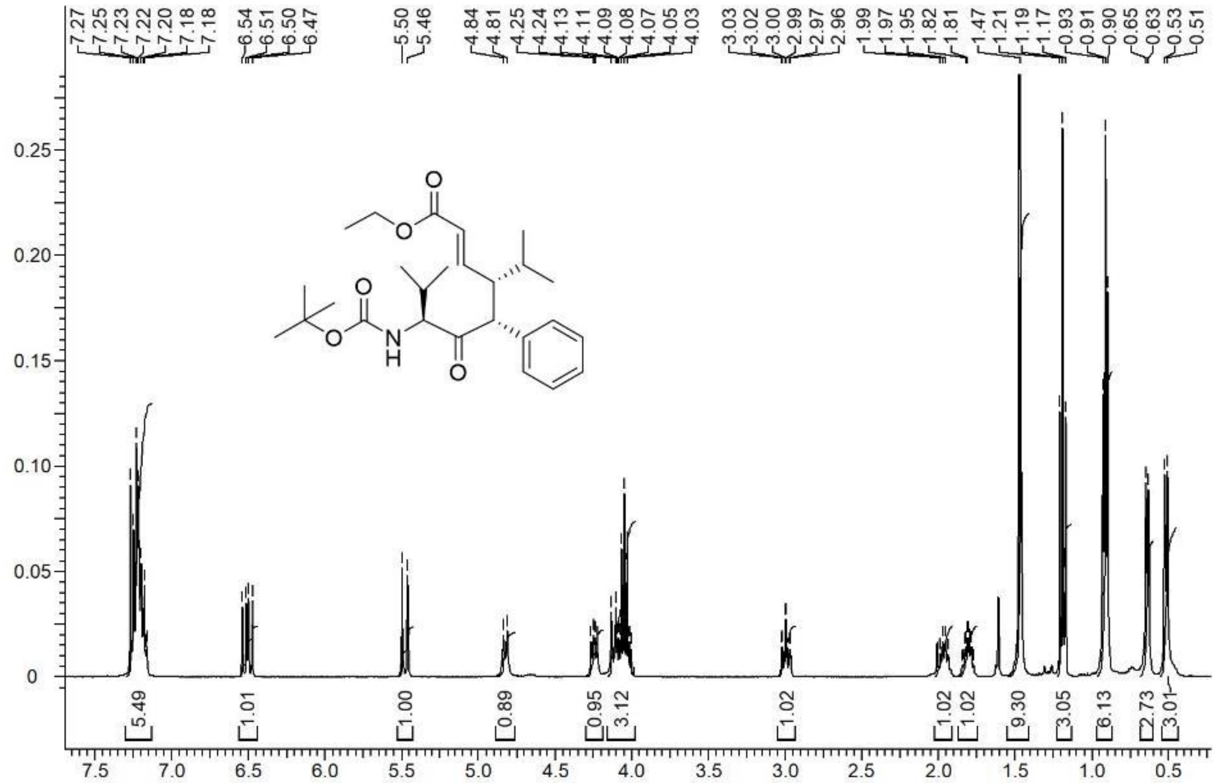

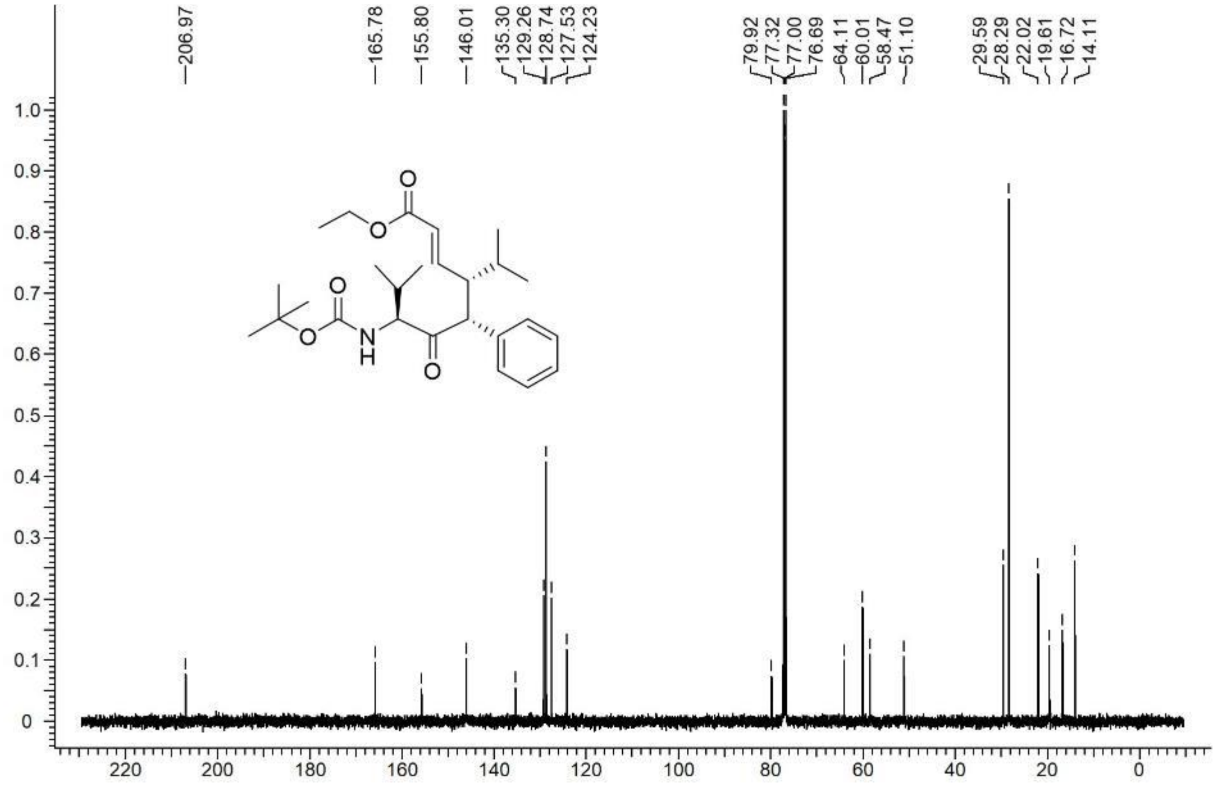

LCMS [(4*S*,5*R*,7*S*,*E*)-14c] – Table 1, entry 17
Column: Phenomenex Luna 3u C18(2) RP (50 x 4.6 mm, 100 Å, 5 μm)
Eluent: MeCN/H_2O 50:50 – 65:35, 30min, 1.0 ml/min

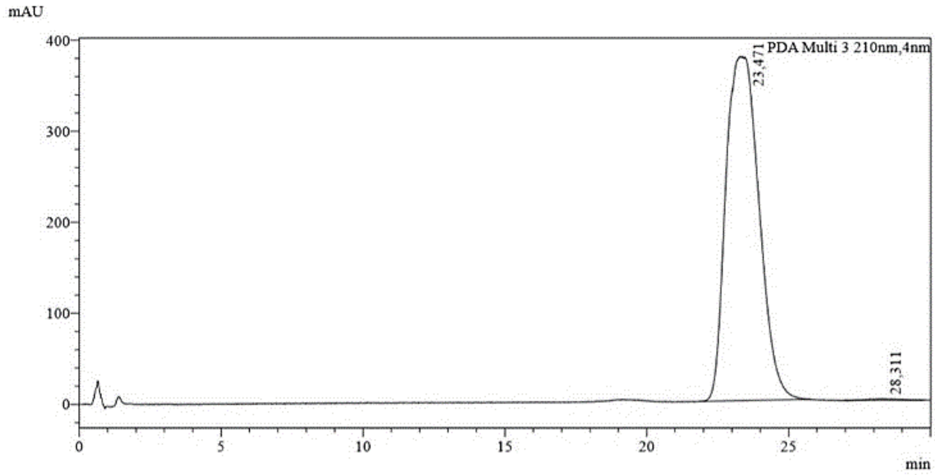

PDA Ch3 210nm

Peak#	Ret. Time	Area	Area%
1	23,471	30501180	99,629
2	28,311	113712	0,371
Total		30614892	100,000

Ethyl (4*S*,5*R*,7*S*,8*S*,*E*)-7-[(*tert*-butoxycarbonyl)amino]-4,8-dimethyl-6-oxo-5-phenyldec-2-enoate (15a)

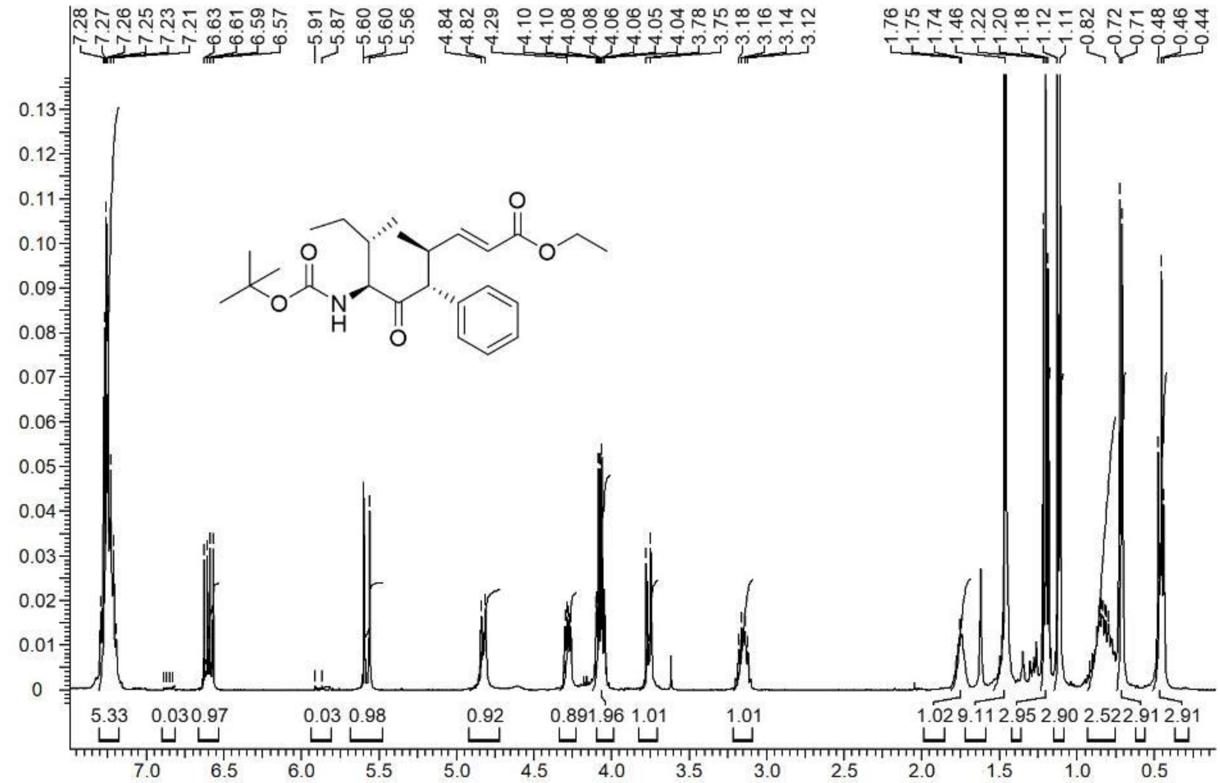

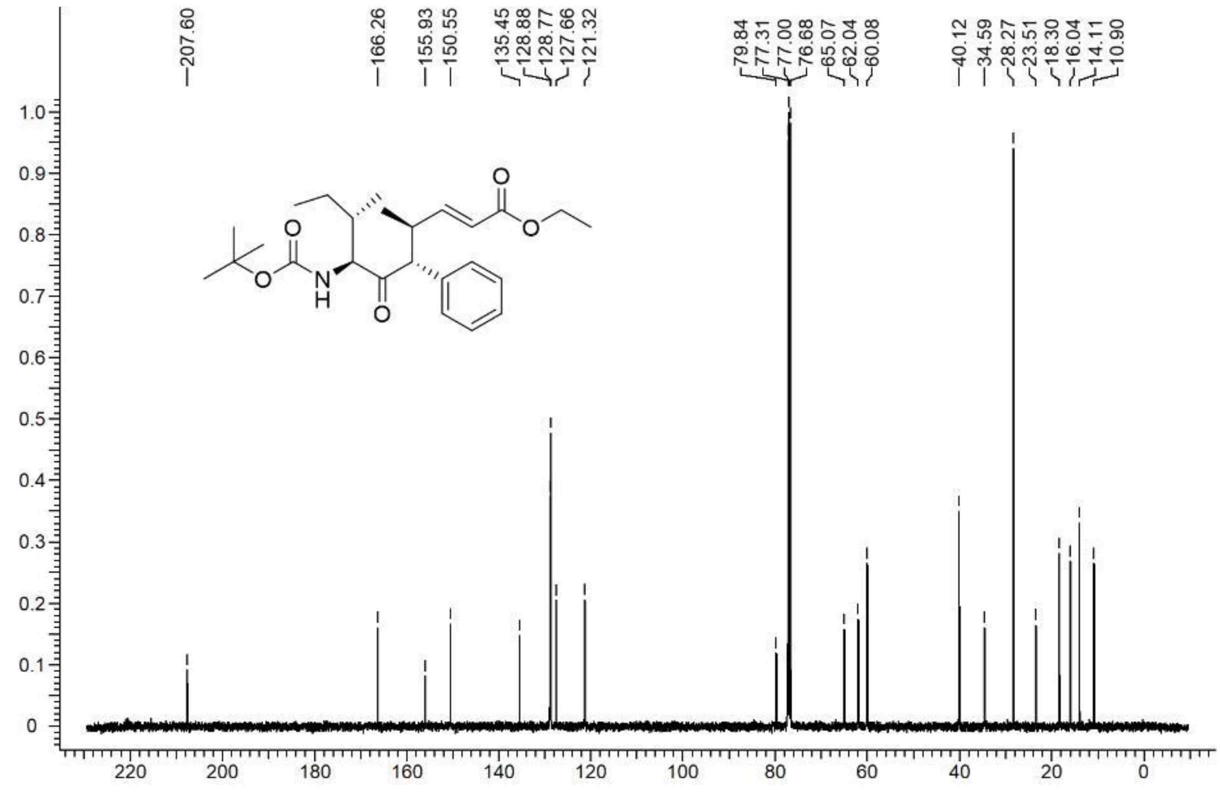

HPLC [(4*S*,5*R*,7*S*,*E*)-15a] – Table 1, entry 8
Column: Reprosil 100 Chiral-NR 8 µm
Eluent: Hexane/iPrOH 99.5:0.5 – 95:5, 40 min, 1.0 ml/min, 20°C, 220 nm

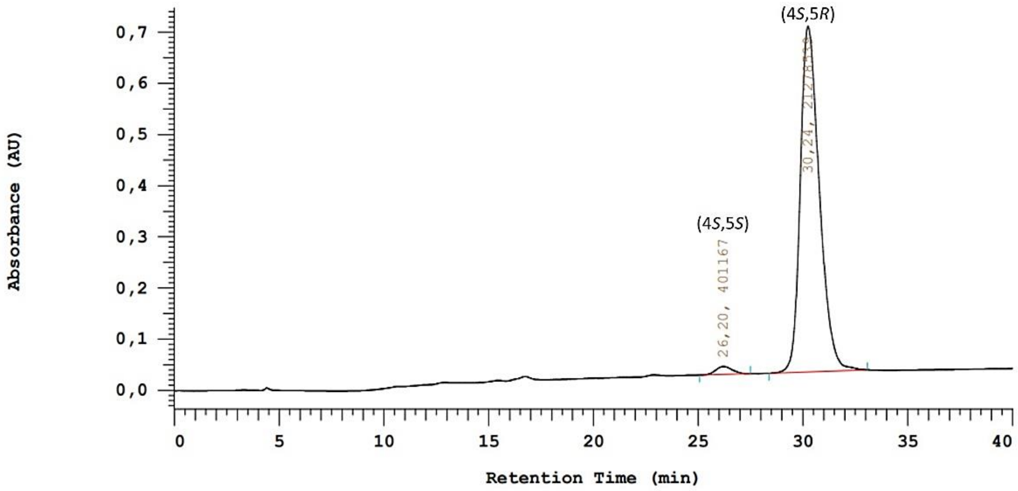

No.	RT	Area	Area %
1	26,20	401167	1,850
2	30,24	21278539	98,150
		21679706	100,000

Ethyl (4*R*,5*R*,7*S*,8*S*,*E*)-7-[(*tert*-butoxycarbonyl)amino]-4,8-dimethyl-6-oxo-5-phenyldec-2-enoate (15a)

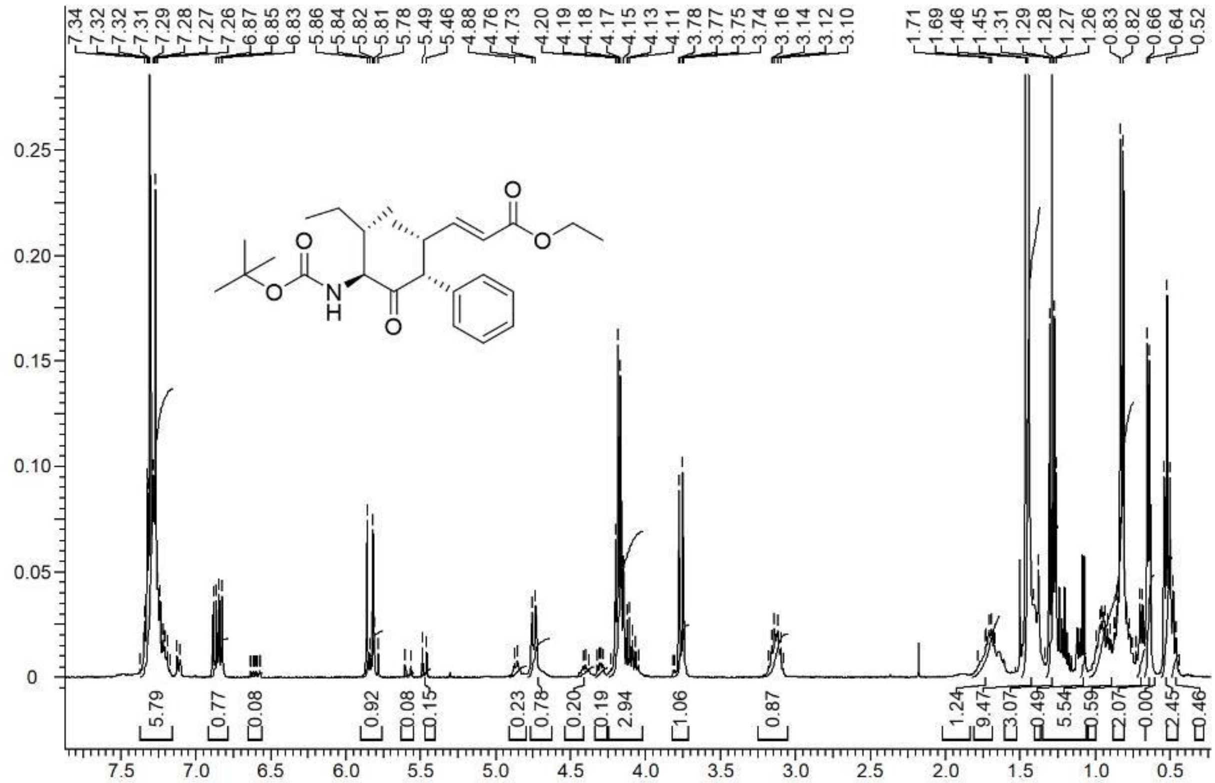

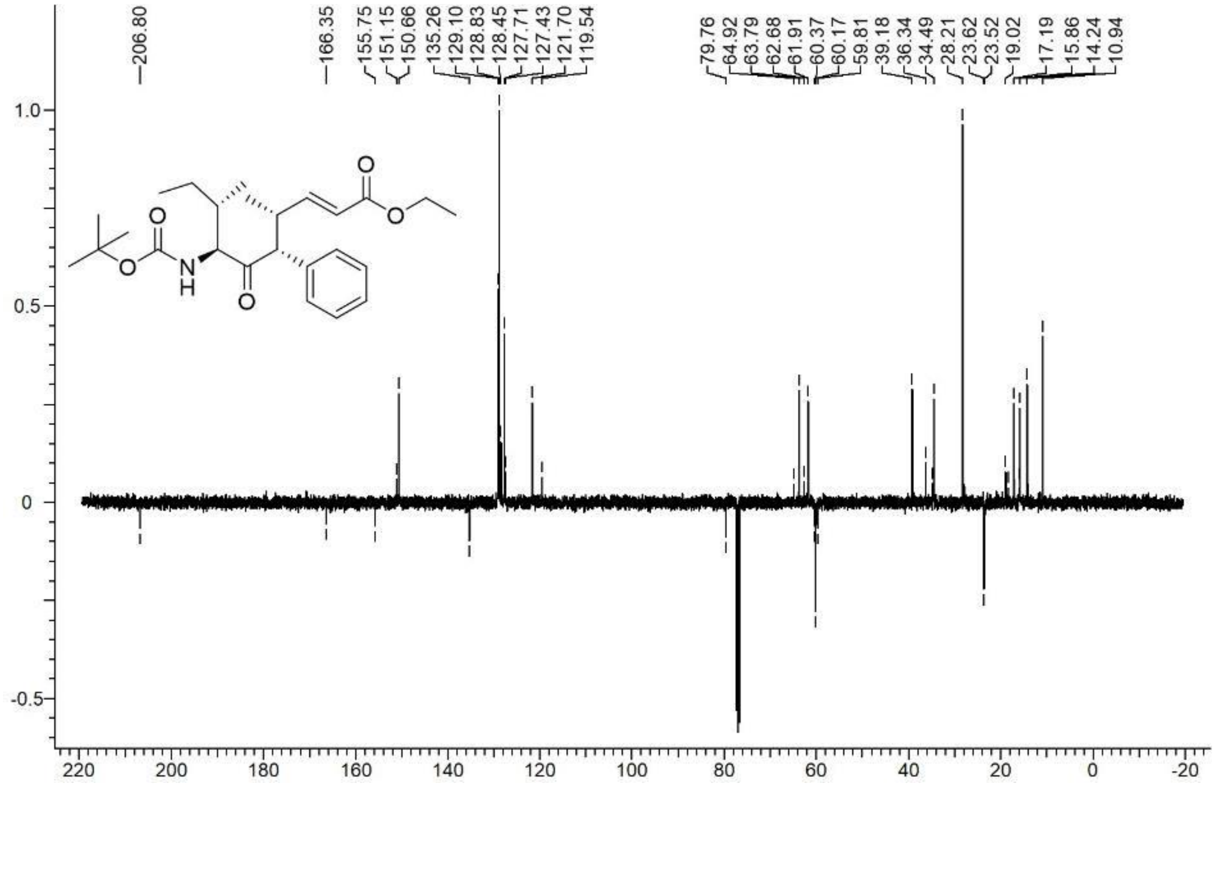

HPLC [(4*R*,5*R*,7*S*,*E*)-15a] – Table 1, entry 10
Column: Reprosil 100 Chiral-NR 8 µm
Eluent: Hexane/iPrOH 99.5:0.5 – 95:5, 40 min, 1.0 ml/min, 20°C, 220 nm

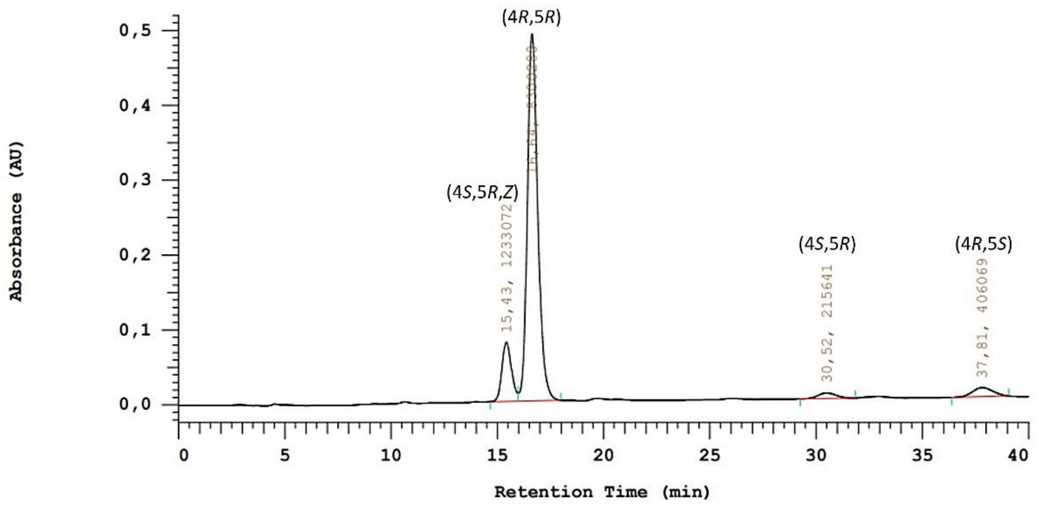

No.	RT	Area	Area %
1	15,43	1233072	12,142
2	16,64	8300280	81,735
3	30,52	215641	2,123
4	37,81	406069	3,999
		10155062	100,000

(5*S*,6*R*,8*S*,*E*)-8-(tert-Butoxycarbonyl)amino-5-methyl-6,9-diphenylnon-3-ene-2,7-dion [(5*S*,6*R*)-16a]

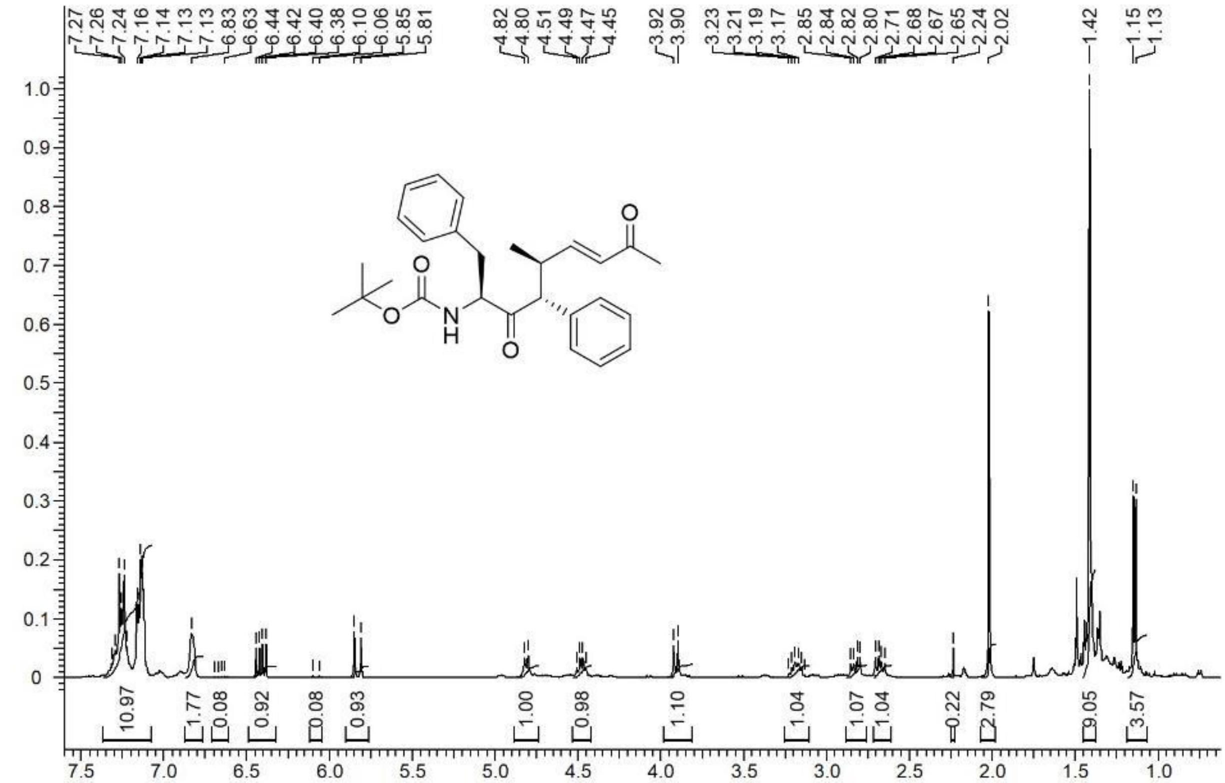

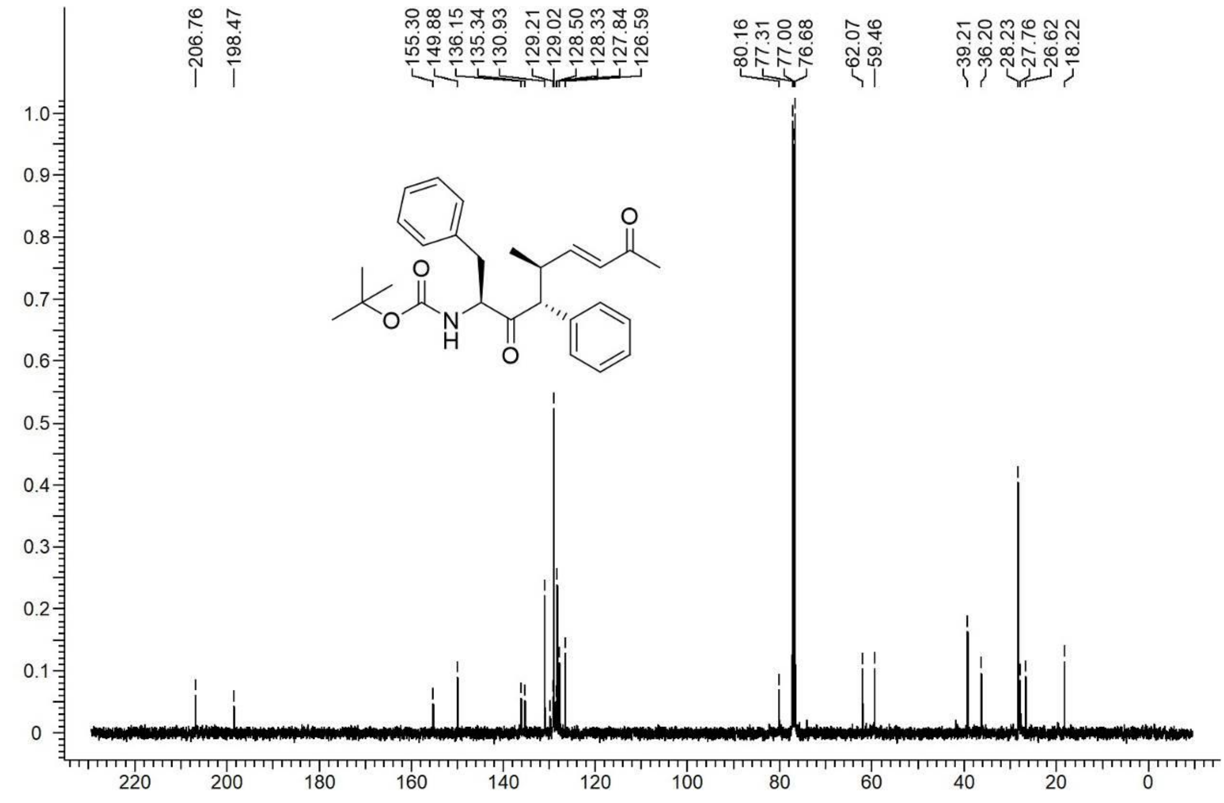

HPLC [(5*S*,6*R*)-16a] – Table 2, entry 1
Column: Reprosil 100 Chiral-NR 8 µm
Eluent: Hexane/iPrOH 90:10, 1.5 ml/min, 20°C, 220 nm

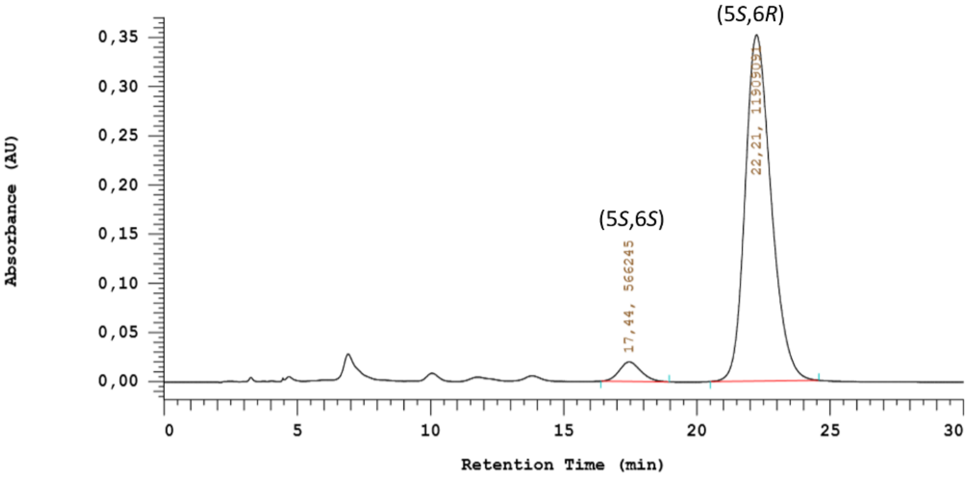

No.	RT	Area	Area %
1	17,44	566245	4,539
2	22,21	11909091	95,461
		12475336	100,000

(5*S*,6*S*,8*S*,*E*)-8-(*tert*-Butoxycarbonyl)amino-5,6-dimethyl-9-phenylnon-3-ene-2,7-dione [(5*S*,6*S*)-16b]

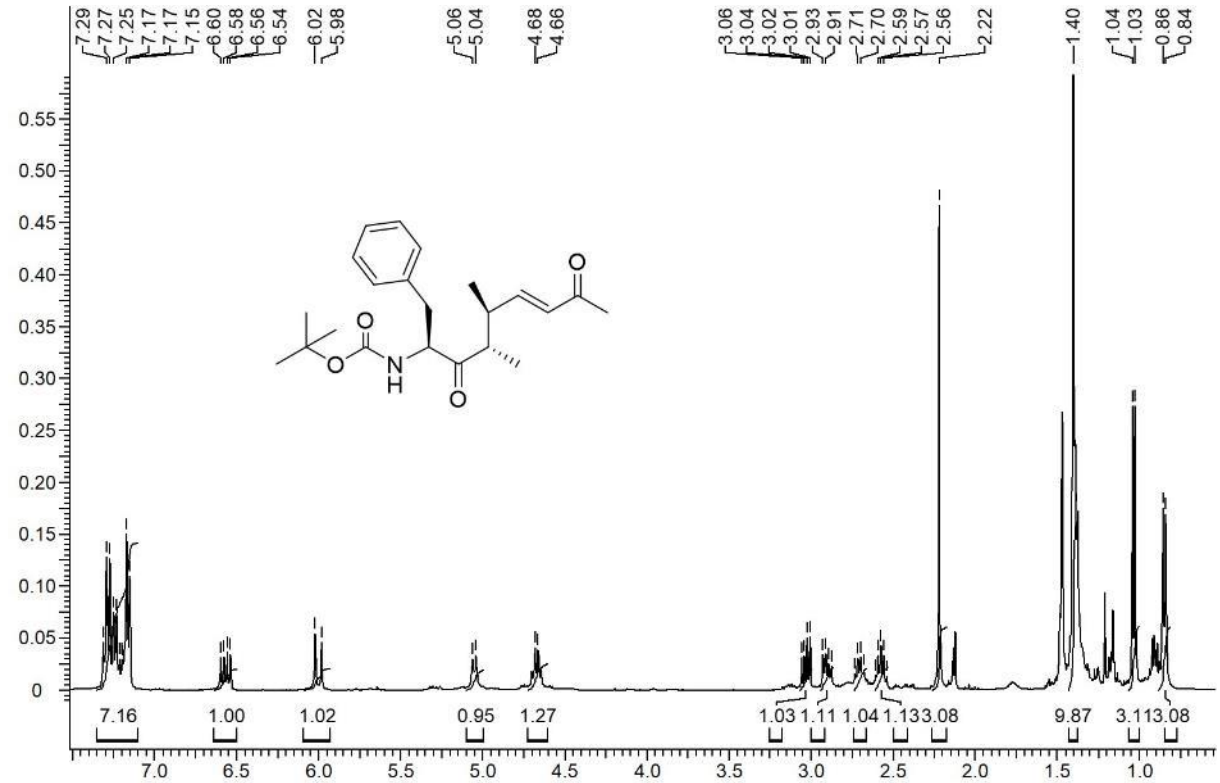

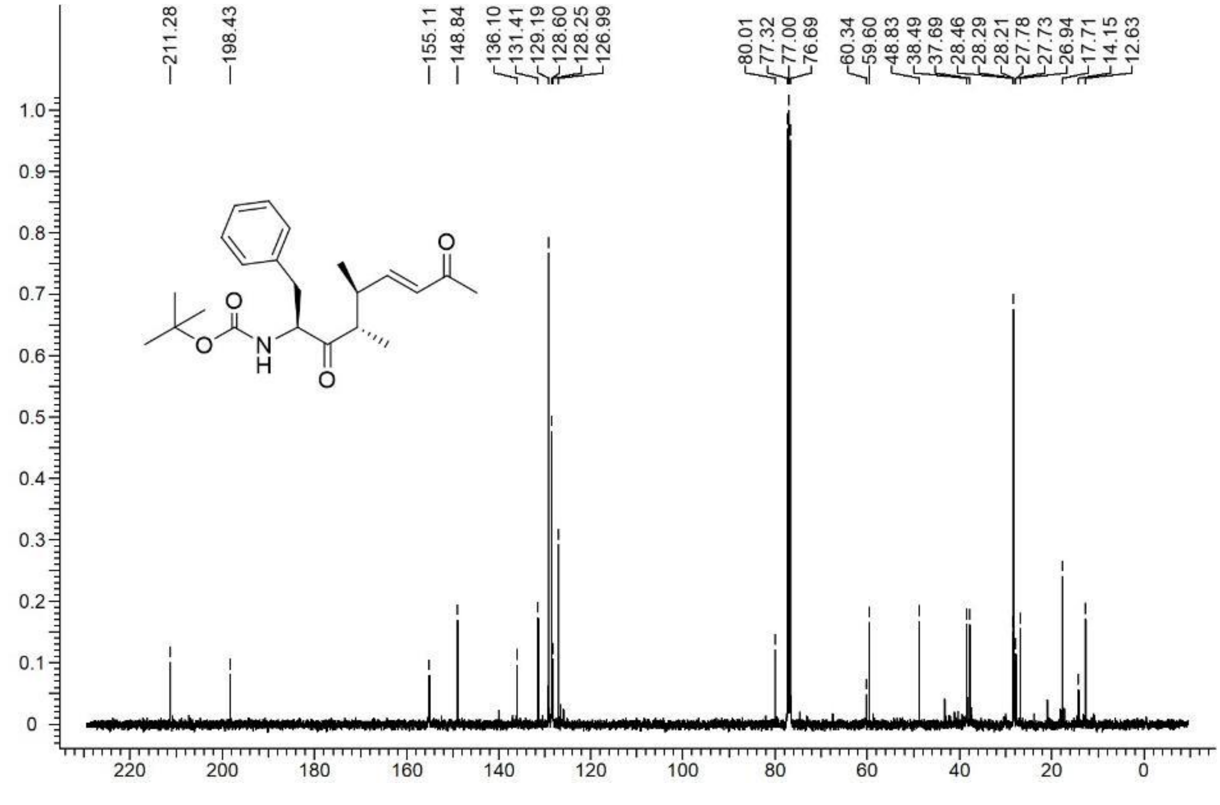

LCMS [(5S,6*S*)-16b] – Table 2, entry 6
Column: Reprosil 100 Chiral-NR 8 µm
Eluent: Hexane/iPrOH 90:10, 1.0 ml/min, 20°C, 220 nm

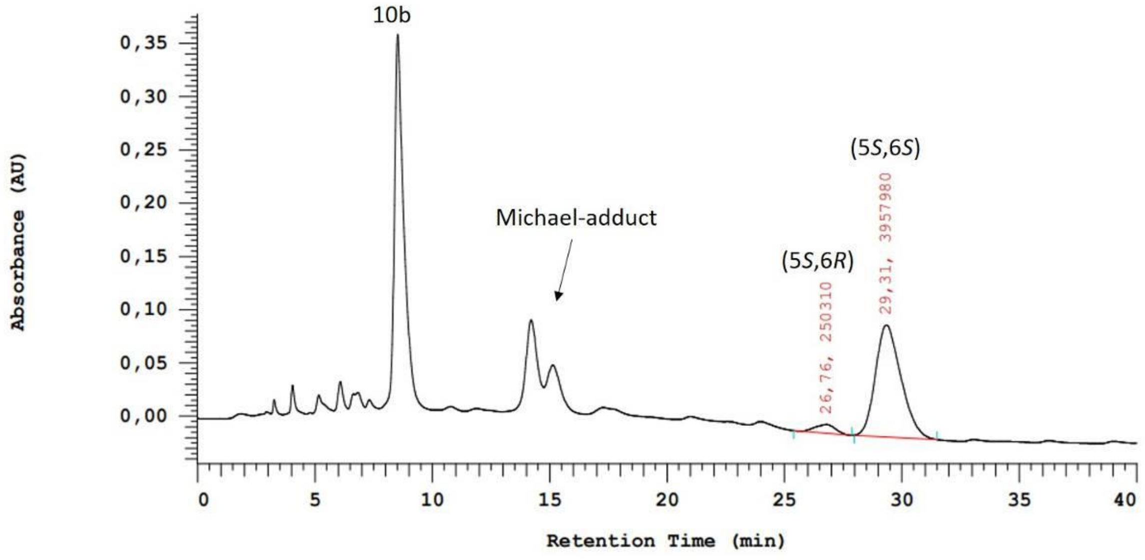

No.	RT	Area	Area %
1	26,76	250310	5,948
2	29,31	3957980	94,052
		4208290	100,000

(5*S*,6*R*,8*S*,*E*)-8-(*tert*-Butoxycarbonyl)amino-5,9-dimethyl-6-phenyldec-3-ene-2,7-dione [(5*S*,6*R*)-17a]

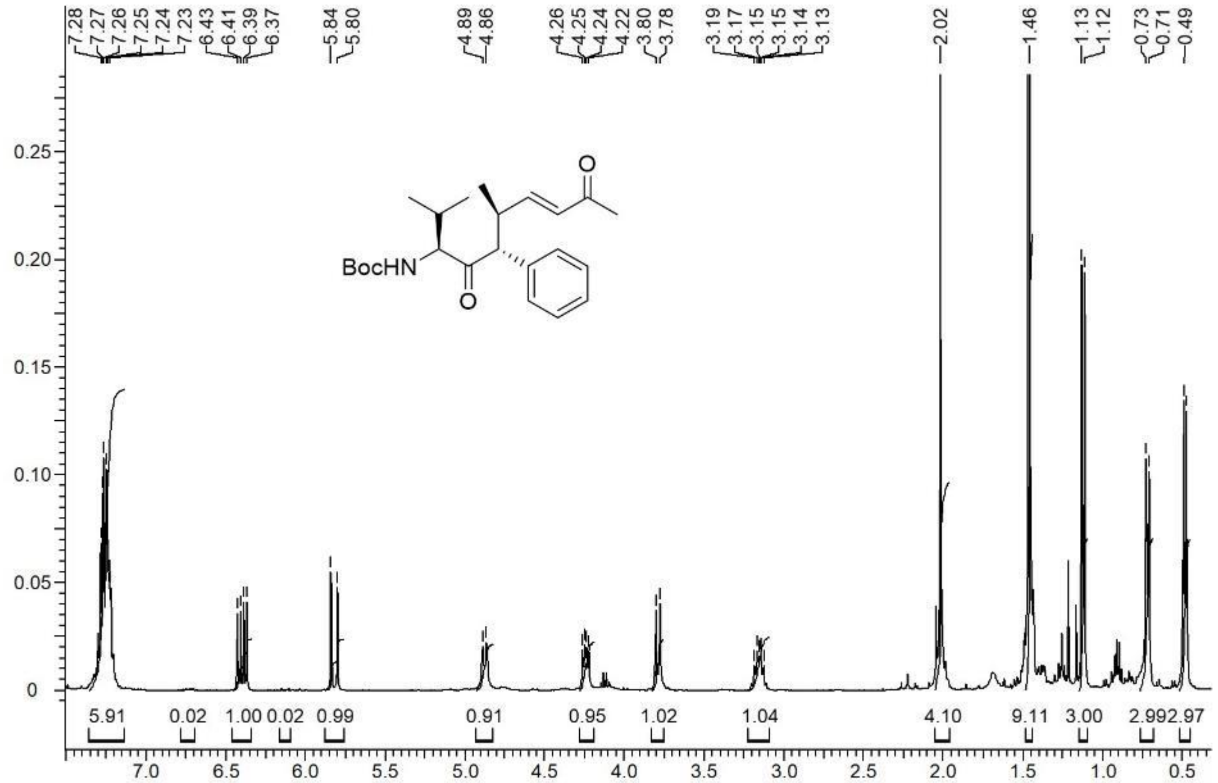

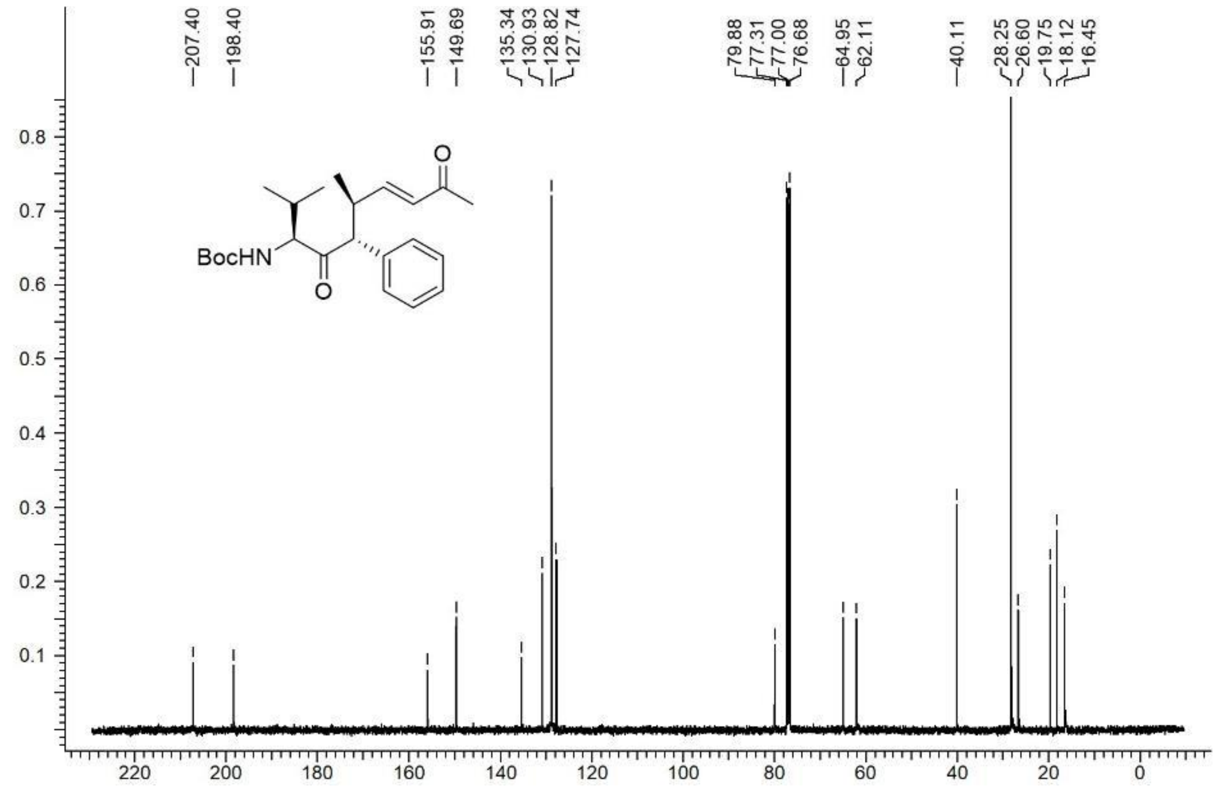

LCMS [(5*S*,6*R*)-17a] – Table 2, entry 3
Column: Phenomenex Luna 3u C18(2) RP (50 x 4.6 mm, 100 Å, 5 µm)
Eluent: MeCN/H2O 40:60 – 50:50, 40min, 1.0 ml/min

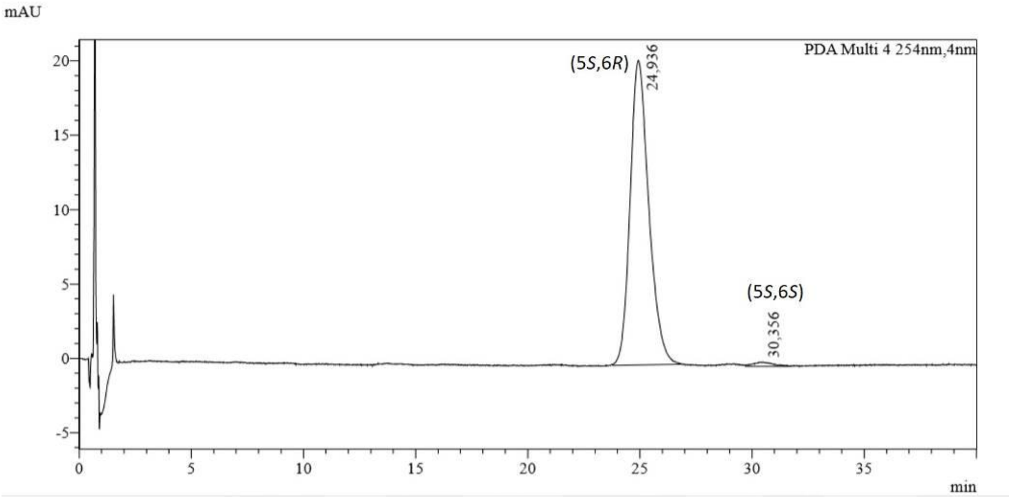

PDA Ch4 254nm

Peak#	Ret. Time	Area	Area%
1	24,936	1187233	98,459
2	30,356	18587	1,541
Total		1205820	100,000

(5*S*,6*R*,8*S*,9*S*,*E*)-8-(*tert*-Butoxycarbonyl)amino-5,9-dimethyl-6-phenylundec-3-ene-2,7-dione [(5*S*,6*R*)-18a]

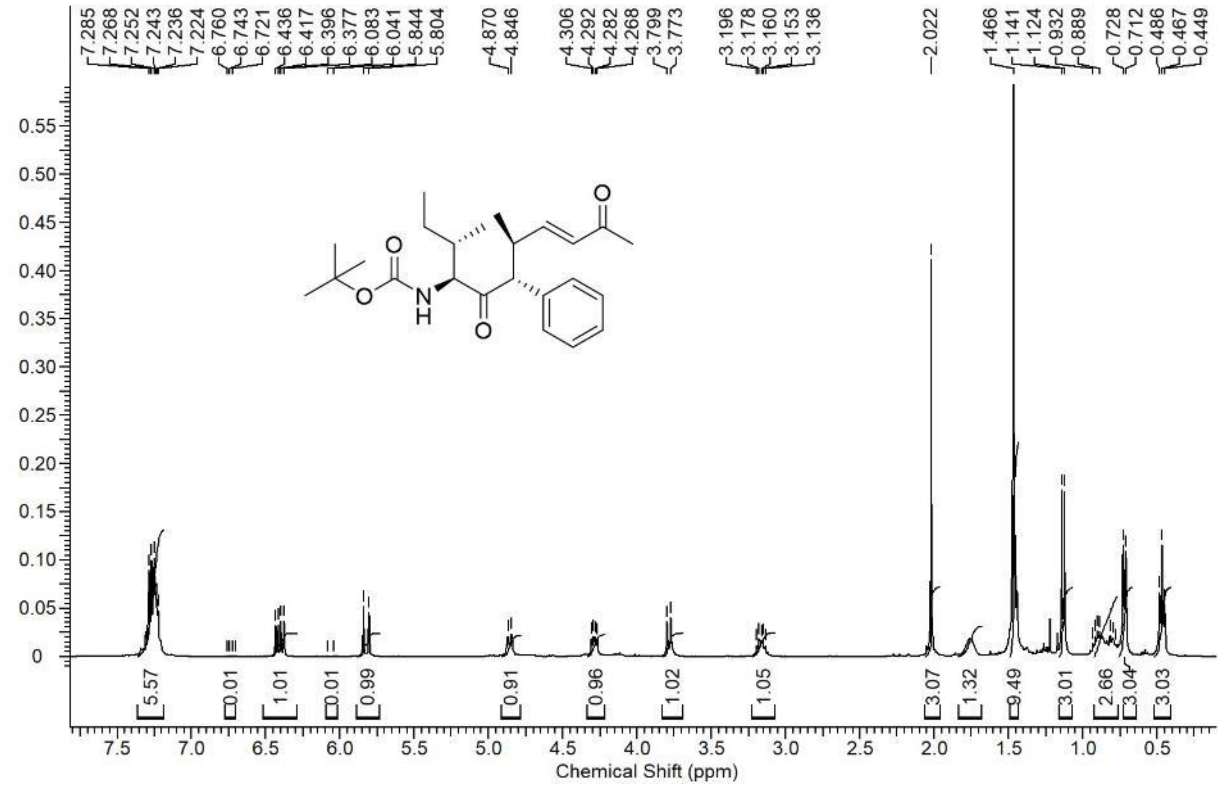

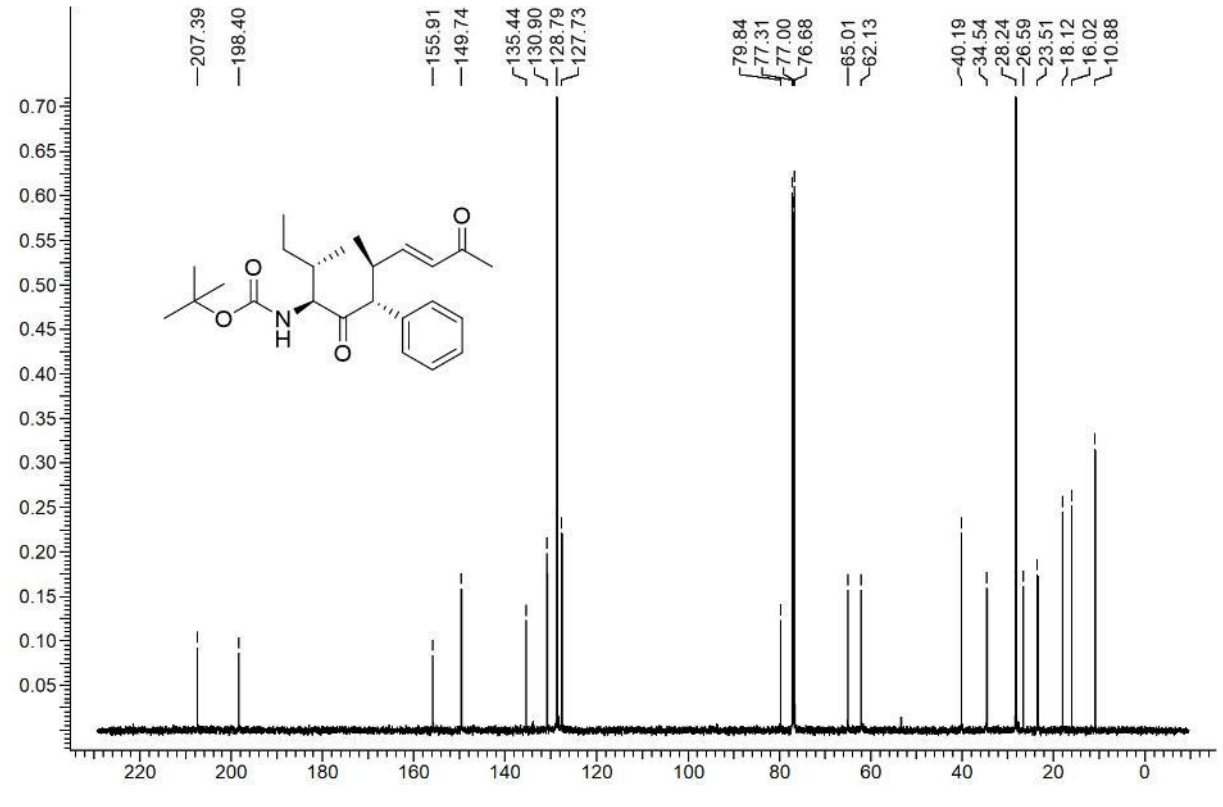

LCMS [(5*S*,6*R*)-18a] – Table 2, entry 5
Column: Phenomenex Luna 3u C18(2) RP (50 x 4.6 mm, 100 Å, 5 µm)
Eluent: MeCN/H_2O 40:60 – 50:50, 40min, 1.0 ml/min

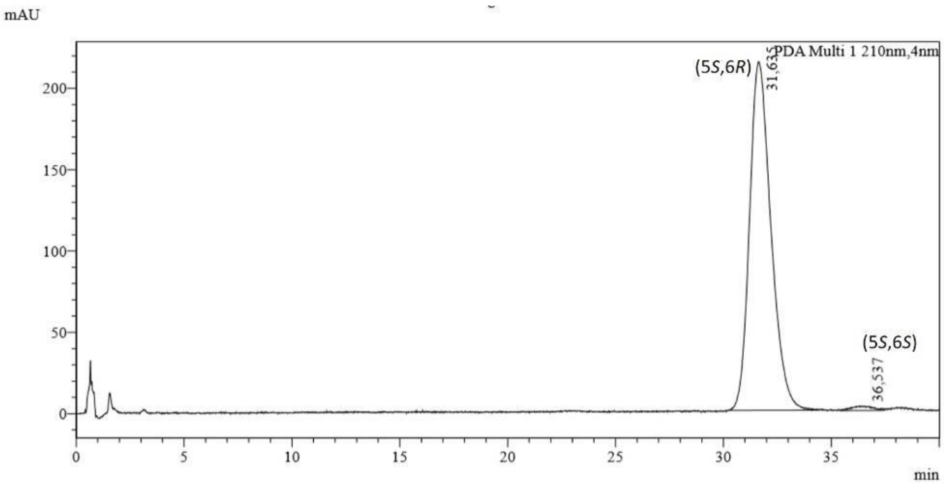

PDA Ch1 210nm

Peak#	Ret. Time	Area	Area%
1	31,635	14799699	98,688
2	36,537	196716	1,312
Total		14996415	100,000

(4*S*/*R*,5*R*,7*S*)-7-((*tert*-Butoxycarbonyl)amino)-4-methyl-6-oxo-5,8-diphenyloct-2-enenitrile (19a)

– E/Z-mixture

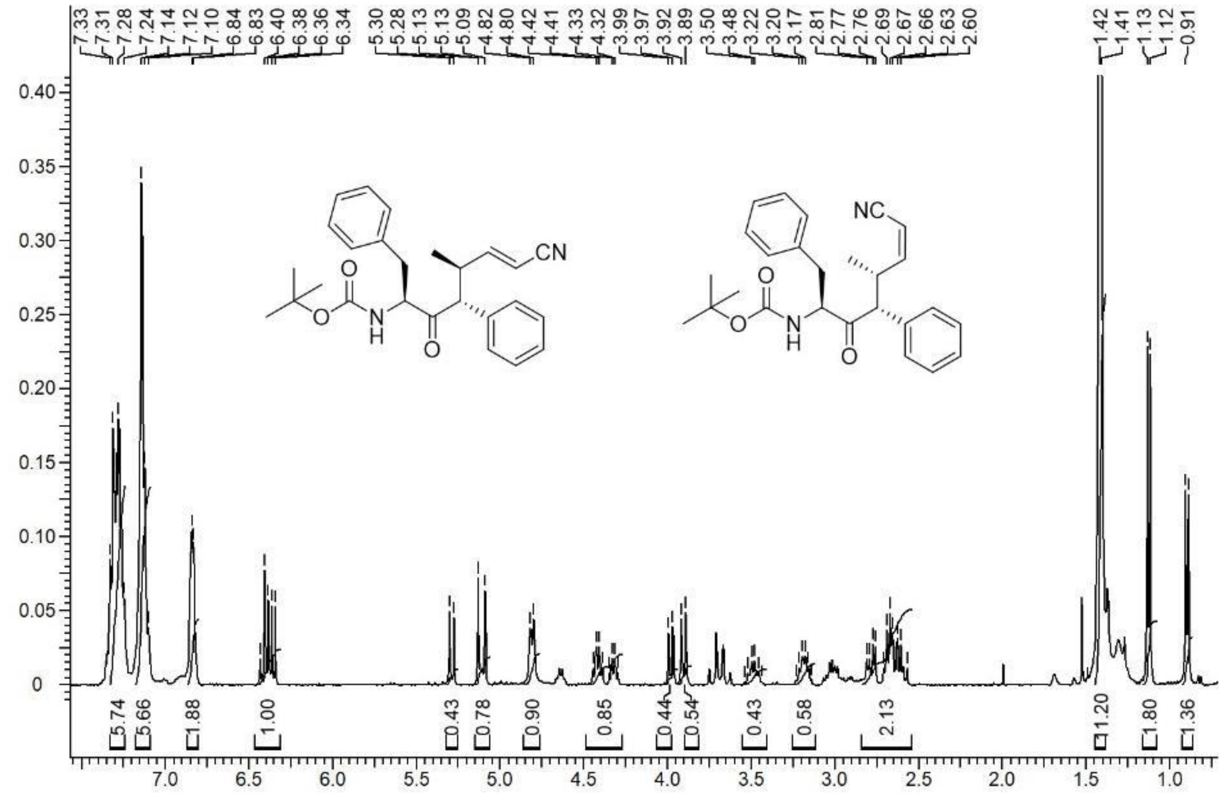

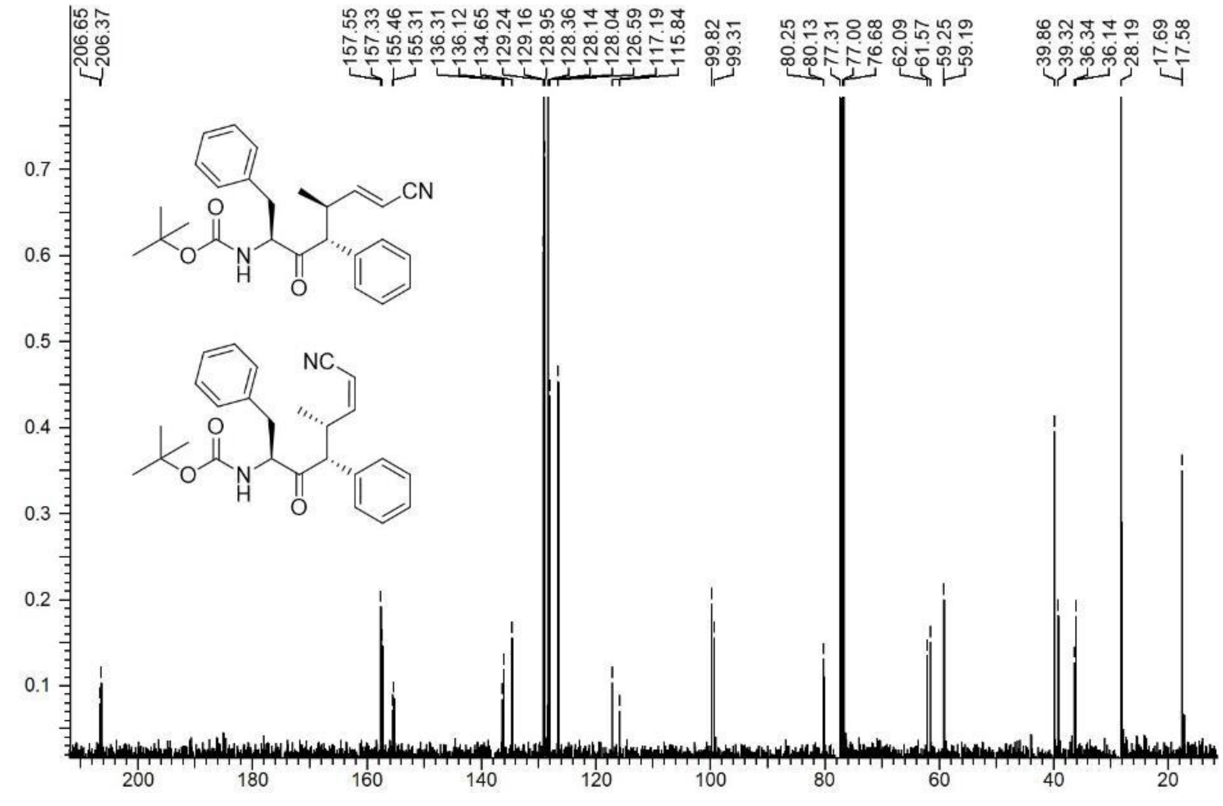

(4*S*,5*R*,7*S*,*E*)-7-((*tert*-Butoxycarbonyl)amino)-4-methyl-6-oxo-5,8-diphenyloct-2-enenitrile [(4*S*,*E*)-19a]

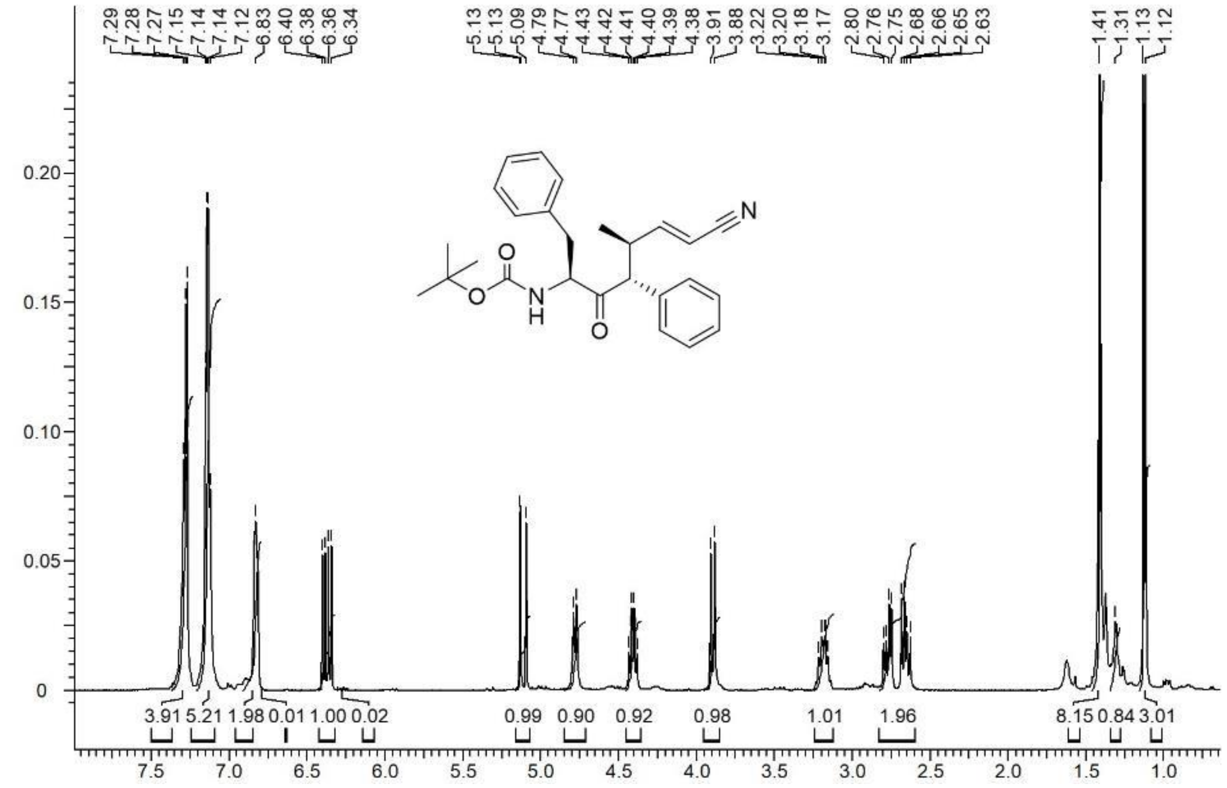

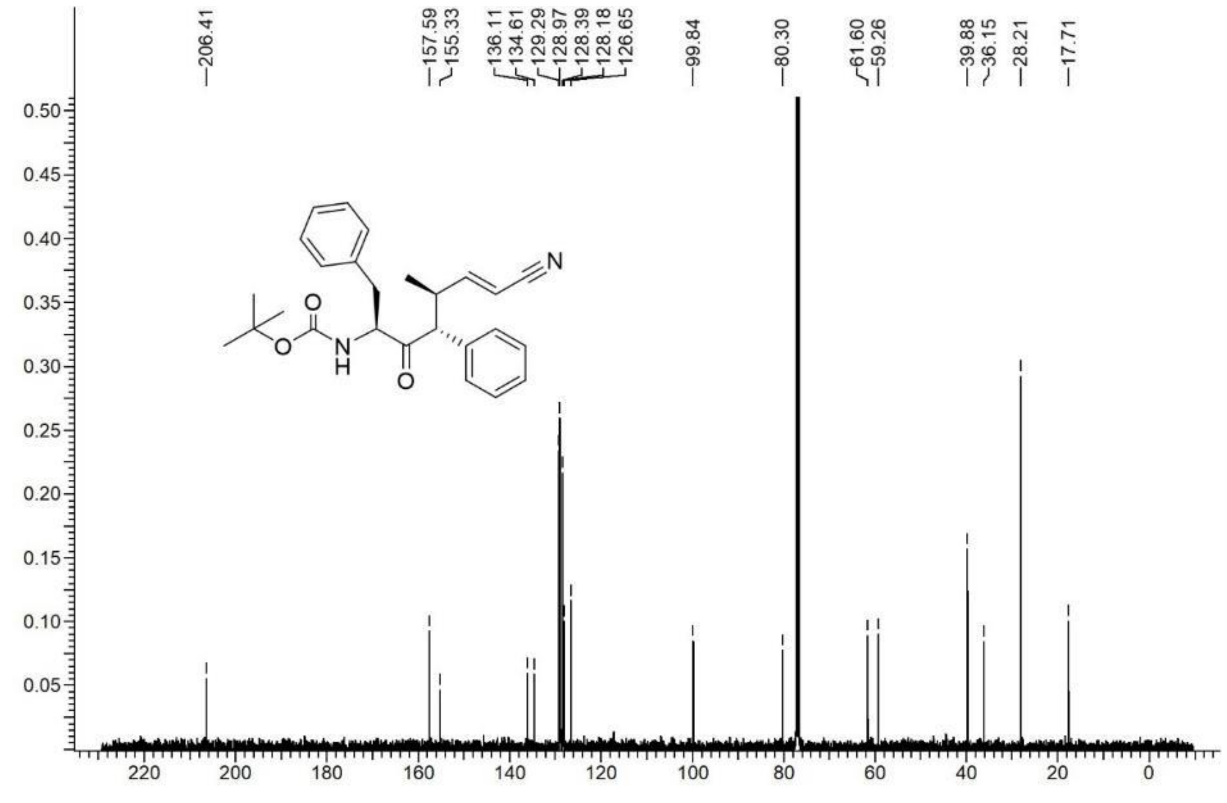

(4*R*,5*R*,7*S*,*Z*)-7-((*tert*-Butoxycarbonyl)amino)-4-methyl-6-oxo-5,8-diphenyloct-2-enenitrile [(4*R*,*Z*)-19a]

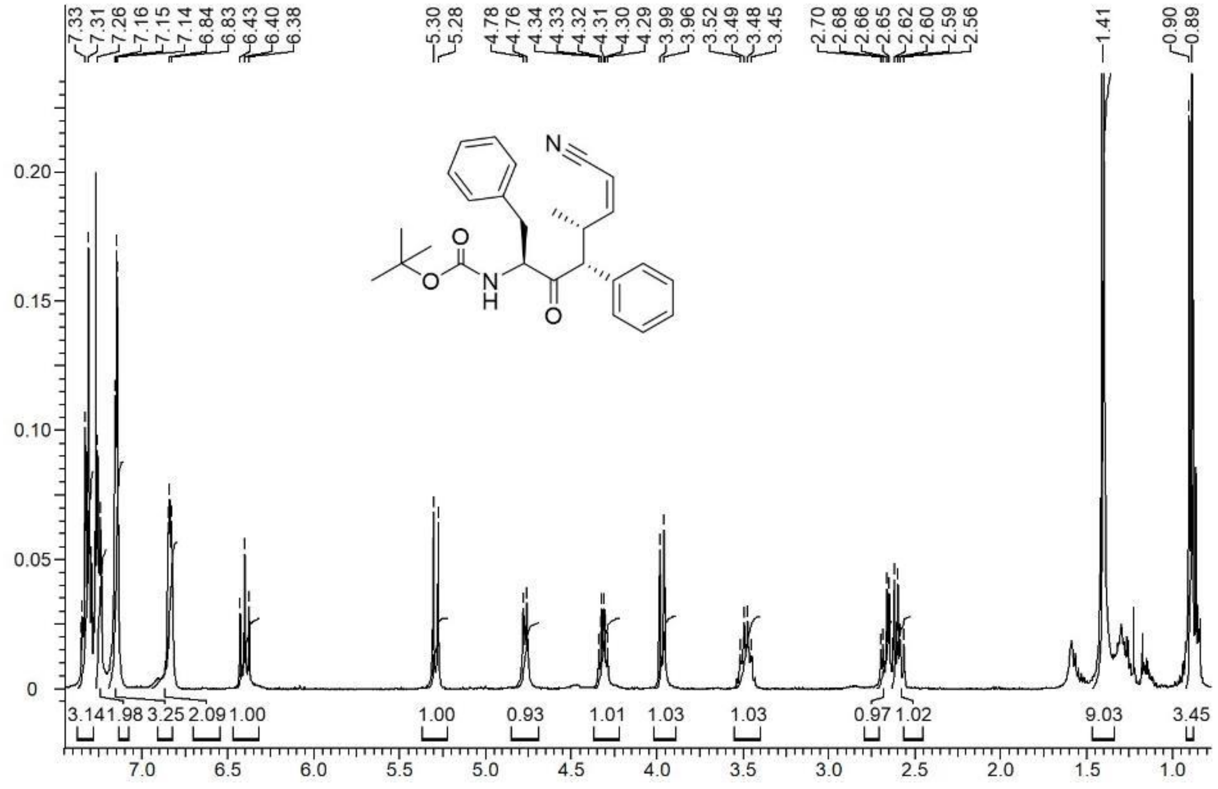

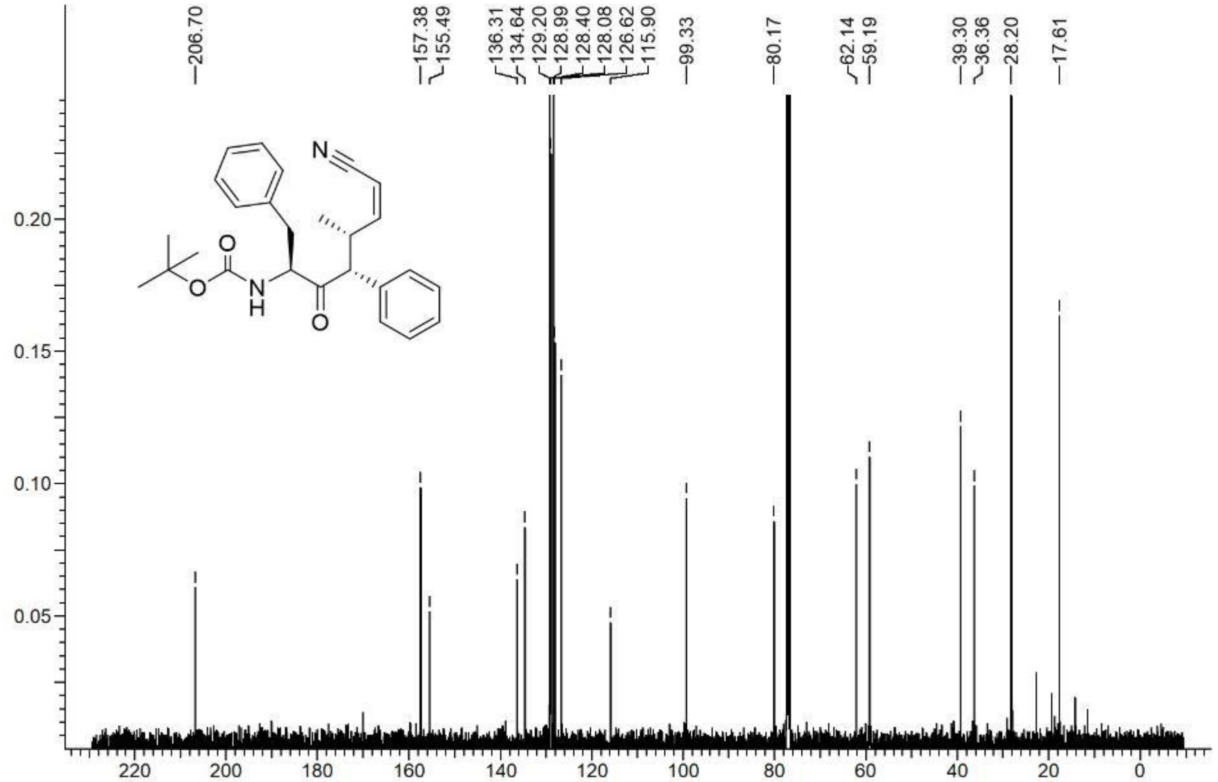

HPLC [(4*R*,*Z*)-19a & (4*S*,*E*)-19a)] – Table 3, entry 2
Column: Reprosil 100 Chiral-NR 8 µm
Eluent: Hexane/iPrOH 97:3 – 93:7, 47 min, 1.0 ml/min, 20°C, 210 nm

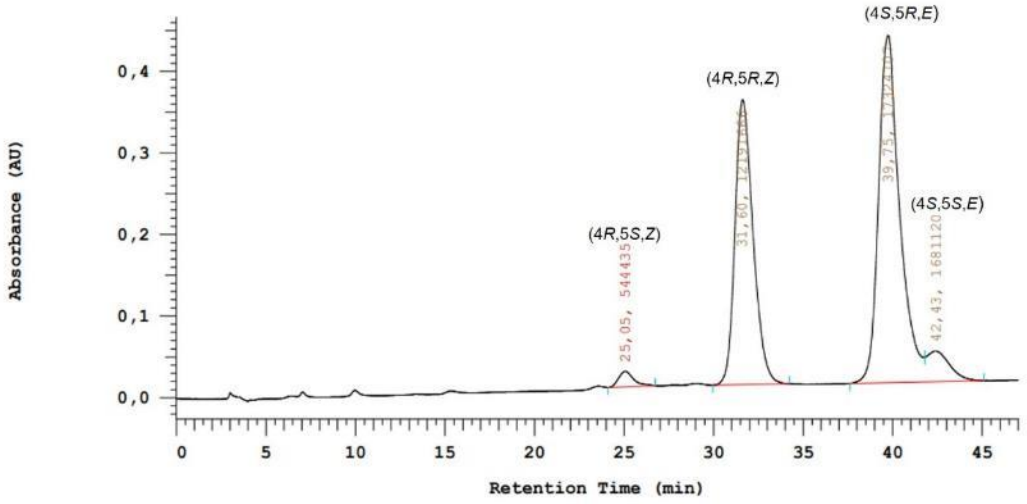

No.	RT	Area	Area %
1	25,05	544435	1,715
2	31,60	12191666	38,409
3	39,75	17324107	54,579
4	42,43	1681120	5,296
		31741328	100,000

(4*R*/*S*,5*R*,7*S*)-7-((*tert*-Butoxycarbonyl)amino)-4-methyl-6-oxo-5,8-diphenyloct-2-enenitrile (19a)

– E/Z-mixture

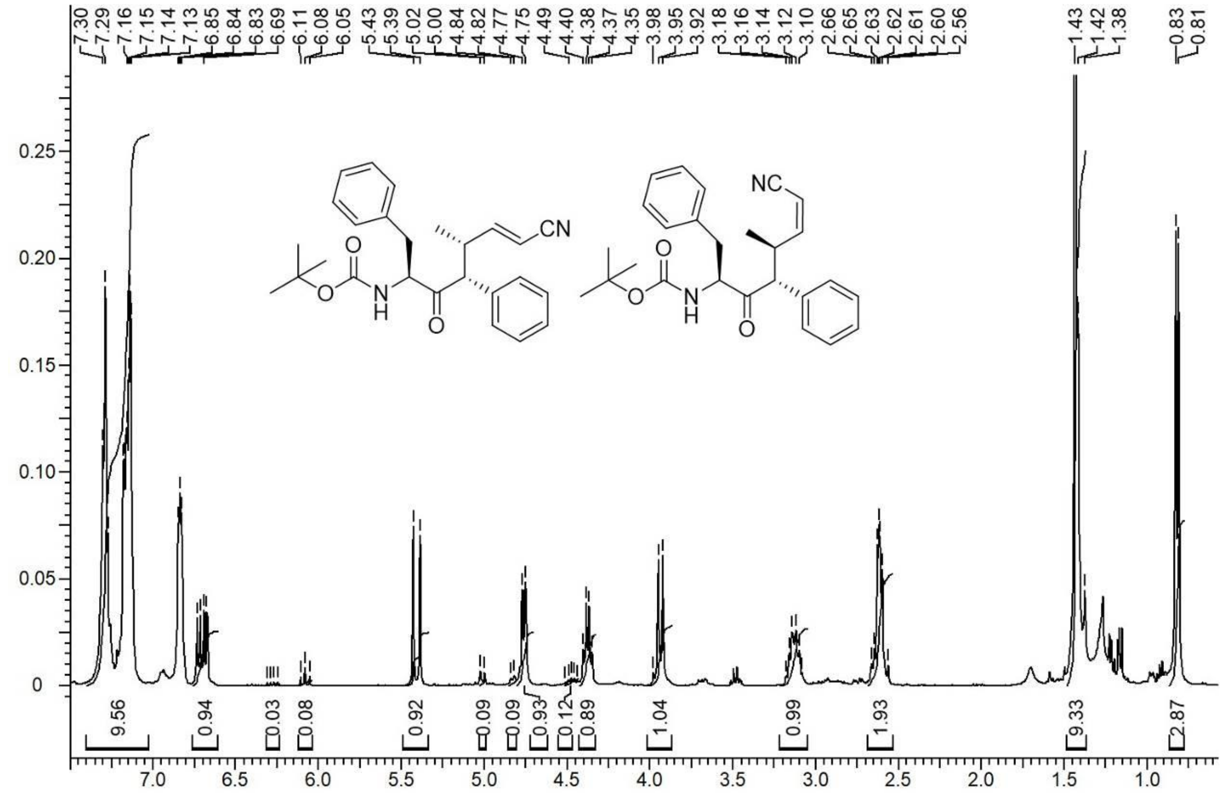

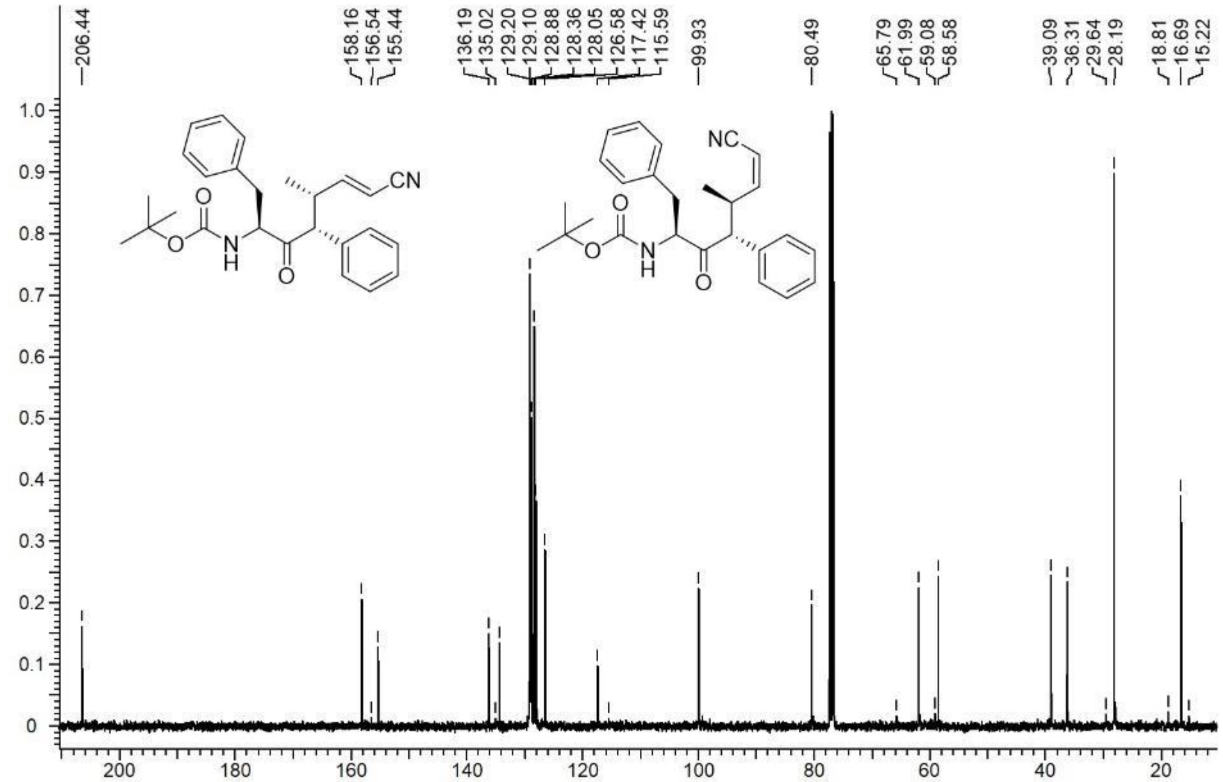

(4*R*,5*R*,7*S*,*E*)-7-((*tert*-Butoxycarbonyl)amino)-4-methyl-6-oxo-5,8-diphenyloct-2-enenitrile [(4*R*,*E*)-19a]

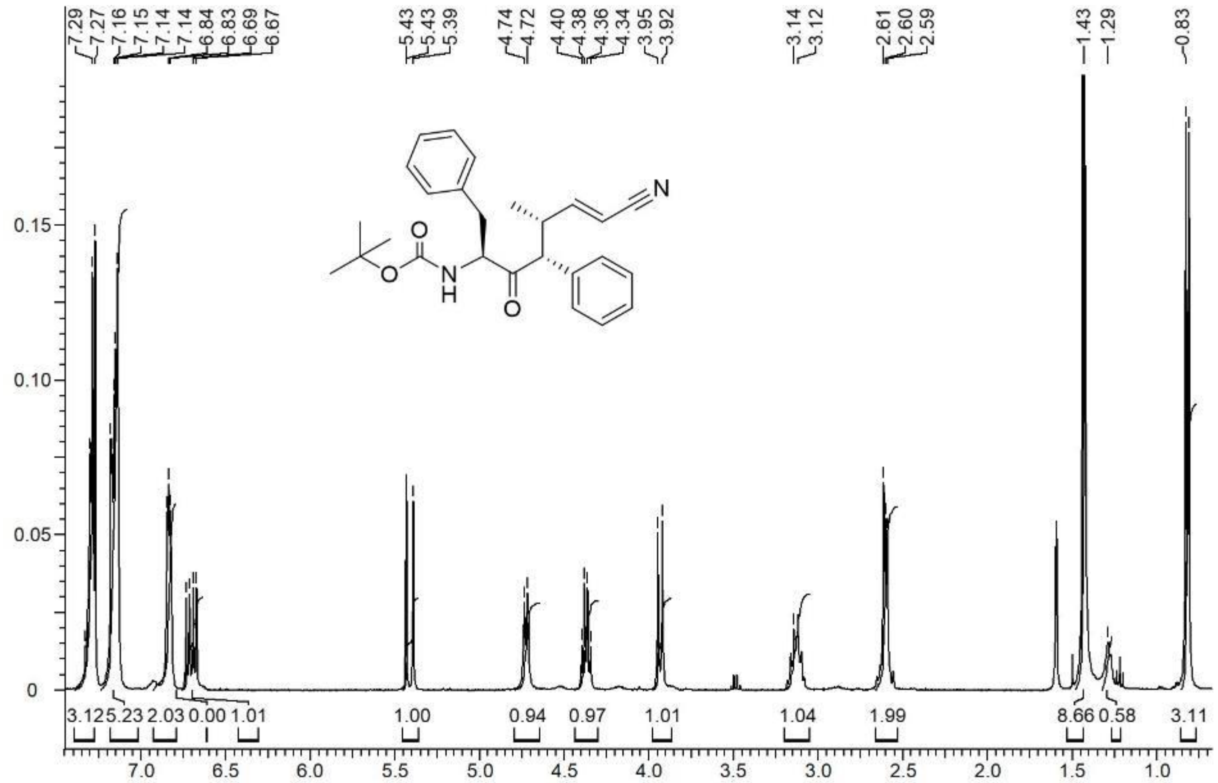

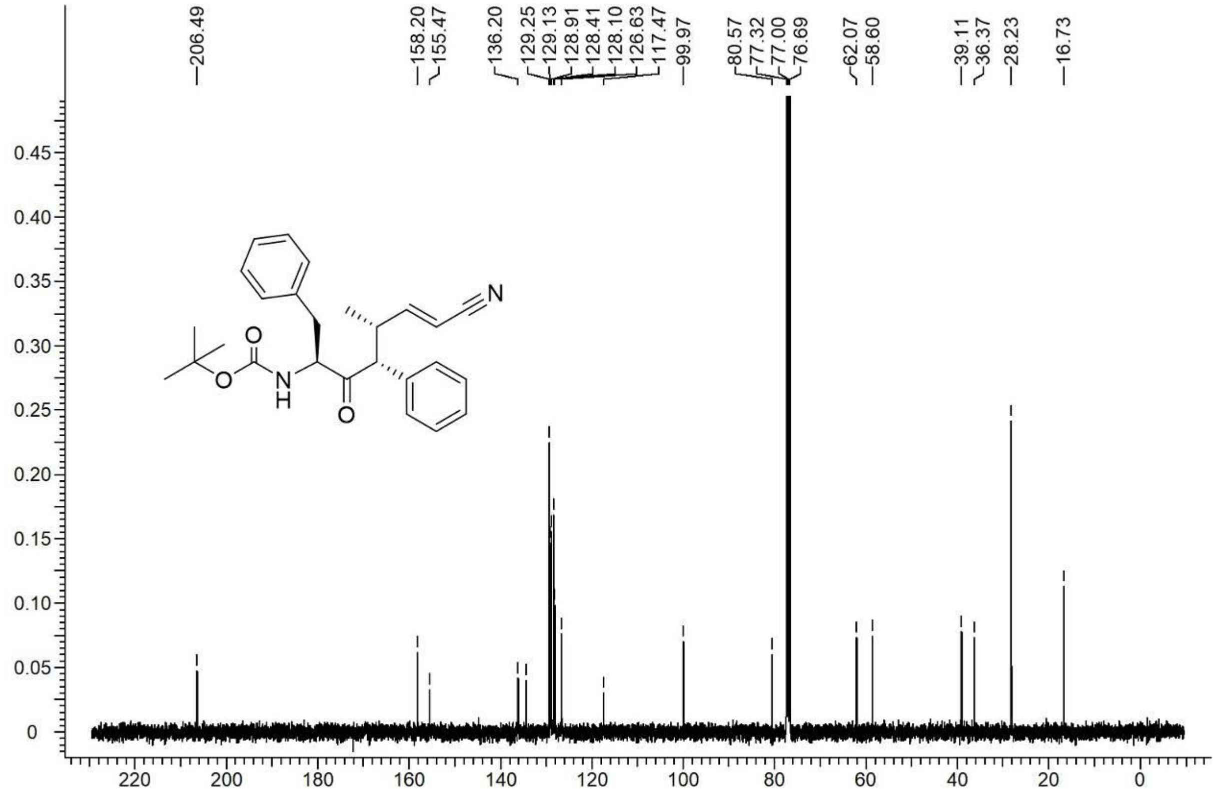

(4*S*,5*R*,7*S*,*Z*)-7-((*tert*-Butoxycarbonyl)amino)-4-methyl-6-oxo-5,8-diphenyloct-2-enenitrile [(4*S*,*Z*)-19a]

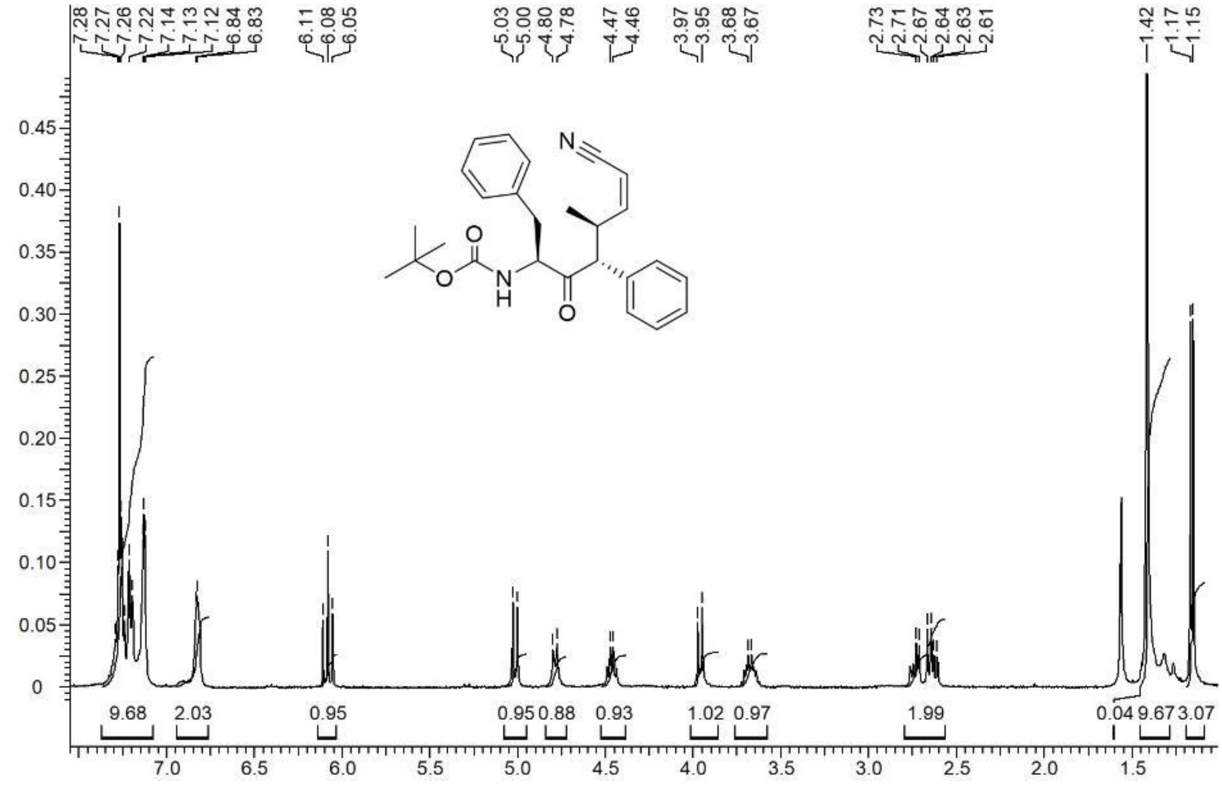

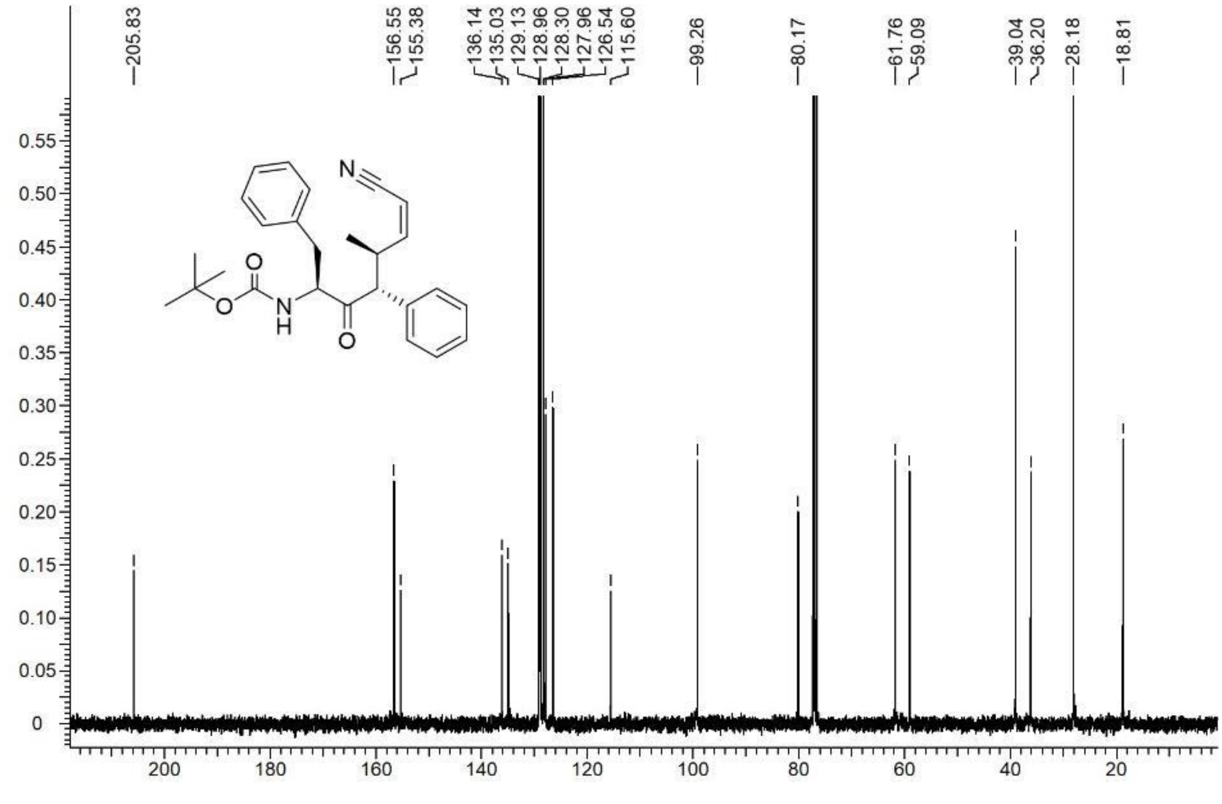

HPLC [(4*R*,*E*)-19a & **(4*S*,*Z*)-19a] –** Table 3, entry 4
Column: Reprosil 100 Chiral-NR 8 µm
Eluent: Hexane/iPrOH 90:10, 1.5 ml/min, 20°C, 220 nm

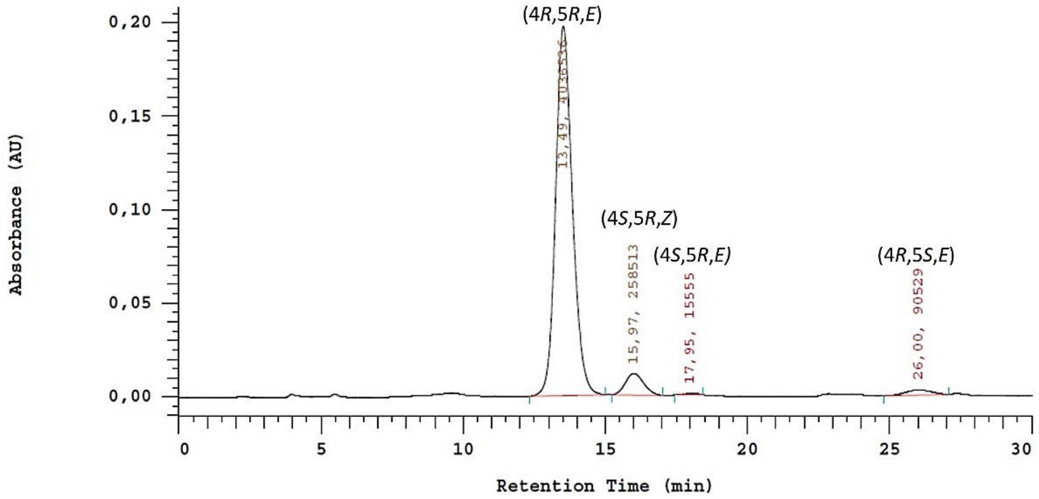

No.	RT	Area	Area %
1	13,49	4036536	91,716
2	15,97	258513	5,874
3	17,95	15555	0,353
4	26,00	90529	2,057
		4401133	100,000

(4*S*/*R*,5*S*,7*S*)-7-((*tert*-Butoxycarbonyl)amino)-4,5-dimethyl-6-oxo-8-phenyloct-2-enenitrile (19b)

– E/Z-mixture

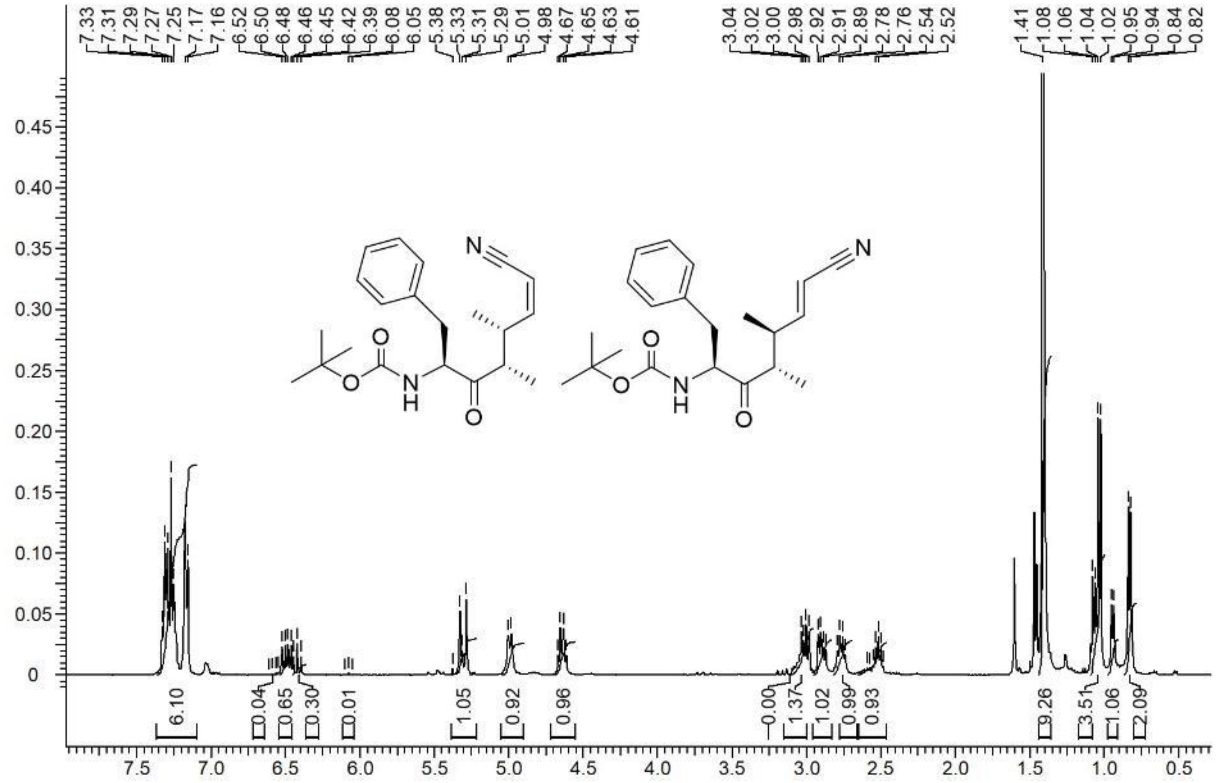

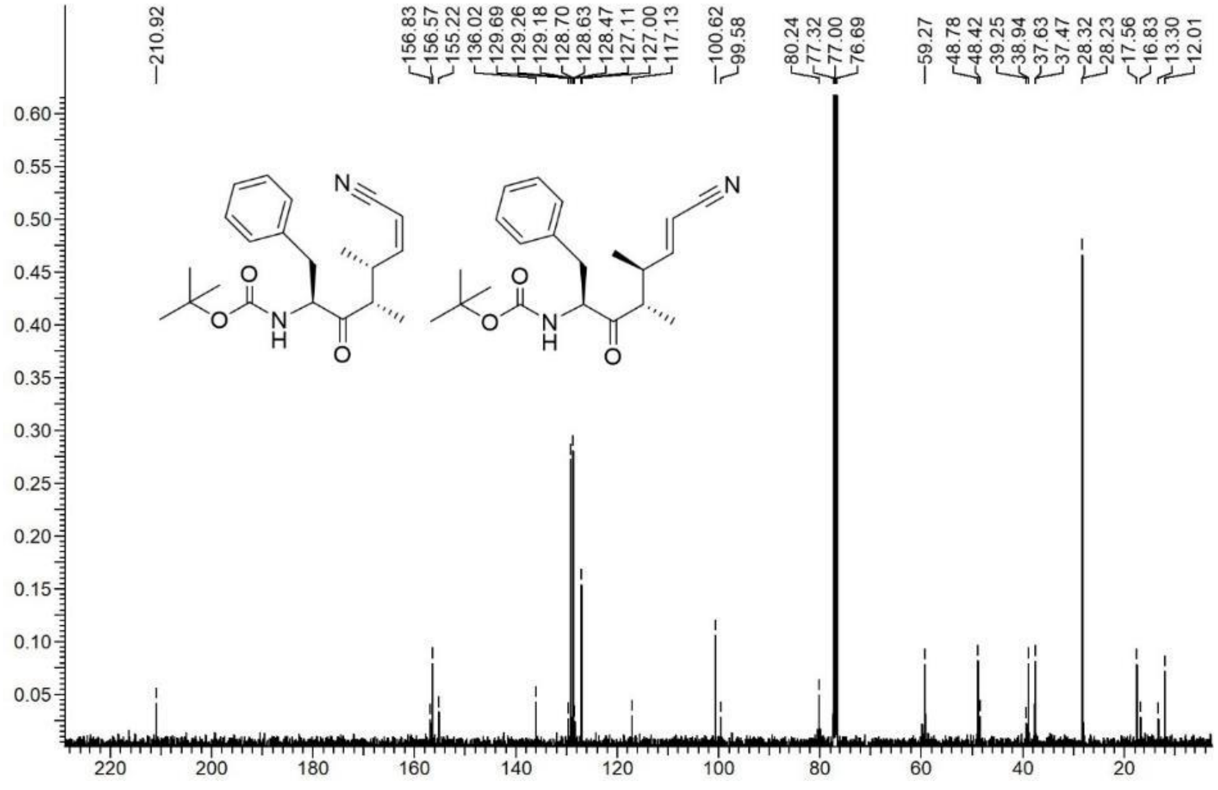

HPLC [crude((4*R*,*Z*)-19b) & **(4*S*,*E*)-19b] –** Table 3, entry 9
Column: Reprosil 100 Chiral-NR 8 µm
Eluent: Hexane/iPrOH 97:3, 1.5 ml/min, 20°C, 220 nm

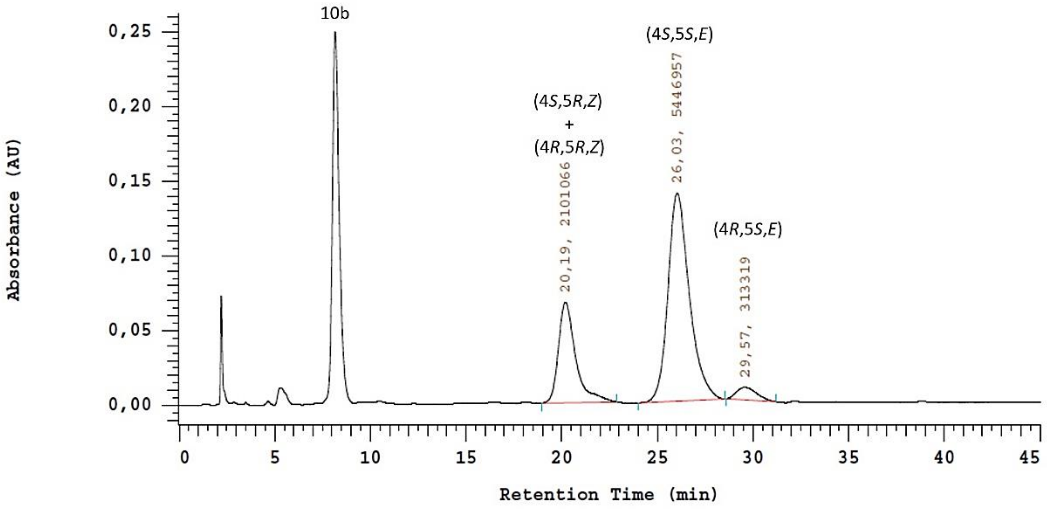

No.	RT	Area	Area %
1	20,19	2101066	26,727
2	26,03	5446957	69,288
3	29,57	313319	3,986
		7861342	100,000

(4*R*/*S*,5*S*,7*S*)-7-((*tert*-Butoxycarbonyl)amino)-4,5-dimethyl-6-oxo-8-phenyloct-2-enenitrile (19b)

– E/Z-mixture

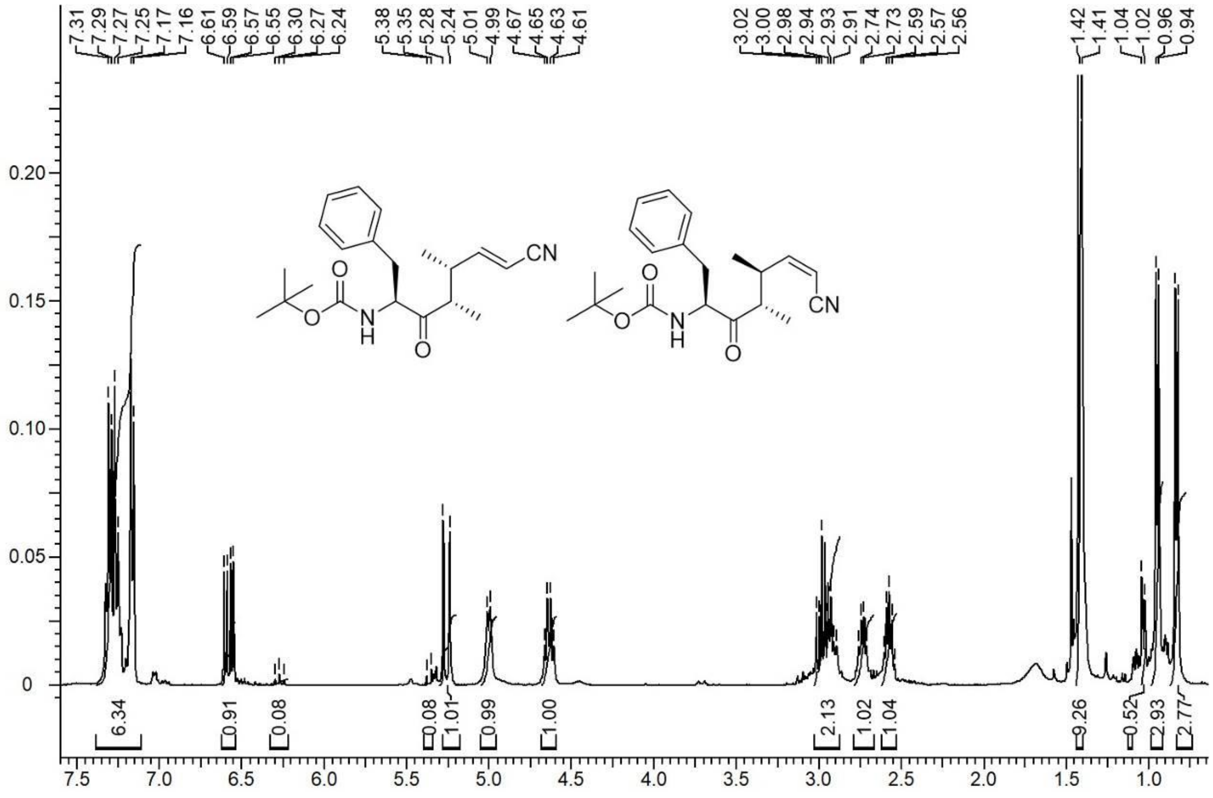

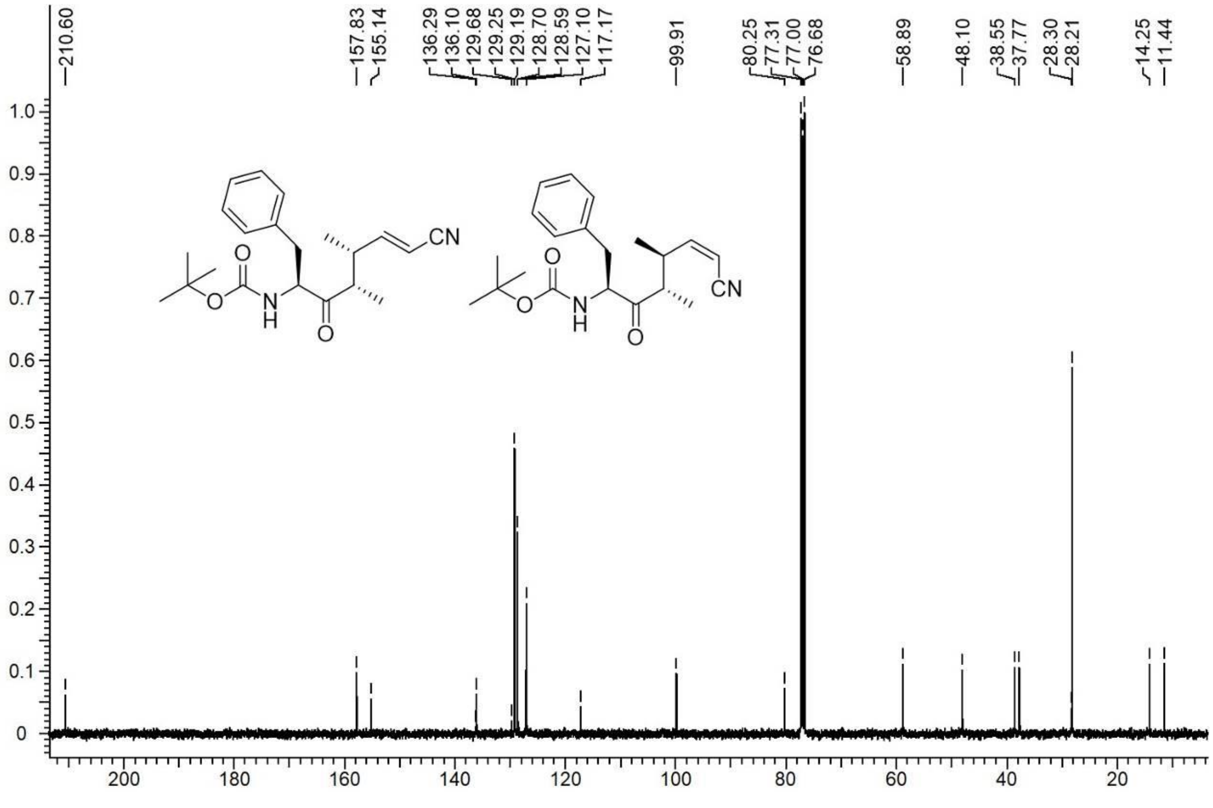

(4*R*,5*S*,7*S*,*E*)-7-((*tert*-Butoxycarbonyl)amino)-4,5-dimethyl-6-oxo-8-phenyloct-2-enenitrile [(4*R*,*E*)-19b]

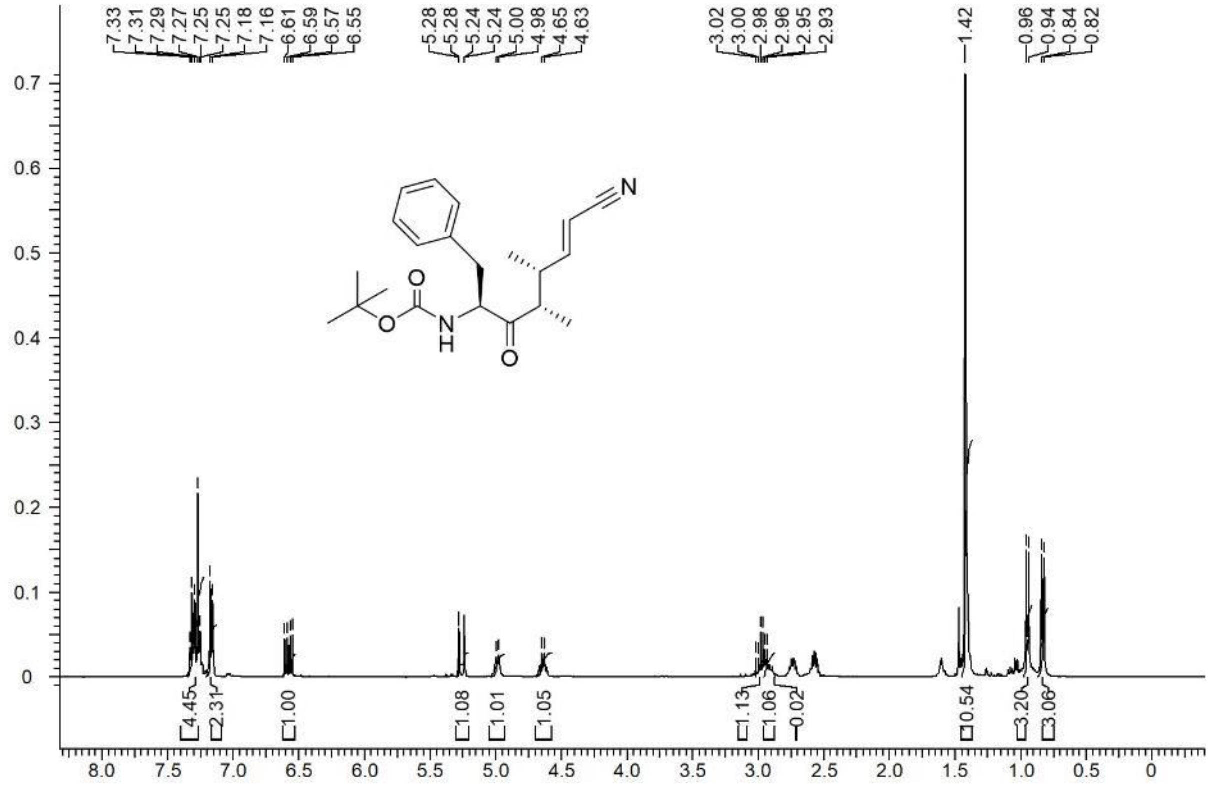

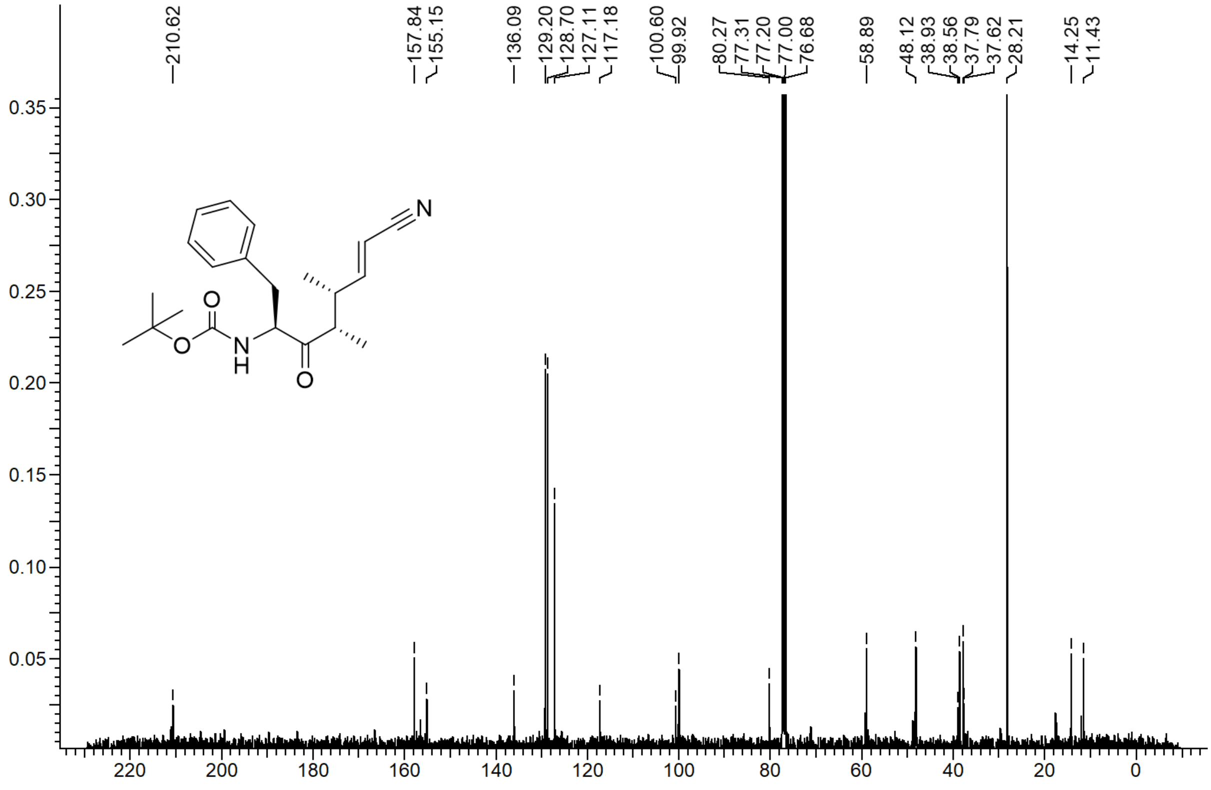

(4*S*,5*S*,7*S*,*Z*)-7-((*tert*-Butoxycarbonyl)amino)-4,5-dimethyl-6-oxo-8-phenyloct-2-enenitrile [(4*S*,*Z*)-19b]

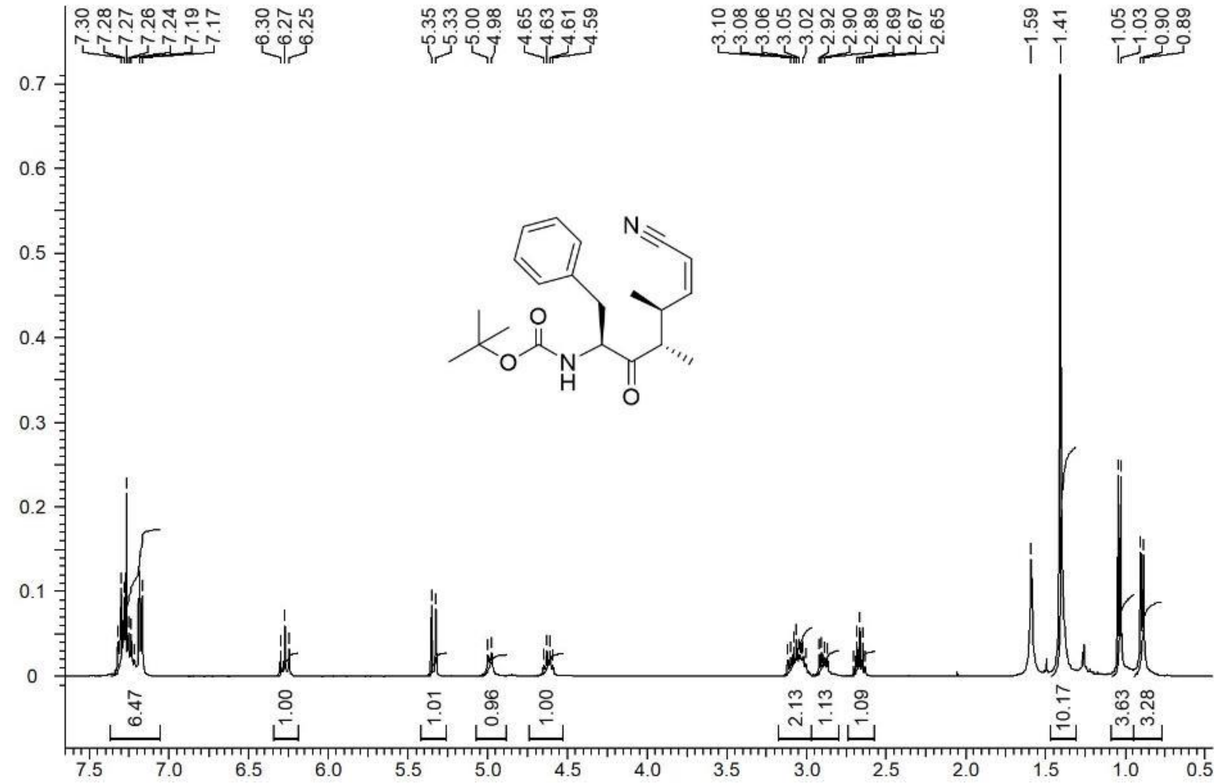

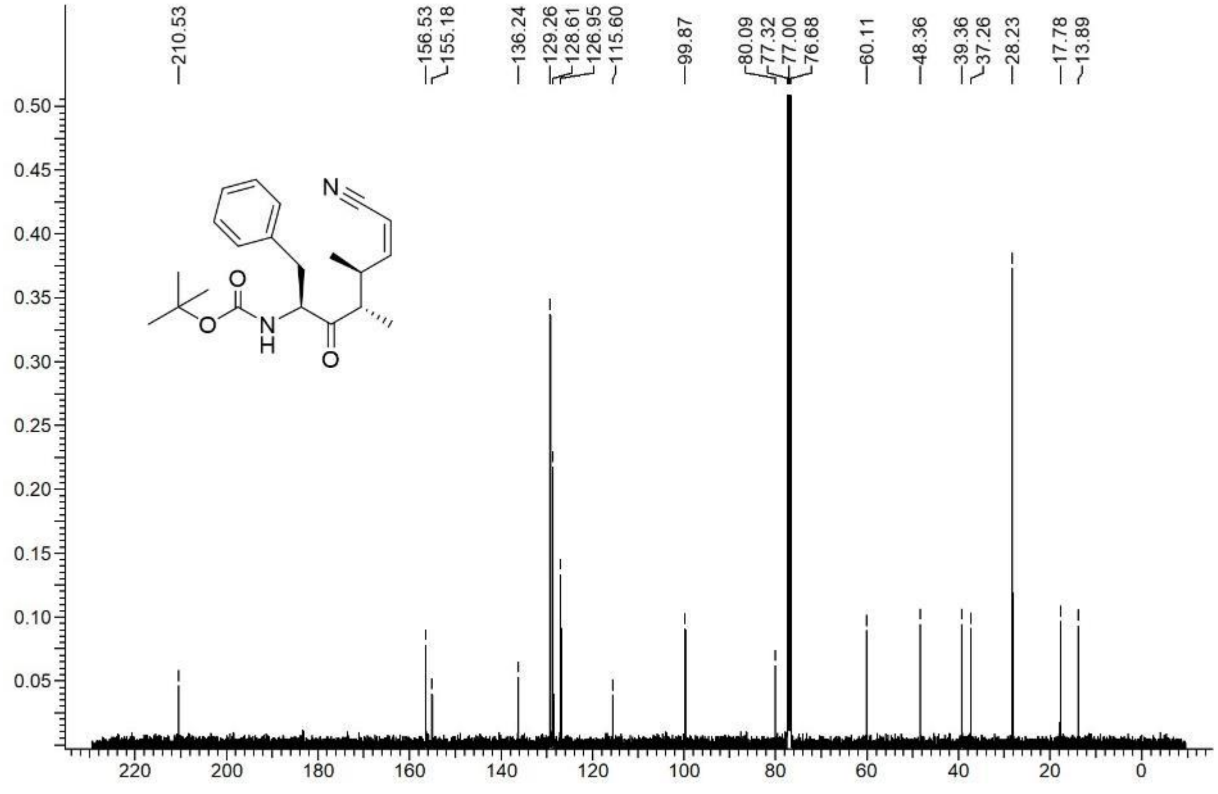

HPLC [(4*R*,*E*)-19b) & (4*S*,*Z*)-19b] – Table 3, entry 10
Column: Chiracel OD-H 5 µm
Eluent: Hexane/iPrOH 95:5, 1.0 ml/min, 20°C, 220 nm

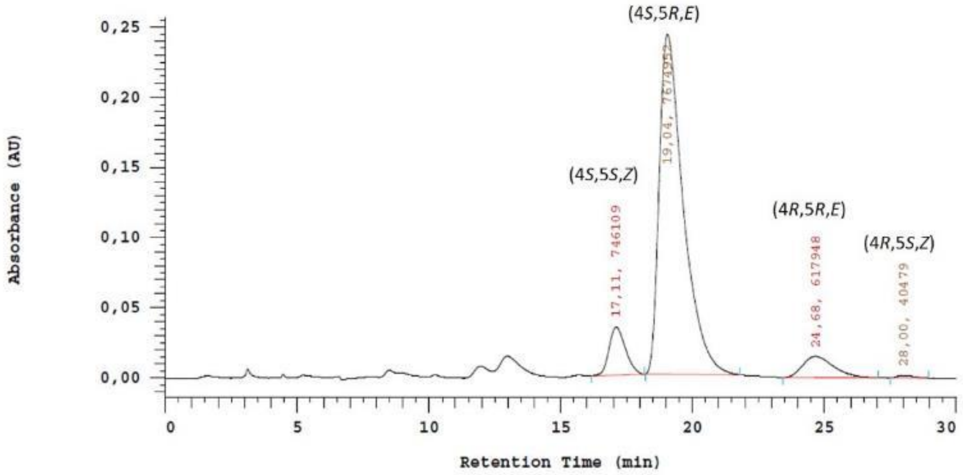

No.	RT	Area	Area %
1	17,11	746109	8,218
2	19,04	7674952	84,531
3	24,68	617948	6,806
4	28,00	40479	0,446
		9079488	100,000

(4*R*/*S*,5*R*,7*S*)-7-((*tert*-Butoxycarbonyl)amino)-4,8-dimethyl-6-oxo-5-phenylnon-2-enenitrile (20a)

– E/Z-mixture

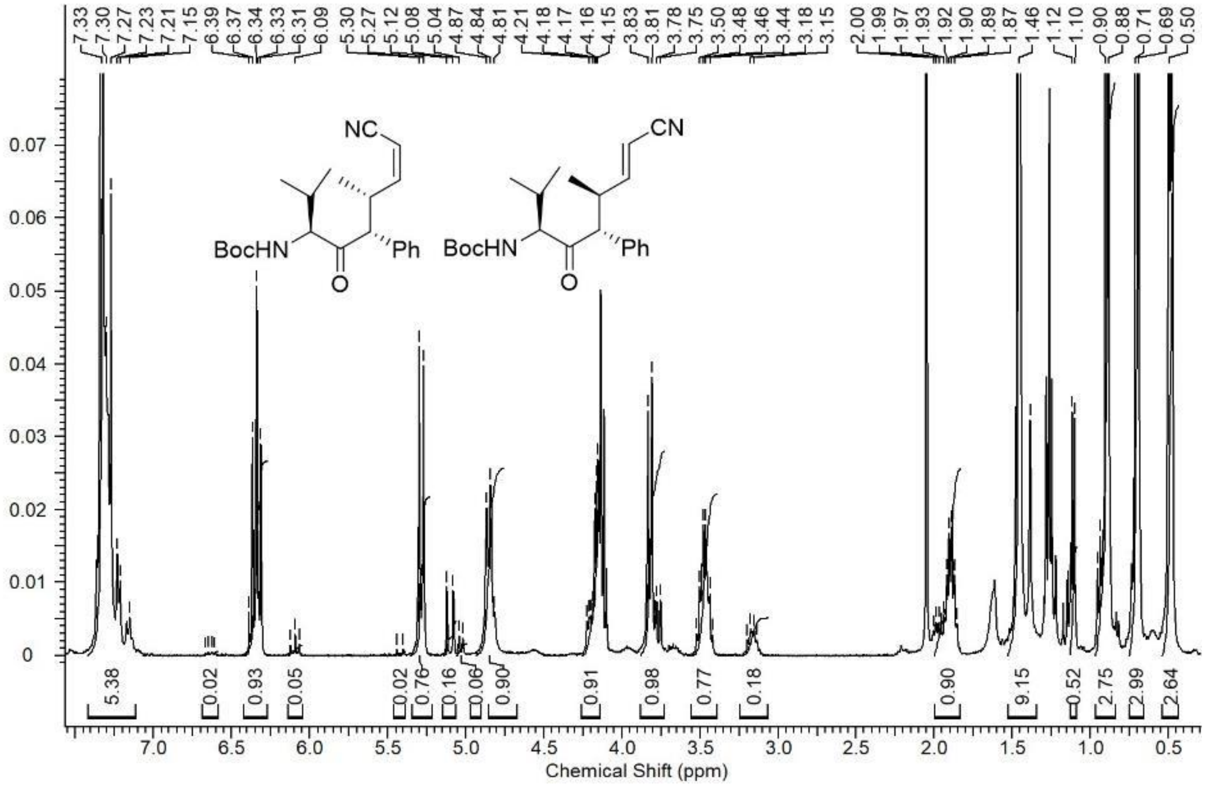

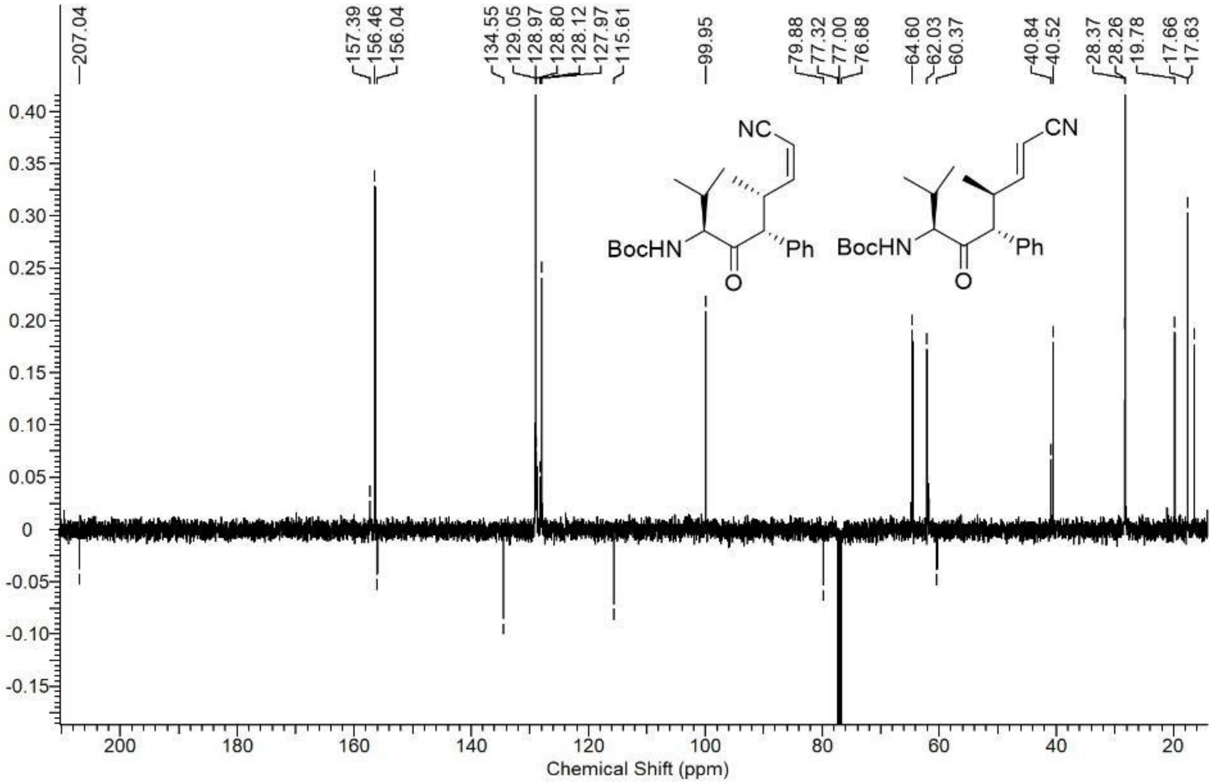

(4*R*,5*R*,7*S*,*Z*)-7-((*tert*-Butoxycarbonyl)amino)-4,8-dimethyl-6-oxo-5-phenylnon-2-enenitrile [(4*R*,*Z*)-20a]

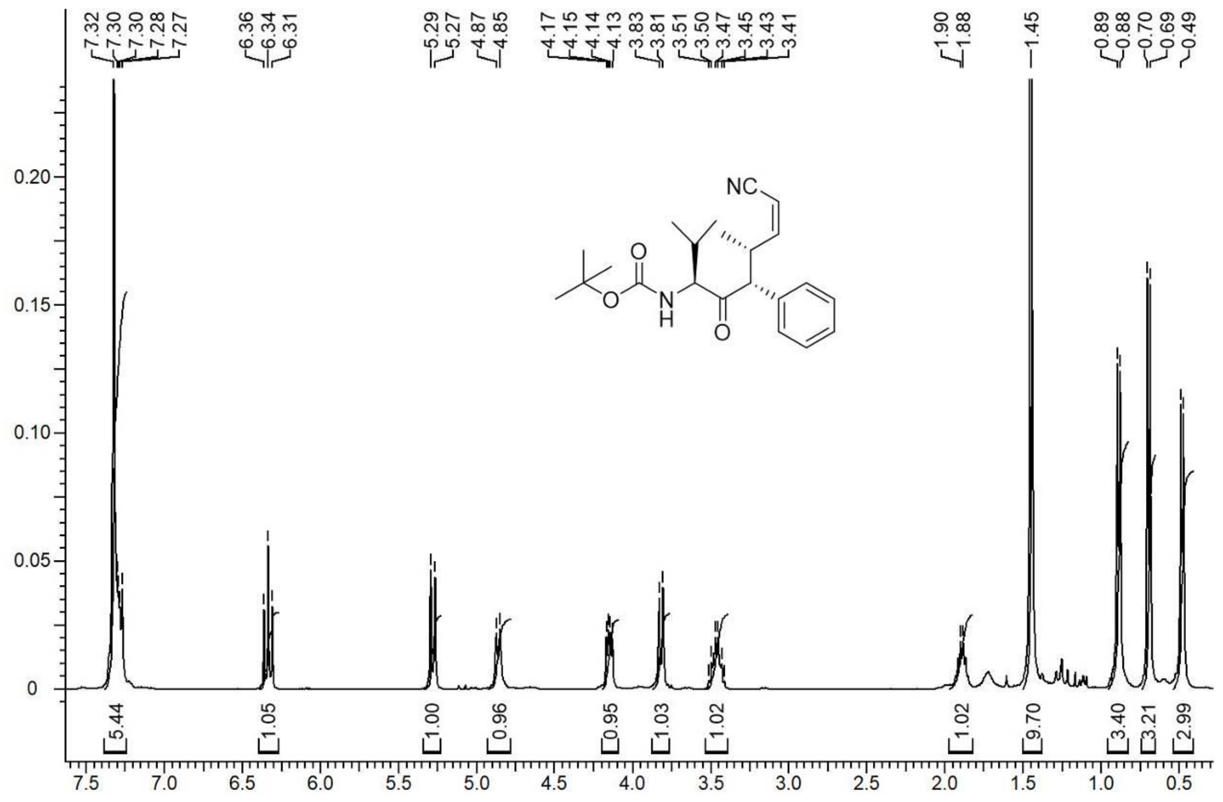

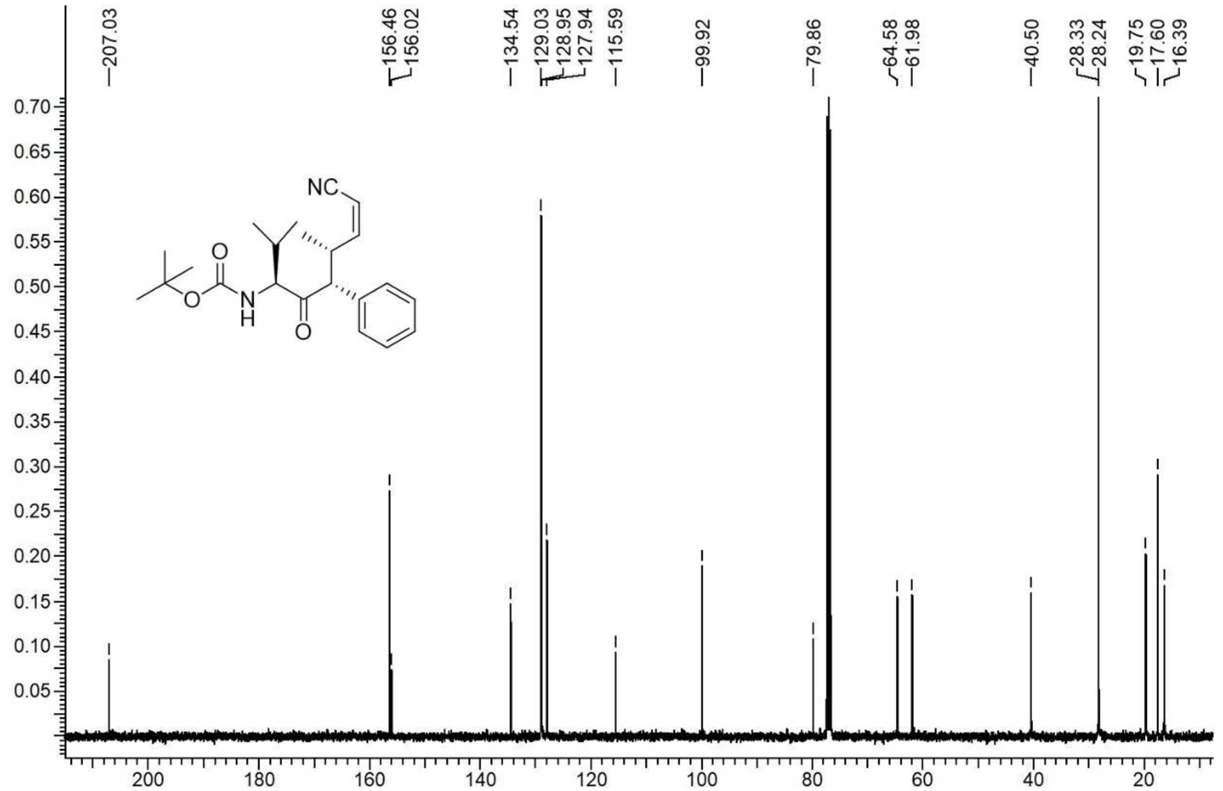

(4*S*,5*R*,7*S*,*E*)-7-((*tert*-Butoxycarbonyl)amino)-4,8-dimethyl-6-oxo-5-phenylnon-2-enenitrile [(4*S*,*E*)-20a]

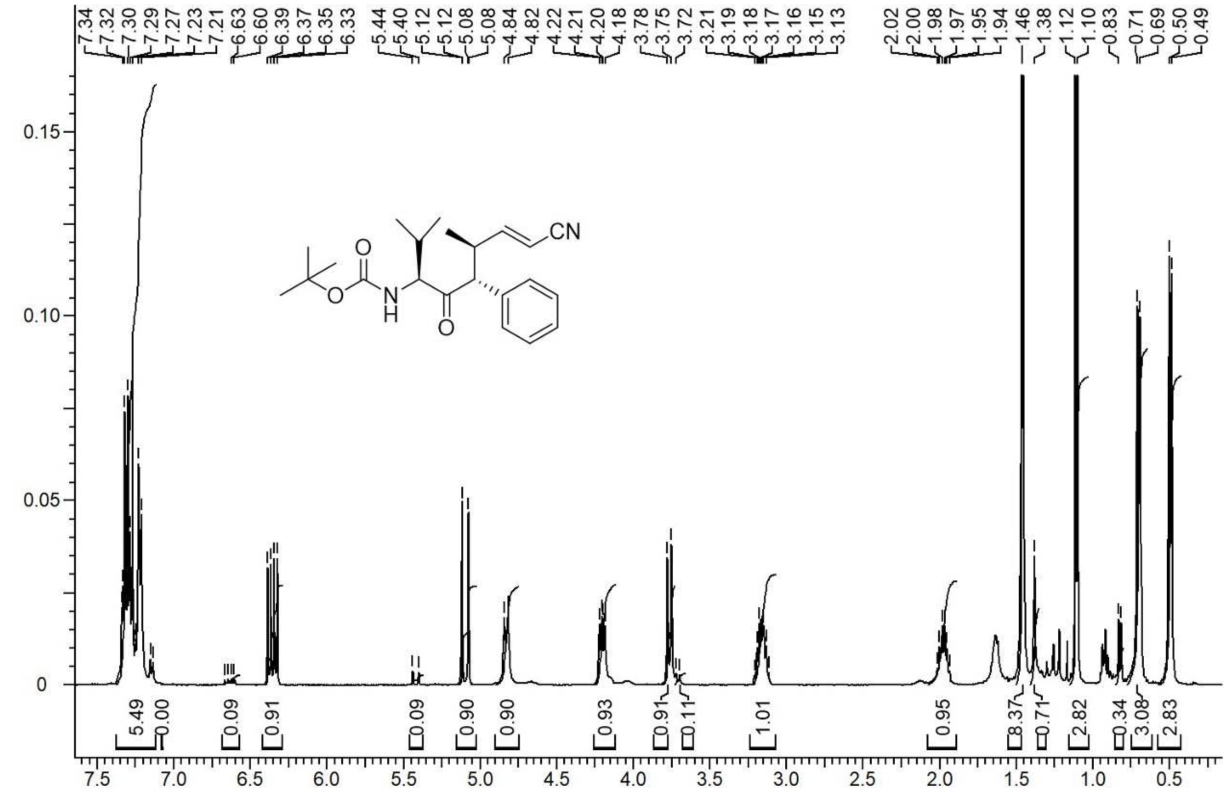

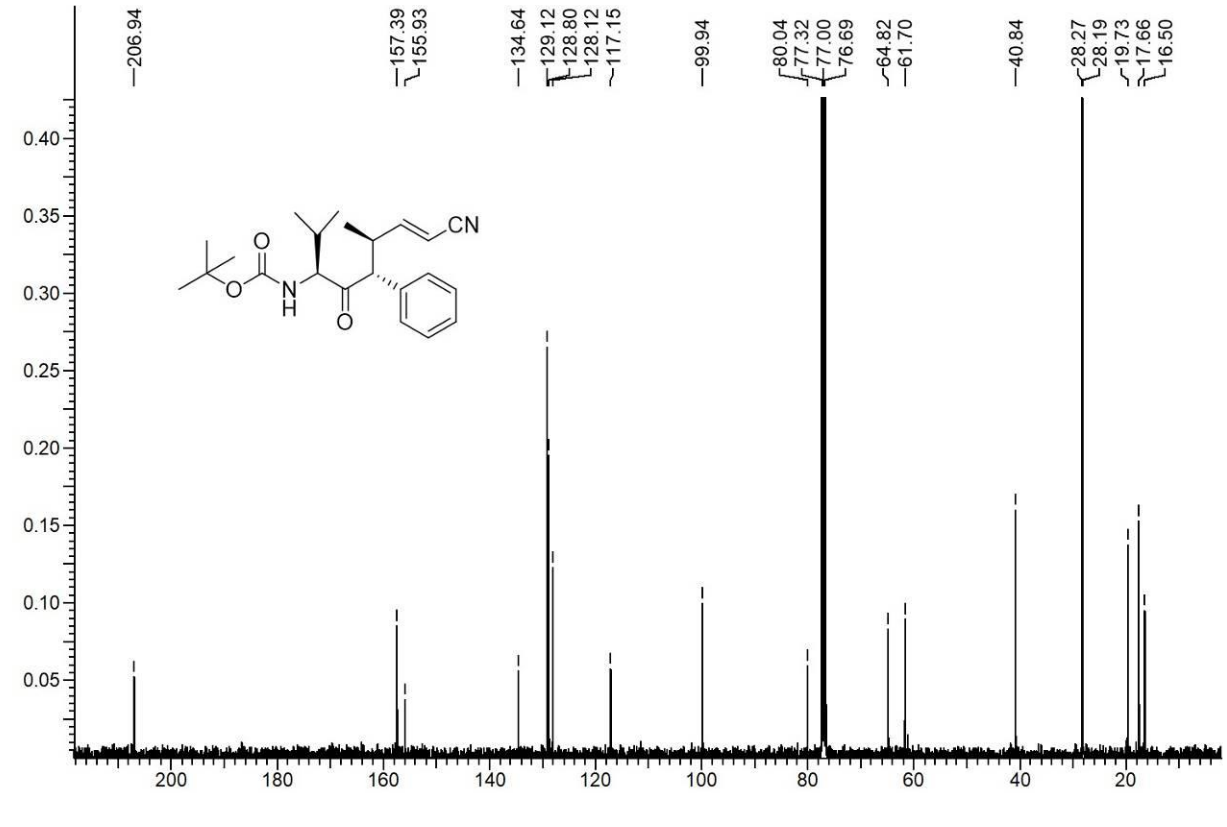

HPLC [(4*S*,*E*)-20a) & **(4*R*,*Z*)-20a] –** Table 3, entry 7
Column: Reprosil 100 Chiral-NR 8 µm
Eluent: Hexane/iPrOH 95:5, 1.5 ml/min, 20°C, 220 nm

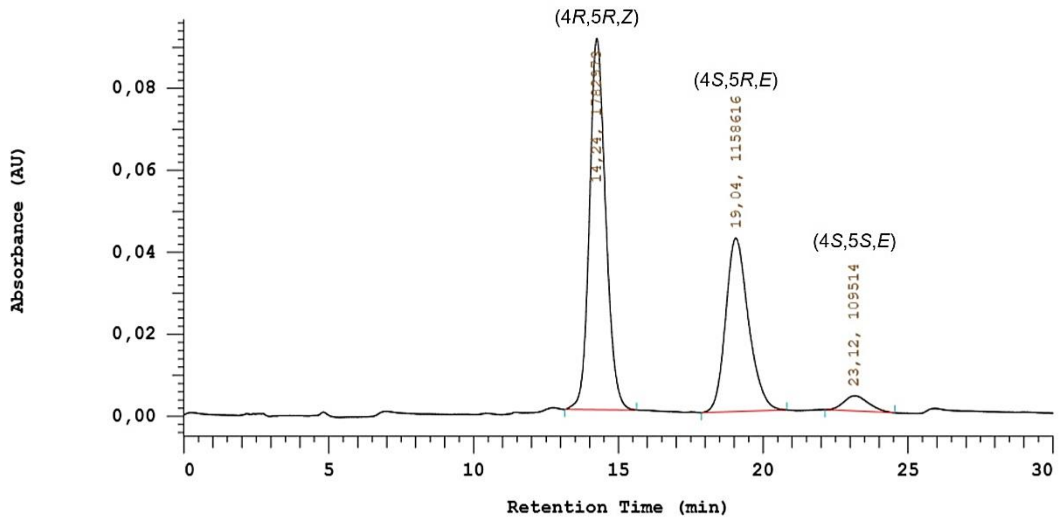

No.	RT	Area	Area %
1	14,24	1782973	58,437
2	19,04	1158616	37,974
3	23,12	109514	3,589
		3051103	100,000

(4*S*/*R*,5*R*,7*S*)-7-((*tert*-Butoxycarbonyl)amino)-4,8-dimethyl-6-oxo-5-phenylnon-2-enenitrile (20a)

– E/Z-mixture

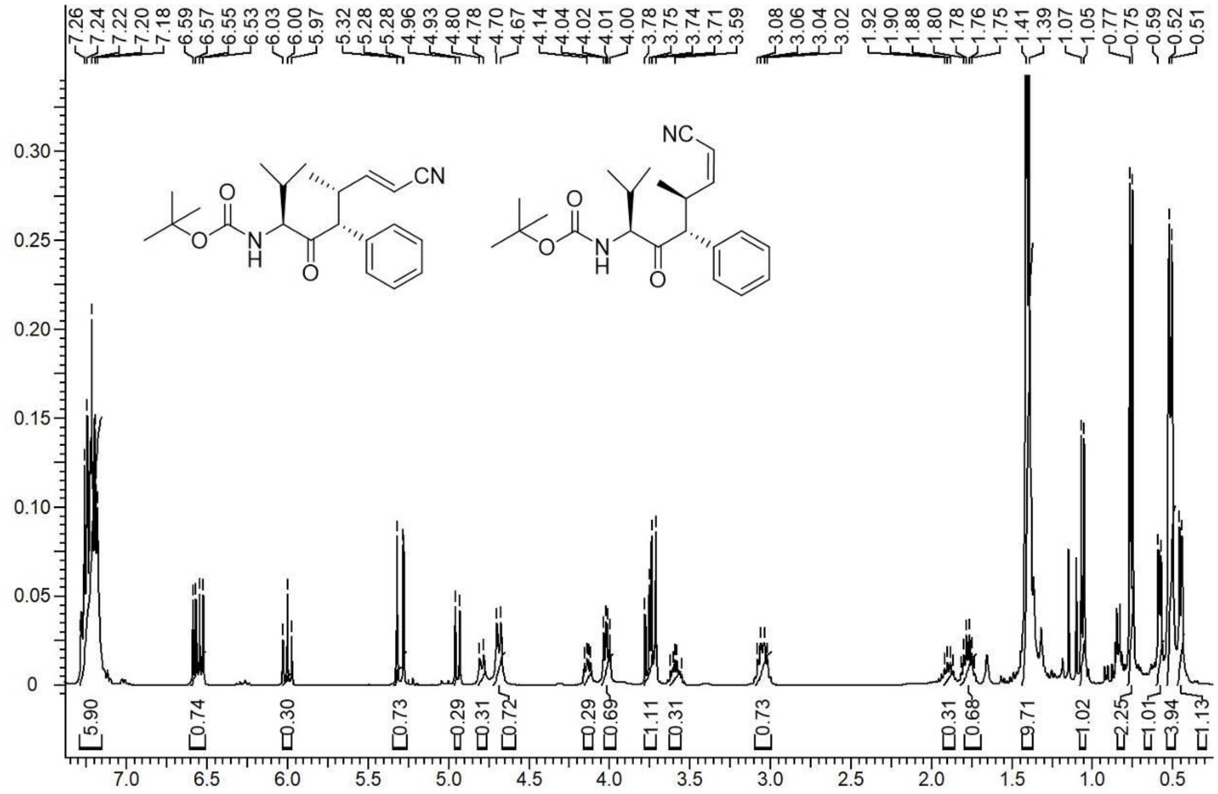

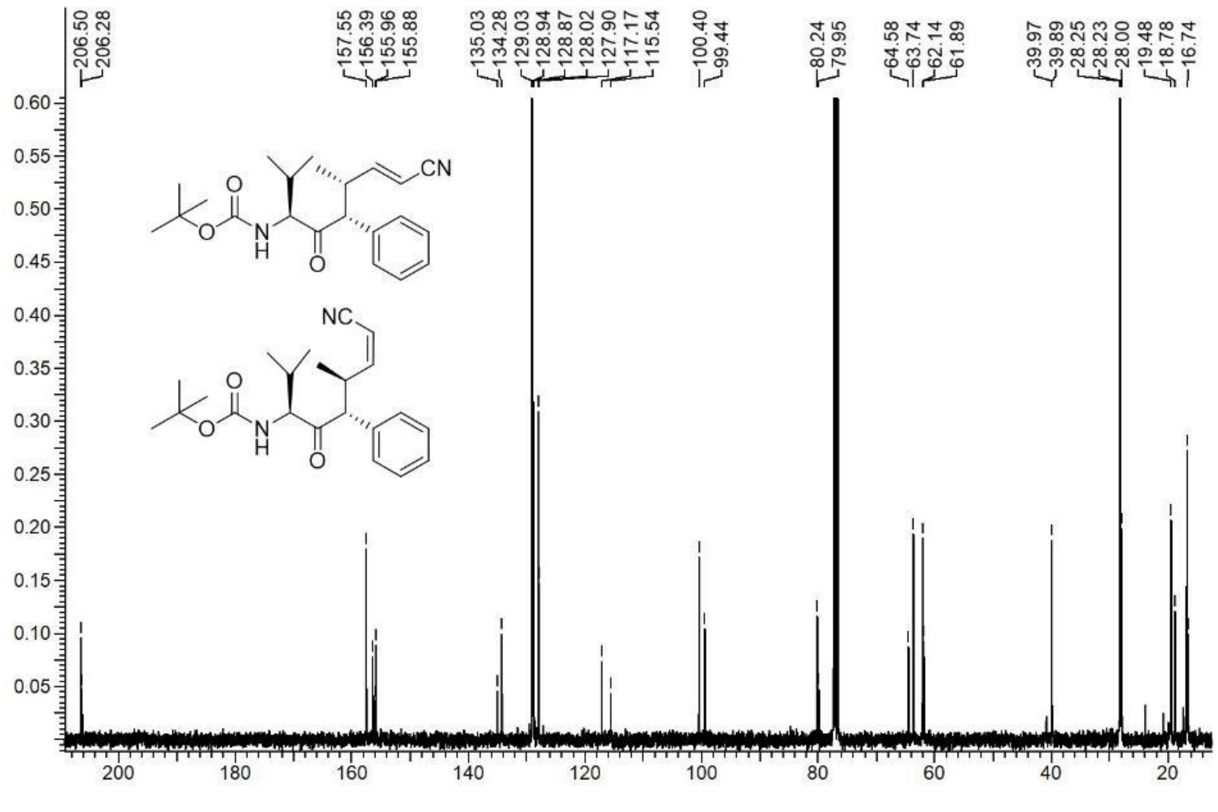

(4*R*,5*R*,7*S*,*E*)-7-((*tert*-Butoxycarbonyl)amino)-4,8-dimethyl-6-oxo-5-phenylnon-2-enenitrile [(4*R*,*E*)-20a]

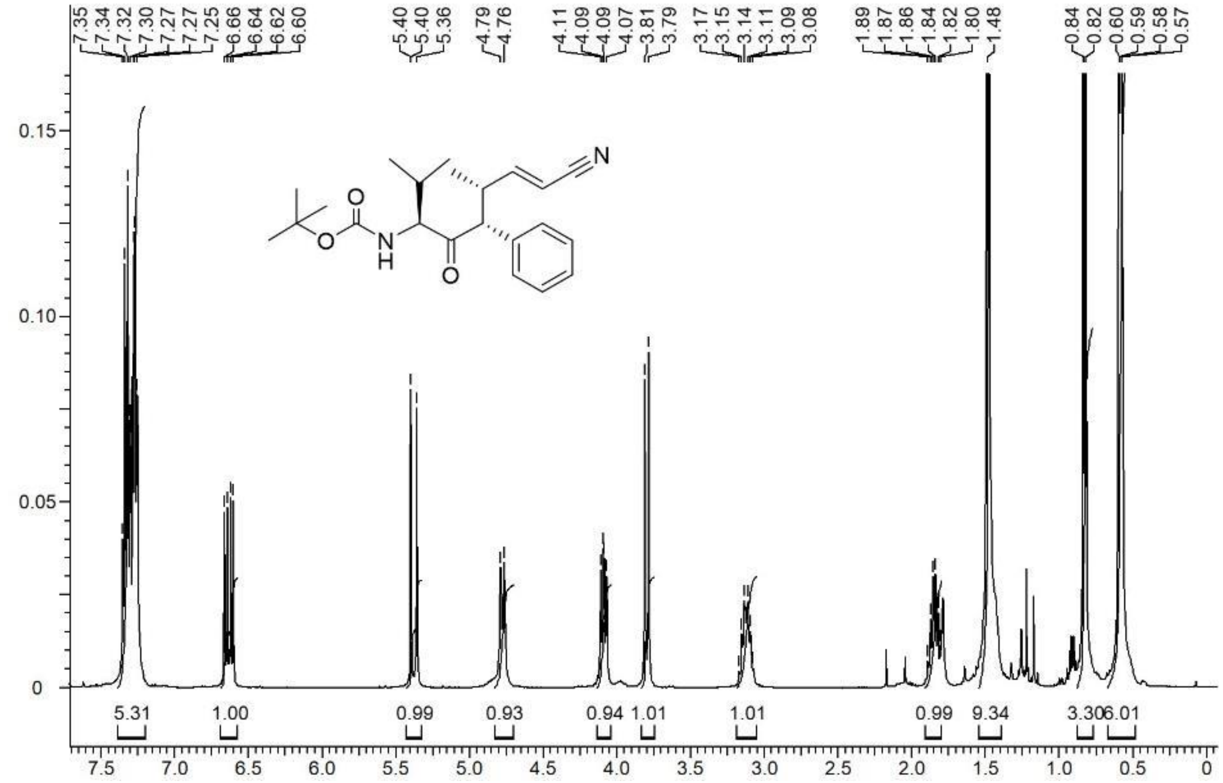

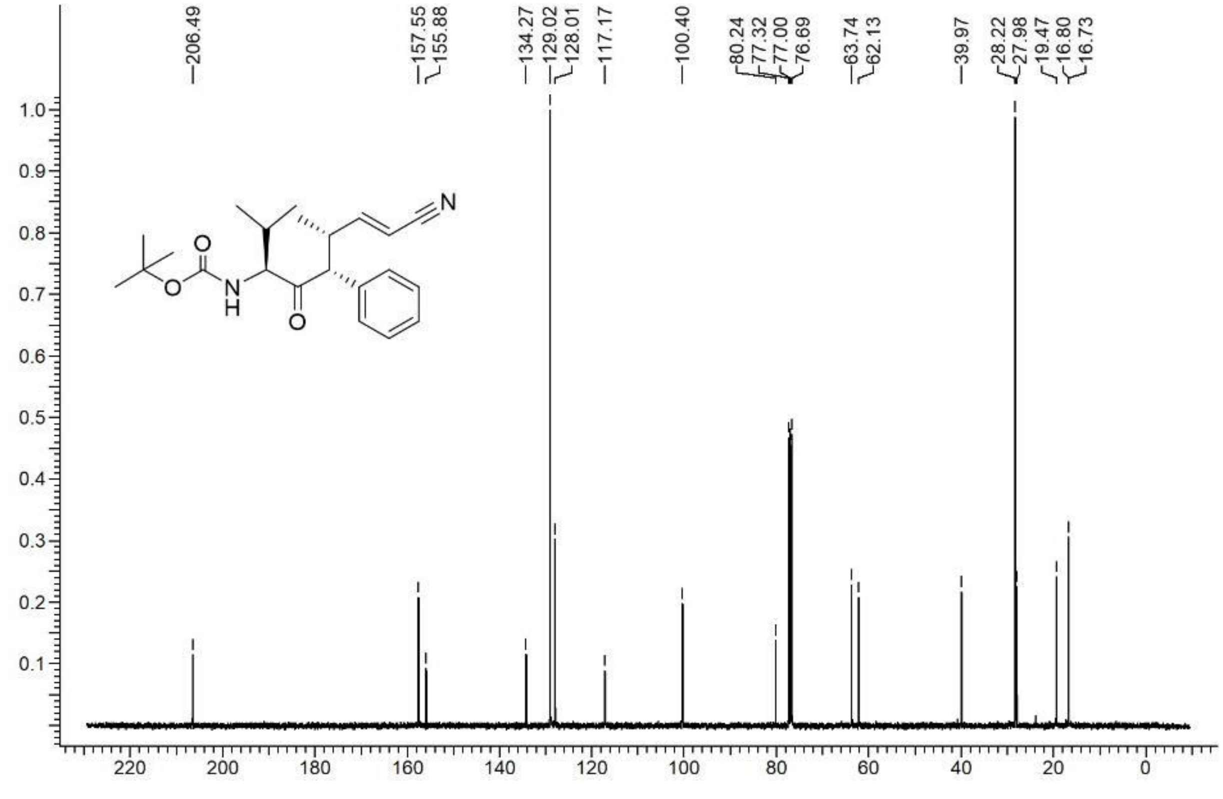

(4*S*,5*R*,7*S*,*Z*)-7-((*tert*-Butoxycarbonyl)amino)-4,8-dimethyl-6-oxo-5-phenylnon-2-enenitrile [(4*S*,*Z*)-20a]

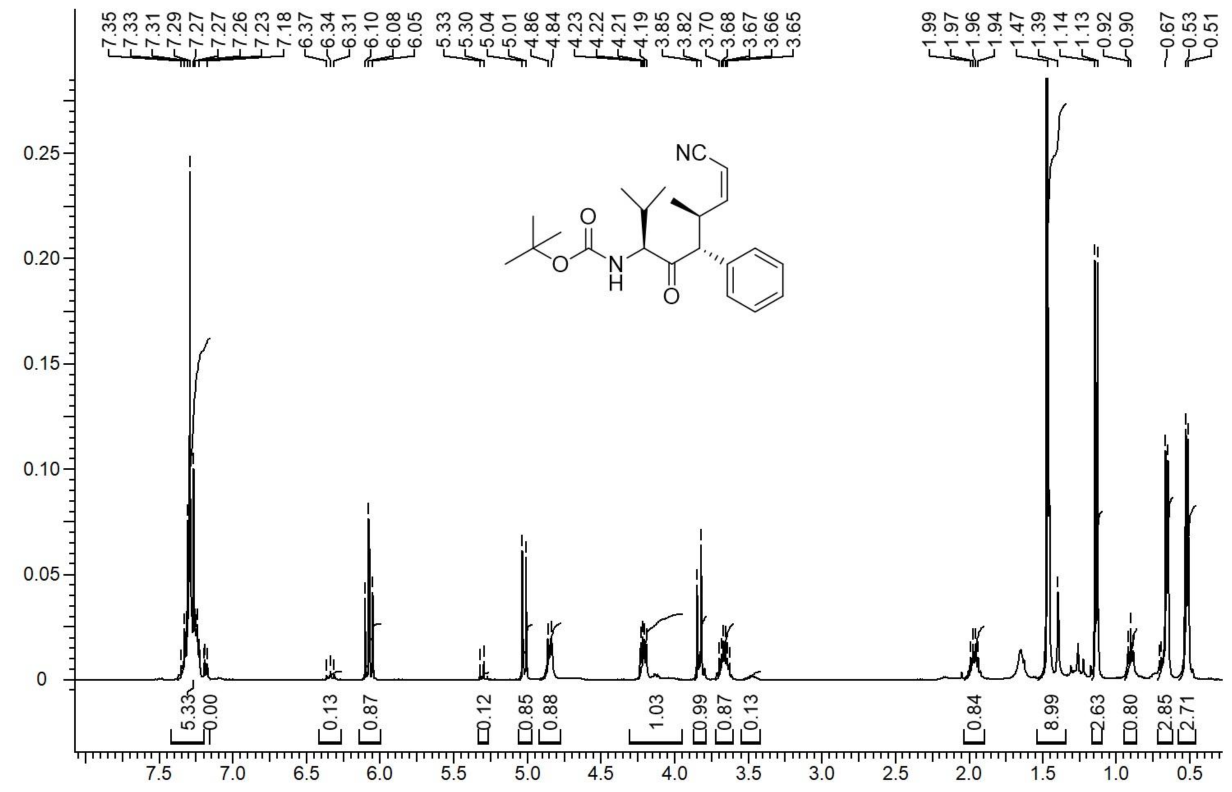

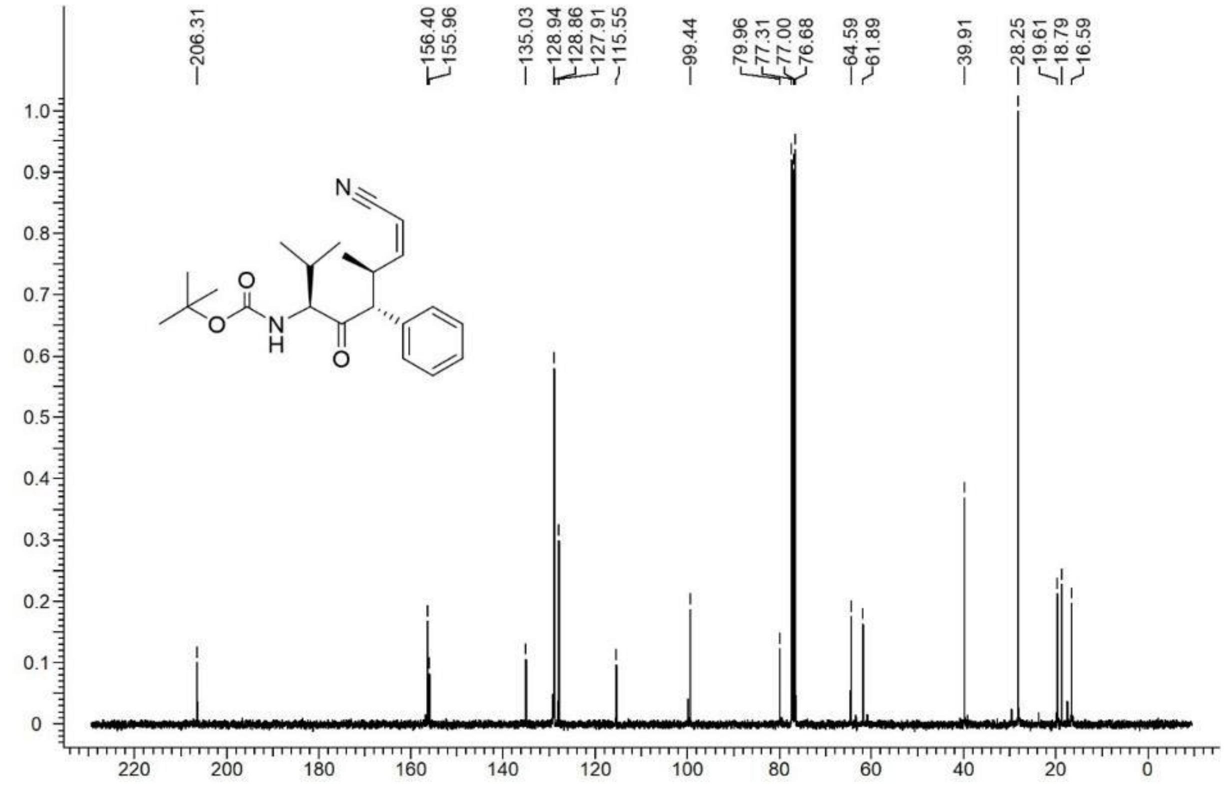

HPLC [(4*R*,*E*)-20a) & **(4*S*,*Z*)-20a] –** Table 3, entry 8
Column: Reprosil 100 Chiral-NR 8 µm
Eluent: Hexane/iPrOH 95:5, 1.5 ml/min, 20°C, 220 nm

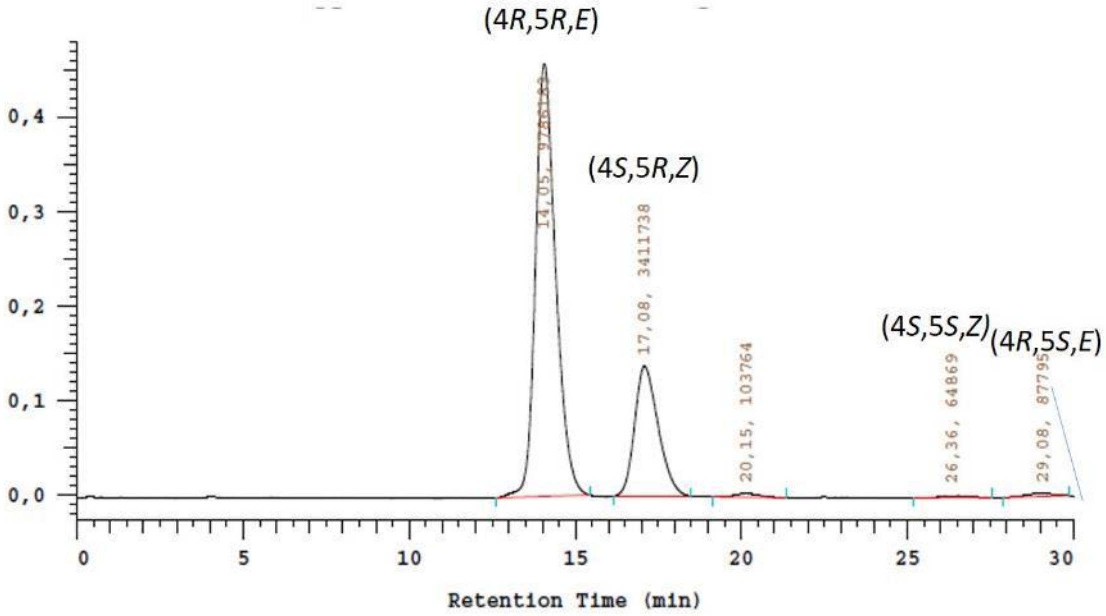

No.	RT	Area	Area %
1	14,05	9786183	72,736
2	17,08	3411738	25,358
3	20,15	103764	0,771
4	26,36	64869	0,482
5	29,08	87795	0,653
		13454349	100,000

Ethyl 2-[(3*R*,4*R*,5*R*,6*S*)-6-benzyl-5-hydroxy-3-methyl-4-phenylpiperidin-2-yl]acetate (21)
– Diasteromeric mixture 2*R*/2*S*

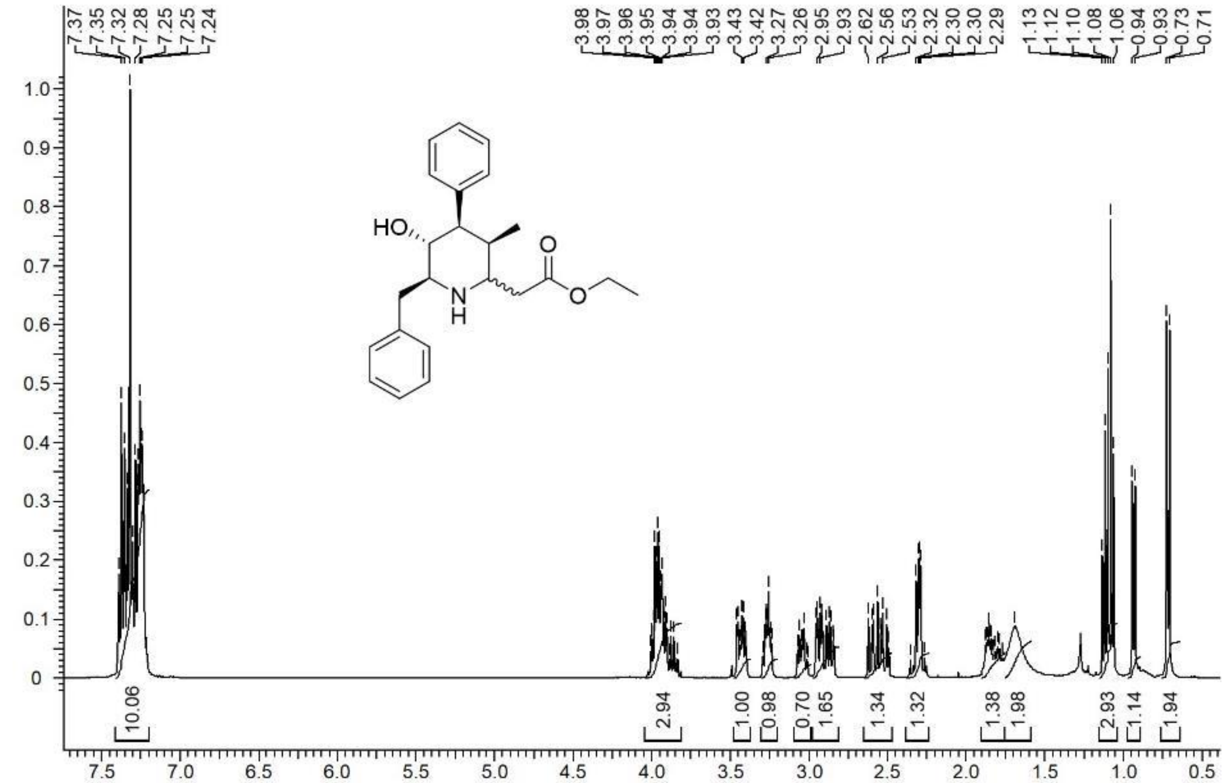

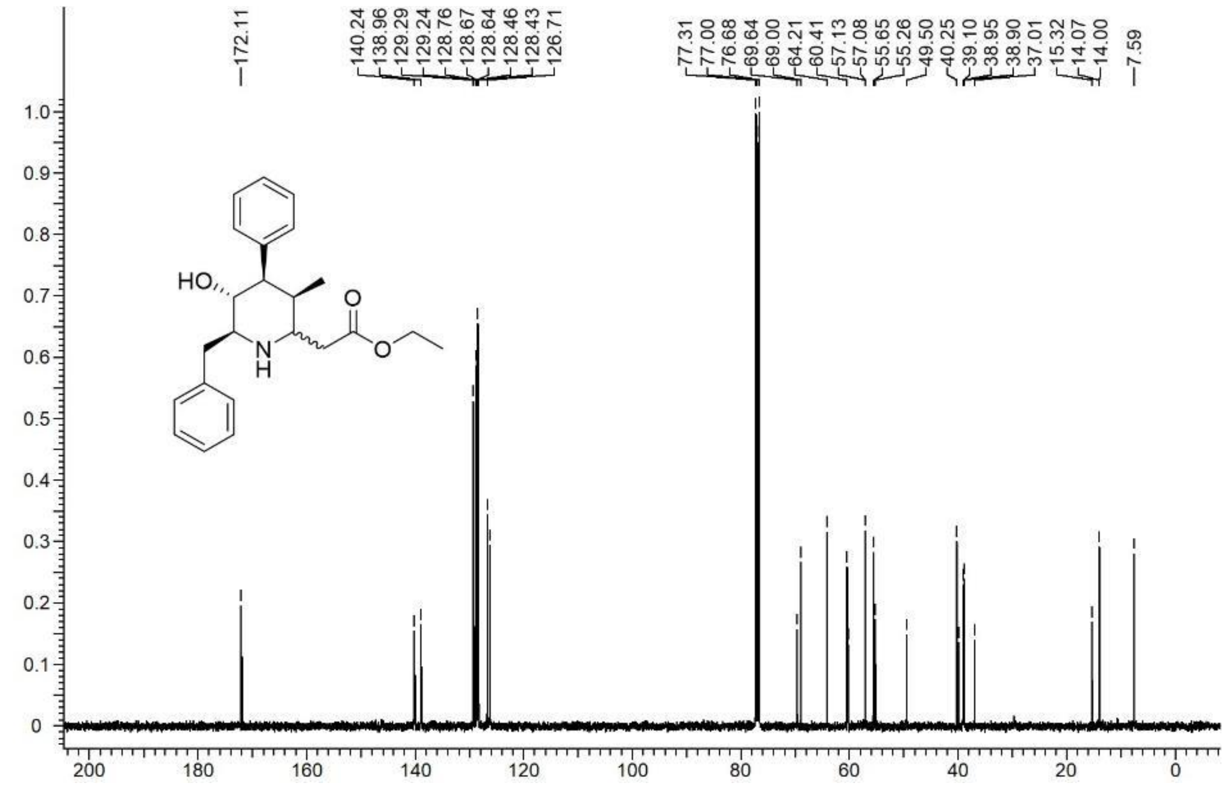

Ethyl 2-[(3*R*,4*R*,5*R*,6*S*)-5-acetoxy-6-benzyl-3-methyl-4-phenylpiperidin-2-yl]acetate (22)
– Diasteromeric mixture 2*R*/2*S*

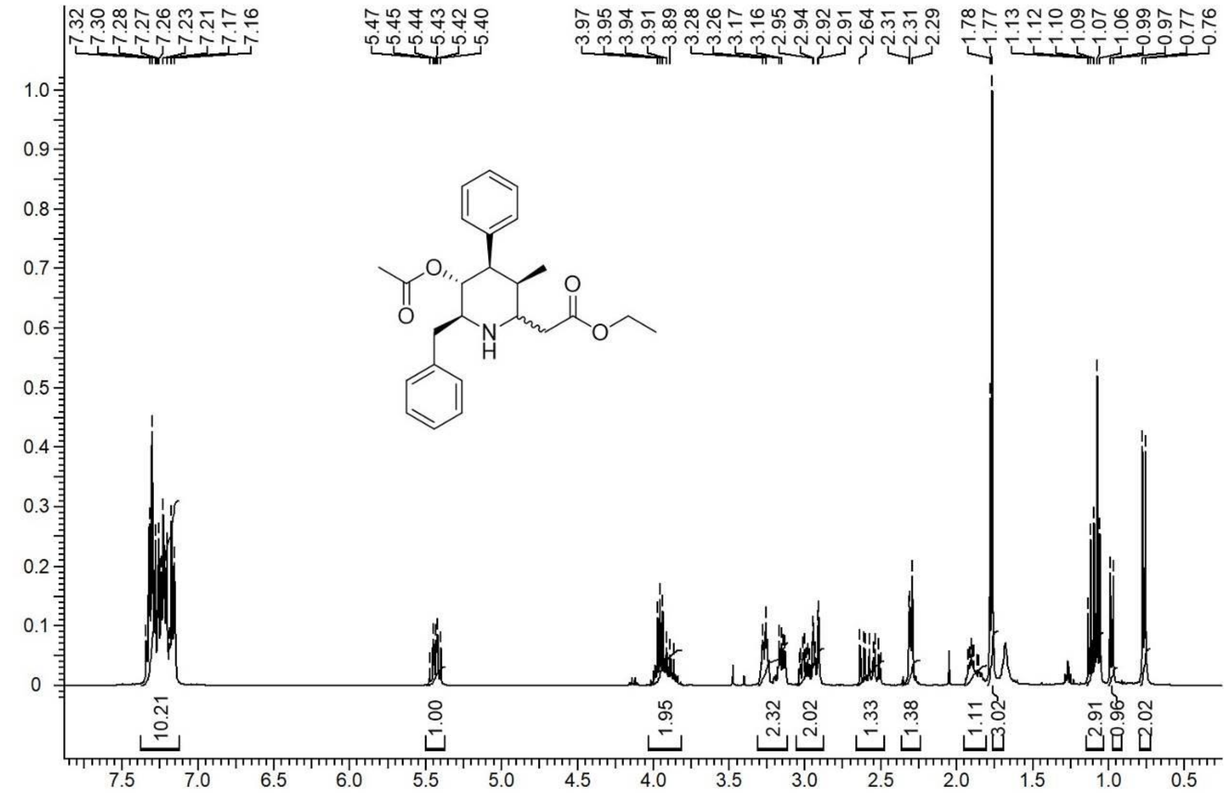

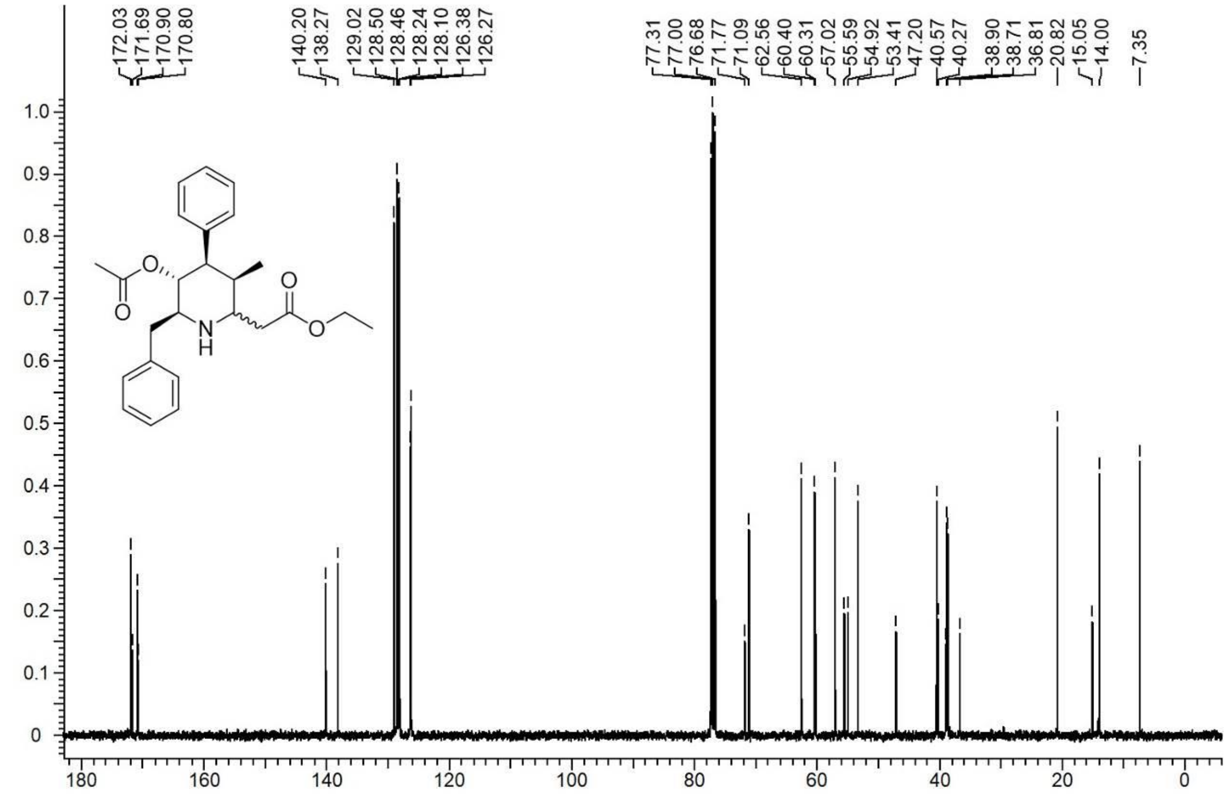

Ethyl 2-[(2*R*,3*R*,4*R*,5*R*,6*S*)-5-acetoxy-6-benzyl-3-methyl-4-phenylpiperidin-2-yl]acetate [(2*R*)-22]
– Major diastereomer

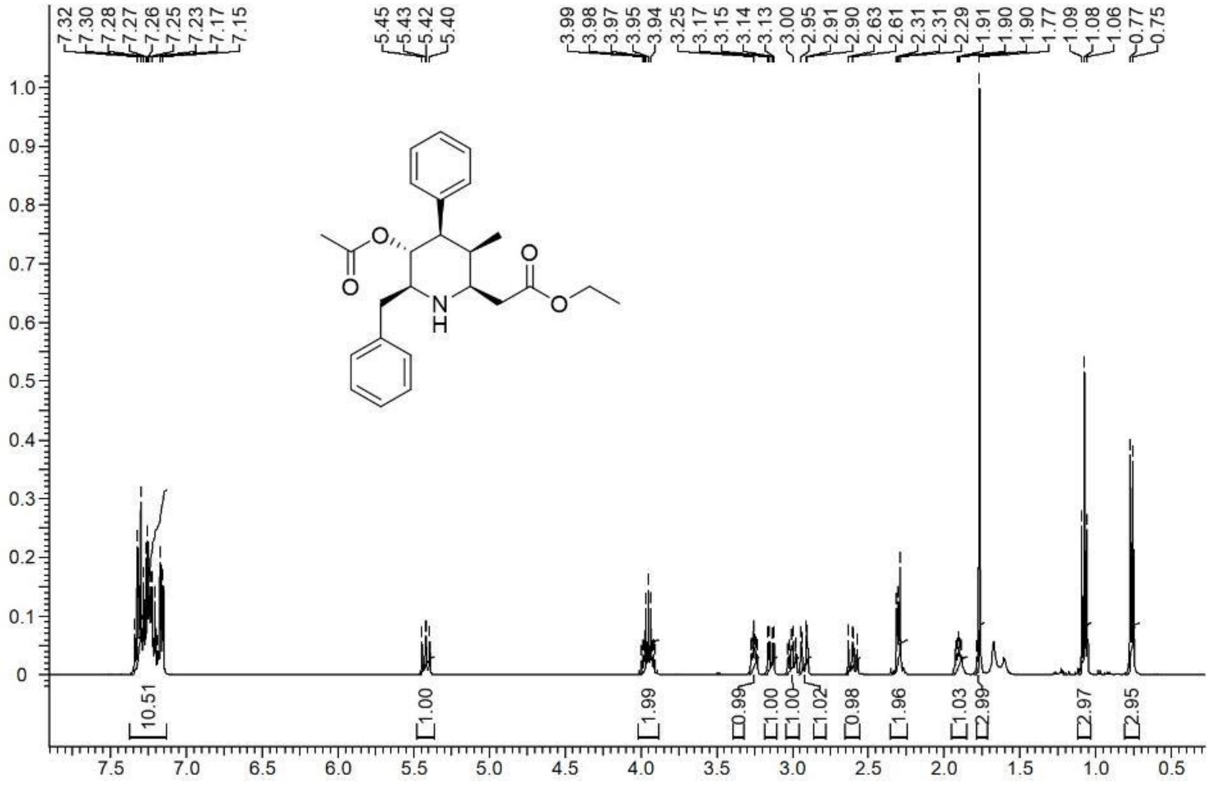

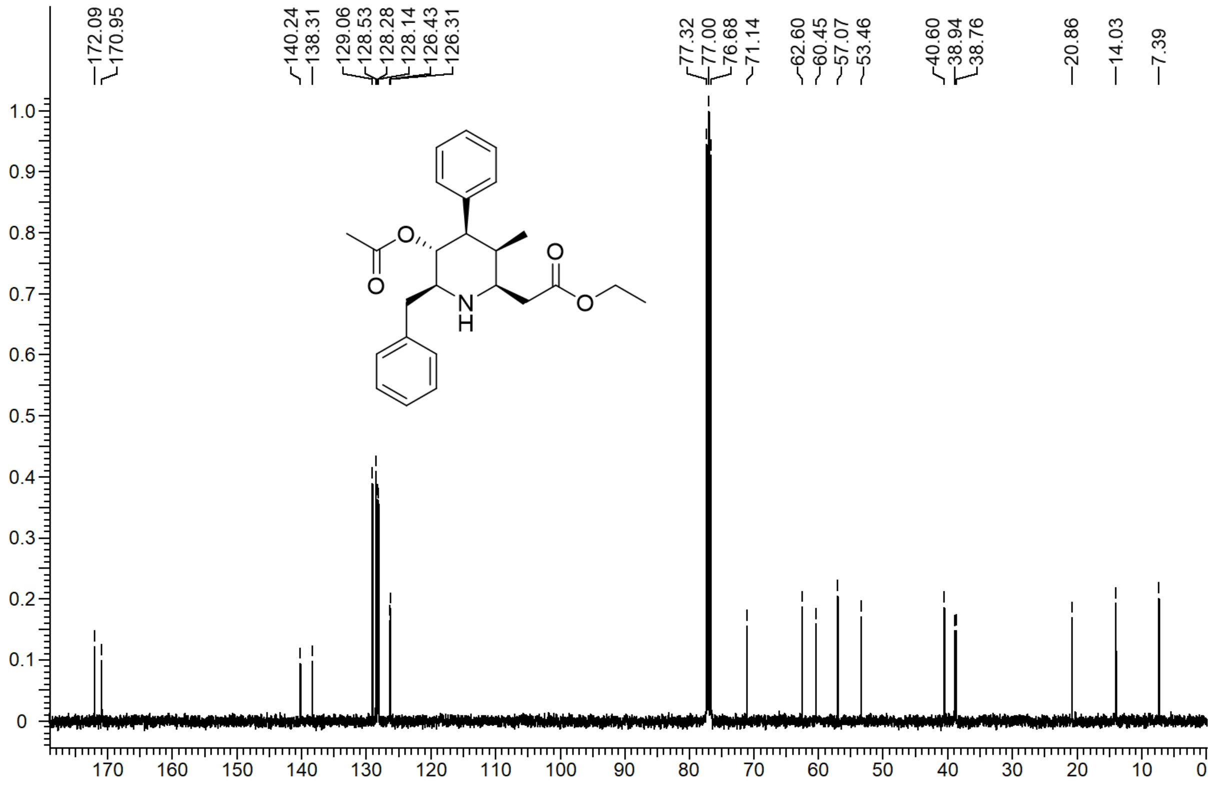

Ethyl 2-[(2*S*,3*R*,4*R*,5*R*,6*S*)-5-acetoxy-6-benzyl-3-methyl-4-phenylpiperidin-2-yl]acetate [(2*S*)-22]

– Minor diastereomer

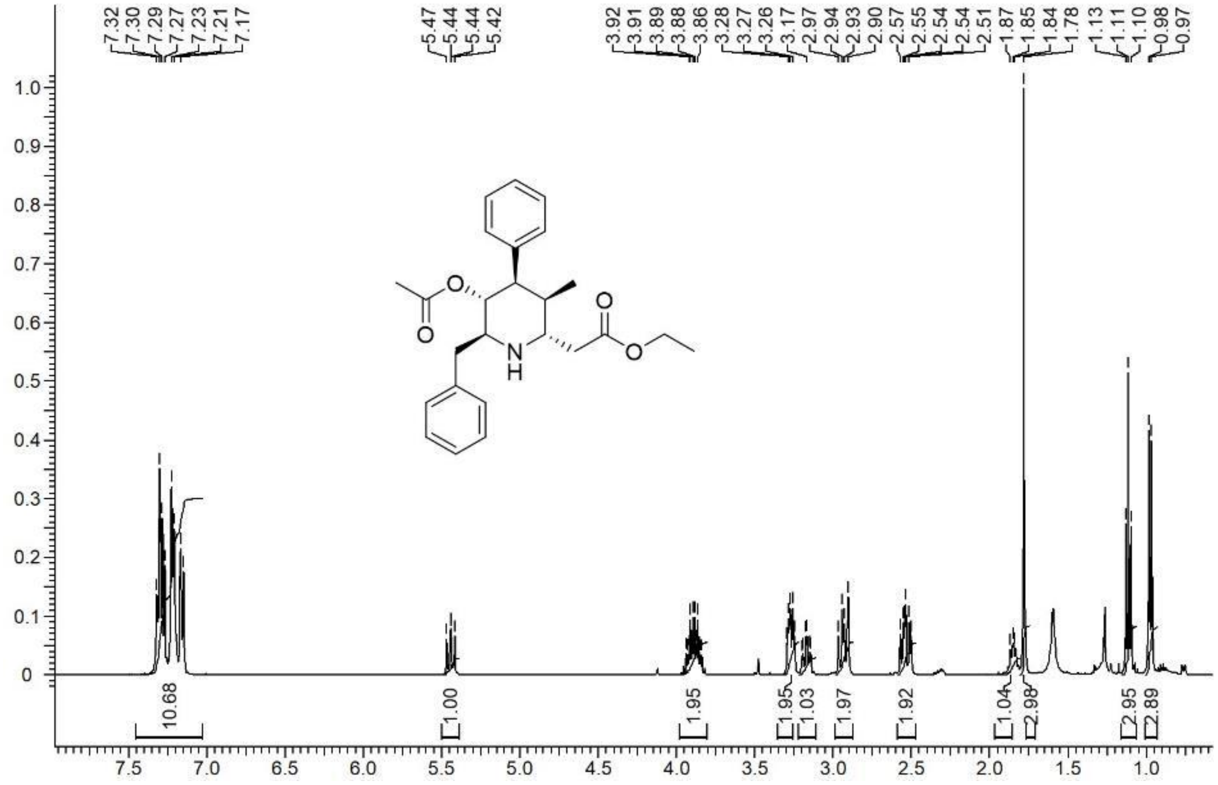

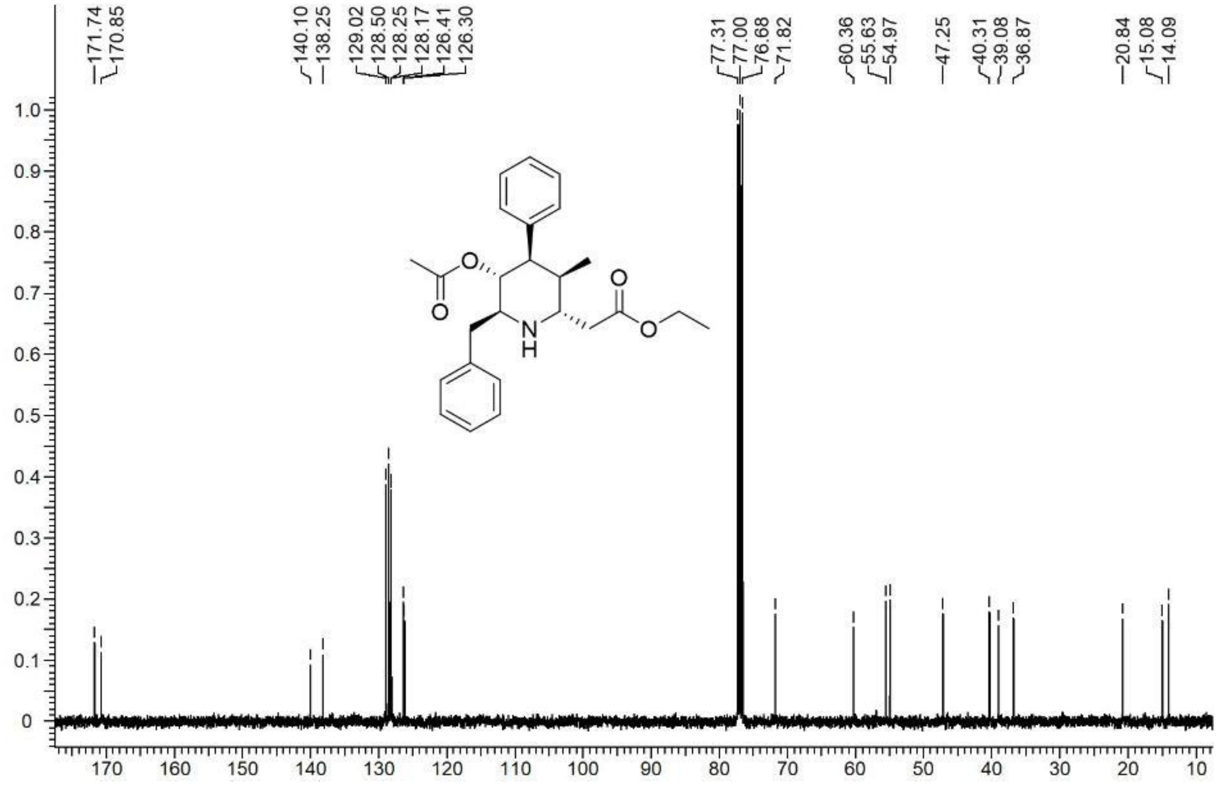

HPLC [(2*R*)-22) & (2*S*)-22]
Column: Reprosil 100 Chiral-NR 8 µm
Eluent: Hexane/iPrOH 95:5, 1.0 ml/min, 20°C, 210 nm

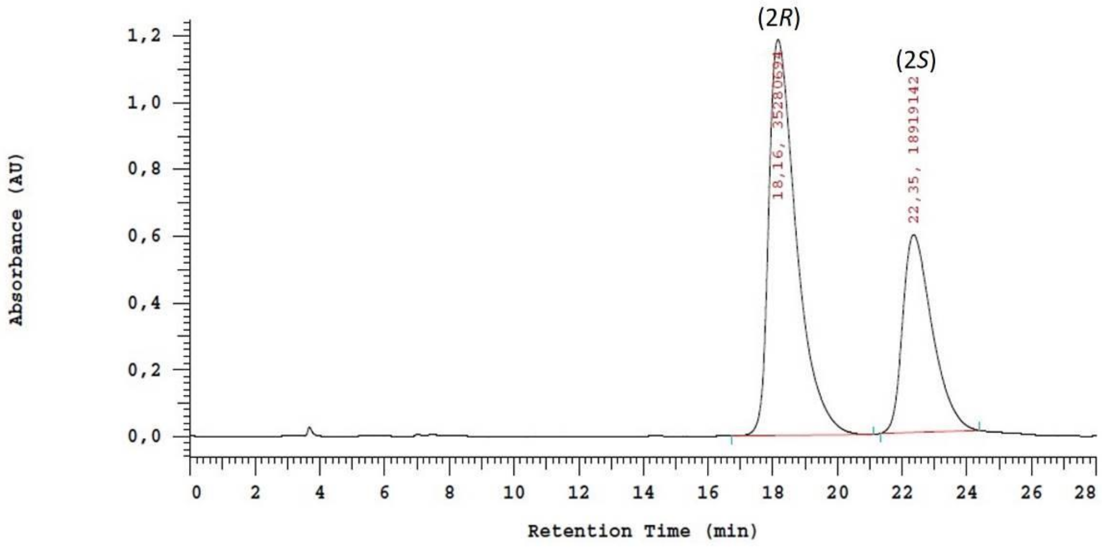

No.	RT	Area	Area %
1	18,16	35280694	65,094
2	22,35	18919142	34,906
		54199836	100,000

2-[(3S,4R,5R,6S)-6-Benzyl-5-hydroxy-3-methyl-4-phenylpiperidin-2-yl]acetonitrile (23a)
– Diastereomeric mixture 2*R*/2*S*

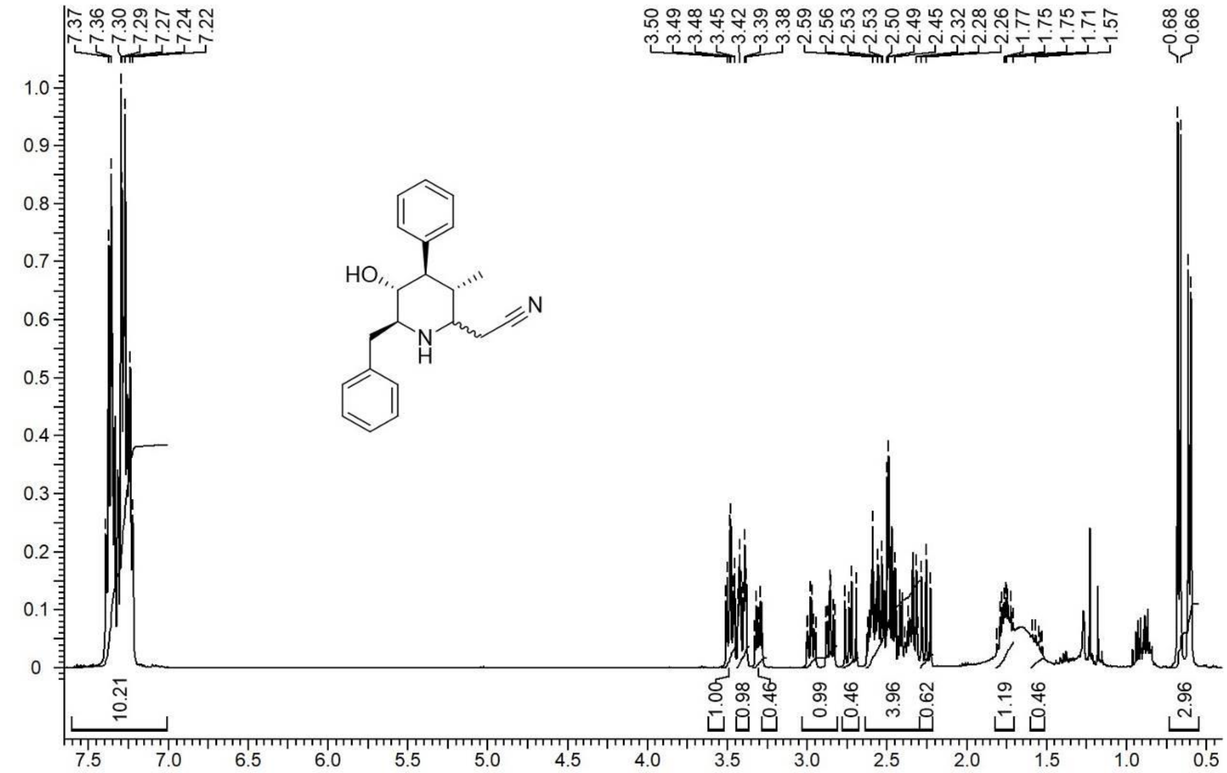

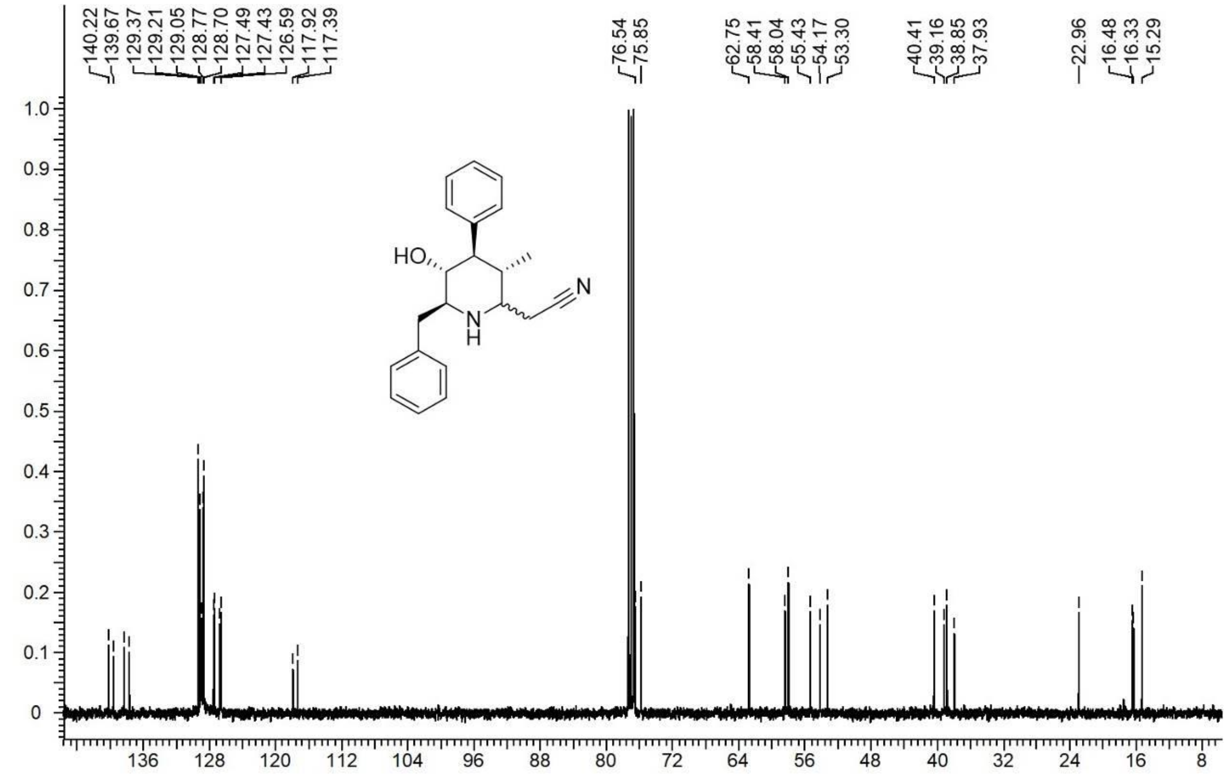

2-[(2*R*,3*S*,4*R*,5*R*,6*S*)-6-Benzyl-5-hydroxy-3-methyl-4-phenylpiperidin-2-yl]acetonitrile [(2*R*)-23a]

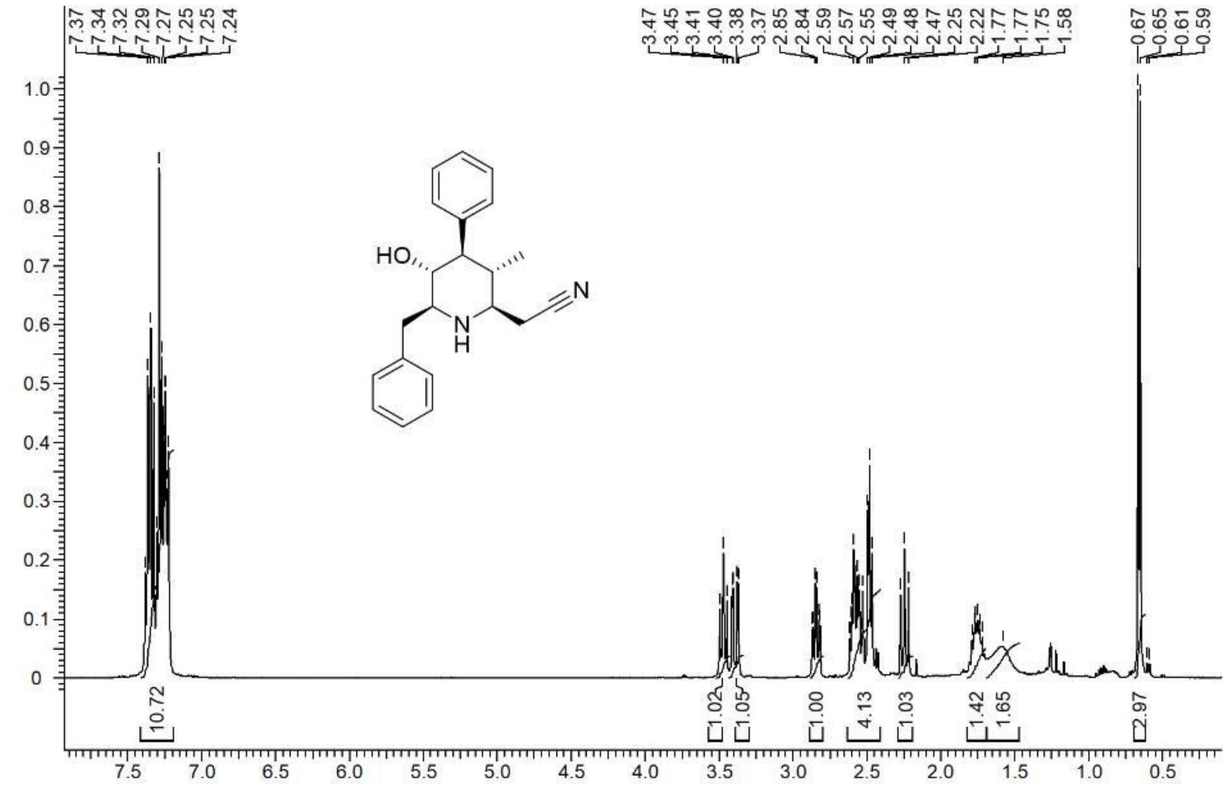

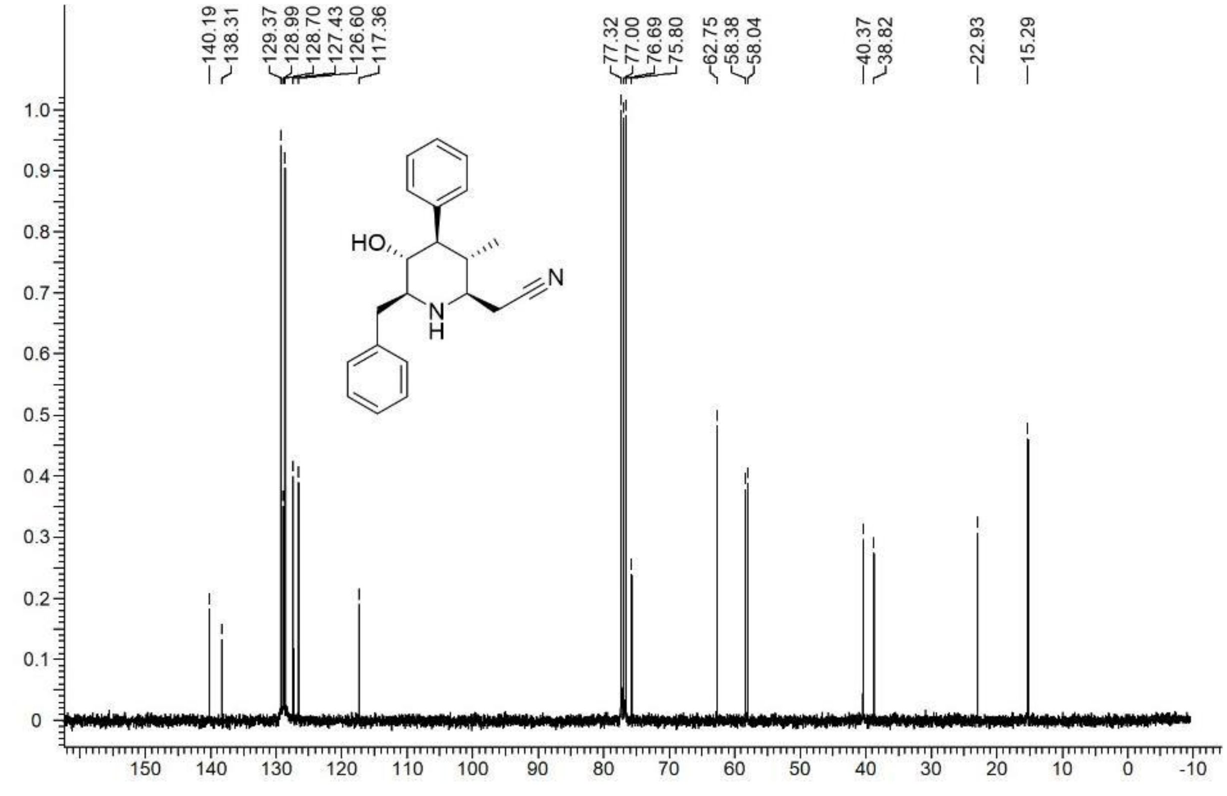

HPLC (23a) from (*E*)-nitrile
Column: Reprosil 100 Chiral-NR 8 μm
Eluent: Hexane/iPrOH 85:15, 1.5 ml/min, 20°C, 210 nm

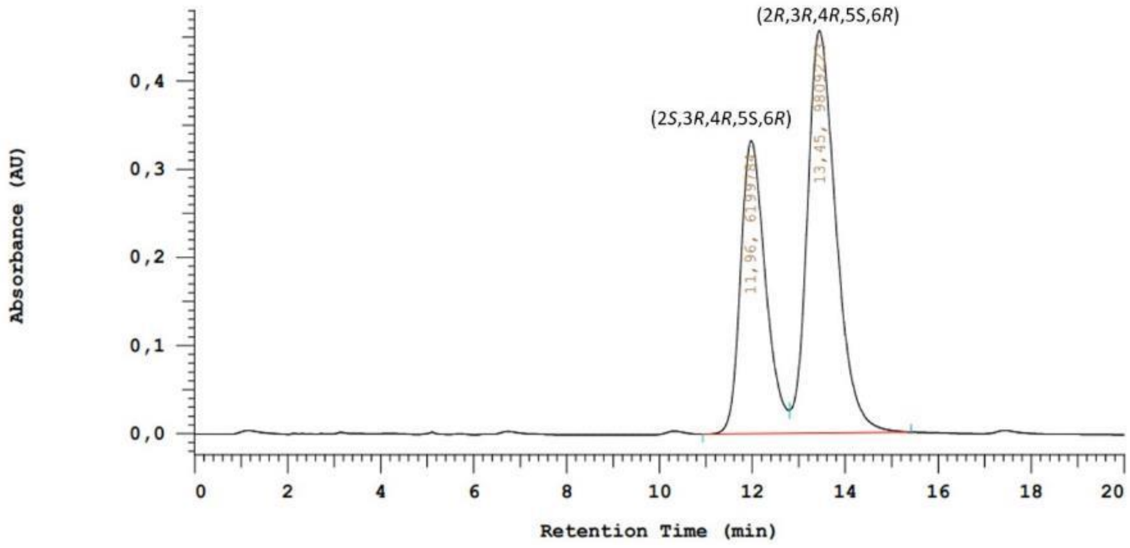

No.	RT	Area	Area %
1	11,96	6199784	38,727
2	13,45	9809223	61,273
		16009007	100,000

HPLC (23a) from (*Z*)-nitrile
Column: Reprosil 100 Chiral-NR 8 μm
Eluent: Hexane/iPrOH 85:15, 1.5 ml/min, 20°C, 210 nm

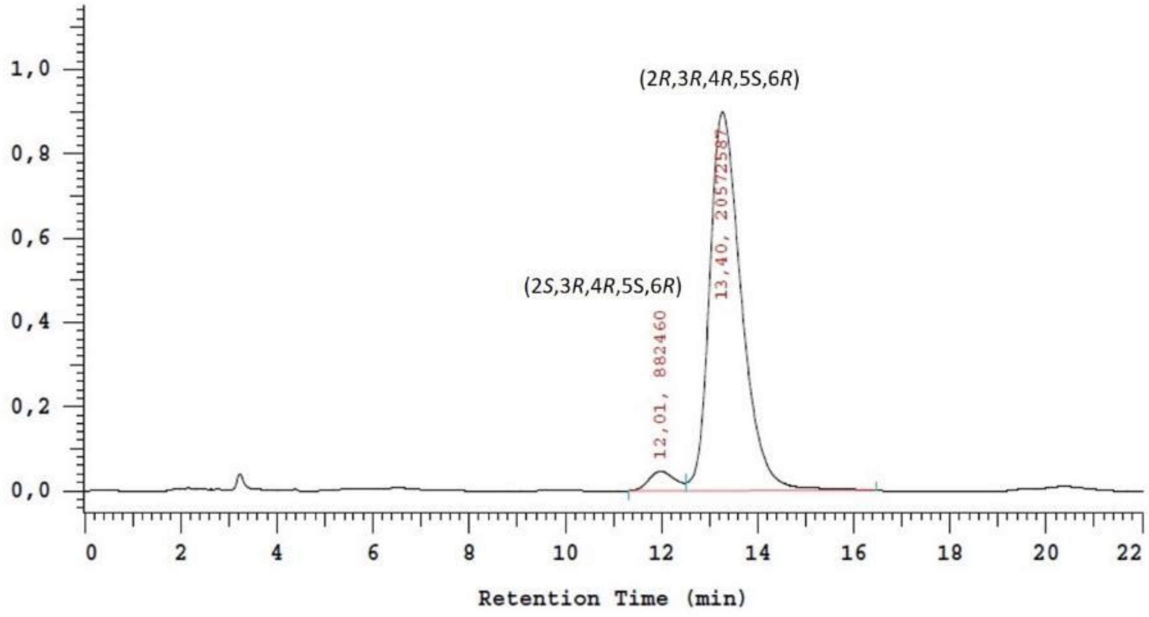

No.	RT	Area	Area %
1	12,01	882460	4,113
2	13,40	20572587	95,887
		21455047	100,000

2-[(3*R*,4*R*,5*R*,6*S*)-6-Benzyl-5-hydroxy-3-methyl-4-phenylpiperidin-2-yl]acetonitrile (23b)
– Diastereomeric mixture 2*R*/2*S*

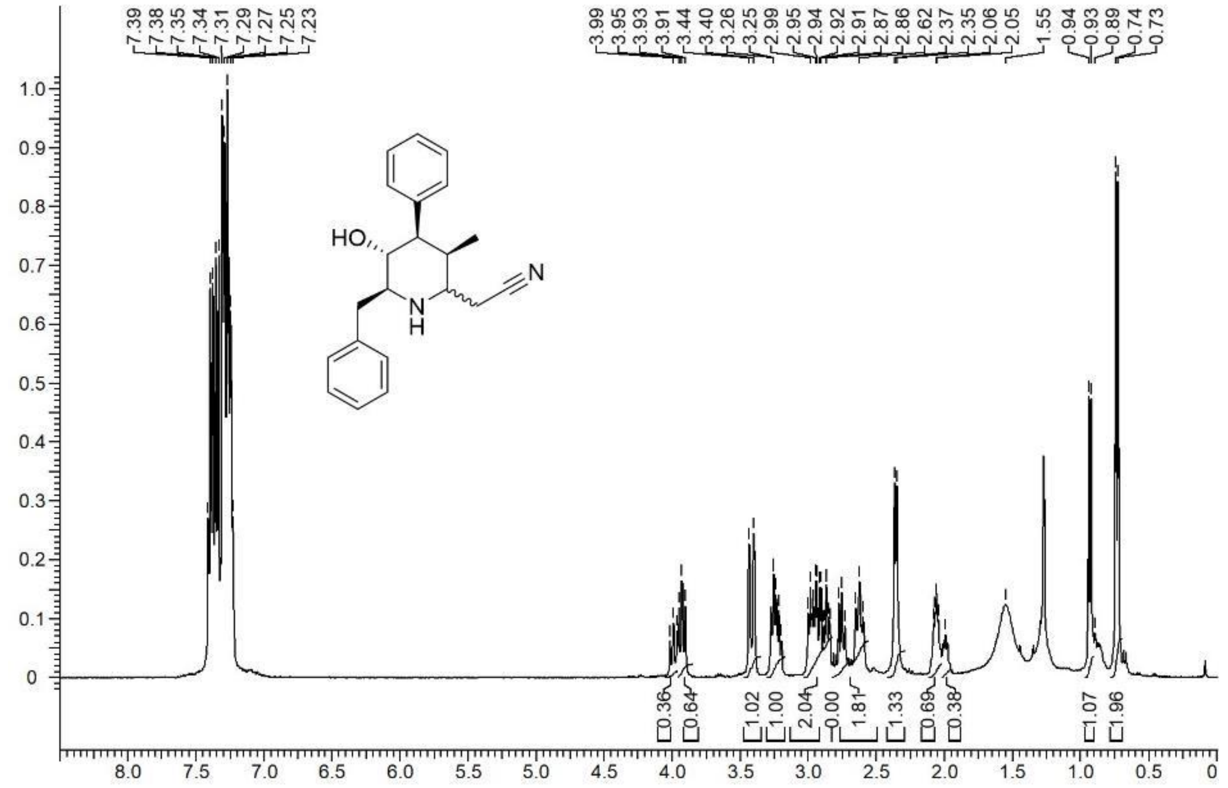

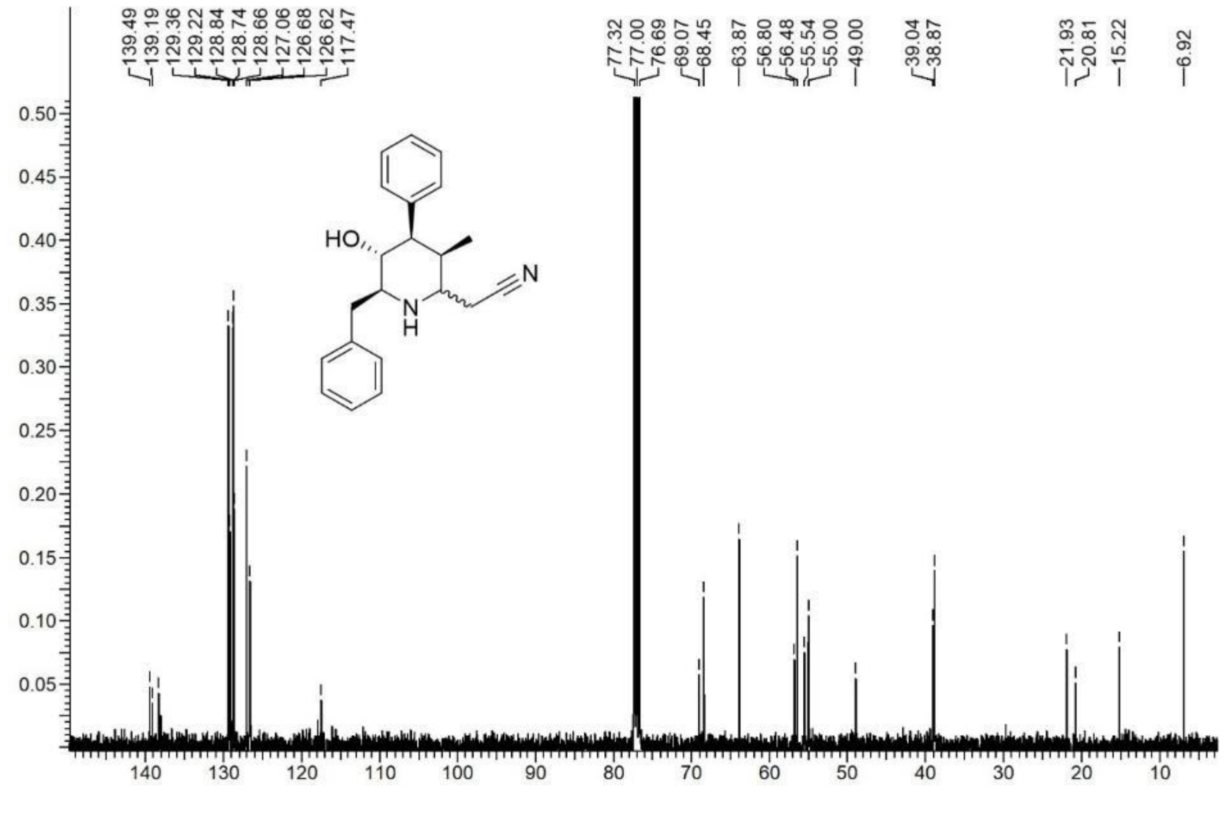

HPLC (23b) from (*E*)-nitrile
Column: Reprosil 100 Chiral-NR 8 µm
Eluent: Hexane/iPrOH 85:15, 1.5 ml/min, 20°C, 210 nm

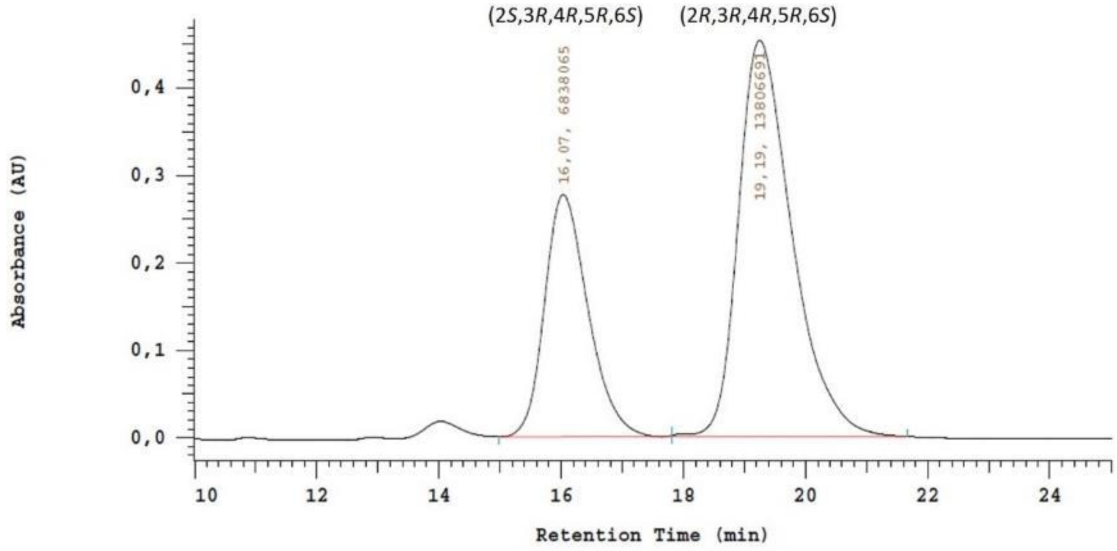

No.	RT	Area	Area %
1	16,07	6838065	33,123
2	19,19	13806691	66,877
		20644756	100,000

HPLC (23b) from (*Z*)-nitrile
Column: Reprosil 100 Chiral-NR 8 µm
Eluent: Hexane/iPrOH 85:15, 1.5 ml/min, 20°C, 210 nm

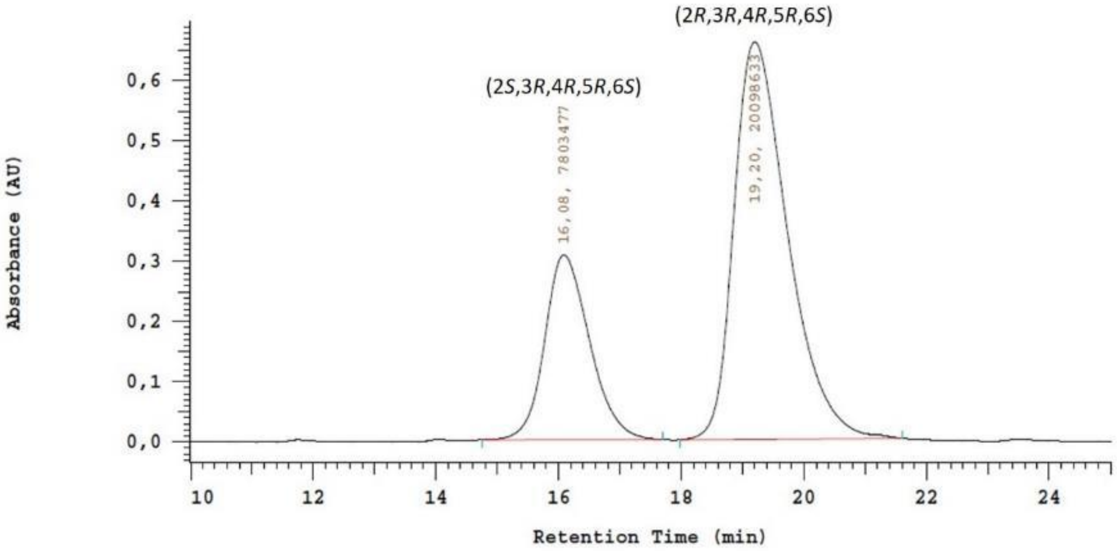

No.	RT	Area	Area %
1	16,08	7803477	27,967
2	19,20	20098633	72,033
		27902110	100,000

2-[(3S,4R,5R,6S)-5-Acetoxy-6-benzyl-3-methyl-4-phenylpiperidin-2-yl]acetonitrile (24a)
− Diastereomeric mixture 2*R*/2*S*

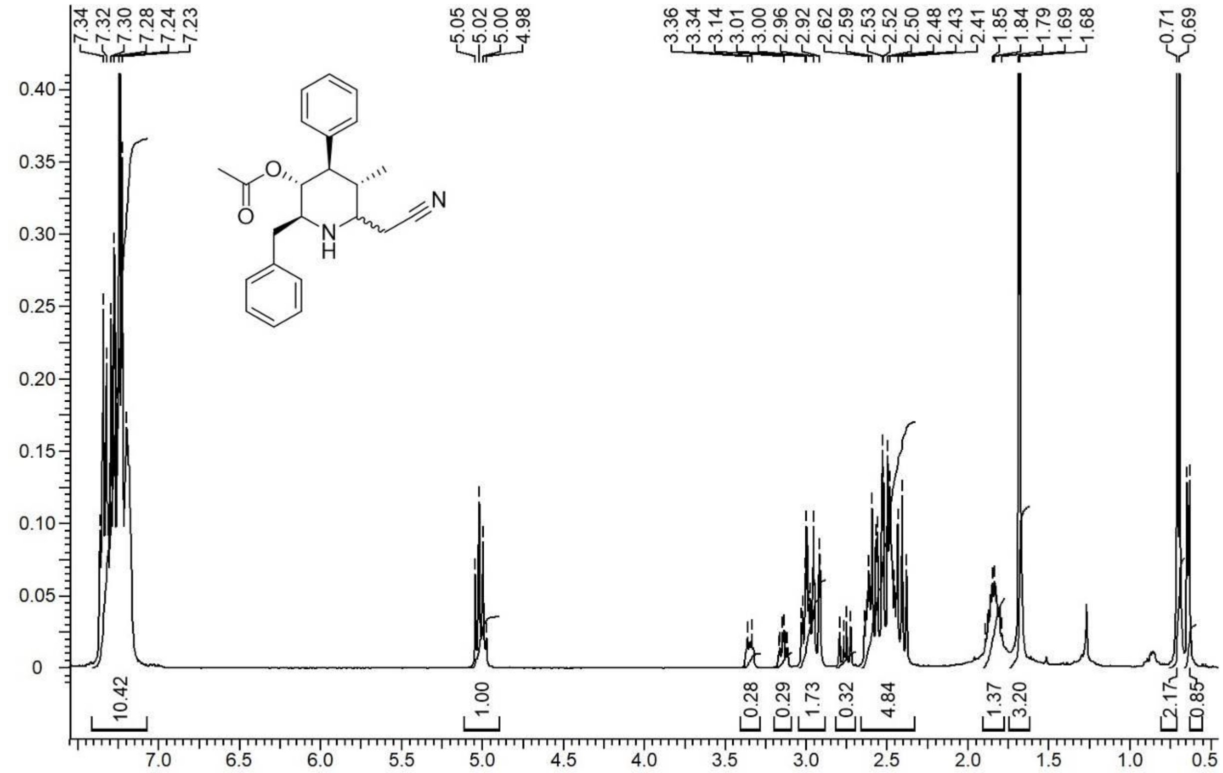

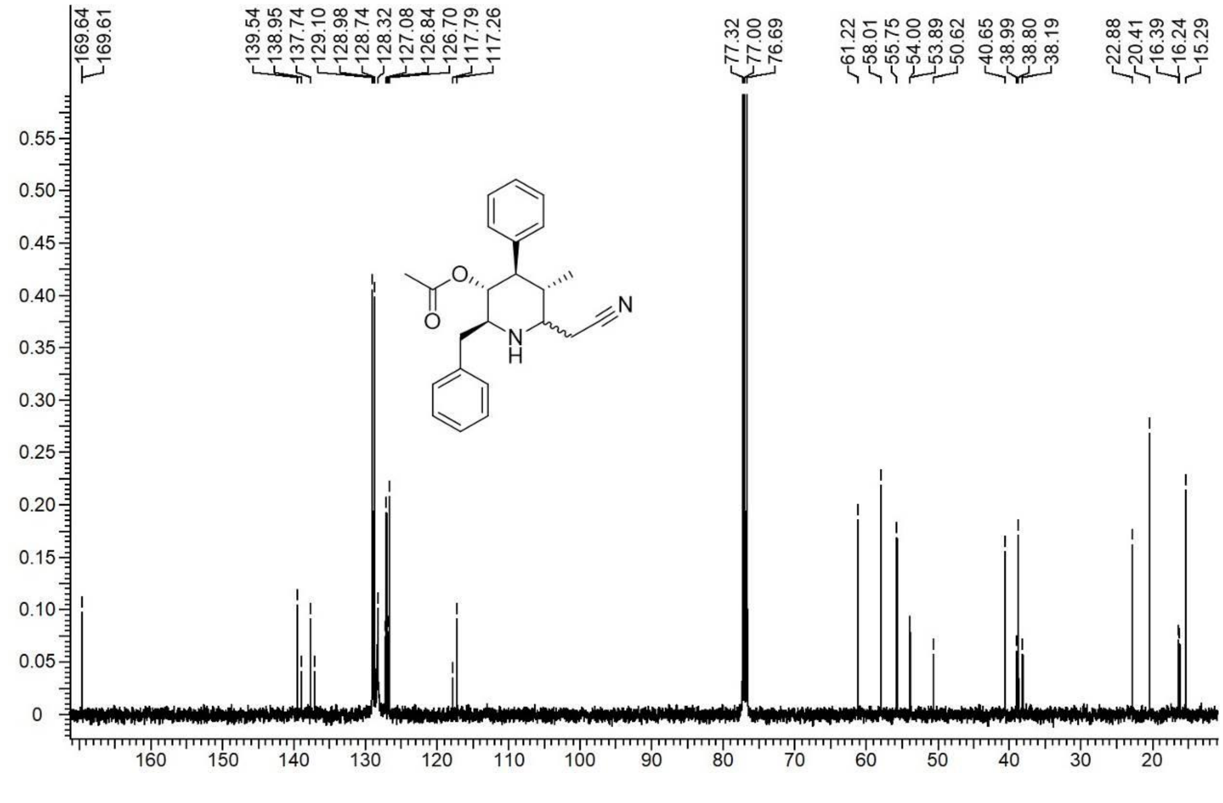

2-[(2*R*,3*S*,4*R*,5*R*,6*S*)-5-Acetoxy-6-benzyl-3-methyl-4-phenylpiperidin-2-yl]acetonitrile [(2*R*)-24a] – Major Diastereomer

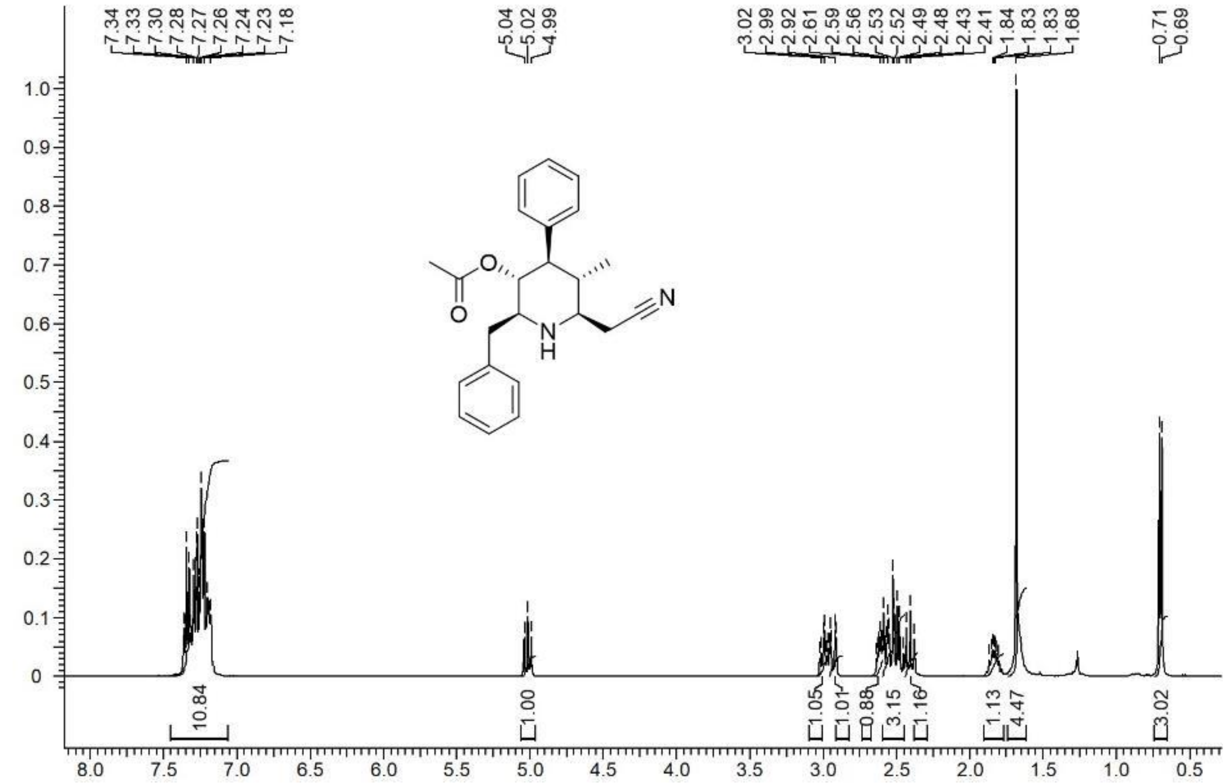

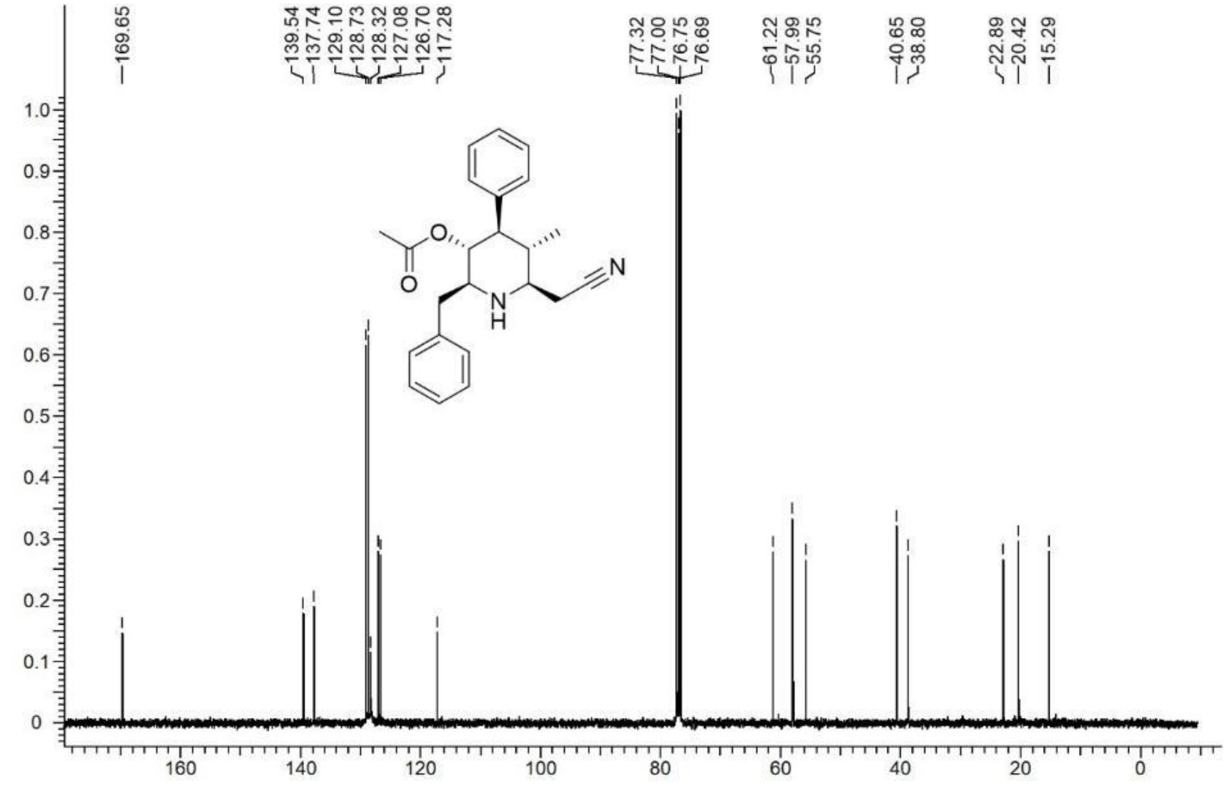

2-[(2*S*,3*S*,4*R*,5*R*,6*S*)-5-Acetoxy-6-benzyl-3-methyl-4-phenylpiperidin-2-yl]acetonitrile [(2*S*)-24a] – Minor Diastereomer

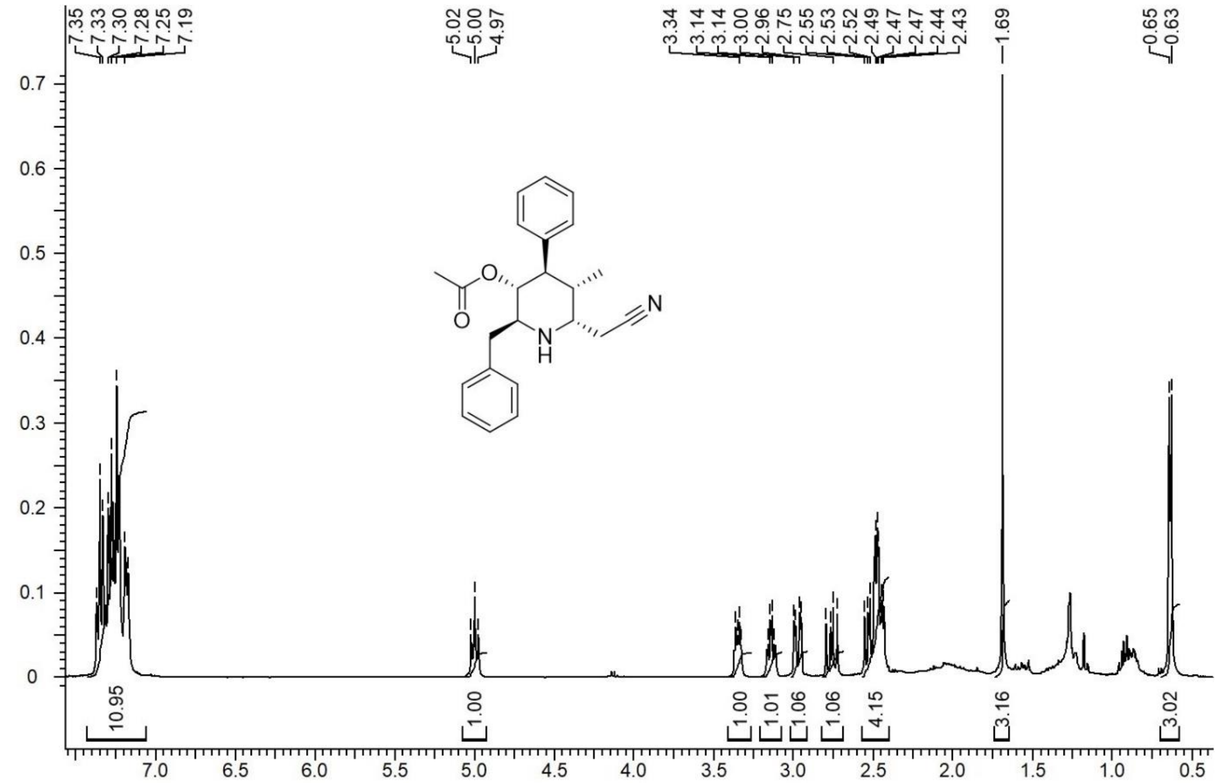

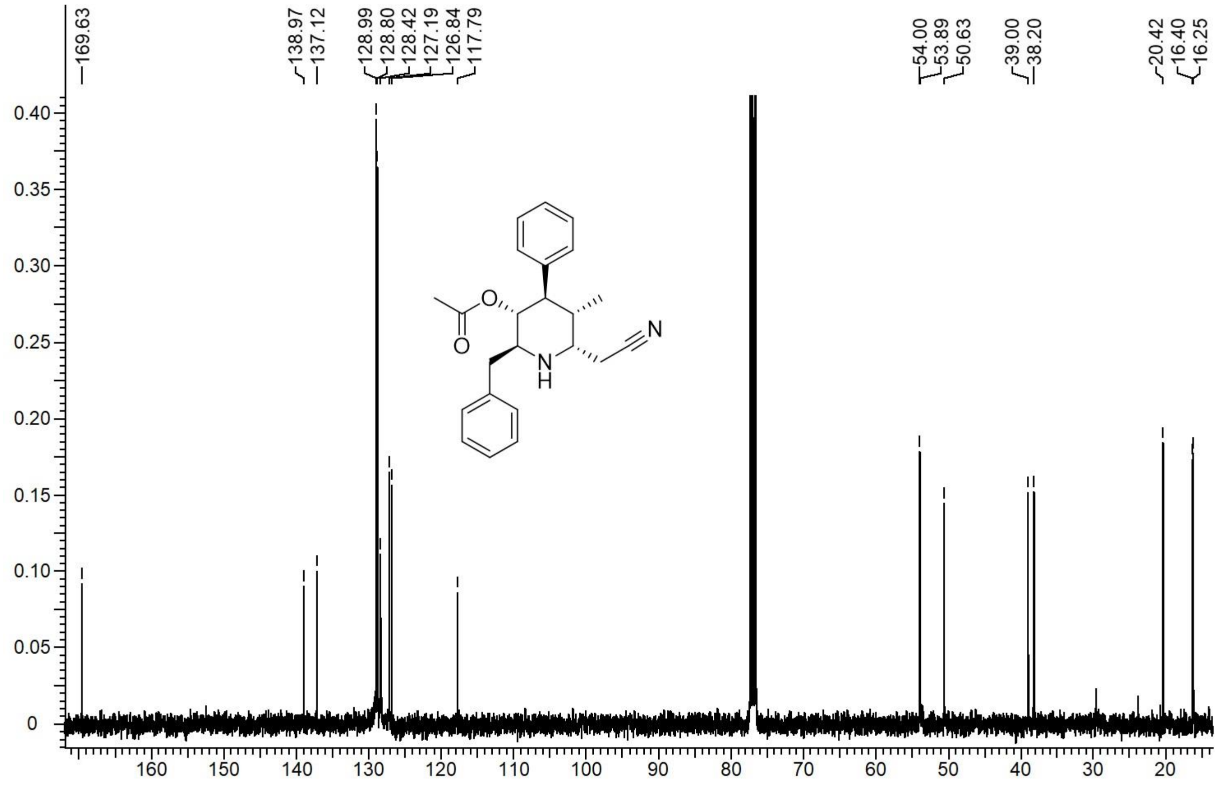

HPLC (24a) from (*E*)-nitrile
Column: Reprosil 100 Chiral-NR 8 µm
Eluent: Hexane/iPrOH 70:30, 1.5 ml/min, 20°C, 210 nm

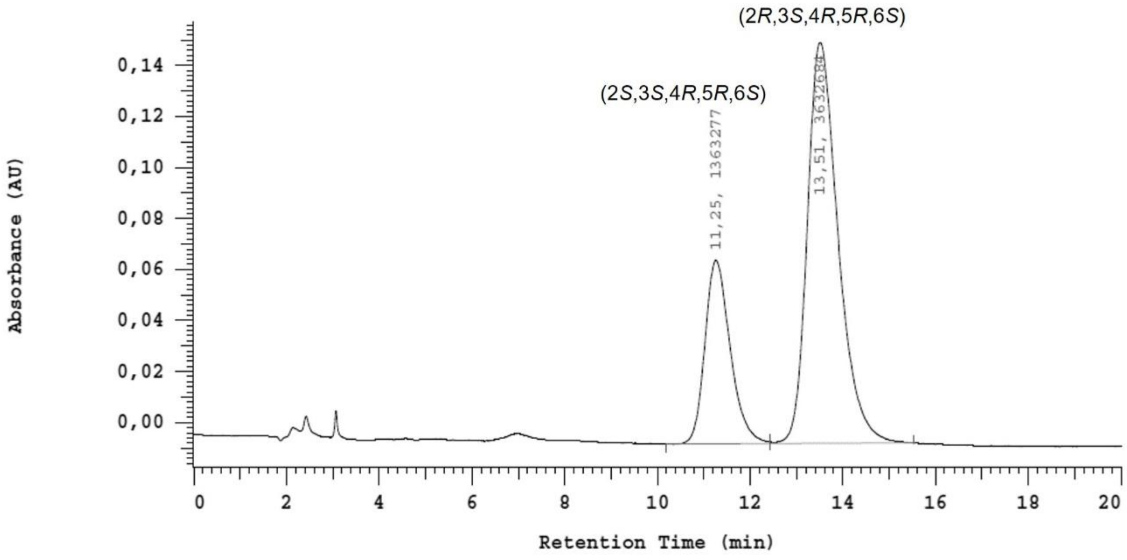

No.	RT	Area	Area %
1	11,25	1363277	27,288
2	13,51	3632684	72,712
		4995961	100,000

HPLC (24a) from (*Z*)-nitrile
Column: Reprosil 100 Chiral-NR 8 µm
Eluent: Hexane/iPrOH 70:30, 1.5 ml/min, 20°C, 210 nm

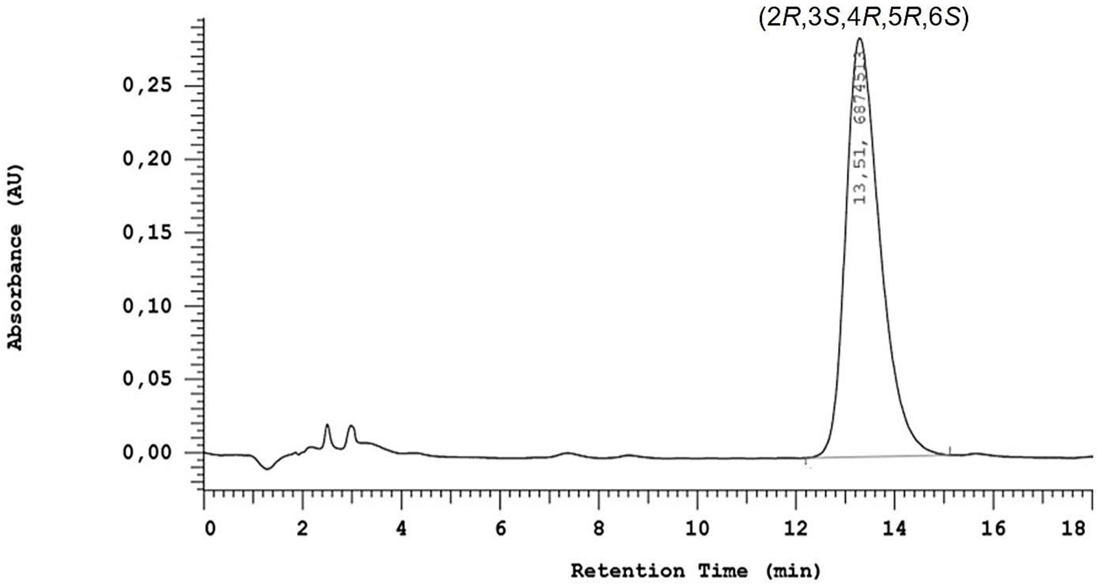

No.	RT	Area	Area %
1	13,51	6874513	100,000
		6874513	100,000

2-[(3*S*,4*R*,5*R*,6*S*)-5-Acetoxy-6-benzyl-3-methyl-4-phenylpiperidin-2-yl]acetonitrile (24b)

− Diastereomeric mixture 2*R*/2*S*

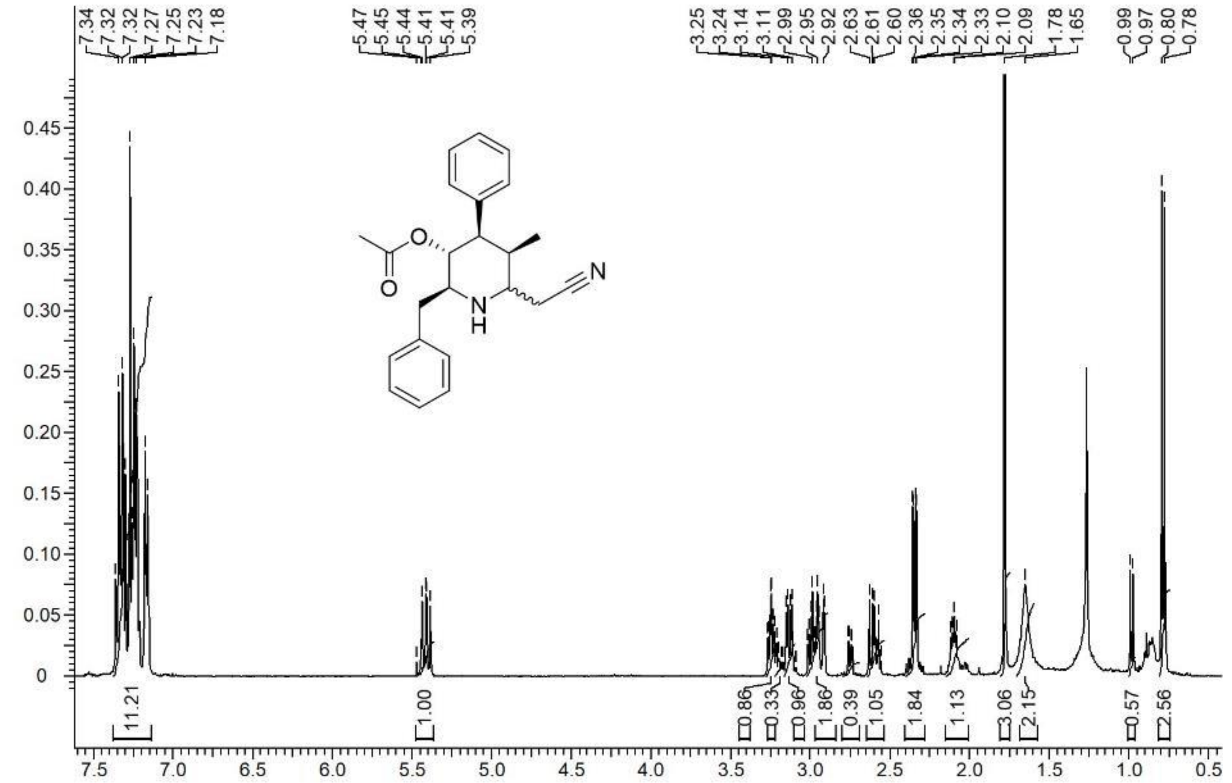

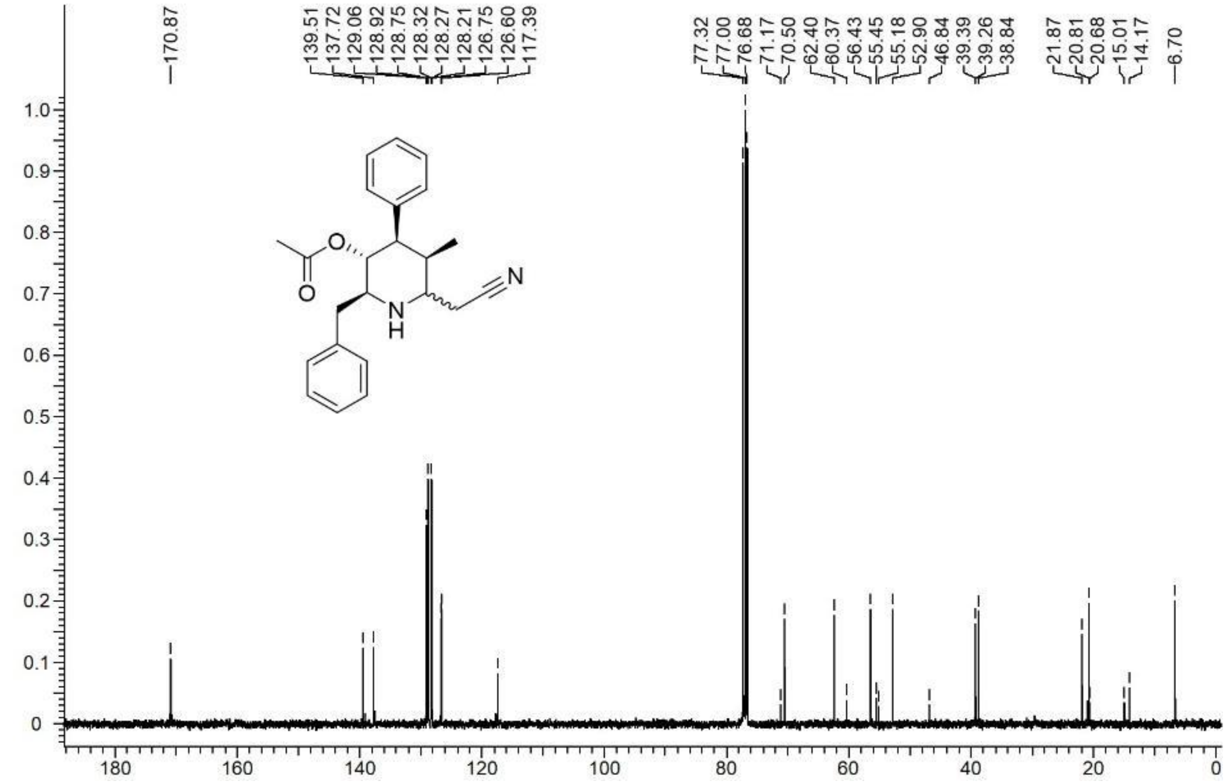

2-[(2*R*,3*S*,4*R*,5*R*,6*S*)-5-Acetoxy-6-benzyl-3-methyl-4-phenylpiperidin-2-yl]acetonitrile [(2*R*)-24b]

− Major diastereomer

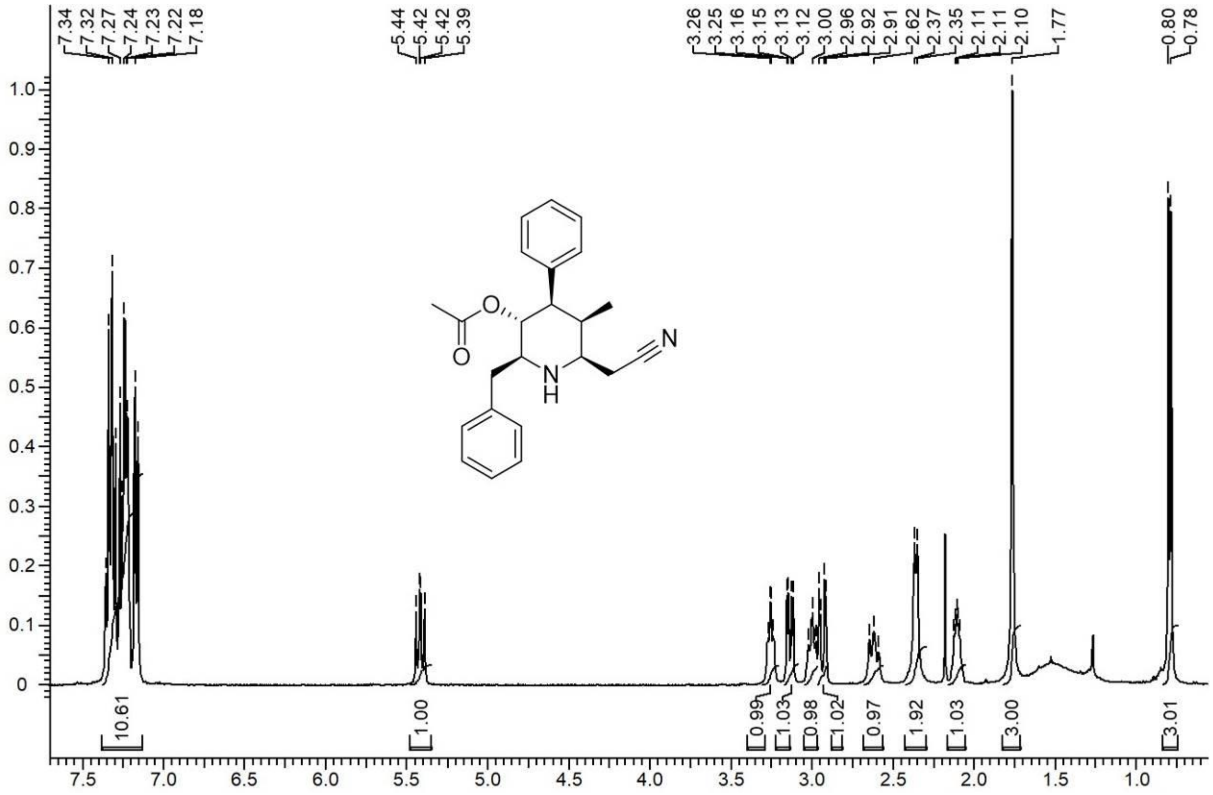

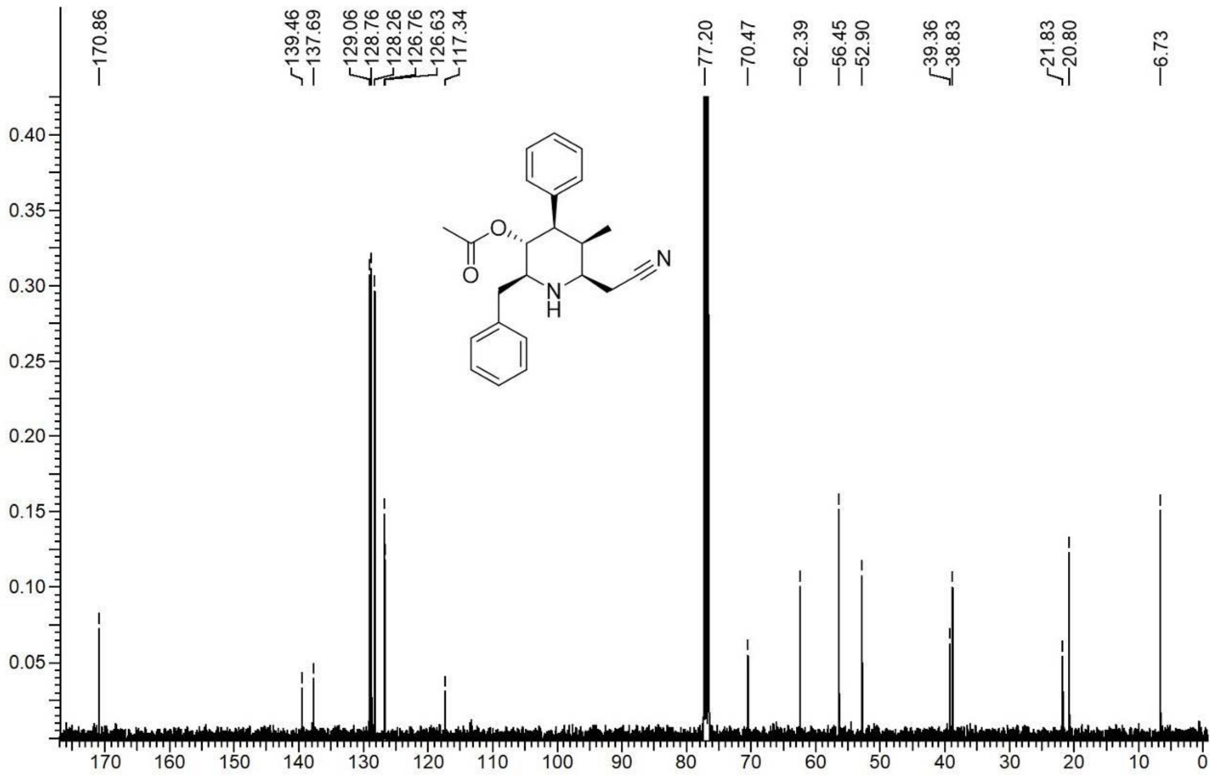

2-[(2*S*,3*S*,4*R*,5*R*,6*S*)-5-Acetoxy-6-benzyl-3-methyl-4-phenylpiperidin-2-yl]acetonitrile [(2*S*)-24b]

− Minor diastereomer

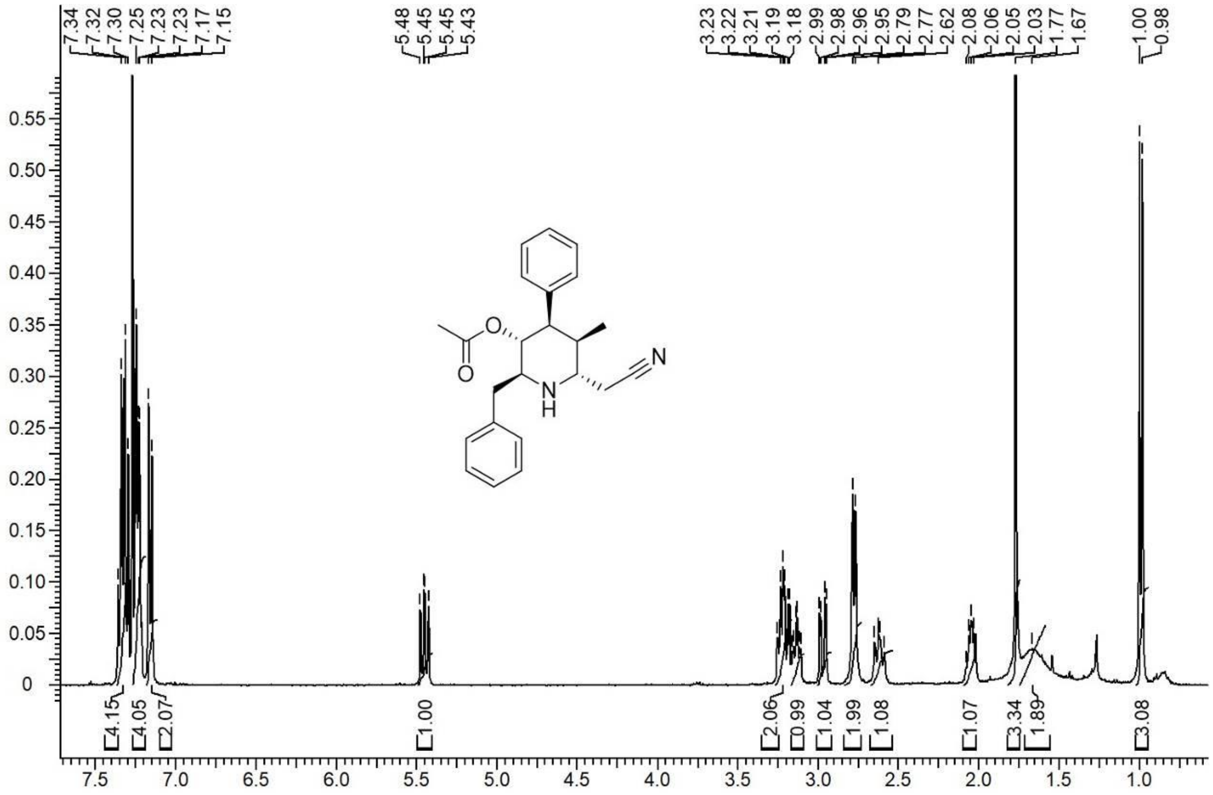

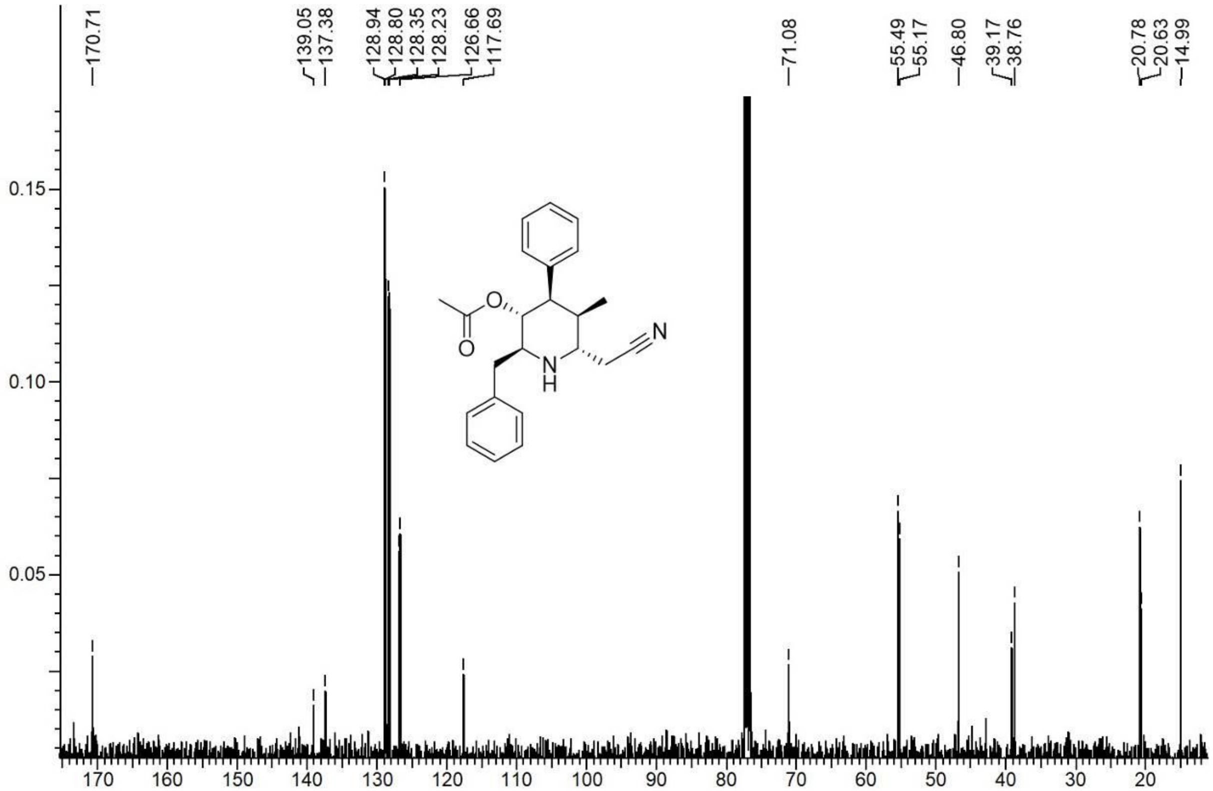

HPLC (24b) from (*E*)-nitrile
Column: Reprosil 100 Chiral-NR 8 µm
Eluent: Hexane/iPrOH 85:15, 1.0 ml/min, 20°C, 210 nm

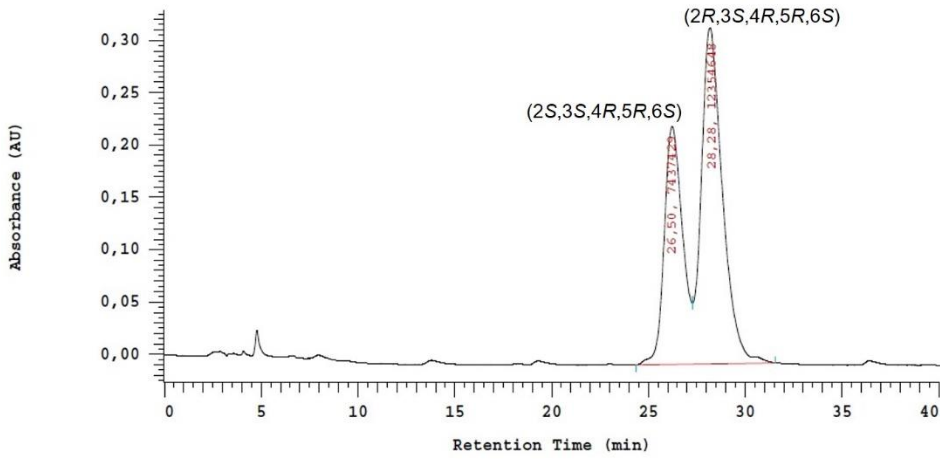

No.	RT	Area	Area %
1	26,50	7437429	37,578
2	28,28	12354648	62,422
		19792077	100,000

HPLC (24b) from (*Z*)-nitrile
Column: Reprosil 100 Chiral-NR 8 µm
Eluent: Hexane/iPrOH 85:15, 1.0 ml/min, 20°C, 210 nm

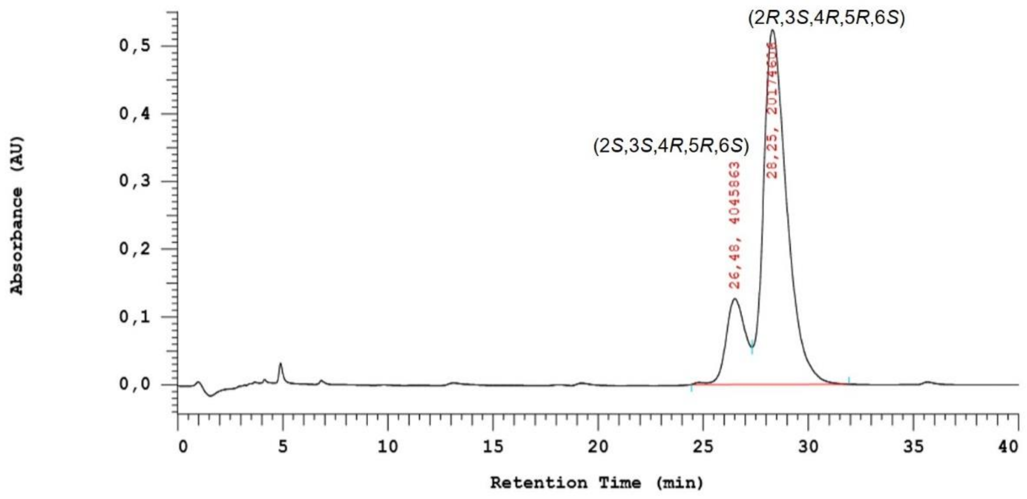

No.	RT	Area	Area %
1	26,48	4045863	16,704
2	28,25	20174606	83,296
		24220469	100,000
